# Ellagic Acid Attenuates Gentamicin Nephrotoxicity by Integrated Modulation of ER Stress-Associated Apoptosis-Autophagy Crosstalk and Attenuation of Nrf2/HO-1 Signaling

**DOI:** 10.3390/biomedicines14061385

**Published:** 2026-06-19

**Authors:** Azad Salimi, Mohammad Javad Khoshnoud, Forouzan Khodaei Halani, Shekoofeh Jokar, Samaneh Bina, Seyyed Sajad Daneshi, Marziyeh Haghshenas, Marzieh Rashedinia

**Affiliations:** 1 Department of Pharmacology and Toxicology, School of Pharmacy, Shiraz University of Medical Sciences, Shiraz 71348-14336, Iran; azadsalimi69@gmail.com (A.S.); khoshnoudm3@gmail.com (M.J.K.); foroozan_009@yahoo.com (F.K.H.); ph.jokar@gmail.com (S.J.); mhagh1055@gmail.com (M.H.); 2 Student Research Committee, Shiraz University of Medical Sciences, Shiraz 71348-14336, Iran; 3 Department of Biology, Marvdasht Branch, Islamic Azad University, Marvdasht 73711-13119, Iran; sbina.biology@gmail.com; 4 Department of Anatomy, School of Medicine, Shiraz University of Medical Sciences, Shiraz 71348-14336, Iran; daneshi.sajad99@gmail.com; 5 Medicinal Plants Processing Research Center, Shiraz University of Medical Sciences, Shiraz 71348-14336, Iran; 6 Food and Drug Research Center, Food and Drug Administration, Ministry of Health and Medical Education, Tehran 14676-64961, Iran

**Keywords:** ellagic acid, gentamicin-induced nephrotoxicity, endoplasmic reticulum stress, Nrf2/HO-1 signaling, apoptosis-autophagy crosstalk, IL-6

## Abstract

**Background:** Gentamicin-induced nephrotoxicity limits clinical pharmacotherapy and involves multiple converging stress-response pathways. Ellagic acid (EA) has renoprotective potential, yet its role in coordinating endoplasmic reticulum (ER) stress-mediated apoptosis, autophagy, and inflammation remains unclear. We hypothesized that EA co-treatment would protect the kidney by modulating ER stress-dependent pathways and associated inflammatory and adaptive signaling. **Methods:** For an integrated mechanistic analysis in a rat model of gentamicin nephrotoxicity, 40 male Sprague-Dawley rats were assigned to control, gentamicin (100 mg/kg), EA (100 mg/kg), and gentamicin + EA groups for 14 days. Renal function, oxidative stress, inflammatory mediators, ER stress markers, apoptosis, autophagy, tubular injury markers, and histopathological changes were assessed. **Results:** Gentamicin induced renal dysfunction, tubular injury, and ER stress across all unfolded protein response (UPR) branches (*IRE1α*, *PERK*, *ATF6*), C/EBP homologous protein (*CHOP*)-associated apoptosis, dysregulated autophagy, and upregulated kidney injury molecule-1 (KIM-1). A selective inflammatory signature was observed, with increased cyclooxygenase-2 (*COX-2*) and interleukin-6 (IL-6), whereas tumor necrosis factor-alpha (TNF-α) and interleukin-1 beta (IL-1β) remained unchanged. Co-administration of ellagic acid with gentamicin significantly improved renal function markers compared to the gentamicin group. In contrast, ellagic acid alone did not show significant differences compared to the control group. Notably, gentamicin induced compensatory upregulation of nuclear factor erythroid 2-related factor 2 (*Nrf2*)/*heme oxygenase-1* (*HO-1*) expression, while ellagic acid co-treatment attenuated this compensatory upregulation, likely secondary to reduced oxidative stress burden. **Conclusions:** This study provides integrated evidence that ER stress is closely associated with gentamicin nephrotoxicity. The key novel findings include selective suppression of IL-6, modulation of the apoptosis-autophagy balance, and attenuation of *Nrf2/HO-1* signaling without direct reactive oxygen species (ROS) scavenging, demonstrating a multi-target framework for EA’s renoprotective effects. These findings suggest that ellagic acid mitigates renal injury in a context-dependent manner rather than confirming a direct causal mechanism.

## 1. Introduction

Gentamicin (GEN), an aminoglycoside antibiotic with broad-spectrum activity against Gram-negative bacteria, remains widely used for its rapid action, chemical stability, and cost-effectiveness [1]. However, its clinical utility is frequently limited by nephrotoxicity [2]. GEN selectively accumulates in the renal cortex, where it disrupts mitochondrial function by inhibiting oxidative phosphorylation, depleting ATP stores, and generating reactive oxygen species (ROS) [3,4]. This oxidative stress triggers lipid peroxidation and initiates inflammatory cascades, collectively driving tubular injury and renal dysfunction [1]. GEN treatment increases the expression of TNF-α, IL-1β, and MCP-1 in kidney tissue and simultaneously activates NF-κB pathways in response to oxidative stress [5]. GEN also induces apoptosis via increased *Bax* and *caspase-3*, decreased *Bcl-2*, mitochondrial dysfunction, and cytochrome-c release [6]. Autophagy, a lysosome-mediated degradation process, is concurrently dysregulated, with differential effects on LC3A and LC3B isoforms in tubular cells [7,8,9]. Kidney Injury Molecule-1 (KIM-1), a proximal tubular injury biomarker, is markedly upregulated by GEN and is closely associated with ER stress and autophagy markers [10,11,12]. The endoplasmic reticulum (ER) is central to protein folding and calcium homeostasis [13]. ER stress triggers the unfolded protein response (UPR), mediated by *IRE1α*, *PERK*, and *ATF6* [14,15]. Under persistent stress, *GRP78/BiP* dissociates, activating CHOP-dependent apoptosis via *Bax/Bak* and caspase cascades [6,16]. ER stress is intrinsically linked to inflammatory mediators such as NF-κB, iNOS, IL-6, and TNFα-R1 [5]. Ellagic acid (EA), a natural polyphenol with potent antioxidant properties found in various medicinal plants, fruits, and vegetables [17], contributes to diverse biological activities including antidiabetic, anti-inflammatory, anticancer, and antimutagenic effects, as well as protection against hepatic, neural, cardiac, and renal damage [18,19]. EA’s nephroprotective mechanisms involve both direct and indirect antioxidant activities [20]; previous studies have shown that EA modulates oxidative stress and enhances endogenous antioxidant defense systems [21]. Although ellagic acid (EA) is known to reduce gentamicin (GEN)-induced oxidative stress and apoptosis, its broader renoprotective actions across multiple pathways and the crosstalk among ER stress, autophagy, inflammation, and apoptosis remain poorly defined [22]. Prior studies did not comprehensively integrate ER stress signaling pathways (*GRP78*, *PERK*, *IRE1α*, *ATF6*, *CHOP*), autophagy (LC3A), apoptosis (*BAX*, *Caspase-3*), and inflammatory mediators, nor did they evaluate the effects of EA on autophagy biomarkers [22]. Based on the existing evidence, we hypothesized that ellagic acid co-treatment mitigates gentamicin-induced nephrotoxicity by modulating ER stress-mediated crosstalk between apoptosis, autophagy, and inflammation, rather than acting solely as a direct antioxidant [6,22]. Therefore, the objectives of this study were (1) to evaluate whether EA co-treatment alleviates ER stress across all three UPR branches; (2) to assess its effects on the balance between apoptosis and autophagy; (3) to characterize the inflammatory response, particularly the selective IL-6/*COX-2* modulation; and (4) to determine the involvement of the *Nrf2/HO-1* pathway. Importantly, this study aimed to compare the effects of ellagic acid alone and in combination with gentamicin to determine whether its protective effects are specifically evident under nephrotoxic conditions. To our knowledge, this study provides an integrated mechanistic analysis of EA against GEN nephrotoxicity, correlating molecular data with quantitative immunofluorescence and histopathology. The novelty of this work lies in identifying a selective suppression of IL-6, a response not observed for TNF-α or IL-1β, and in demonstrating that EA co-treatment attenuates GEN-induced *Nrf2/HO-1* upregulation rather than directly activating it.

## 2. Material and Methods

### 2.1. Materials (Chemicals and Reagents)

GEN sulfate was obtained from Exir Pharmaceutical Company (Iran). 2′,7′-dichlorofluorescein diacetate (DCFH-DA), ellagic acid (EA), methanol, thiobarbituric acid (TBA), 4′,6-diamidino-2-phenylindole, Triton X-100, and TBS solution were purchased from Sigma-Aldrich (St. Louis, MO, USA). Specific primers targeting genes of interest were synthesized by Sangon Biotech (Shanghai, China). Anti-LC3A (Rabbit monoclonal; Cell Signaling Technology (CST), Danvers, MA, USA; Cat# 4599; dilution 1:200) Anti-KIM-1 (Rabbit polyclonal; Abcam, Cambridge, UK; Cat# ab47635; dilution 1:100); goat anti-rabbit IgG HRP-conjugated (Abcam, Cambridge, UK; Cat# ab6721; dilution 1:500).

### 2.2. Methods

#### 2.2.1. Study Design and Animal Treatment

Male Sprague-Dawley rats (n = 40, weighing 250 ± 20 g) were obtained from Shiraz University of Medical Sciences, Shiraz, Iran. The animals were maintained under standard laboratory conditions (12 h light/dark cycle, relative humidity of 45 ± 5%, and temperature of 23 ± 1 °C) with free access to tap water and standard rat diet (RoyanFeed^®^, Isfahan, Iran). This study adhered to the ethical guidelines for laboratory animal care and use established by Shiraz University of Medical Sciences (Ethics code: IR.SUMS.AEC.1403.003). Clinical trial number: not applicable.

The animals were randomly divided into four experimental groups (n = 10 per group): Control group: received 0.5% carboxymethyl cellulose (CMC) (2 mL/kg, oral gavage); GEN group: received GEN (100 mg/kg, intraperitoneal [i.p.] injection); GEN + EA group: received GEN (100 mg/kg, i.p.) + ellagic acid (100 mg/kg, oral gavage); EA group: received ellagic acid (100 mg/kg, oral gavage) [22,23]. Ellagic acid was prepared as a suspension in 0.5% carboxymethyl cellulose (CMC) and administered by oral gavage to ensure consistent dosing across treatment groups. Control animals received the same volume of 0.5% CMC under identical experimental conditions to exclude potential vehicle-related effects. Gentamicin was administered intraperitoneally in equal volumes across all experimental groups. This design ensured that the observed effects could be attributed to pharmacological interventions rather than differences in animal handling, vehicle exposure, or administration routes. All experimental groups received equal volumes of vehicle to ensure consistency across treatments.

The selected doses and treatment duration for gentamicin and ellagic acid were based on previously validated experimental models of nephrotoxicity and renoprotection and were chosen to ensure pharmacological relevance rather than clinical dose equivalence. The study protocol, including doses and treatment duration, was designed based on previous studies [22,24,25].

All treatments were administered daily for 14 consecutive days between 9:00 and 14:00. The doses of GEN and EA used in this study were based on previous studies and preliminary experiments [5,26]. After the 14-day treatment period, the animals were placed in a metabolic cage for 24 h of urine collection. Then, the animals were deeply anesthetized with an intraperitoneal injection of thiopental (70 mg/kg). Blood samples were collected from the abdominal vein using sterile syringes (5 mL) and collection tubes, after which the animals were euthanized by exsanguination under deep anesthesia, performed by trained personnel. All euthanasia procedures were performed in accordance with the American Veterinary Medical Association (AVMA) Guidelines for the Euthanasia of Animals (2020) [27]. Serum was separated by centrifugation (4000 *g*, 15 min, 4 °C). The kidney tissues were excised, washed with cold normal saline, and stored at −70 °C for biochemical analysis and RNA extraction. The Parsazmoon^®^ kit (Tehran, Iran) was used to assess the serum and urine biomarkers.

#### 2.2.2. Measurement of Inflammatory Cytokines in Serum via ELISA

The quantitative determination of inflammatory cytokines (TNF-α, IL-6, and IL-1β) in serum samples was performed using commercially available enzyme-linked immunosorbent assay (ELISA) kits (Monobind, Lake Forest, CA, USA). All assays were conducted according to the manufacturer’s instructions.

#### 2.2.3. MDA Measurement

The levels of lipid peroxidation were evaluated using thiobarbituric acid reactive substances (TBARS). Briefly, the reaction mixture consisted of 0.375% *w*/*v* thiobarbituric acid, 1% *w*/*v* phosphoric acid (pH 2), and 500 μL of tissue homogenate prepared in KCl (10% *w*/*v*). The mixture was subsequently heated in boiling water for 45 min; after cooling, 2 mL of n-butanol was added to the samples and vortexed. Finally, the samples were centrifuged for 5 min at 3000 *g*, and the absorbance of the resulting color in the supernatant was measured at 532 nm using a microplate reader (Synergy™ HTX Multi-Mode Microplate Reader, BioTek, Winooski, VT, USA) [28]. Results were expressed as nmol of MDA per mg protein (nmol/mg protein).

#### 2.2.4. Measurement of Reactive Oxygen Species (ROS)

Reactive oxygen species (ROS) levels in kidney tissue were estimated using a previously described method. Kidney tissues were homogenized in ice-cold 40 mM Tris-HCl buffer (pH 7.4) at a ratio of 1:10 (*w*/*v*). An aliquot of 100 μL of the resulting tissue homogenate was mixed with 1 mL of Tris-HCl buffer and 5 μL of 2′,7′-dichlorofluorescein diacetate (10 μM). The mixture was incubated for 30 min at 37 °C. Fluorescence intensity was measured using a microplate reader (Synergy™ HTX Multi-Mode Microplate Reader, BioTek, USA) at an excitation wavelength of 485 nm and an emission wavelength of 525 nm [22]. Results were expressed as a percentage of fluorescence intensity relative to control.

#### 2.2.5. Evaluation of Inflammatory, ER Stress and Apoptotic Markers by Quantitative Real Time-PCR

Total RNA was extracted from kidney tissues using an RNA extraction kit (Pars Tous, Mashhad, Iran). Subsequently, cDNA was synthesized using the Easy cDNA Synthesis kit (Pars Tous, Mashhad, Iran). The concentration and purity of RNA were measured using NanoDrop ND-1000 spectrophotometer (Nano Drop, Wilmington, DE, USA). The obtained cDNA was quantified after reverse transcription and used for subsequent RT-qPCR experiments. The mRNA expression levels of genes associated with ER stress, such as Inositol-requiring enzyme 1 (*IRE1*), Activating transcription factor 6 (*ATF6*), Glucose-regulated protein 78 (*GRP78/BiP*), Protein kinase RNA-like ER kinase (*PERK*), and C/EBP homologous protein (*CHOP*), were evaluated. Additionally, the mRNA expression levels of apoptosis-related genes, including *Bcl-2*-associated X protein (*BAX*), B-cell lymphoma 2 (*Bcl-2*), and *caspase-3*, along with other relevant genes such as Nuclear factor erythroid 2-related factor 2 (*Nrf2*), Heme oxygenase-1 (*HO-1*), and Cyclooxygenase-2 (*COX-2*), were assessed in kidney tissue using real-time PCR. The primer sequences are presented in Table 1. The RT-qPCR experimental configuration consisted of the following components: 1 μL of cDNA template at a concentration of 30 ng, 2 μL of primers (10 μM), 10 μL of Ampliqon SYBR green Master Mix High ROX, and deionized water added to reach a total volume of 20 μL. The amplification process for RT-qPCR was performed as follows: an initial incubation for 30 s at 95 °C, followed by 40 amplification cycles. Each amplification cycle consisted of a denaturation step for 5 s at 95 °C, followed by an annealing step for 30 s at 60–63 °C and extension at 72 °C for 30 s. Real-time PCR was performed with a thermocycler and SYBR green detection system (Applied Biosystems, Foster City, CA, USA). Relative mRNA expression levels were calculated using the 2^(−ΔΔCt)^ method, with superscript and subscript formatting, and normalized to *β-actin* [29].

#### 2.2.6. Immunofluorescence Analysis

To determine the expression levels of KIM-1 and LC3A in kidney tissue, immunofluorescence analysis was employed. In summary, immediately after excision, the left kidney tissues were fixed in a 10% formalin solution. Subsequently, after tissue processing, paraffin was utilized to harden and prepare the tissue for embedding and sectioning. The samples were sectioned with a microtome to a thickness of 5 μm and placed on silanized glass slides for further analysis. The tissue sections were then incubated with a primary antibody. After an overnight incubation, a fluorophore-conjugated secondary antibody was introduced. Nuclear counterstaining was conducted using DAPI (D9542, Sigma-Aldrich) for 20 min. Fluorescent images were captured using an Olympus fluorescence microscope at 40× magnification to assess marker expression and localization within the kidney tissue. For quantitative analysis, mean fluorescence intensity was measured in five randomly selected fields per section from five rats per group using ImageJ software (ver sion 1.53t; NIH, USA; https://imagej.net/ij/; accessed on 1 June 2026) and normalized to the control group (set as 100%). This quantitative approach allows for objective comparison of protein expression levels across treatment groups.

#### 2.2.7. Tissue Histopathology and Scoring

Animals were euthanized, kidneys excised, fixed in 10% formalin, dehydrated with different degrees of alcohol (60, 70, 80, 96, and 100), and then embedded in paraffin blocks. Afterward, samples were sectioned with a thickness of 5 µm and stained with hematoxylin and eosin (H&E) (Leica, Wetzlar, Germany) and Masson’s trichrome (Merck, Darmstadt, Germany). Subsequently, tissue sections were evaluated by a blinded pathologist. For semi-quantitative assessment of renal injury, a scoring system was applied as follows: 0 = no damage; 1 = mild damage (affecting <25% of tubules); 2 = moderate damage (25–50% of tubules); 3 = severe damage (50–75% of tubules); 4 = very severe damage (>75% of tubules). Parameters evaluated included tubular dilation, cast formation, brush border loss, vacuolization, and glomerular shrinkage. At least 10 non-overlapping fields per section (400× magnification) were examined. This scoring system provides a quantitative measure of histopathological changes, complementing the molecular data.

#### 2.2.8. Statistical Analysis

Statistical analysis was performed using GraphPad Prism 6 software (version 6.0; GraphPad Software Inc., San Diego, CA, USA; https://www.graphpad.com/; accessed on 4 June 2026). Normality was assessed using the Shapiro–Wilk test. For data with normal distribution, one-way ANOVA followed by Tukey’s post hoc test was used. For non-normal data, Kruskal–Wallis followed by Dunn’s post-test was applied. *p* < 0.05 was considered to be statistically significant. Data are presented as mean ± SEM.

All figures, including the graphical abstract, were created using Adobe Illustrator (version not specified; Adobe Inc., San Jose, CA, USA; https://www.adobe.com/products/illustrator.html; accessed on 1 June 2026) and Microsoft PowerPoint (version not specified; Microsoft Corporation, Redmond, WA, USA; https://www.microsoft.com/en-us/microsoft-365/powerpoint; accessed on 1 June 2026). BioRender was not used. Raw, uncropped, unedited microscopy images for immunofluorescence are provided as separate Supplementary Files.

## 3. Results

### 3.1. Effects of GEN and Ellagic Acid on Serum Inflammatory Cytokines

**Figure 1:**
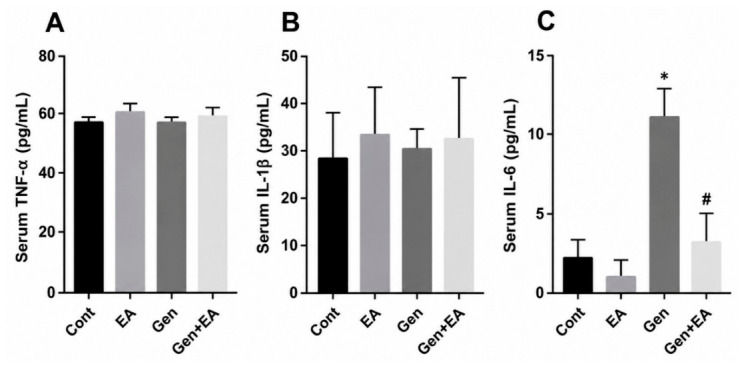
Effects of GEN and ellagic acid treatments on serum levels of inflammatory cytokines TNF-α (**A**), IL-1β (**B**), and IL-6 (**C**) in different experimental groups. Data are presented as mean ± SEM (n = 10). * *p* < 0.05 compared to the control group; # *p* < 0.05 compared to the GEN group. Cont: Control group, EA: Ellagic acid group (100 mg/kg; oral gavage); GEN: Gentamicin group (100 mg/kg; i.p.), GEN + EA: Gentamicin plus ellagic acid group.

To assess the selective anti-inflammatory effect of EA, serum levels of TNF-α, IL-1β, and IL-6 were measured. Serum TNF-α concentrations did not significantly differ between the control and gentamicin (GEN, 100 mg/kg, i.p.) groups, with values (mean ± SEM) of 57.3 ± 2.1 pg/mL in control and 58.1 ± 2.4 pg/mL, respectively (*p* > 0.05; Figure 1A). Similarly, no significant differences were observed in serum IL-1β levels across the experimental groups (*p* > 0.05; Figure 1B). In contrast, GEN treatment significantly elevated serum IL-6 levels compared with the control group (* *p* < 0.05; Figure 1C). Co-administration of EA (100 mg/kg, oral gavage) with GEN (100 mg/kg, i.p.) for 14 days significantly attenuated this elevation (#*p* < 0.05 vs. GEN). The lack of change in TNF-α and IL-1β may reflect the specific time point of measurement (14 days) or differences in assay sensitivity; further time-course studies would be needed to determine whether these cytokines are involved at earlier stages.

### 3.2. Effects of GEN and Ellagic Acid on Kidney Tissue Inflammatory Gene Expression

**Figure 2:**
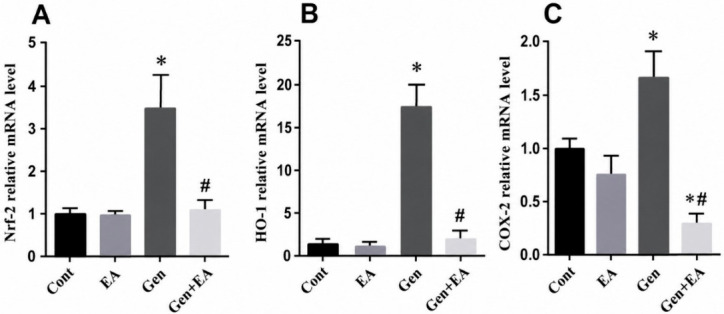
Effects of GEN and ellagic acid treatments on mRNA expression levels of inflammation-related genes in kidney tissue: *Nrf2* (**A**), *HO-1* (**B**), and *COX-2* (**C**). Data are presented as mean ± SEM (n = 6). * *p* < 0.05 compared to the control group; # *p* < 0.05 compared to the GEN group. Cont: Control group, EA: Ellagic acid group (100 mg/kg; oral gavage); GEN: Gentamicin group (100 mg/kg; i.p.), GEN + EA: Gentamicin plus ellagic acid group.

To assess the effect of EA on GEN-induced inflammatory and oxidative stress-related gene expression, mRNA levels of *Nrf2*, *HO-1*, and *COX-2* were measured in kidney tissue. GEN (100 mg/kg, i.p.) significantly upregulated *Nrf2* mRNA expression compared with the control group (* *p* < 0.05; Figure 2A). Similarly, HO-1 mRNA expression was significantly increased in the GEN group (* *p* < 0.05; Figure 2B). GEN also significantly elevated *COX-2* mRNA levels compared with the control group (* *p* < 0.05; Figure 2C). Co-administration of EA (100 mg/kg, oral gavage) with GEN (100 mg/kg, i.p.) normalized the GEN-induced upregulation of *Nrf2* and *HO-1*, returning expression levels toward baseline (#*p* < 0.05 vs. GEN). Co-administration of EA also significantly reduced *COX-2* expression compared with the GEN group (#*p* < 0.05 vs. GEN).

### 3.3. Effects of GEN and Ellagic Acid on Biochemical Markers of Renal Injury

**Figure 3:**
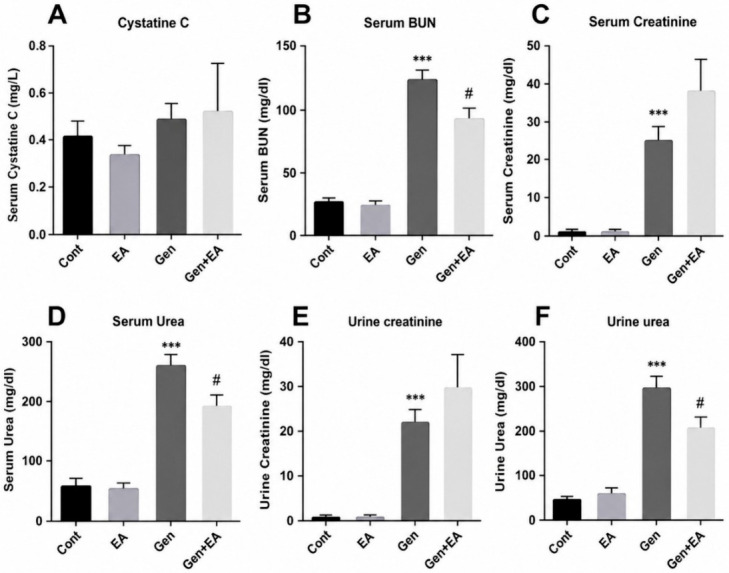
Effects of GEN and ellagic acid on serum cystatin C (**A**), BUN (**B**), creatinine (**C**), and urea (**D**), as well as urinary creatinine (**E**) and urea (**F**) levels. Data are presented as mean ± SEM (n = 10). *** *p* < 0.05 compared to the control group; # *p* < 0.05 compared to the GEN group. Cont: Control group, EA: Ellagic acid group (100 mg/kg; oral gavage); GEN: Gentamicin group (100 mg/kg; i.p.), GEN + EA: Gentamicin plus ellagic acid group.

To assess the protective effect of EA on renal function, serum and urinary markers of kidney injury were measured. GEN significantly increased serum urea, BUN, and creatinine levels compared with the control group (*** *p* < 0.05; Figure 3B–D). No significant changes were observed in serum cystatin C levels (Figure 3A). Urinary urea and creatinine levels were also significantly elevated in the GEN group (*** *p* < 0.05; Figure 3E,F). Co-administration of EA (100 mg/kg, oral gavage) with GEN significantly reduced serum urea, BUN, and urinary urea levels compared with the GEN group (# *p* < 0.05; Figure 3B,D,F). Serum creatinine levels were not significantly improved by co-administration of EA. This may be due to the relatively mild degree of renal impairment in this model or because creatinine is a less sensitive marker than BUN and urea for detecting early tubular injury.

### 3.4. Effects of GEN and Ellagic Acid on Oxidative Stress Factors

**Figure 4:**
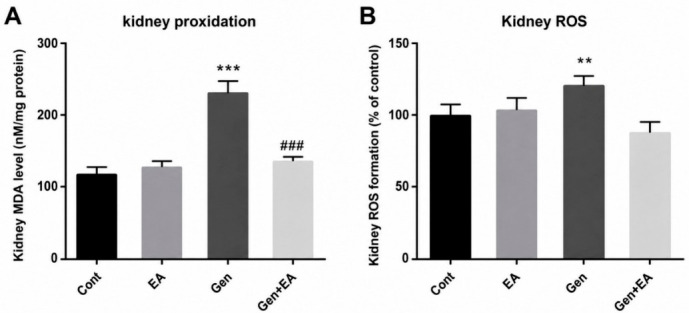
Effects of GEN and ellagic acid on oxidative stress markers MDA levels (**A**) and ROS formation (**B**) in kidney tissue. MDA data are expressed as nmol/mg protein; ROS data are expressed as percentage of control (mean ± SEM, n = 10). ** *p* < 0.01 compared to the control group; *** *p* < 0.001 compared to the Control group; ### *p* < 0.001 compared to the GEN group. Cont: Control group, EA: Ellagic acid group (100 mg/kg; oral gavage); GEN: Gentamicin group (100 mg/kg; i.p.), GEN + EA: Gentamicin plus ellagic acid group.

To determine the effect of EA on GEN-induced oxidative stress, renal MDA and ROS levels were measured. GEN significantly increased MDA levels and produced a modest but statistically significant elevation in ROS levels compared with the control group (*** *p* < 0.05 for MDA and ** *p* < 0.05 for ROS, respectively; Figure 4A,B). Co-administration of EA (100 mg/kg, oral gavage) with GEN significantly and reduced MDA levels (### *p* < 0.05 vs. GEN). However, co-administration of EA did not significantly reduce ROS levels (*p* > 0.05).

### 3.5. Effects of GEN and Ellagic Acid on Endoplasmic Reticulum Stress Markers Gene Expression

**Figure 5:**
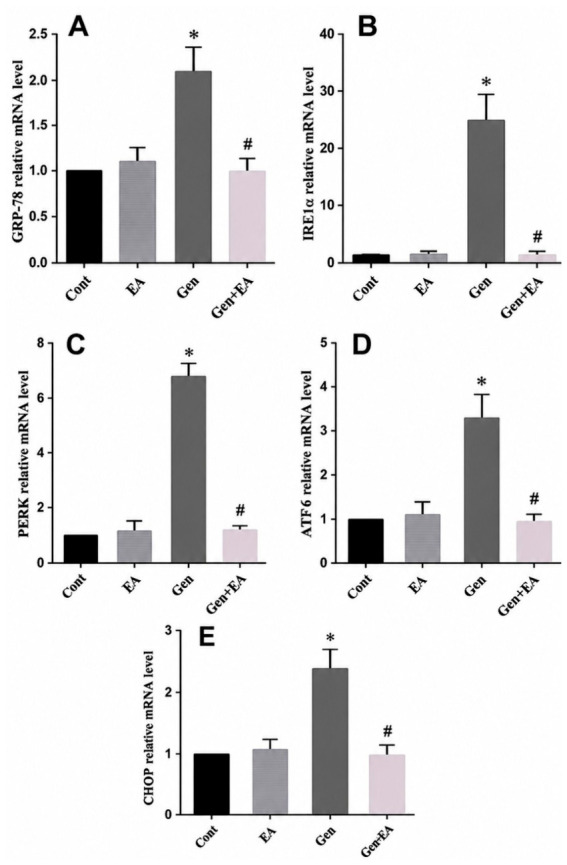
Effects of GEN and ellagic acid treatments on mRNA expression levels of endoplasmic reticulum stress-related genes in kidney tissue: *GRP78* (**A**), *IRE1α* (**B**), *PERK* (**C**), *ATF6* (**D**), and *CHOP* (**E**). Data are presented as mean ± SEM (n = 6). * *p* < 0.05 compared to the control group; # *p* < 0.05 compared to the GEN group. Cont: Control group, EA: Ellagic acid group (100 mg/kg; oral gavage); GEN: Gentamicin group (100 mg/kg; i.p.), GEN + EA: Gentamicin plus ellagic acid group.

To determine the effect of EA on GEN-induced ER stress, mRNA expression of *GRP78*, *IRE1α*, *PERK*, *ATF6*, and *CHOP* was measured. GEN (100 mg/kg, i.p.) significantly upregulated the mRNA expression of all five markers compared with the control group (* *p* < 0.05 for each; Figure 5A–E). Co-administration of EA (100 mg/kg, oral gavage) with GEN significantly attenuated the GEN-induced upregulation of *GRP78*, *IRE1α*, *PERK*, *ATF6*, and *CHOP* (* *p* < 0.05 vs. GEN). Treatment with ellagic acid alone had minimal effects on baseline expression of these ER stress markers compared with the control group (* *p* > 0.05 for all markers).

### 3.6. Effects of GEN and Ellagic Acid on Apoptosis-Related Gene Expression

**Figure 6:**
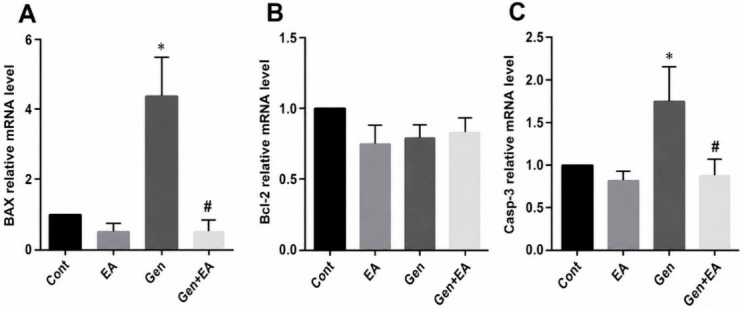
Effects of GEN and ellagic acid treatments on mRNA expression levels of apoptosis-related genes in kidney tissue: *Bax* (**A**), Bcl-2 (**B**), and *caspase-3* (**C**). Data are presented as mean ± SEM (n = 6). * *p* < 0.05 compared to the control group; # *p* < 0.05 compared to the GEN group. Cont: Control group, EA: Ellagic acid group (100 mg/kg; oral gavage); GEN: Gentamicin group (100 mg/kg; i.p.), GEN + EA: Gentamicin plus ellagic acid group.

To investigate the effect of EA on GEN-induced apoptosis, mRNA expression of *BAX*, *Bcl-2*, and *caspase-3* was measured. GEN (100 mg/kg, i.p.) significantly upregulated *BAX* mRNA expression compared with the control group (* *p* < 0.05; Figure 6A). *Bcl-2* mRNA expression was not significantly altered by GEN treatment (*p* > 0.05; Figure 6B). GEN also significantly increased *caspase-3* mRNA expression (* *p* < 0.05; Figure 6C). Co-administration of EA (100 mg/kg, oral gavage) with GEN significantly attenuated the GEN-induced upregulation of *BAX* and *Caspase-3* (* *p* < 0.05 vs. GEN).

### 3.7. Effects of GEN and Ellagic Acid on Autophagy and Kidney Injury Markers

**Figure 7:**
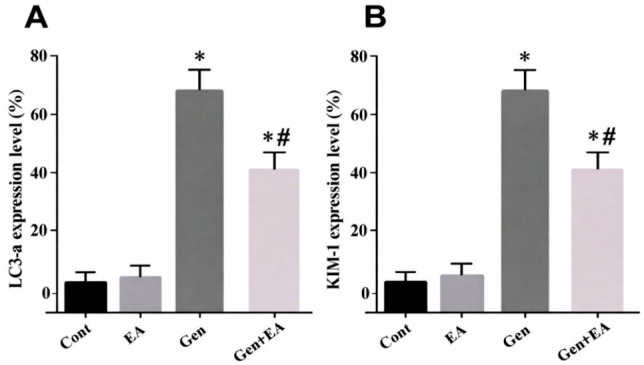
Effects of GEN and ellagic acid treatments on protein expression levels of the autophagy marker LC3A (**A**) and the kidney injury marker KIM-1 (**B**) in kidney tissue, as assessed by immunofluorescence staining. Quantitative analysis was performed using ImageJ software on five randomly selected non-overlapping fields per group, and the immunopositive area for each marker was measured after background subtraction and expressed as a percentage of the total microscopic field area. Data are presented as mean ± SEM (n = 5 fields per group from five rats). * *p* < 0.05 compared to the control group; # *p* < 0.05 compared to the GEN group. Cont: Control group; EA: Ellagic acid group (100 mg/kg; oral gavage); GEN: Gentamicin group (100 mg/kg; i.p.); GEN + EA: Gentamicin plus ellagic acid group.

To investigate the effect of EA on autophagy and tubular injury, protein expression of LC3A and KIM-1 was assessed by immunofluorescence. Following background subtraction, the immunopositive area for each marker was measured, normalized to the control group (set as 100%), and expressed as a percentage of the control (mean ± SEM). Quantitative analysis (five randomly selected fields from each of five rats per group) showed that GEN (100 mg/kg, i.p.) significantly increased LC3A and KIM-1 immunoreactivity compared with the control group (* *p* < 0.05; Figure 7A,B). Co-administration of EA (100 mg/kg, oral gavage) with GEN for 14 days significantly reduced GEN-induced LC3A and KIM-1 expression (* *p* < 0.05 vs. GEN). LC3A immunofluorescence reflects changes in autophagy-related protein expression; however, autophagic flux was not directly assessed in the present study.

### 3.8. Immunofluorescence Analysis of LC3A Expression in Kidney Tissue

**Figure 8:**
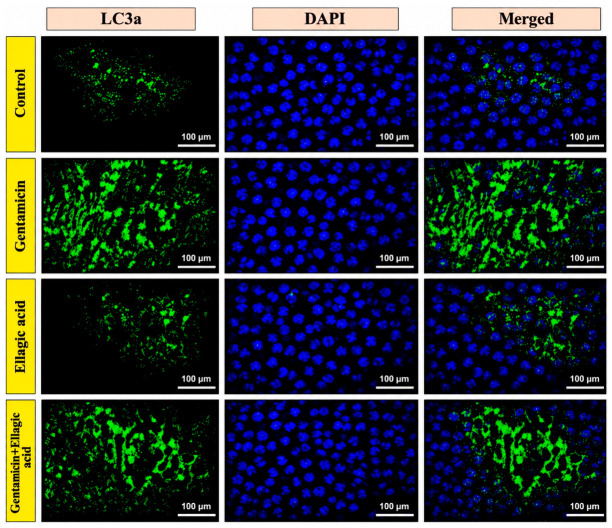
Immunofluorescence analysis of LC3A expression in kidney tissue. Representative images (40× magnification, scale bar = 100 μm). Green: LC3A; blue: DAPI (nuclei). Control group: baseline LC3A expression. GEN group (100 mg/kg, i.p.): pronounced increases LC3A fluorescence intensity with perinuclear localization. EA group (100 mg/kg, oral gavage): slight increase in LC3A compared with control. GEN + EA group (GEN 100 mg/kg, i.p. + EA 100 mg/kg, oral gavage, 14 days): reduced LC3A immunoreactivity compared with GEN group. As noted in Section 3.7, LC3A immunofluorescence alone does not measure autophagic flux.

To visualize and compare LC3A expression patterns across treatment groups, immunofluorescence staining was performed. Representative images are shown in Figure 8. Control samples exhibited baseline LC3A expression. GEN-treated kidneys (100 mg/kg, i.p.) displayed a pronounced increase in LC3A fluorescence intensity, with staining observed predominantly in perinuclear cytoplasmic regions, consistent with its reported subcellular distribution [7]. Co-administration of EA (100 mg/kg, oral gavage) with GEN for 14 days resulted in reduced LC3A immunoreactivity compared with the GEN-only group. As noted in Section 3.7, LC3A immunofluorescence alone does not measure autophagic flux; additional experiments such as LC3-II turnover in the presence of lysosomal inhibitors, would be required to confirm changes in flux [30].

### 3.9. Immunofluorescence Analysis of KIM-1 Expression in Kidney Tissue

**Figure 9:**
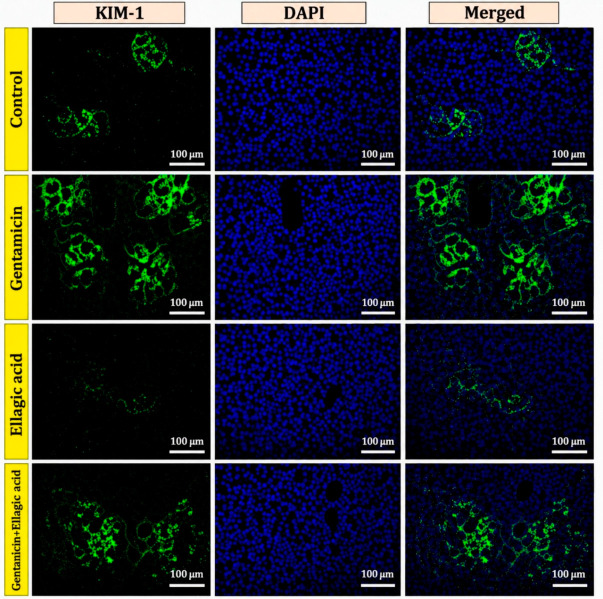
Immunofluorescence analysis of KIM-1 expression in kidney tissue. Representative images (40× magnification, scale bar = 100 μm). KIM-1 immunoreactivity (green); DAPI (blue). Control group: minimal KIM-1 fluorescence. GEN group (100 mg/kg, i.p.): intense KIM-1 staining, predominantly in proximal tubular epithelia. EA group (100 mg/kg, oral gavage): slight, scattered KIM-1 expression. GEN + EA group (GEN 100 mg/kg, i.p. +EA 100 mg/kg, oral gavage, 14 days): reduced KIM-1 immunoreactivity compared with GEN group.

To evaluate the effect of EA on tubular injury, KIM-1 expression was assessed by immunofluorescence. Quantitative analysis (five randomly selected fields from each of five rats per group) showed that control kidneys exhibited minimal KIM-1 immunoreactivity (Figure 9). In contrast, GEN-treated kidneys (100 mg/kg, i.p.) showed intense KIM-1 staining localized to proximal tubular epithelia. Co-administration of EA (100 mg/kg, oral gavage) with GEN for 14 days resulted in reduced KIM-1 immunoreactivity compared with the GEN group.

### 3.10. Histopathological Analysis of Kidney Tissue and Scoring

**Figure 10:**
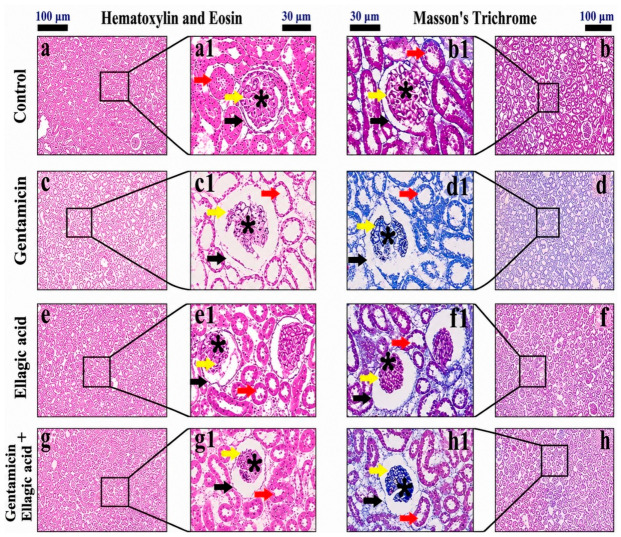
Histopathological analysis of renal cortex tissue (H&E and Masson’s trichrome staining). Representative photographs of rat kidney cortex sections from each experimental group. Low magnification (scale bar = 100 μm): (**a**) Control group: normal glomeruli and intact cortical tubules; (**b**) Control group (second image): normal renal architecture; (**c**) GEN group (100 mg/kg, i.p.): loss of spherical glomerular structure, degenerated capillaries, and necrotic Bowman’s capsule epithelium; (**d**) GEN group (second image): marked tubular degeneration and reduced epithelial thickness; (**e**) EA group (100 mg/kg, oral gavage): near-normal renal histology; (**f**) EA group (second image): preserved glomerular and tubular structures; (**g**) GEN + EA group (GEN 100 mg/kg i.p. + EA 100 mg/kg oral gavage, 14 days): relatively preserved glomeruli with no endothelial cell erosion; (**h**) GEN + EA group (second image): preserved Bowman’s capsule continuity. High magnification (scale bar = 30 μm): (**a1**,**b1**) Control group: normal glomerulus, intact Bowman’s capsule, and healthy tubular epithelium; (**c1**,**d1**) GEN group: glomerular atrophy (star), degenerated capillaries and eroded endothelial cells (yellow arrow), necrotic and discontinuous Bowman’s capsule epithelium (black arrow), and reduced tubular epithelial thickness (red arrow); (**e1**,**f1**) EA group: preserved glomerular and tubular structures with normal morphology; (**g1**,**h1**) GEN + EA group: relatively preserved glomeruli (star), no endothelial cell erosion (yellow arrow), preserved Bowman’s capsule continuity but dilated space (black arrow).

**Figure 11:**
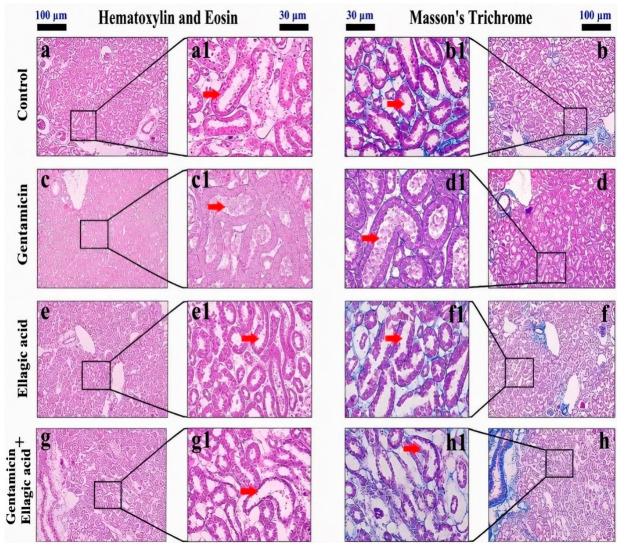
Histopathological analysis of renal medulla tissue (H&E and Masson’s trichrome staining). Representative photographs of rat kidney medulla sections from each experimental group. Low magnification (scale bar = 100 μm): (**a**) Control group (H&E): normal renal architecture with intact tubules; (**b**) Control group (Masson’s trichrome): normal structure with minimal collagen deposition; (**c**) GEN group (100 mg/kg, i.p.) (H&E): marked histopathological alterations in renal tubules; (**d**) GEN group (Masson’s trichrome): disrupted tubular architecture associated with renal injury; (**e**) EA group (100 mg/kg, oral gavage) (H&E): near-normal morphology with preserved tubular organization; (**f**) EA group (Masson’s trichrome): preserved structure with minimal connective tissue deposition; (**g**) GEN + EA group (GEN 100 mg/kg i.p. + EA 100 mg/kg oral gavage, 14 days) (H&E): improved tubular architecture compared with the GEN group; (**h**) GEN + EA group (Masson’s trichrome): partial restoration of normal tissue organization with reduced pathological alterations. High magnification (scale bar = 30 μm): (**a1**,**b1**) Control group: intact tubules without cast formation or epithelial degeneration; (**c1**,**d1**) GEN group: tubular epithelial degeneration and prominent tubular cast formation (red arrow); (**e1**,**f1**) EA group: preserved tubular epithelial integrity without evidence of cast formation; (**g1**,**h1**) GEN + EA group: reduced tubular epithelial degeneration and decreased cast formation compared with the GEN group (red arrow). Red arrows in high-magnification panels indicate tubular epithelial degeneration and/or tubular cast formation.

To assess the protective effect of EA on renal tissue structure, histopathological examination was performed using H&E and Masson’s trichrome staining. Microscopic changes in the renal cortex and medulla are shown in Figure 10 and Figure 11, respectively. Control and EA-treated rats showed normal glomerular and tubular architecture (Figure 10a,b,e,f and Figure 11(a1,b1,e1,f1)). GEN-treated rats (100 mg/kg, i.p.) displayed glomerular degeneration, capillary damage, tubular dilation, brush border loss, and medullary cast formation (Figure 10(c1,d1) and Figure 11(c1,d1)). No inflammatory cell infiltration or capillary congestion was observed. In the group receiving GEN + EA (100 mg/kg EA, oral gavage) for 14 days, glomeruli appeared qualitatively smaller than those in the control and EA groups, with no endothelial cell erosion, although Bowman’s space remained dilated. Medullary cast excretion was reduced compared with the GEN group (Figure 10(g1,h1) and Figure 11(g1,h1)). Although quantitative glomerular morphometry was not performed, qualitative observations suggested partial preservation of glomerular architecture. The tubular injury score was defined as follows: 0 = no damage; 1 = mild damage (affecting <25% of tubules); 2 = moderate damage (25–50%); 3 = severe damage (50–75%); 4 = very severe damage (>75%). Semi-quantitative histological scoring (Table 2) confirmed that GEN treatment significantly increased tubular injury scores compared with control (3.2 ± 0.3 vs. 0.3 ± 0.2, *** *p* < 0.001). Co-treatment with EA significantly reduced these scores (1.5 ± 0.2, ** *p* < 0.01 vs. GEN). Histological scoring was performed on 5 randomly selected rats per group (n = 5) because of the labor-intensive nature of quantitative histomorphometry; however, this sample size is consistent with previous studies and was sufficient to detect statistically significant differences.

## 4. Discussion

A key finding of the present study is that ellagic acid alone did not significantly alter renal parameters, whereas it markedly attenuated gentamicin-induced alterations when co-administered. GEN is widely used for serious Gram-negative infections but causes nephrotoxicity in 10–20% of cases [31,32], primarily due to its accumulation in the renal cortex [1]. The present findings suggest a coordinated interplay between ER stress, oxidative stress, and inflammatory pathways in gentamicin-induced renal injury, with the protective effects of ellagic acid being primarily evident under pathological conditions rather than in normal physiological states. This integrated mechanistic analysis simultaneously evaluated ER stress, apoptosis, autophagy, selective inflammation, and *Nrf2/HO-1* activation within a single experimental model. The results indicate that EA co-treatment attenuates ER stress across all three UPR branches and is associated with modulation of the apoptosis-autophagy balance. It selectively suppresses IL-6 and *COX-2* without affecting TNF-α or IL-1β, and attenuates the GEN-induced compensatory upregulation of *Nrf2/HO-1* and reduces lipid peroxidation and tissue injury. These findings suggest that EA’s renoprotective effects involve integrated modulation of multiple stress-response pathways rather than a simple antioxidant mechanism. Consistent with earlier reports, GEN elevated serum BUN, urea, and creatinine [33]. EA and EA-rich extracts have been shown to modulate these markers and oxidative stress in variable ways [34,35]. Specifically, studies reported variable effects on urea and antioxidant enzymes [36]. Other studies demonstrated nephroprotection through oxidative stress contribution [37]. However, those studies did not examine ER stress or autophagy [22].

Our investigation revealed a selective inflammatory response to GEN, characterized by a significant increase in serum IL-6 levels, without notable alterations in TNF-α or IL-1β. This suggests that IL-6 may act as a primary mediator in GEN-induced nephrotoxicity and serve as a sensitive biomarker of renal injury. At the tissue level, GEN significantly upregulated *COX-2* expression, suggesting localized renal inflammation [38]. GEN also activated intrinsic apoptotic pathways, as evidenced by significant upregulation of *BAX* and caspase-3, without a corresponding increase in *Bcl-2* expression, demonstrating a pro-apoptotic shift in the *BAX*/*Bcl-2* ratio [39]. These findings align with previous reports demonstrating altered expression of pro-apoptotic indicators in renal cortical tissue following GEN exposure [6]. In addition, EA co-treatment was associated with selective anti-inflammatory effects on GEN-induced IL-6 elevation and significantly attenuated *COX-2* upregulation at the tissue level. These findings are consistent with reports that EA modulates inflammatory mediators [40]. EA’s therapeutic applications extend beyond nephroprotection to antidiabetic, anti-inflammatory, anticancer, and antimutagenic effects [18,41]. In inflammation contexts, EA modulates inflammatory mediators, attenuating GEN-induced increases in inflammatory biomarkers. Selective involvement of inflammatory mediators suggests targeted mechanisms against GEN-induced inflammatory cascades [42]. EA was associated with significant anti-apoptotic effects, effectively attenuating GEN-induced *BAX* and *caspase-3* increase. These findings align with previous studies showing EA ameliorates *caspase-3* activation and alters the *Bax/Bcl-2* ratio [37]. EA may exert nephroprotective effects, at least in part, through involvement in of apoptosis-related pathways.

GEN exposure was associated with increased LC3A expression and altered immunofluorescence distribution across renal tissue, suggesting changes in autophagy-related protein expression in response to GEN-induced cellular stress [43]. However, as noted in Section 3.7, LC3A immunofluorescence alone does not measure autophagic flux; therefore, these findings reflect changes in autophagy-related protein expression rather than definitive flux. The distinct regulation of autophagy markers supports earlier observations that LC3A and LC3B exhibit differential roles in cellular stress responses [8]; however, their specific roles in nephrotoxic responses, particularly in gentamicin-induced injury, require further investigation. Moreover, the significant elevation of KIM-1, confirmed by immunofluorescence, is consistent with proximal tubular injury, consistent with established patterns of GEN-induced nephrotoxicity.

The parallel elevation of KIM-1 alongside ER stress markers (*GRP78*, *CHOP*) and the autophagy-related marker LC3A was observed, suggesting a potential interplay among these pathways during GEN-induced renal injury. Notably, pharmacological inhibition of ER stress or autophagy attenuated KIM-1 expression and renal injury, further supports KIM-1 as a potential regulator of these pathways in GEN-induced nephrotoxicity [11]. ER stress occurs when misfolded proteins accumulate in the ER lumen, triggering the unfolded protein response (UPR), a cellular adaptive mechanism that restores ER homeostasis [44]. GEN accumulation in the ER induces ER stress and triggers the UPR [45]. This mechanism manifests through increased expression of ER stress markers, including *GRP78*, *CHOP*, and *caspase-12*, in renal tissues following GEN exposure [6]. Our comprehensive analysis suggested that GEN significantly upregulated all three branches of the UPR pathway: *IRE1α*, *PERK*, and *ATF6*, along with their upstream regulator *GRP78* and downstream effector *CHOP*.

EA co-treatment significantly reduced GEN-induced LC3A and KIM-1 expression, suggesting nephroprotective effects through modulation of autophagy and kidney injury markers, with limited direct impact on ROS formation. This suggests that EA’s renoprotective mechanism primarily involves regulation of cellular survival pathways rather than a simple antioxidant scavenging effect. Notably, EA co-treatment was associated with simultaneous modulation of multiple stress-response pathways, including attenuation of ER stress across all UPR branches (*GRP78*, *IRE1α*, *PERK*, *ATF6,* and *CHOP*), attenuation of apoptotic signaling (reduced *BAX* and *caspase-3*), and selective inflammatory pathway suppression (decreased IL-6 and *COX-2* expression). These actions collectively modulate renal cellular resilience against GEN-induced injury.

**Figure 12:**
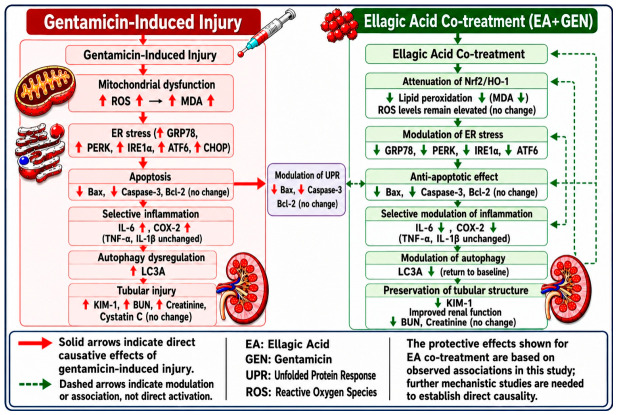
Proposed mechanistic model of gentamicin-induced nephrotoxicity and the protective effects of ellagic acid co-treatment with gentamicin. Dashed arrows indicate modulation or association. not direct activation. Solid red arrows indicate direct causative effects associated with gentamicin-induced injury, whereas dashed green arrows indicate modulation or association rather than direct activation. Upward (↑) and downward (↓) arrows represent increases and decreases in the indicated parameters.

Based on the integrated molecular, inflammatory, and histopathological findings of the present study, a comprehensive mechanistic model summarizing the protective effects of ellagic acid against gentamicin-induced nephrotoxicity is proposed in Figure 12.

Gentamicin administration is associated with mitochondrial dysfunction, oxidative stress, ER stress, apoptosis, selective inflammation, and renal dysfunction, which may ultimately contribute to tubular injury and impaired renal function. Ellagic acid co-treatment is associated with reduction in lipid peroxidation, attenuation of apoptosis and selective inflammation, and modulation of autophagy-related markers. Furthermore, EA co-treatment attenuates the gentamicin-induced enhancement of *Nrf2/HO-1* signaling. This attenuation is associated with reduced lipid peroxidation, alleviation of ER stress, and preservation of tubular structure and renal function. Although ROS levels remained elevated following EA treatment, downstream modulation of ER stress, apoptosis, inflammation, and autophagy-related pathways appears sufficient to confer renal protection. This proposed model suggests that EA co-treatment preserves renal function primarily by modulating stress-response pathways rather than direct scavenging of ROS, which is consistent with our experimental observations.

ER stress maintains intricate relationships with inflammatory, apoptotic, autophagic, and oxidative stress pathways. The significant correlation between elevated ER stress markers and increased levels of inflammatory mediators (IL-6, *COX-2*) in our study is consistent with previous findings that ER stress signaling may modulate inflammatory cascades [46]. The *IRE1α* pathway, which exhibited increased expression in our GEN model, may stimulate the JNK pathway, potentially promoting both inflammation and apoptosis by regulation pro-inflammatory cytokines and pro-apoptotic proteins [47]. The relationship between ER stress and apoptosis was suggested via concurrent upregulation of *CHOP* and pro-apoptotic factors *BAX* and *caspase-3*. *CHOP*, a downstream effector of the UPR, may contribute to *Bax/Bak* activation and subsequent caspase cascade initiation, suggesting a potential mechanistic link between prolonged ER stress and apoptotic cell death [48]. GEN’s nephrotoxic effects are associated with *CHOP* overexpression in the renal cortex, which may lead to *Bax* translocation from cytosol to mitochondria, potentially resulting in mitochondrial membrane potential dissipation, cytochrome c release, *caspase-3* activation, and apoptosis initiation [16].

Our findings also highlight the bidirectional relationship between ER stress and autophagy, as evidenced by simultaneous increases in ER stress markers and LC3A expression following GEN administration. This relationship is consistent with previous studies suggesting that ER stress may induce autophagy in various kidney diseases. potentially representing an adaptive mechanism to remove misfolded proteins and damaged organelles [49].

The observed increases in *GRP78*, *IRE1α*, PERK, *ATF6*, and *CHOP* expression in our study may suggest a mechanistic link between GEN-induced ER stress and subsequent apoptotic pathways in renal injury. The pathophysiology of GEN-induced nephrotoxicity involves a complex interplay between ER stress sensors (*IRE1* and *ATF6*), kinase signaling pathways (ERK and JNK), transcription factors (*ATF6* and *CHOP*), chaperone proteins (*BiP*), and inflammatory and apoptotic mediators [50]. ER stress-induced *CHOP* overexpression may lead to *Bax* translocation, mitochondrial dysfunction, and subsequent apoptosis initiation, potentially via cytochrome c release and *caspase-3* activation [26]. The notable upregulation of IRE1α in our study suggests that this UPR branch may play a role in GEN-induced ER stress responses. The *IRE1α* pathway activates XBP1 through its endoribonuclease activity and can stimulate inflammatory pathways via interaction with TRAF2, which could contribute to inflammatory signaling [47].

EA co-treatment significantly attenuated GEN-induced LC3A upregulation, providing further observations on the less extensively studied effects of EA on autophagy-related markers. Differential modulation of autophagy markers may exert opposing effects on tubular cell survival, with regulated autophagy potentially protecting against GEN-induced apoptosis [51]. Similarly, differential modulation of LC3A and LC3B may have opposing survival effects [52]. EA co-treatment attenuated GEN-induced ER stress, which was associated with reducing expression of *GRP78*, *IRE1α*, *PERK*, *ATF6*, and *CHOP*. These findings are consistent studies showing reports of EA downregulating ER stress sensors [12,26]. Given that *IRE1* dysfunction disrupts autophagy and TRAF2 contributes to inflammatory signaling, EA’s actions may be associated with therapeutic benefits through ER stress regulation [47]. The observed attenuation of ER stress markers suggests potential protective mechanisms that may contribute to broader nephroprotective effects.

Our findings reveal the complex interplay between multiple pathways in GEN nephrotoxicity. The observed upregulation of *Nrf2* and *HO-1* together with increased ROS formation and ER stress markers suggests an adaptive response to oxidative stress. GEN induced *Nrf2/HO-1* expression, likely as an adaptive response, and EA co-treatment attenuated this response while reducing lipid peroxidation and tissue injury. This interpretation is consistent with the observation that EA did not directly reduce ROS levels yet still conferred protection against ER stress, apoptosis, and tissue damage [53]. GEN exhibits complex effects of Nrf2, potentially activating adaptive responses or inhibiting activity depending on dose, duration, and frequency of administration [14]. This simultaneous activation of cytoprotective and pro-inflammatory mechanisms may represent adaptive cellular responses attempting to counteract oxidative damage while initiating inflammatory cascades.

EA-ER stress relationships in GEN nephrotoxicity involve complex interconnections with inflammatory, apoptotic, and oxidative pathways. EA may modulate the expression and activity, including *Nrf2*, *HO-1*, *COX-2*, *BAX*, *caspase-3*, and LC3A collectively. thereby enhancing renal cellular resilience while dampening inflammatory responses and ER stress-mediated apoptosis [54]. EA co-treatment was associated with attenuation of GEN-induced *Nrf2/HO-1* upregulation and reduction in inflammatory markers, suggesting a modulatory rather than a direct activating role [53]. Particularly noteworthy was EA’s attenuation of both the *Nrf2/HO-1* and inflammatory *COX-2* pathways, suggesting that EA reduces the underlying cellular stress burden rather than simply boosting antioxidant capacity [54,55]. EA’s protective effects extended to modulation of ER stress-mediated apoptosis, which was associated with reduced expression of ER stress markers and pro-apoptotic factors through downregulation of *GRP78* and *CHOP*, attenuating subsequent caspase activation and *BAX* upregulation [54].

Sepand et al. [22] reported that EA reduced oxidative stress and apoptosis in GEN-treated rats but did not evaluate ER stress, autophagy, or the selective IL-6/*COX-2* response; the present study integrates these pathways.

The modest but statistically significant increase in ROS observed in the GEN group is consistent with the oxidative stress reported in other nephrotoxicity models and likely contributes to cellular dysfunction. The fact that EA did not reduce this ROS elevation, yet still provided significant protection against ER stress, apoptosis, and tissue damage, suggests that EA’s primary mechanism may involve modulation of downstream signaling pathways rather than direct antioxidant scavenging. This interpretation is supported by the observed attenuation of *Nrf2/HO-1* signaling and ER stress markers. Similar observations have been reported for other phytochemicals that exert cytoprotective effects through *Nrf2* activation without directly affecting ROS levels.

### Limitations

This study has several limitations that should be acknowledged. First, although it provides comprehensive mechanistic insights into EA’s protective role against GEN-induced nephrotoxicity, mechanistic validation was restricted to mRNA expression levels, and protein-level validation by Western blotting was not performed. Lack of quantitative glomerular morphometry is another limitation of this study. Second, autophagy was assessed primarily through LC3A immunofluorescence without direct evaluation of autophagic flux using lysosomal inhibitors or LC3-II turnover assays; therefore, changes in autophagic flux cannot be confirmed. Third, mechanistic inhibition studies targeting specific UPR pathways (e.g., using gene silencing or pharmacological inhibitors) were beyond the scope of this study, and the causal involvement of ER stress, apoptosis, and autophagy in EA’s nephroprotection cannot be established from the present data. Fourth, the findings are based on a single animal model, a single dose (100 mg/kg GEN and EA), and a single time point (14 days); further investigations with varying doses, time points, and translational human models are necessary to establish generalizability and clinical relevance. Finally, a detailed evaluation of EA’s pharmacokinetics, potential interactions with aminoglycosides, and clinical trials is warranted to confirm its safety and therapeutic efficacy in patients at risk of aminoglycoside-induced nephrotoxicity.

## 5. Conclusions

While the present findings support an integrated involvement of ER stress, apoptosis, autophagy, and selective inflammation in gentamicin-induced nephrotoxicity, these associations should not be interpreted as definitive evidence of direct causal relationships.

Ellagic acid alone did not significantly affect renal parameters. However, its co-administration with gentamicin significantly mitigated renal injury. This protection was associated with reduced IL-6 and *COX-2*-associated inflammatory responses, modulation of autophagy-related markers (LC3A), alleviation of ER stress (*GRP78*, *IRE1α*, *PERK*, *ATF6, CHOP*), and attenuation of apoptotic signaling (*BAX*, *caspase-3*), which was associated with reduced tubular injury as evidenced by reduced KIM-1. Collectively, these results indicate that EA is associated with attenuation of stress-related pathways rather than establishing direct causality. Given its dietary origin and favorable safety profile, EA may represent a potential adjunctive candidate for further investigation to prevent drug-induced kidney injury. Further studies are required to elucidate the precise mechanisms involved.

## Figures and Tables

**Table 1:** Primer sequences for real-time PCR.

Gene	Sequences (5′ → 3′)	PCR Product Size (bp)
*IRE1*	F: 5′-GCAGTTCCAGTACATTGCCATTG-3′	163
	R: 5′-CAGGTCTCTGTGAACAATGTTGA-3′	
*ATF6*	F: 5′-TCAACTCAGCACGTTCCTGA-3′	130
	R: 5′-GACCAGTGACAGGCTTCTCT-3′	
*PERK*	F: 5′-GATGCCGAGAATCATGGGAA-3′	148
	R: 5′-AGATTCGAGAAGGGACTCCA-3′	
(*GRP78*/*BiP*)	F: 5′-CATGCAGTTGTGACTGTACCAG-3′	143
	R: 5′-CTCTTATCCAGGCCATATGCAA-3′	
*CHOP*	F: 5′-CGGAGTGTACCCAGCACCATCA-3′	93
	R: 5′-CCCTCTCCTTTGGTCTACCCTCA-3′	
*Nrf-2*	F: 5′-TTTGTAGATGACCATGAGTCGC-3′	161
	R: 5′-TCCTGCCAAACTTGCTCCA-3′	
*HO-1*	F: 5′-ACAGGGTGACAGAAGAGGCTA A-3′	107
	R: 5′-CTGTGAGGGACTCTGGTCTTTG-3′	
*COX-2*	F: 5′-GATTGACAGCCCACCAACTT-3′	199
	R: 5′-CGGGATGAACTCTCTCCTCA-3′	
*BAX*	F: 5′-ATGGGCTGGACACTGGACTTC-3′	147
	R: 5′-GAGCGAGGCGGTGAGGAC-3′	
*BCL-2*	F: 5′-TCCTTCCAGCCTGAGAGCAAC-3′	174
	R: 5′-GCGACGGTAGCGACGAGAG-3′	
*caspase-3*	F: 5′-GCAGCAGCCTCAAATTGTTGACTA-3′	144
	R: 5′-TGCTCCGGCTCAAACCATC-3′	
*β-actin*	F: 5′-ACTATCGGCAATGAGCGGTTC-3′	148
	R: 5′-CTGTGTTGGCATAGAGGTCTTTAC-3′	

**Table 2:** Semi-quantitative histological scoring of renal injury.

Group	Tubular Injury Score (Mean ± SEM)
Control	0.3 ± 0.2
EA	0.4 ± 0.2
GEN	3.2 ± 0.3 ***
GEN + EA	1.5 ± 0.2 **

Data are mean ± SEM (n = 5 rats per group). *** *p* < 0.001 vs. control; ** *p* < 0.01 vs. GEN.

## Data Availability

All data generated or analyzed during this study are included in this published article.

## Supplementary Materials

The following supporting information can be downloaded at: https://www.mdpi.com/article/10.3390/biomedicines14061385/s1.

## Abbreviations

ANOVA, analysis of variance; *ATF6*, activating transcription factor 6; *BAX*, *Bcl-2*-associated X protein; *Bcl-2*, B-cell lymphoma 2; CAT, catalase; *CHOP*, C/EBP homologous protein; *COX-2*, cyclooxygenase-2; DMSO, dimethyl sulfoxide; EA, ellagic acid; ELISA, enzyme-linked immunosorbent assay; ER, endoplasmic reticulum; FBS, fetal bovine serum; GEN, gentamicin; *GRP78*, glucose-regulated protein 78; GSH, reduced glutathione; *HO-1*, heme oxygenase-1; HSP70, heat shock protein 70; *IRE1α,* inositol-requiring enzyme 1 alpha; IL-6, interleukin-6; KIM-1, kidney injury molecule-1; LC3A, microtubule-associated protein 1 light chain 3 alpha; MDA, malondialdehyde; mRNA, messenger ribonucleic acid; *Nrf2*, nuclear factor erythroid 2-related factor 2; PBS, phosphate-buffered saline; PCR, polymerase chain reaction; *PERK*, protein kinase RNA-like endoplasmic reticulum kinase; qPCR, quantitative polymerase chain reaction; ROS, reactive oxygen species; SD, standard deviation; SEM, standard error of the mean; SOD, superoxide dismutase; TBARS, thiobarbituric acid reactive substances; TUNEL, terminal deoxynucleotidyl transferase dUTP nick end labeling.
