# Supplementary material for: Ellagic Acid Attenuates Gentamicin Nephrotoxicity by Integrated Modulation of ER Stress-Associated Apoptosis-Autophagy Crosstalk and Attenuation of Nrf2/HO-1 Signaling
Source: Biomedicines. 2026 Jun 19;14(6):1385. doi: 10.3390/biomedicines14061385 (PMC13296924; doi:10.3390/biomedicines14061385)
Supplement: Supplementary file 1 [file biomedicines-14-01385-s001.zip › biomedicines-4229880_Raw_Images_Figures_7-11.zipw folder/Protein Expression (IF) .Results.pptx]

## Slide 1
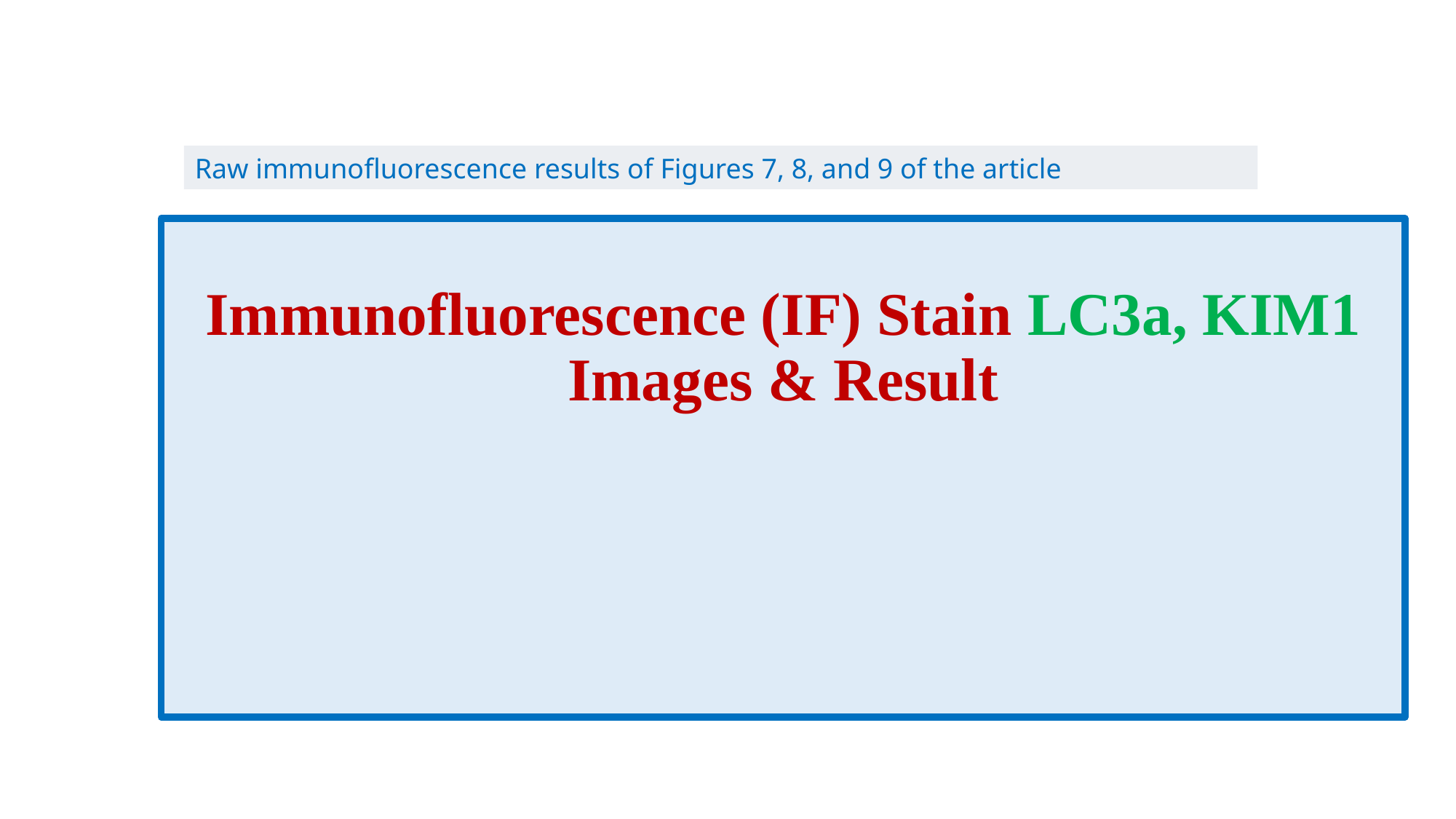

Raw immunofluorescence results of Figures 7, 8, and 9 of the article
Immunofluorescence (IF) Stain LC3a, KIM1
Images & Result

## Slide 2
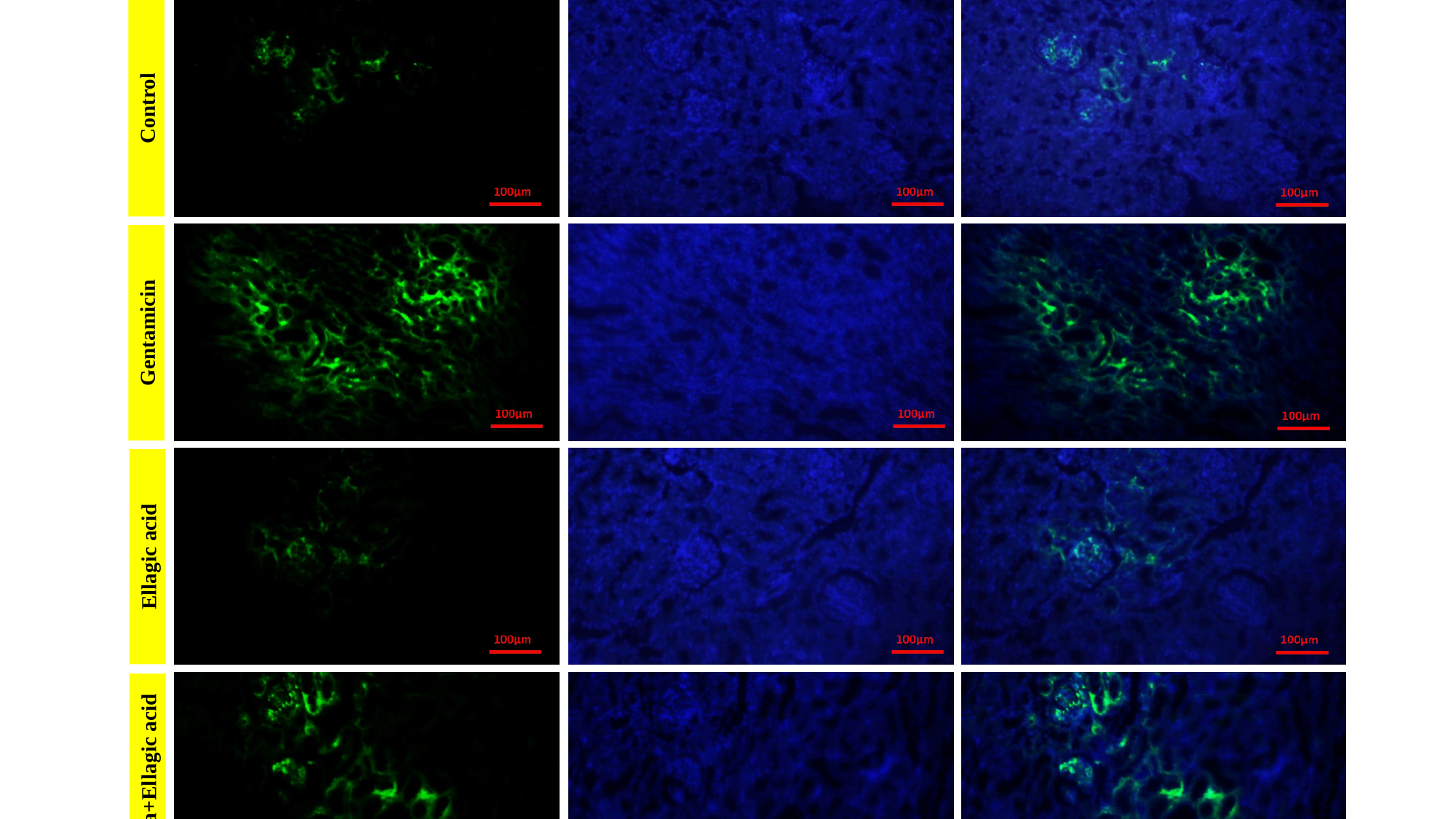

LC3a
Merged
DAPI
Control
Gentamicin
Ellagic acid
Genta+Ellagic acid

## Slide 3
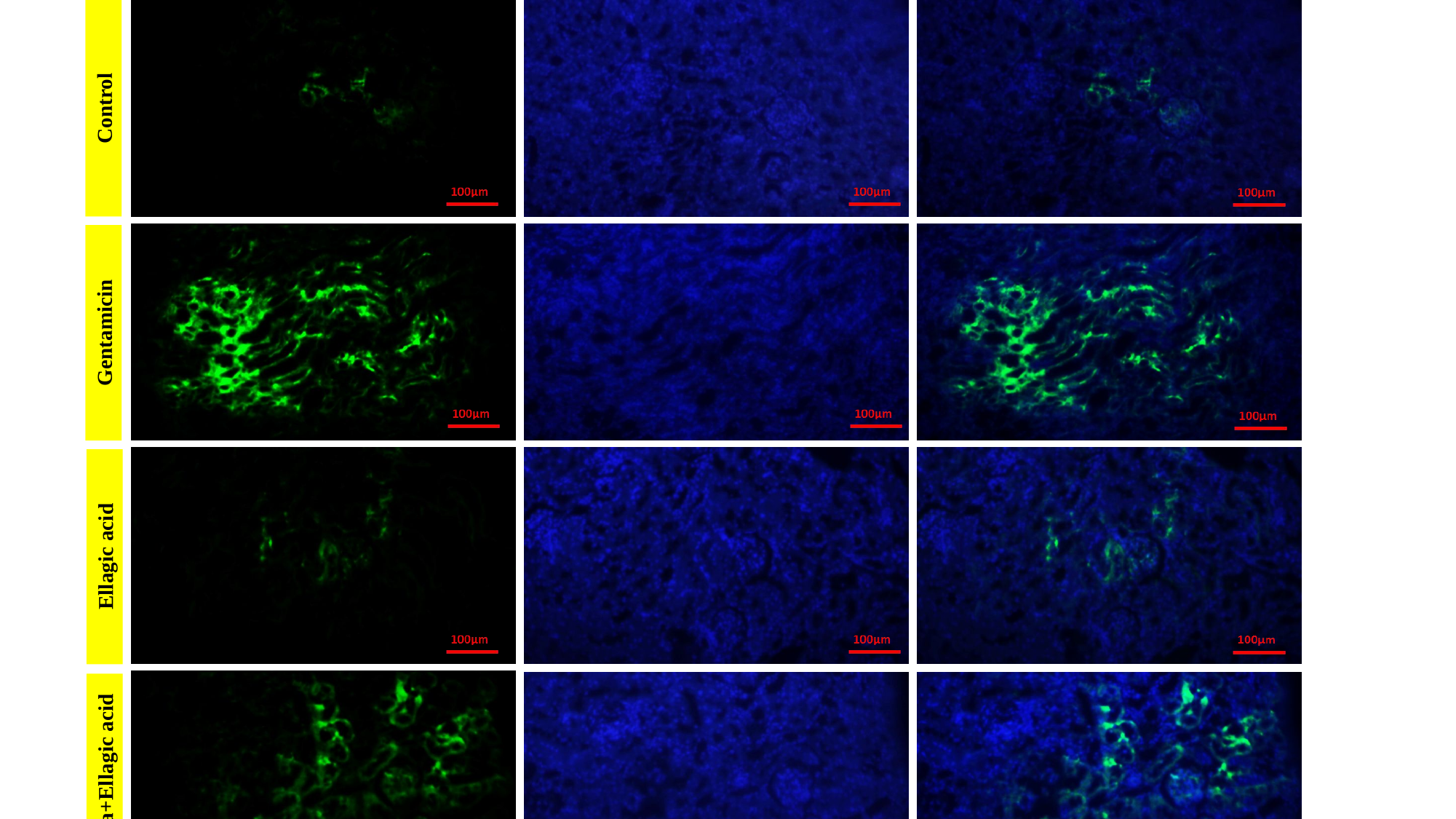

LC3a
Merged
DAPI
Control
Gentamicin
Ellagic acid
Genta+Ellagic acid

## Slide 4
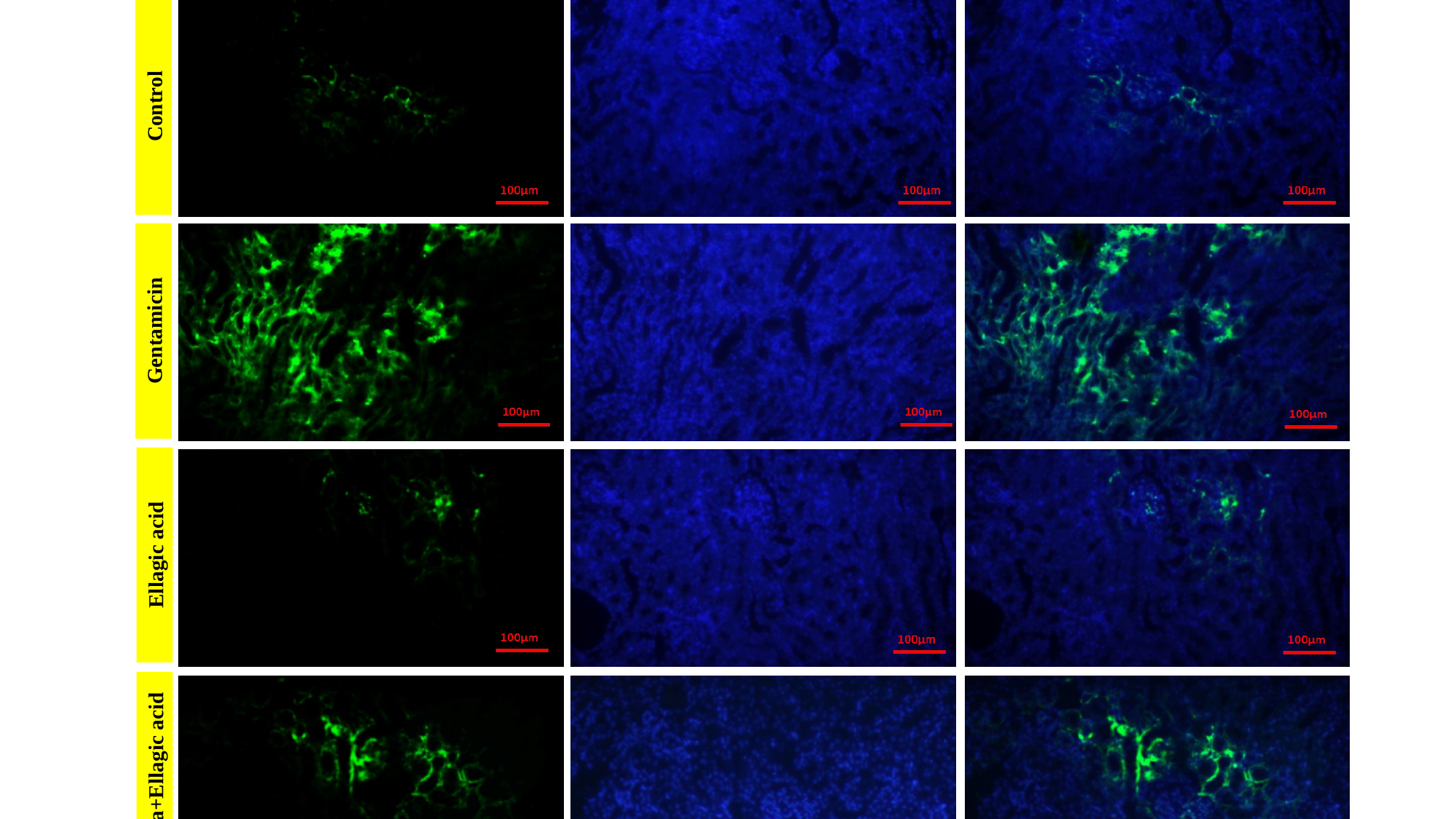

LC3a
Merged
DAPI
Control
Gentamicin
Ellagic acid
Genta+Ellagic acid

## Slide 5
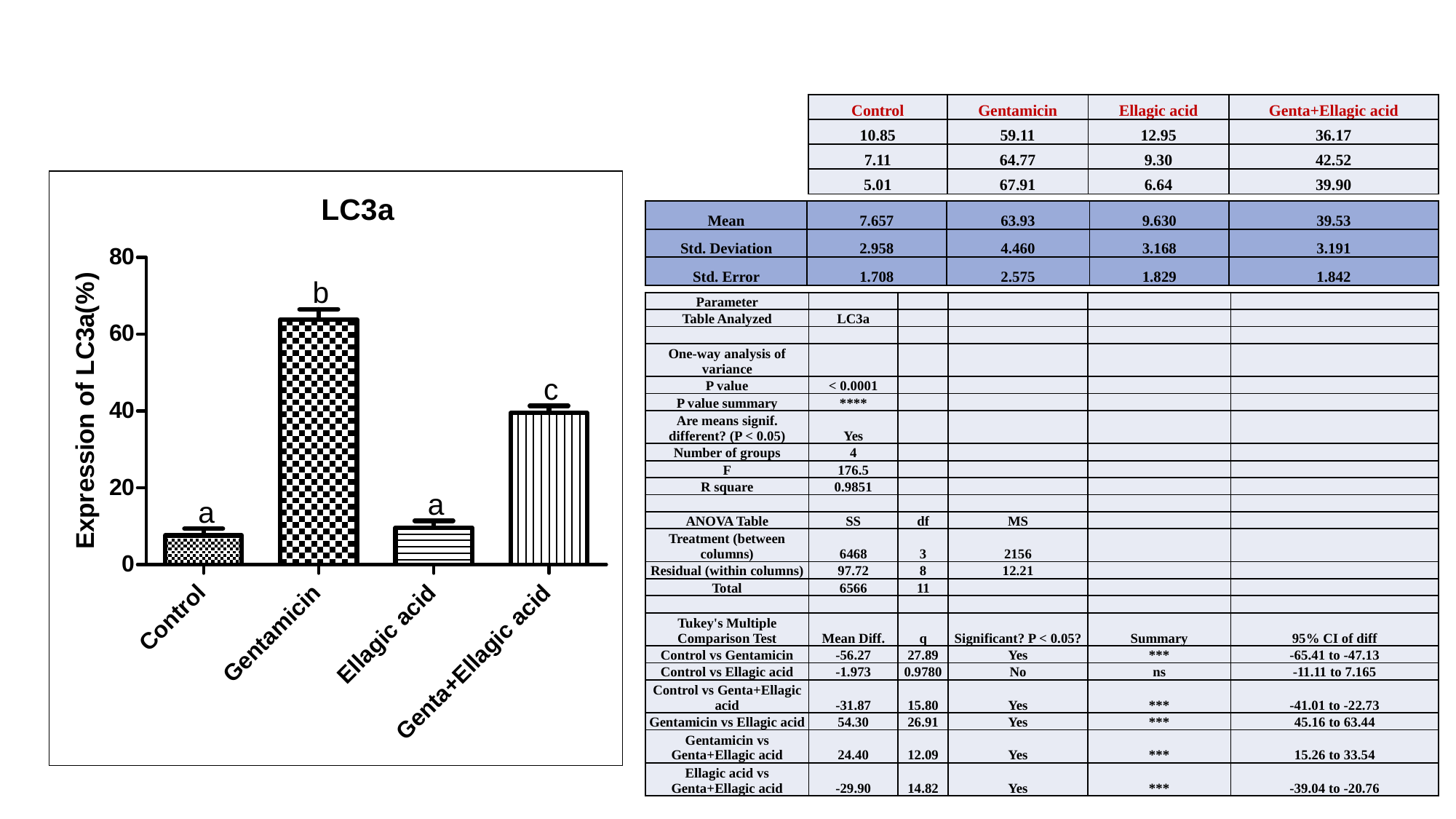

| Control | Gentamicin | Ellagic acid | Genta+Ellagic acid |
| --- | --- | --- | --- |
| 10.85 | 59.11 | 12.95 | 36.17 |
| 7.11 | 64.77 | 9.30 | 42.52 |
| 5.01 | 67.91 | 6.64 | 39.90 |
| Mean | 7.657 | 63.93 | 9.630 | 39.53 |
| --- | --- | --- | --- | --- |
| Std. Deviation | 2.958 | 4.460 | 3.168 | 3.191 |
| Std. Error | 1.708 | 2.575 | 1.829 | 1.842 |
| Parameter | | | | | |
| --- | --- | --- | --- | --- | --- |
| Table Analyzed | LC3a | | | | |
| | | | | | |
| One-way analysis of variance | | | | | |
| P value | < 0.0001 | | | | |
| P value summary | \*\*\*\* | | | | |
| Are means signif. different? (P < 0.05) | Yes | | | | |
| Number of groups | 4 | | | | |
| F | 176.5 | | | | |
| R square | 0.9851 | | | | |
| | | | | | |
| ANOVA Table | SS | df | MS | | |
| Treatment (between columns) | 6468 | 3 | 2156 | | |
| Residual (within columns) | 97.72 | 8 | 12.21 | | |
| Total | 6566 | 11 | | | |
| | | | | | |
| Tukey's Multiple Comparison Test | Mean Diff. | q | Significant? P < 0.05? | Summary | 95% CI of diff |
| Control vs Gentamicin | -56.27 | 27.89 | Yes | \*\*\* | -65.41 to -47.13 |
| Control vs Ellagic acid | -1.973 | 0.9780 | No | ns | -11.11 to 7.165 |
| Control vs Genta+Ellagic acid | -31.87 | 15.80 | Yes | \*\*\* | -41.01 to -22.73 |
| Gentamicin vs Ellagic acid | 54.30 | 26.91 | Yes | \*\*\* | 45.16 to 63.44 |
| Gentamicin vs Genta+Ellagic acid | 24.40 | 12.09 | Yes | \*\*\* | 15.26 to 33.54 |
| Ellagic acid vs Genta+Ellagic acid | -29.90 | 14.82 | Yes | \*\*\* | -39.04 to -20.76 |

## Slide 6
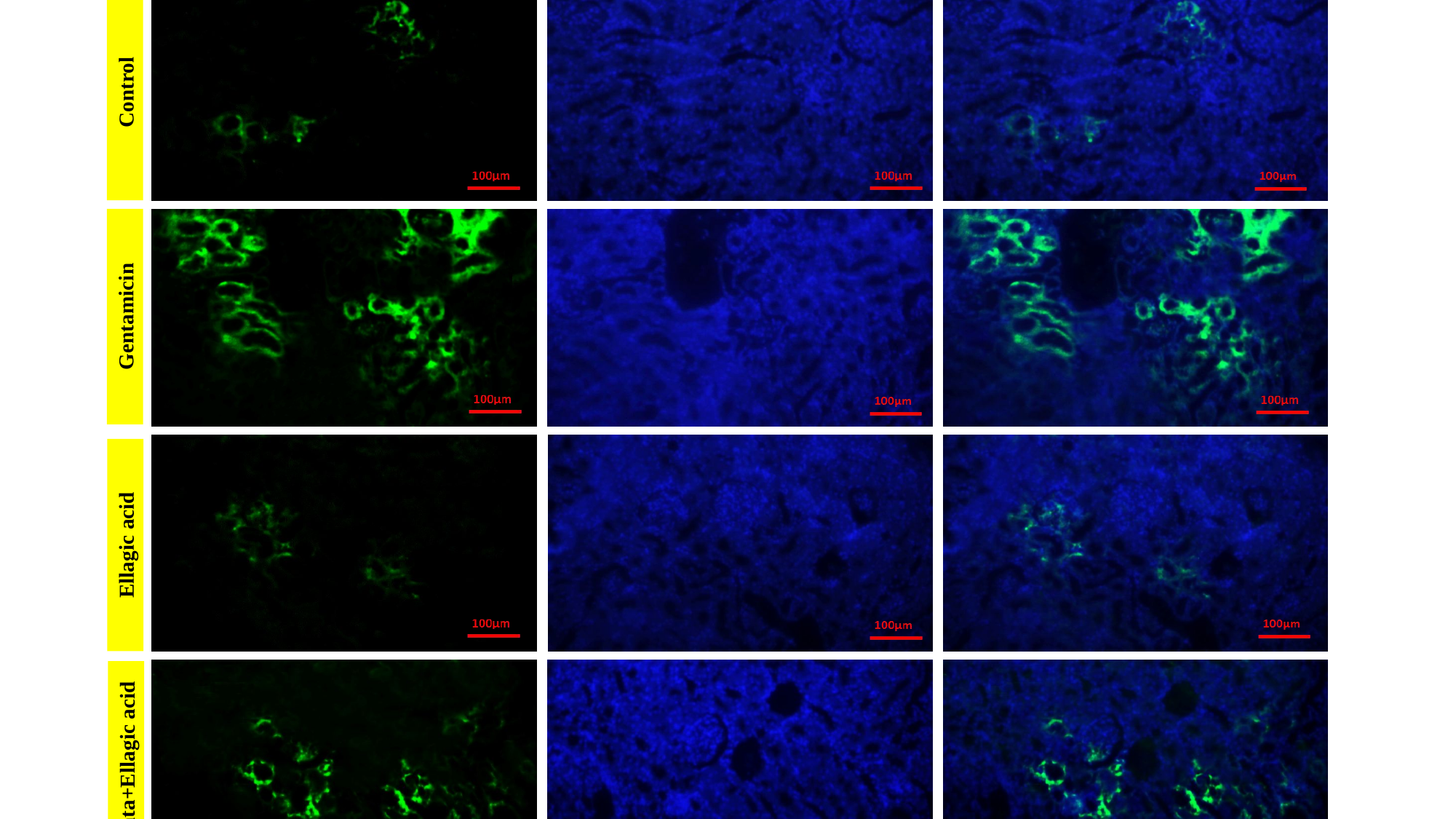

KIM-1
Merged
DAPI
Control
Gentamicin
Ellagic acid
Genta+Ellagic acid

## Slide 7
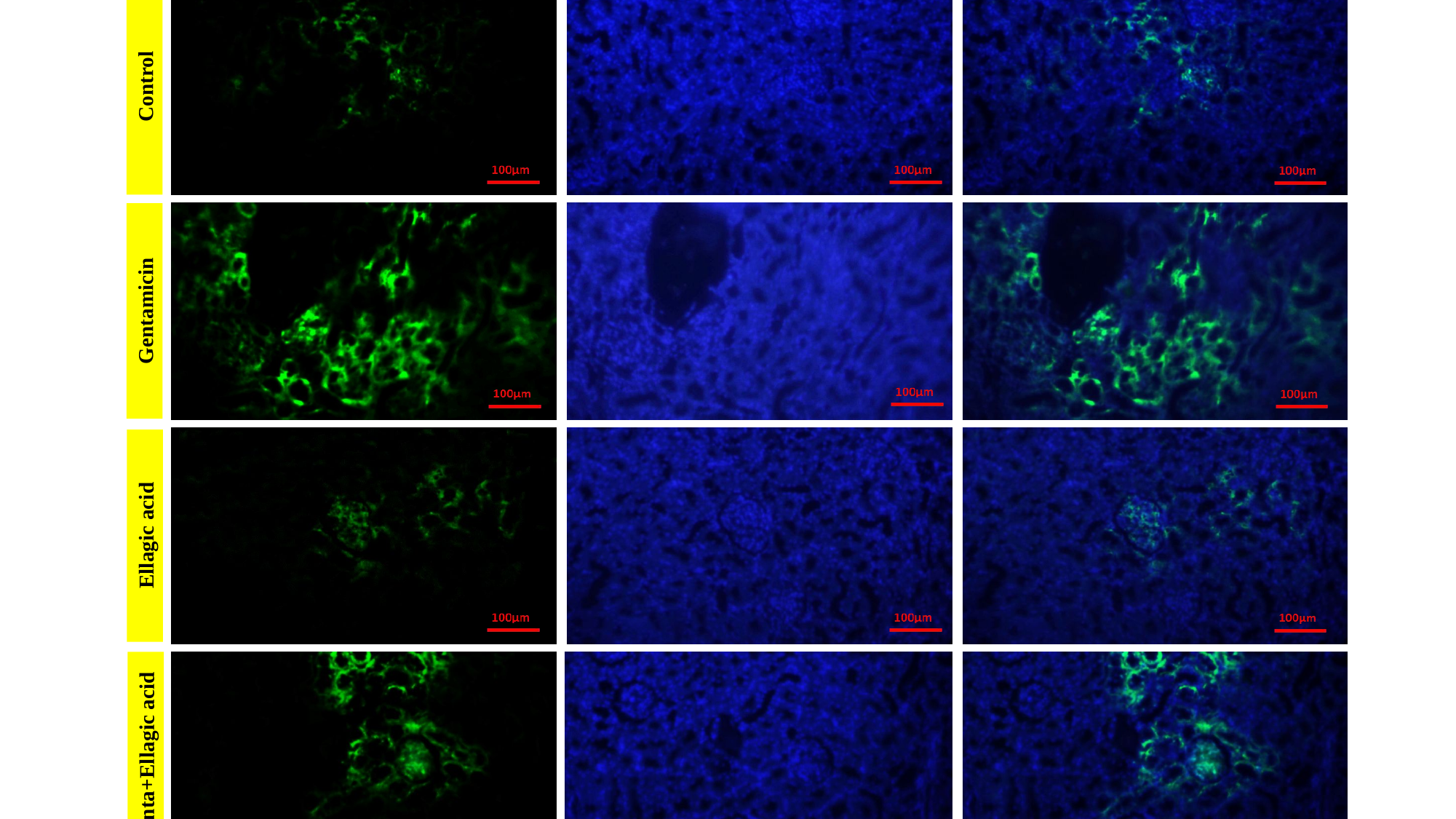

KIM-1
Merged
DAPI
Control
Gentamicin
Ellagic acid
Genta+Ellagic acid

## Slide 8
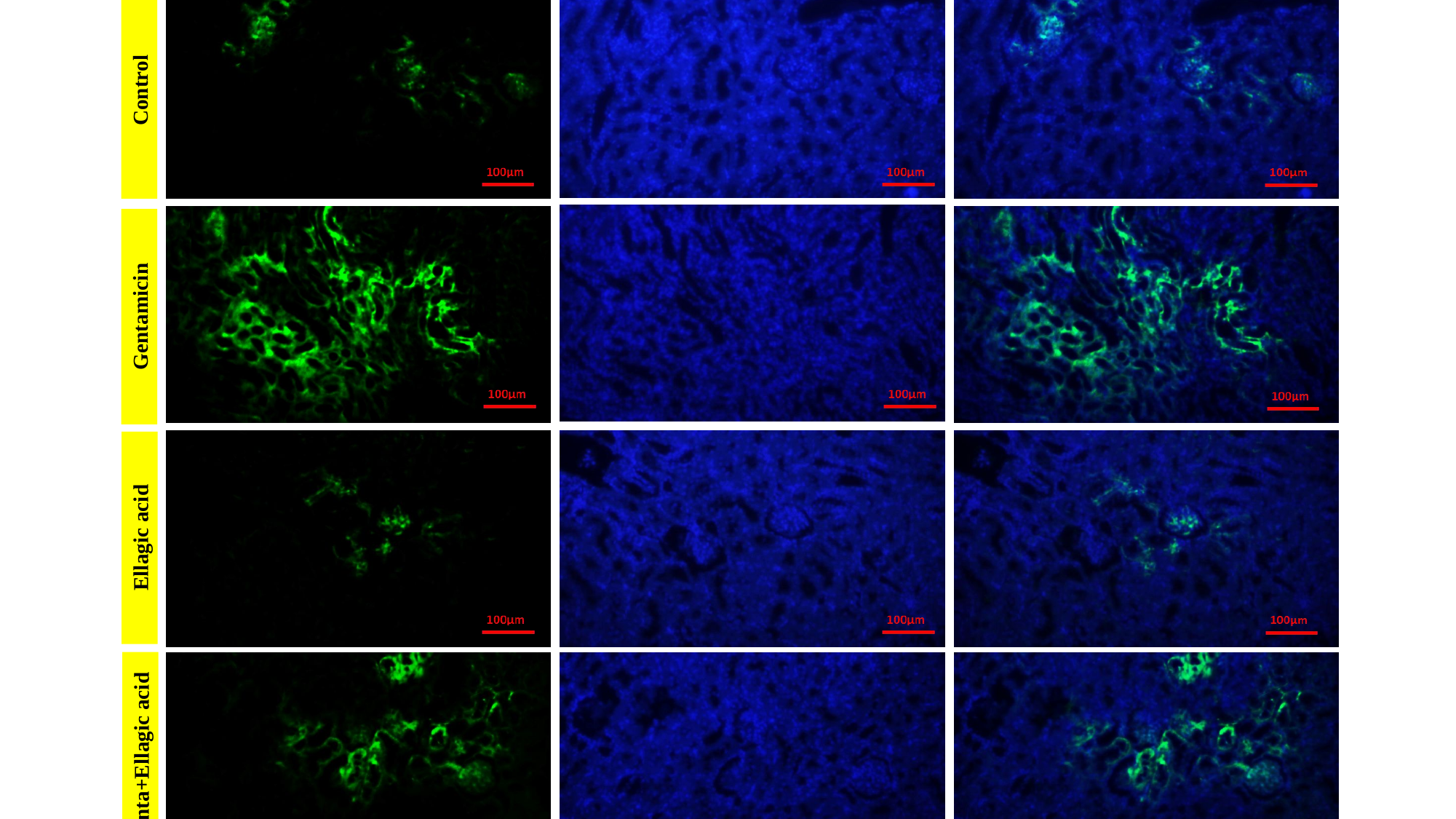

DAPI
KIM-1
Merged
Control
Gentamicin
Ellagic acid
Genta+Ellagic acid

## Slide 9
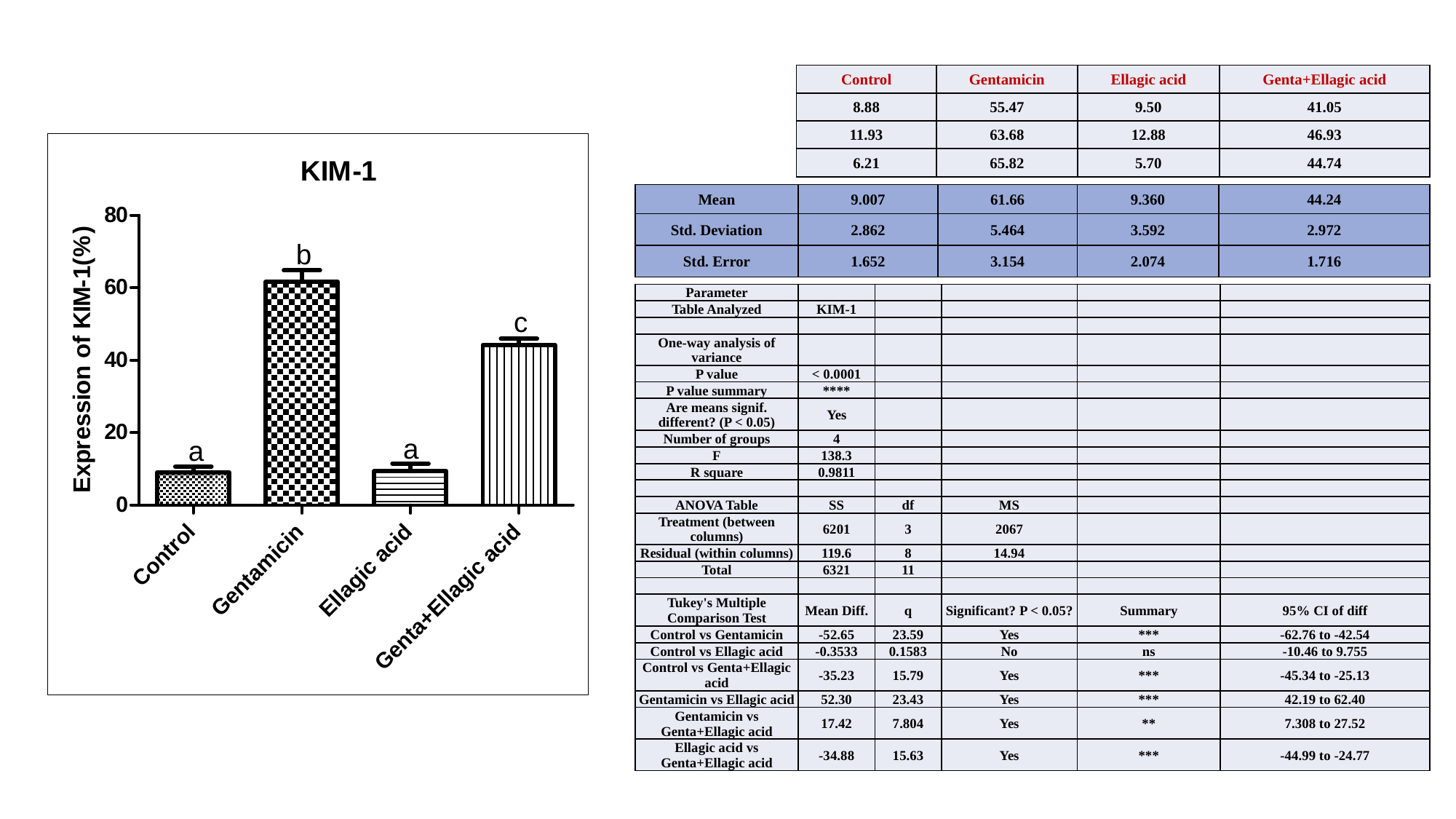

| Control | Gentamicin | Ellagic acid | Genta+Ellagic acid |
| --- | --- | --- | --- |
| 8.88 | 55.47 | 9.50 | 41.05 |
| 11.93 | 63.68 | 12.88 | 46.93 |
| 6.21 | 65.82 | 5.70 | 44.74 |
| Mean | 9.007 | 61.66 | 9.360 | 44.24 |
| --- | --- | --- | --- | --- |
| Std. Deviation | 2.862 | 5.464 | 3.592 | 2.972 |
| Std. Error | 1.652 | 3.154 | 2.074 | 1.716 |
| Parameter | | | | | |
| --- | --- | --- | --- | --- | --- |
| Table Analyzed | KIM-1 | | | | |
| | | | | | |
| One-way analysis of variance | | | | | |
| P value | < 0.0001 | | | | |
| P value summary | \*\*\*\* | | | | |
| Are means signif. different? (P < 0.05) | Yes | | | | |
| Number of groups | 4 | | | | |
| F | 138.3 | | | | |
| R square | 0.9811 | | | | |
| | | | | | |
| ANOVA Table | SS | df | MS | | |
| Treatment (between columns) | 6201 | 3 | 2067 | | |
| Residual (within columns) | 119.6 | 8 | 14.94 | | |
| Total | 6321 | 11 | | | |
| | | | | | |
| Tukey's Multiple Comparison Test | Mean Diff. | q | Significant? P < 0.05? | Summary | 95% CI of diff |
| Control vs Gentamicin | -52.65 | 23.59 | Yes | \*\*\* | -62.76 to -42.54 |
| Control vs Ellagic acid | -0.3533 | 0.1583 | No | ns | -10.46 to 9.755 |
| Control vs Genta+Ellagic acid | -35.23 | 15.79 | Yes | \*\*\* | -45.34 to -25.13 |
| Gentamicin vs Ellagic acid | 52.30 | 23.43 | Yes | \*\*\* | 42.19 to 62.40 |
| Gentamicin vs Genta+Ellagic acid | 17.42 | 7.804 | Yes | \*\* | 7.308 to 27.52 |
| Ellagic acid vs Genta+Ellagic acid | -34.88 | 15.63 | Yes | \*\*\* | -44.99 to -24.77 |
