# Supplementary figures and images for: Ellagic Acid Attenuates Gentamicin Nephrotoxicity by Integrated Modulation of ER Stress-Associated Apoptosis-Autophagy Crosstalk and Attenuation of Nrf2/HO-1 Signaling (part 2 of 2)
Source: Biomedicines. 2026 Jun 19;14(6):1385. doi: 10.3390/biomedicines14061385 (PMC13296924; doi:10.3390/biomedicines14061385)

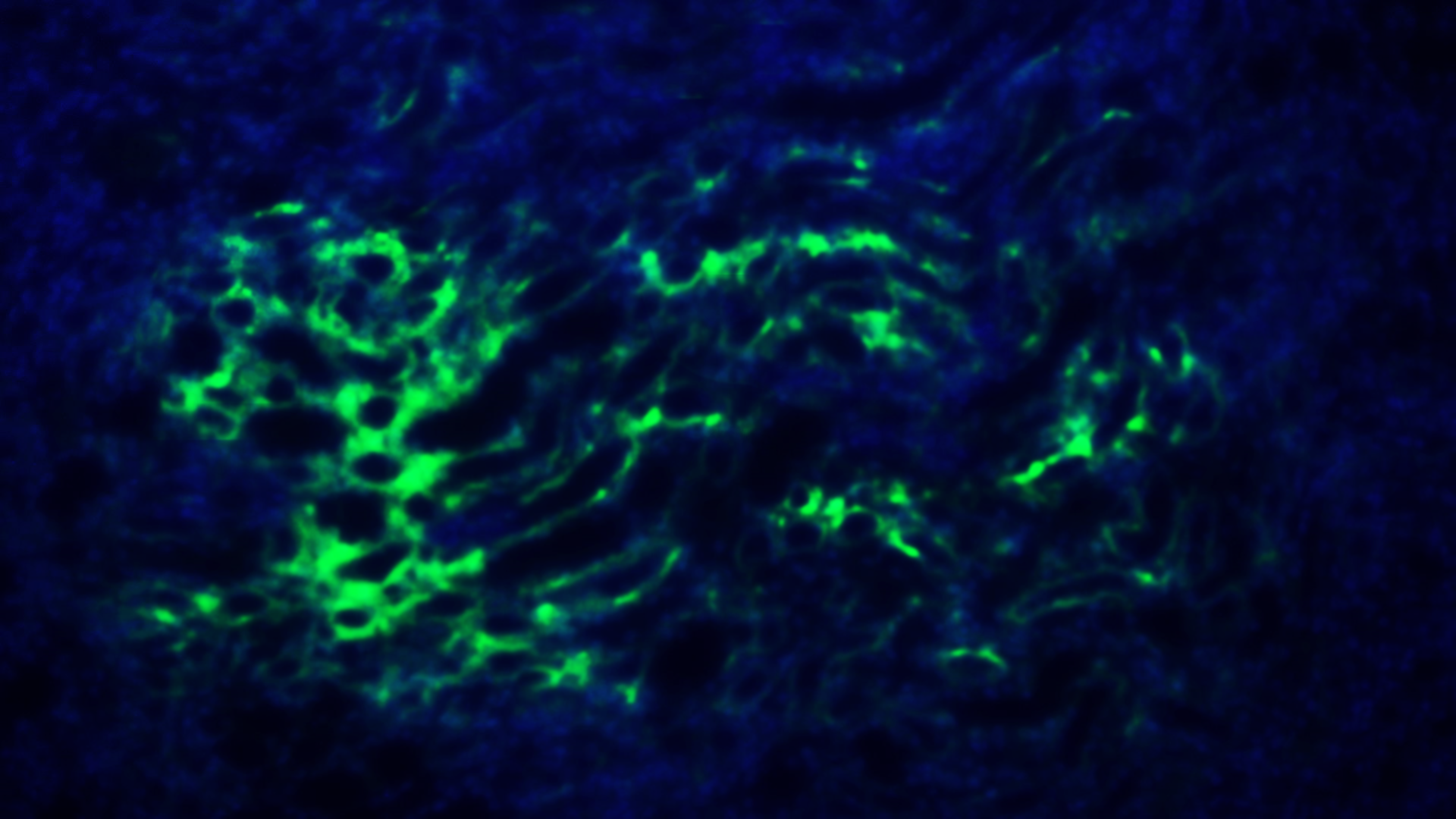

Supplement: Supplementary file 1 [file biomedicines-14-01385-s001.zip › biomedicines-4229880_Raw_Images_Figures_7-11.zipw folder/Original microscopy imgesRaw immunofluorescence results of Figures 7, 8, and 9 of the article/LC3A/Gentamicin/2/3.tif]

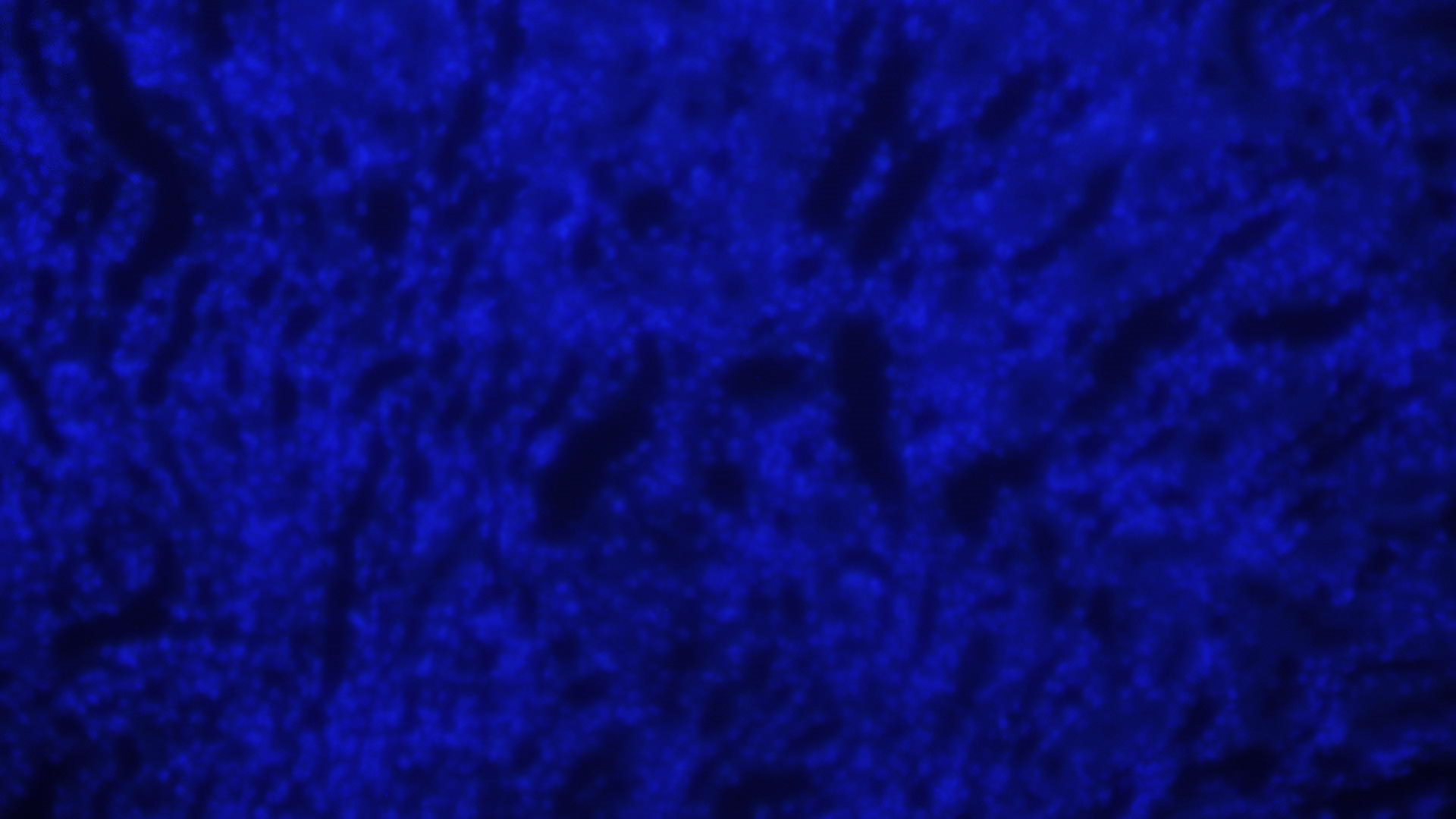

Supplement: Supplementary file 1 [file biomedicines-14-01385-s001.zip › biomedicines-4229880_Raw_Images_Figures_7-11.zipw folder/Original microscopy imgesRaw immunofluorescence results of Figures 7, 8, and 9 of the article/LC3A/Gentamicin/3/1.tif]

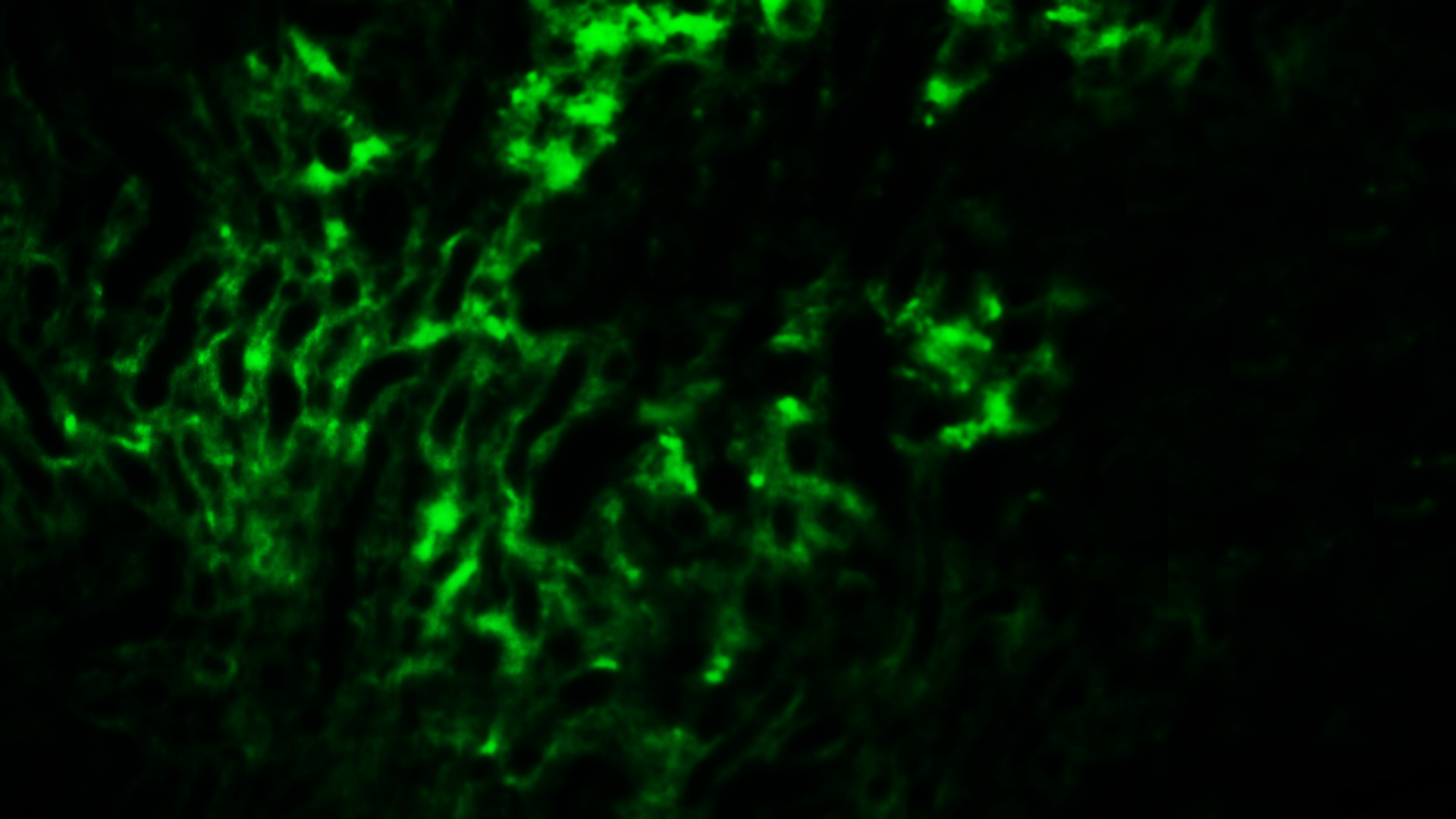

Supplement: Supplementary file 1 [file biomedicines-14-01385-s001.zip › biomedicines-4229880_Raw_Images_Figures_7-11.zipw folder/Original microscopy imgesRaw immunofluorescence results of Figures 7, 8, and 9 of the article/LC3A/Gentamicin/3/2.tif]

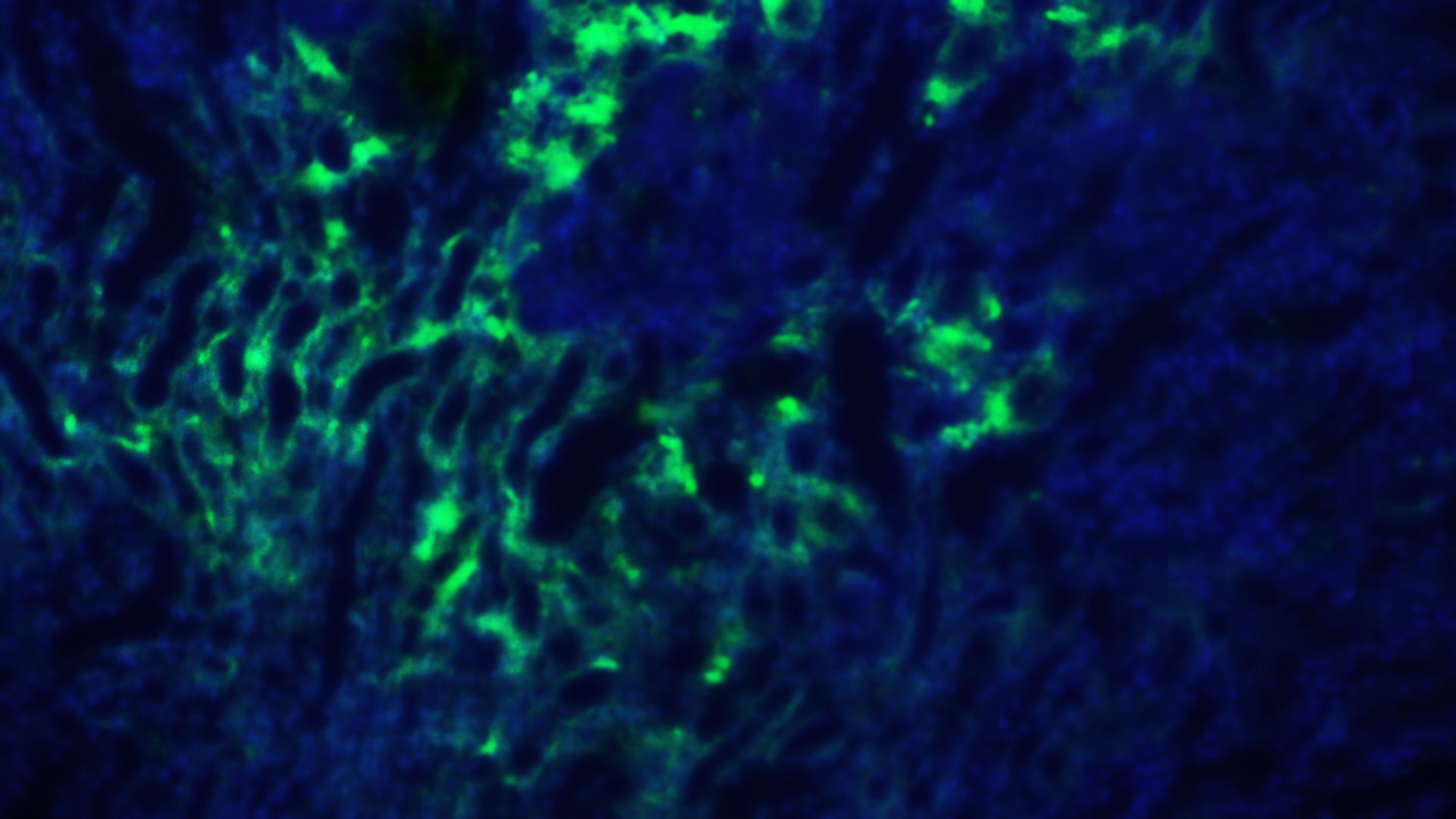

Supplement: Supplementary file 1 [file biomedicines-14-01385-s001.zip › biomedicines-4229880_Raw_Images_Figures_7-11.zipw folder/Original microscopy imgesRaw immunofluorescence results of Figures 7, 8, and 9 of the article/LC3A/Gentamicin/3/3.tif]
