# Supplementary figures and images for: Ellagic Acid Attenuates Gentamicin Nephrotoxicity by Integrated Modulation of ER Stress-Associated Apoptosis-Autophagy Crosstalk and Attenuation of Nrf2/HO-1 Signaling (part 1 of 2)
Source: Biomedicines. 2026 Jun 19;14(6):1385. doi: 10.3390/biomedicines14061385 (PMC13296924; doi:10.3390/biomedicines14061385)

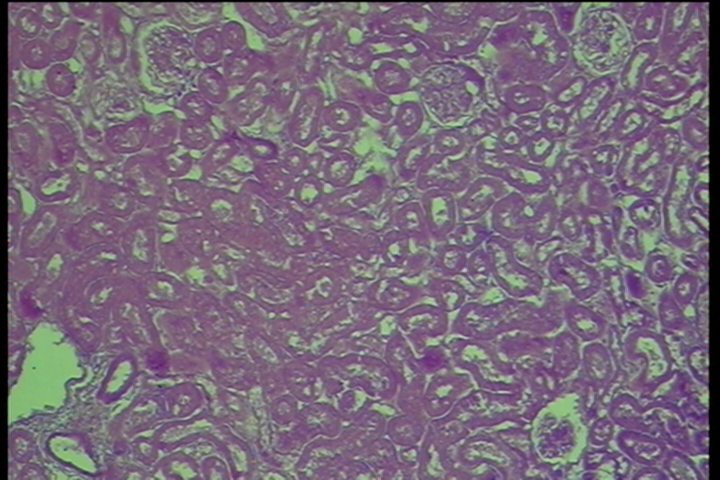

Supplement: Supplementary file 1 [file biomedicines-14-01385-s001.zip › biomedicines-4229880_Raw_Images_Figures_7-11.zipw folder/6624109822729330434_63719530759489/kidney/Cortex/Control/0000.bmp]

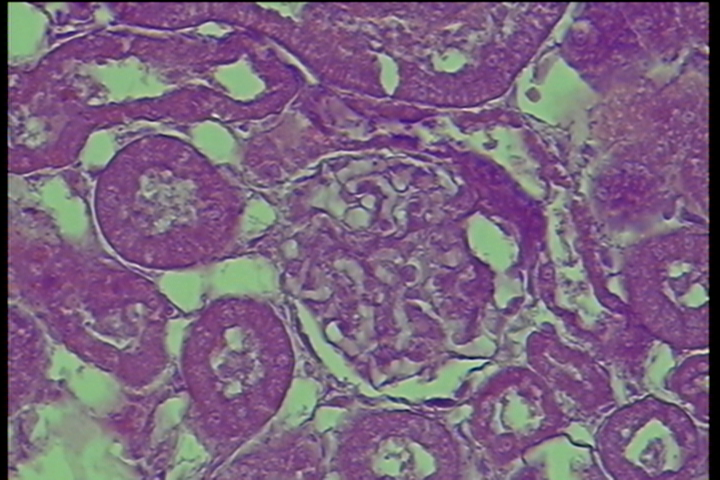

Supplement: Supplementary file 1 [file biomedicines-14-01385-s001.zip › biomedicines-4229880_Raw_Images_Figures_7-11.zipw folder/6624109822729330434_63719530759489/kidney/Cortex/Control/0002.bmp]

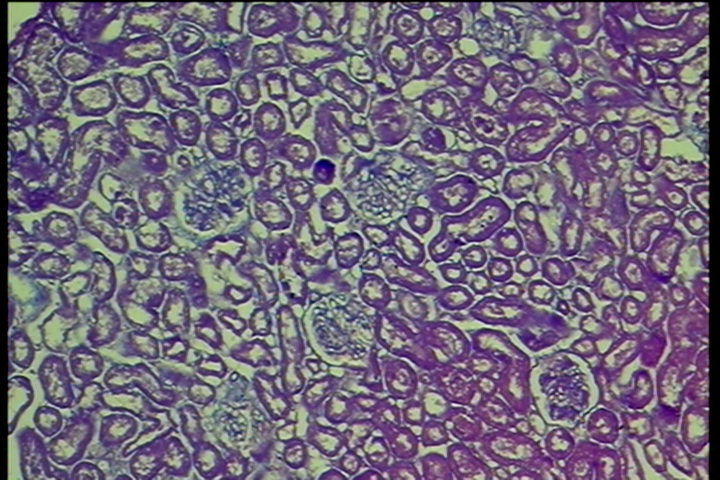

Supplement: Supplementary file 1 [file biomedicines-14-01385-s001.zip › biomedicines-4229880_Raw_Images_Figures_7-11.zipw folder/6624109822729330434_63719530759489/kidney/Cortex/Control/0006.bmp]

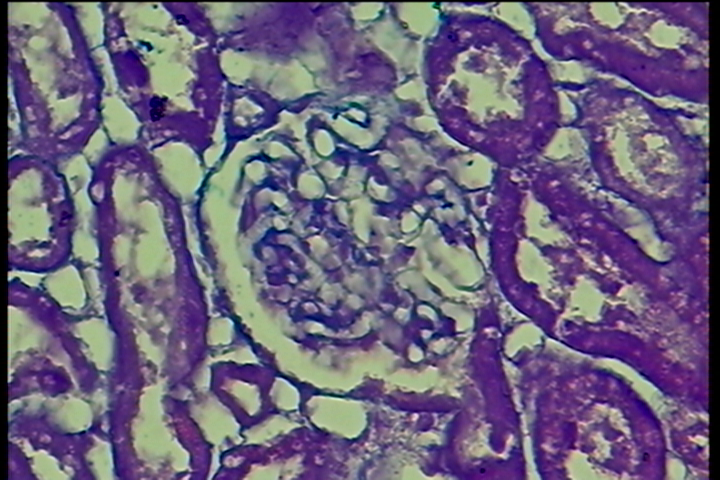

Supplement: Supplementary file 1 [file biomedicines-14-01385-s001.zip › biomedicines-4229880_Raw_Images_Figures_7-11.zipw folder/6624109822729330434_63719530759489/kidney/Cortex/Control/0007.bmp]

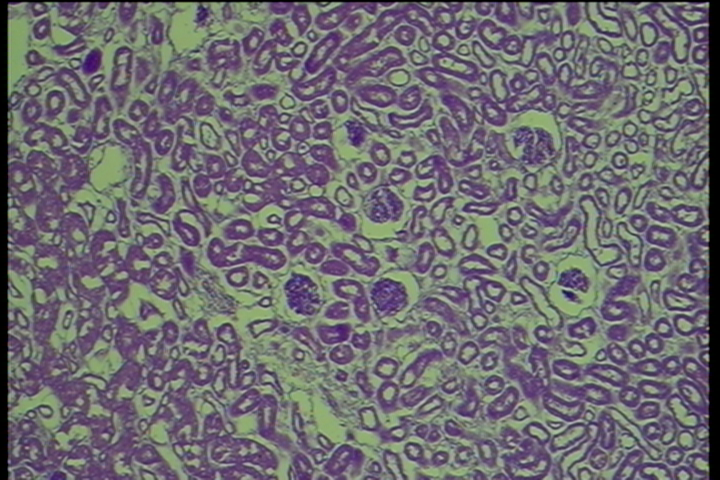

Supplement: Supplementary file 1 [file biomedicines-14-01385-s001.zip › biomedicines-4229880_Raw_Images_Figures_7-11.zipw folder/6624109822729330434_63719530759489/kidney/Cortex/Ella Genta/0000.bmp]

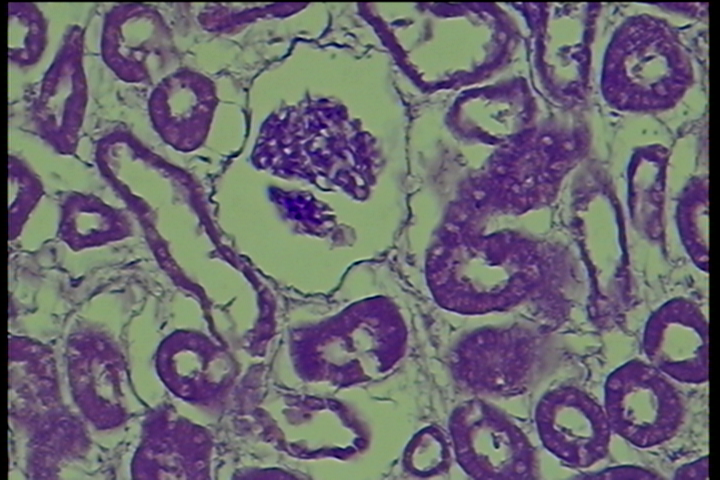

Supplement: Supplementary file 1 [file biomedicines-14-01385-s001.zip › biomedicines-4229880_Raw_Images_Figures_7-11.zipw folder/6624109822729330434_63719530759489/kidney/Cortex/Ella Genta/0002.bmp]

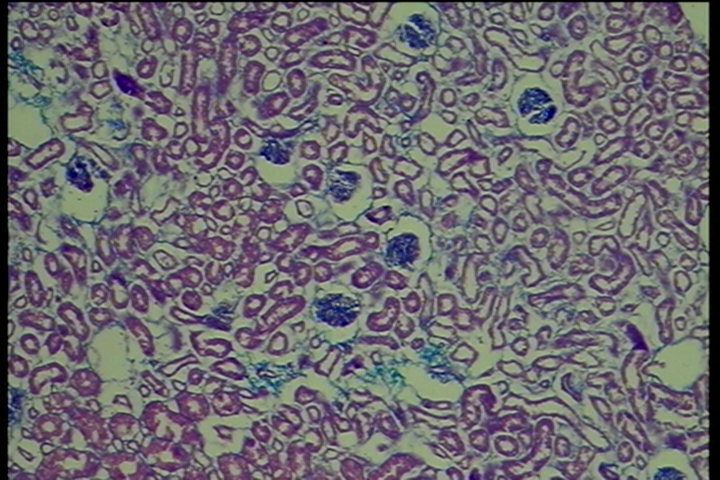

Supplement: Supplementary file 1 [file biomedicines-14-01385-s001.zip › biomedicines-4229880_Raw_Images_Figures_7-11.zipw folder/6624109822729330434_63719530759489/kidney/Cortex/Ella Genta/0004.bmp]

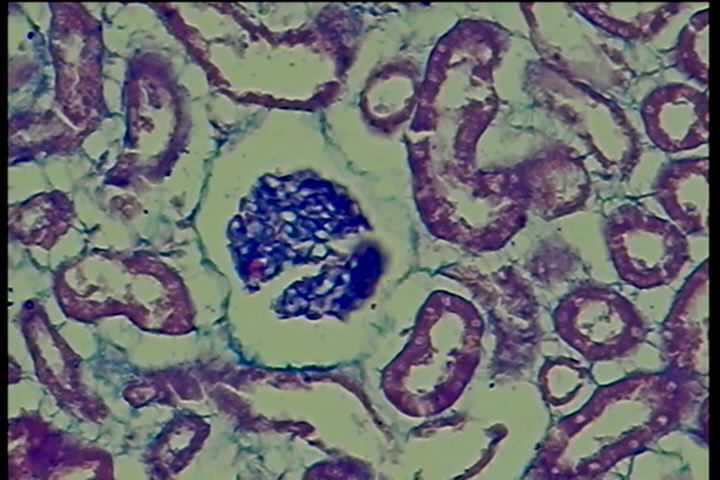

Supplement: Supplementary file 1 [file biomedicines-14-01385-s001.zip › biomedicines-4229880_Raw_Images_Figures_7-11.zipw folder/6624109822729330434_63719530759489/kidney/Cortex/Ella Genta/0005.bmp]

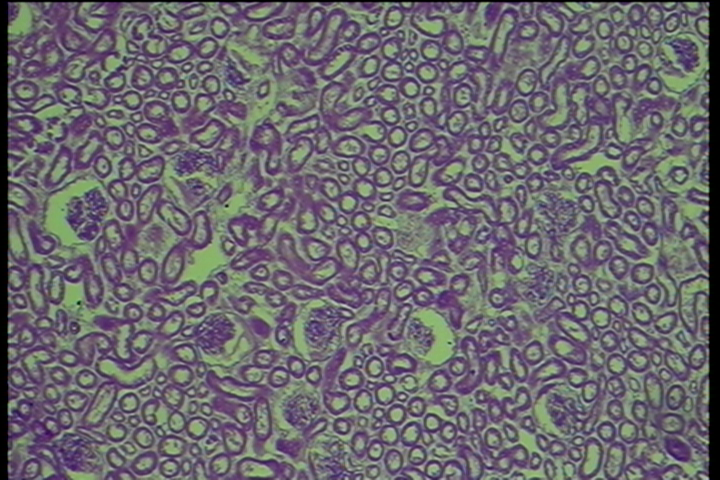

Supplement: Supplementary file 1 [file biomedicines-14-01385-s001.zip › biomedicines-4229880_Raw_Images_Figures_7-11.zipw folder/6624109822729330434_63719530759489/kidney/Cortex/Ella/0000.bmp]

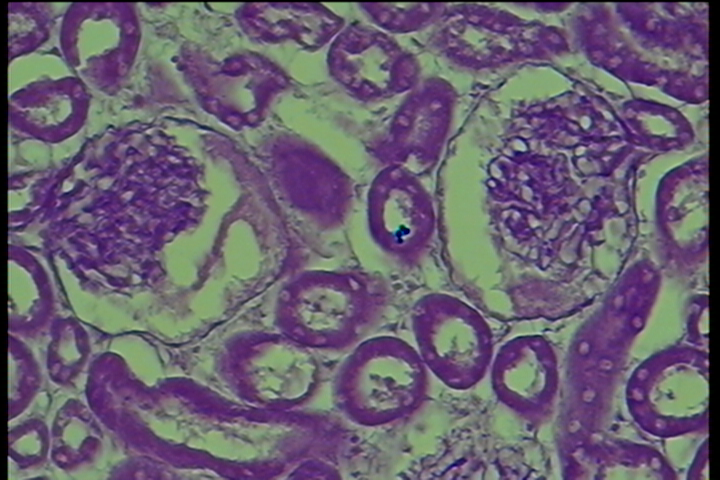

Supplement: Supplementary file 1 [file biomedicines-14-01385-s001.zip › biomedicines-4229880_Raw_Images_Figures_7-11.zipw folder/6624109822729330434_63719530759489/kidney/Cortex/Ella/0002.bmp]

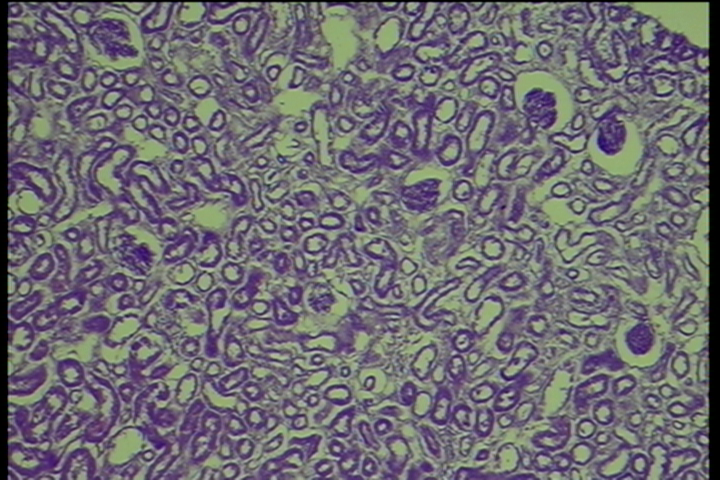

Supplement: Supplementary file 1 [file biomedicines-14-01385-s001.zip › biomedicines-4229880_Raw_Images_Figures_7-11.zipw folder/6624109822729330434_63719530759489/kidney/Cortex/Ella/00200.bmp]

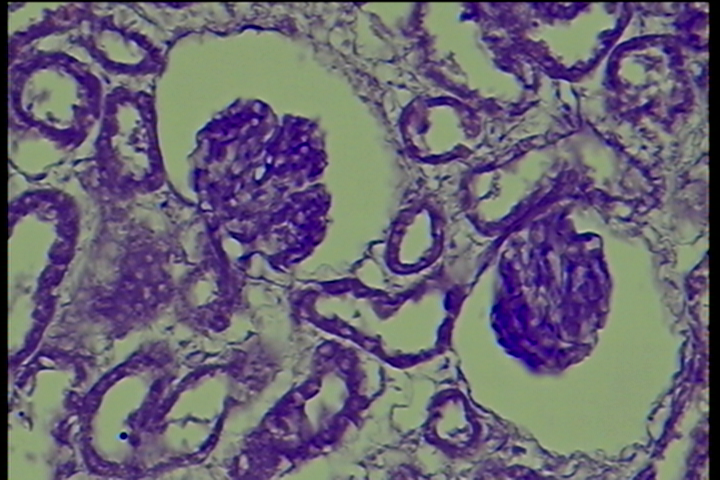

Supplement: Supplementary file 1 [file biomedicines-14-01385-s001.zip › biomedicines-4229880_Raw_Images_Figures_7-11.zipw folder/6624109822729330434_63719530759489/kidney/Cortex/Ella/00201.bmp]

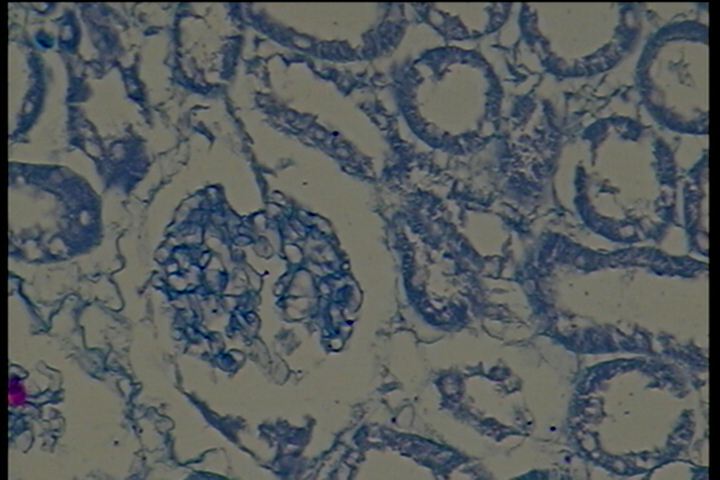

Supplement: Supplementary file 1 [file biomedicines-14-01385-s001.zip › biomedicines-4229880_Raw_Images_Figures_7-11.zipw folder/6624109822729330434_63719530759489/kidney/Cortex/Genta/0002.bmp]

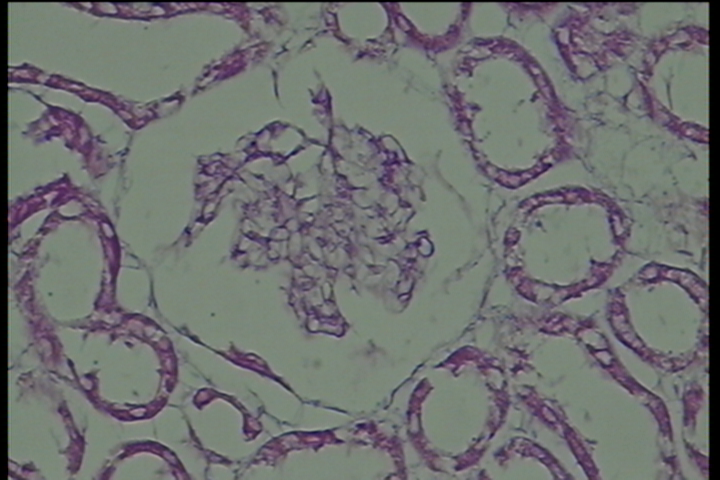

Supplement: Supplementary file 1 [file biomedicines-14-01385-s001.zip › biomedicines-4229880_Raw_Images_Figures_7-11.zipw folder/6624109822729330434_63719530759489/kidney/Cortex/Genta/c 1 he.bmp]

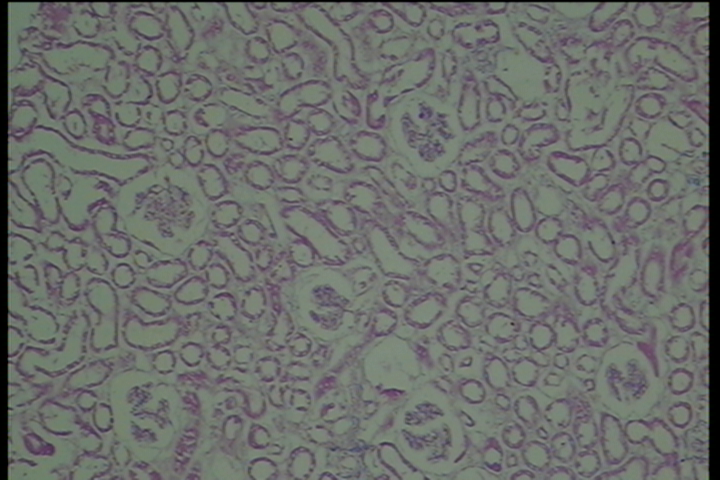

Supplement: Supplementary file 1 [file biomedicines-14-01385-s001.zip › biomedicines-4229880_Raw_Images_Figures_7-11.zipw folder/6624109822729330434_63719530759489/kidney/Cortex/Genta/c 10x he.bmp]

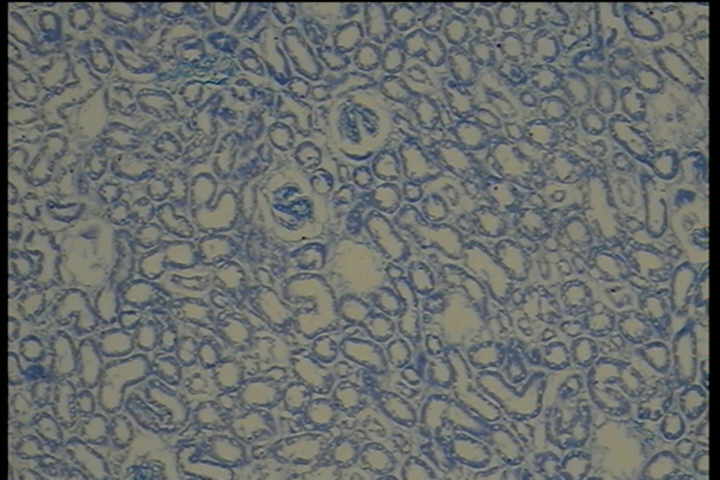

Supplement: Supplementary file 1 [file biomedicines-14-01385-s001.zip › biomedicines-4229880_Raw_Images_Figures_7-11.zipw folder/6624109822729330434_63719530759489/kidney/Cortex/Genta/c 10x mt.bmp]

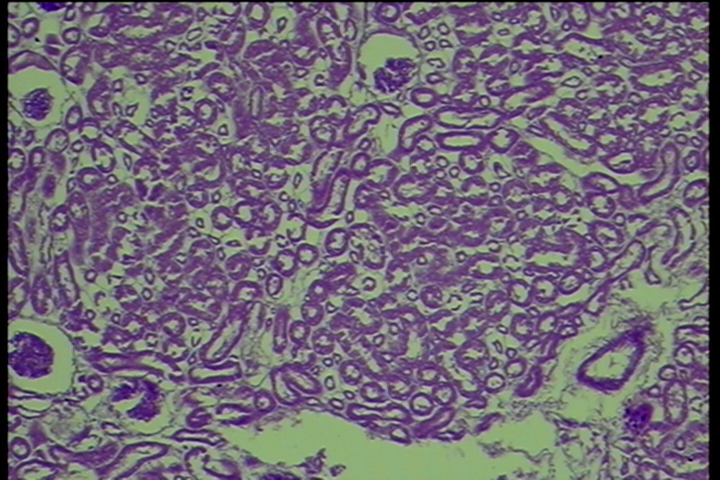

Supplement: Supplementary file 1 [file biomedicines-14-01385-s001.zip › biomedicines-4229880_Raw_Images_Figures_7-11.zipw folder/6624109822729330434_63719530759489/kidney/Medulla/Cont/0004.bmp]

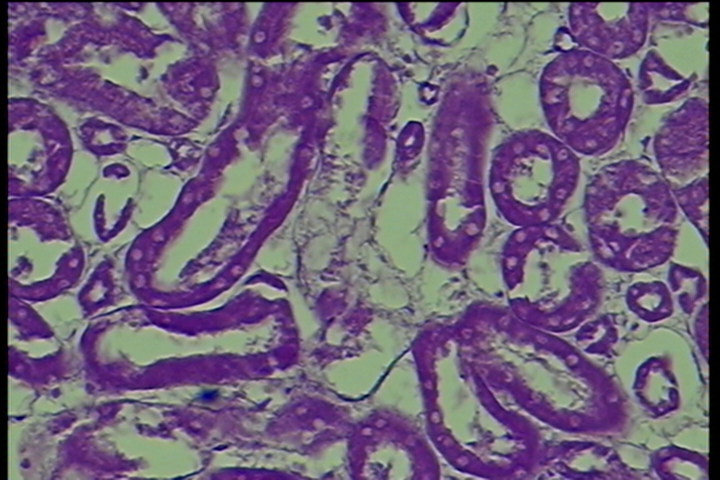

Supplement: Supplementary file 1 [file biomedicines-14-01385-s001.zip › biomedicines-4229880_Raw_Images_Figures_7-11.zipw folder/6624109822729330434_63719530759489/kidney/Medulla/Cont/0005.bmp]

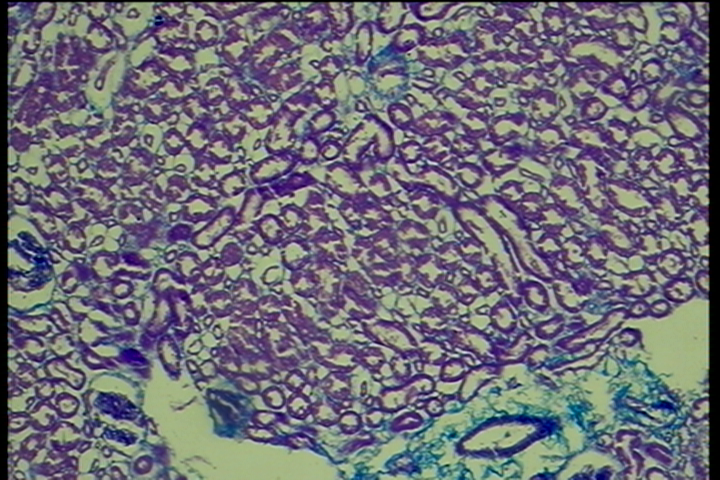

Supplement: Supplementary file 1 [file biomedicines-14-01385-s001.zip › biomedicines-4229880_Raw_Images_Figures_7-11.zipw folder/6624109822729330434_63719530759489/kidney/Medulla/Cont/0006.bmp]

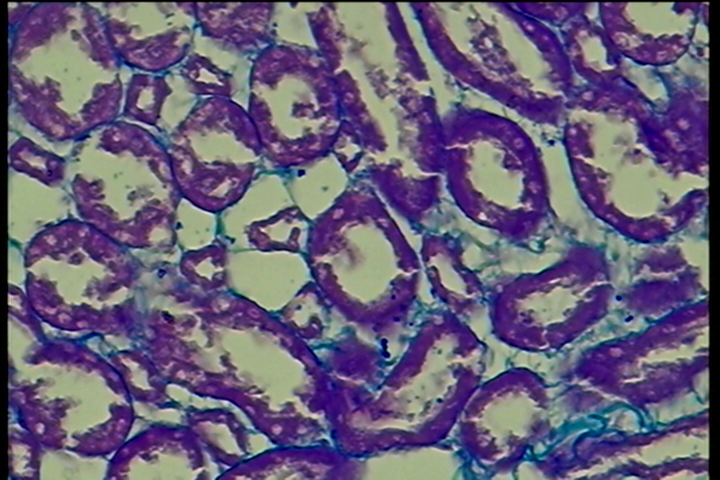

Supplement: Supplementary file 1 [file biomedicines-14-01385-s001.zip › biomedicines-4229880_Raw_Images_Figures_7-11.zipw folder/6624109822729330434_63719530759489/kidney/Medulla/Cont/0007.bmp]

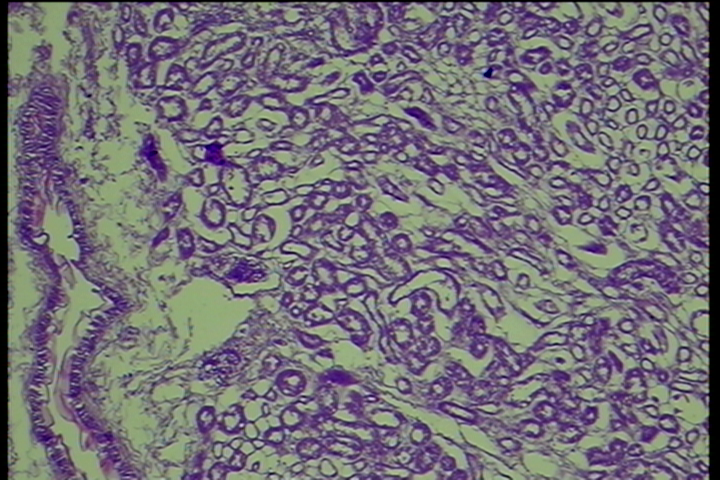

Supplement: Supplementary file 1 [file biomedicines-14-01385-s001.zip › biomedicines-4229880_Raw_Images_Figures_7-11.zipw folder/6624109822729330434_63719530759489/kidney/Medulla/Ella Genta/0000.bmp]

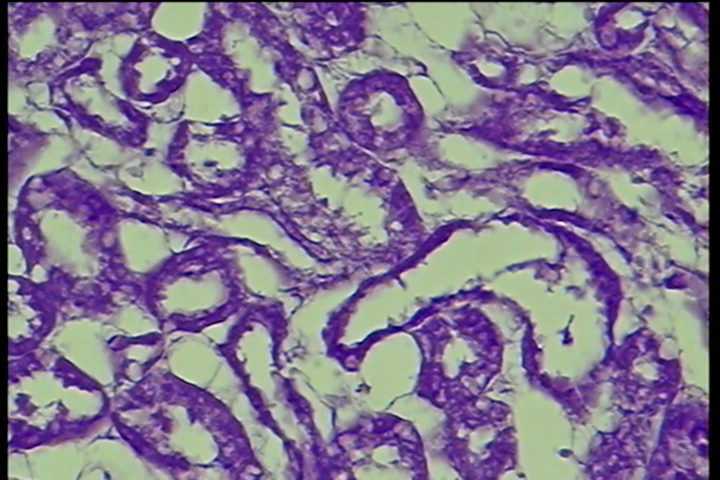

Supplement: Supplementary file 1 [file biomedicines-14-01385-s001.zip › biomedicines-4229880_Raw_Images_Figures_7-11.zipw folder/6624109822729330434_63719530759489/kidney/Medulla/Ella Genta/0001.bmp]

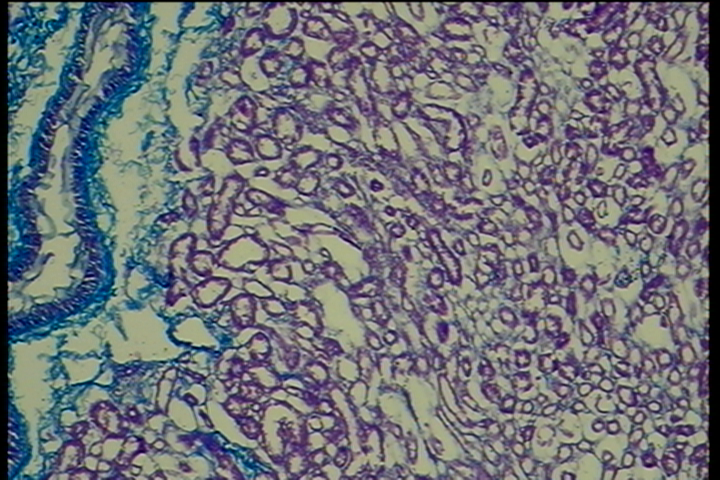

Supplement: Supplementary file 1 [file biomedicines-14-01385-s001.zip › biomedicines-4229880_Raw_Images_Figures_7-11.zipw folder/6624109822729330434_63719530759489/kidney/Medulla/Ella Genta/0002.bmp]

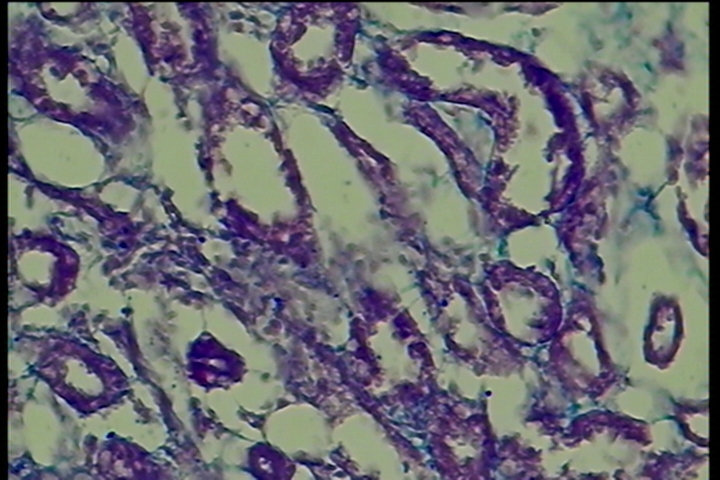

Supplement: Supplementary file 1 [file biomedicines-14-01385-s001.zip › biomedicines-4229880_Raw_Images_Figures_7-11.zipw folder/6624109822729330434_63719530759489/kidney/Medulla/Ella Genta/0003.bmp]

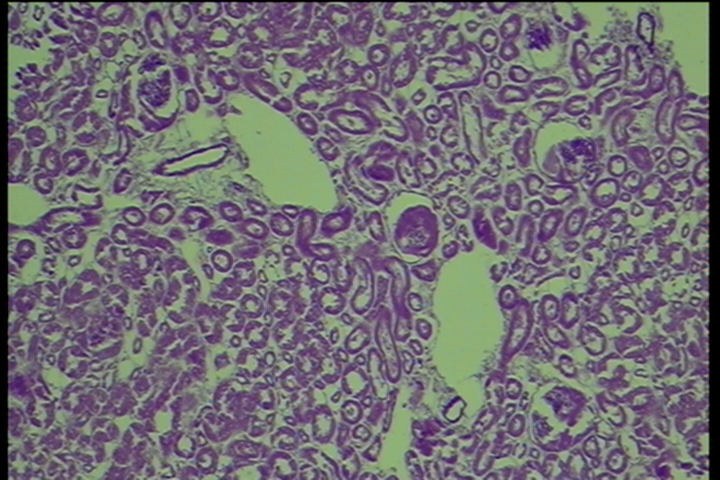

Supplement: Supplementary file 1 [file biomedicines-14-01385-s001.zip › biomedicines-4229880_Raw_Images_Figures_7-11.zipw folder/6624109822729330434_63719530759489/kidney/Medulla/Ella/0011.bmp]

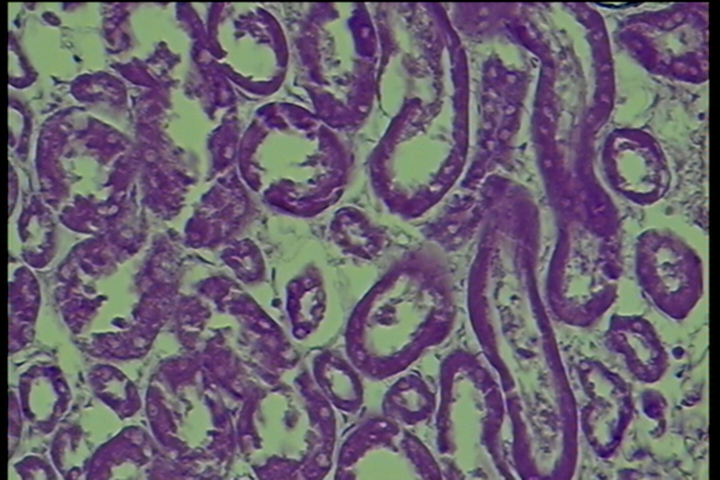

Supplement: Supplementary file 1 [file biomedicines-14-01385-s001.zip › biomedicines-4229880_Raw_Images_Figures_7-11.zipw folder/6624109822729330434_63719530759489/kidney/Medulla/Ella/0012.bmp]

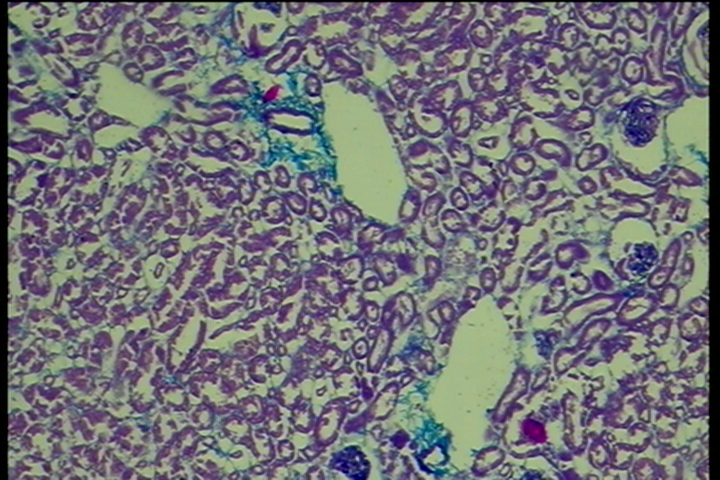

Supplement: Supplementary file 1 [file biomedicines-14-01385-s001.zip › biomedicines-4229880_Raw_Images_Figures_7-11.zipw folder/6624109822729330434_63719530759489/kidney/Medulla/Ella/0013.bmp]

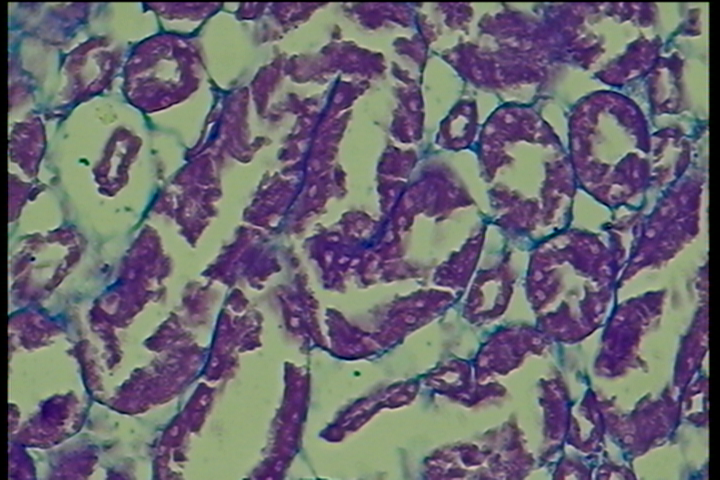

Supplement: Supplementary file 1 [file biomedicines-14-01385-s001.zip › biomedicines-4229880_Raw_Images_Figures_7-11.zipw folder/6624109822729330434_63719530759489/kidney/Medulla/Ella/0014.bmp]

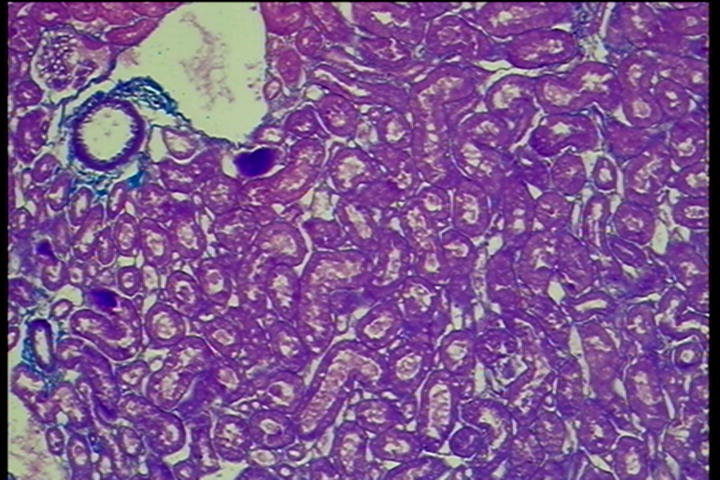

Supplement: Supplementary file 1 [file biomedicines-14-01385-s001.zip › biomedicines-4229880_Raw_Images_Figures_7-11.zipw folder/6624109822729330434_63719530759489/kidney/Medulla/Genta/0012.bmp]

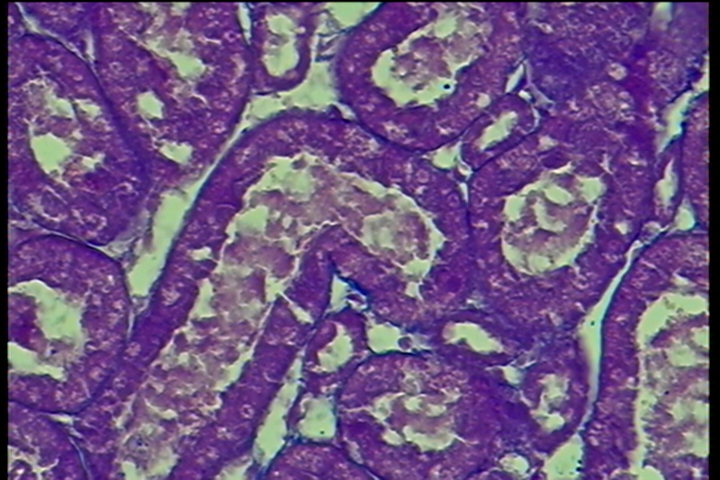

Supplement: Supplementary file 1 [file biomedicines-14-01385-s001.zip › biomedicines-4229880_Raw_Images_Figures_7-11.zipw folder/6624109822729330434_63719530759489/kidney/Medulla/Genta/0013.bmp]

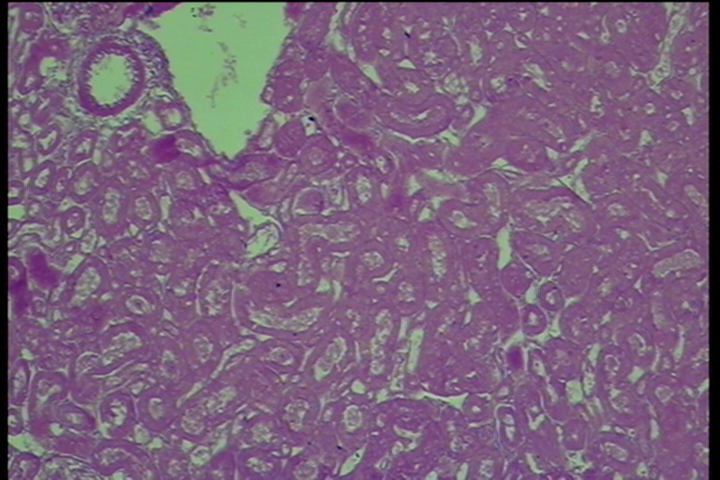

Supplement: Supplementary file 1 [file biomedicines-14-01385-s001.zip › biomedicines-4229880_Raw_Images_Figures_7-11.zipw folder/6624109822729330434_63719530759489/kidney/Medulla/Genta/0014.bmp]

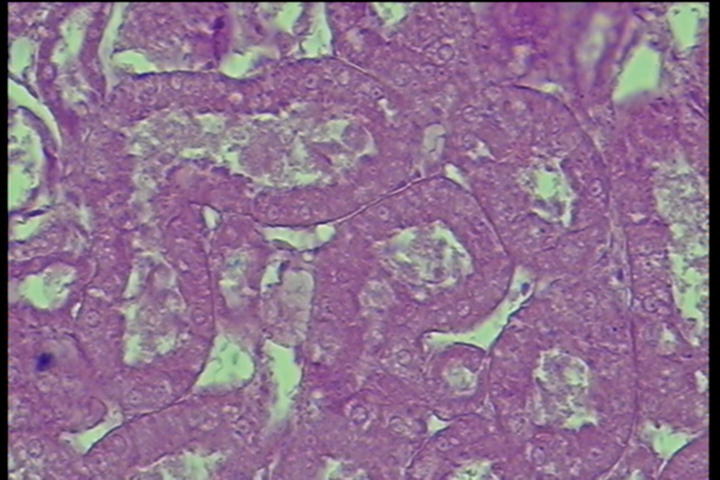

Supplement: Supplementary file 1 [file biomedicines-14-01385-s001.zip › biomedicines-4229880_Raw_Images_Figures_7-11.zipw folder/6624109822729330434_63719530759489/kidney/Medulla/Genta/0016.bmp]

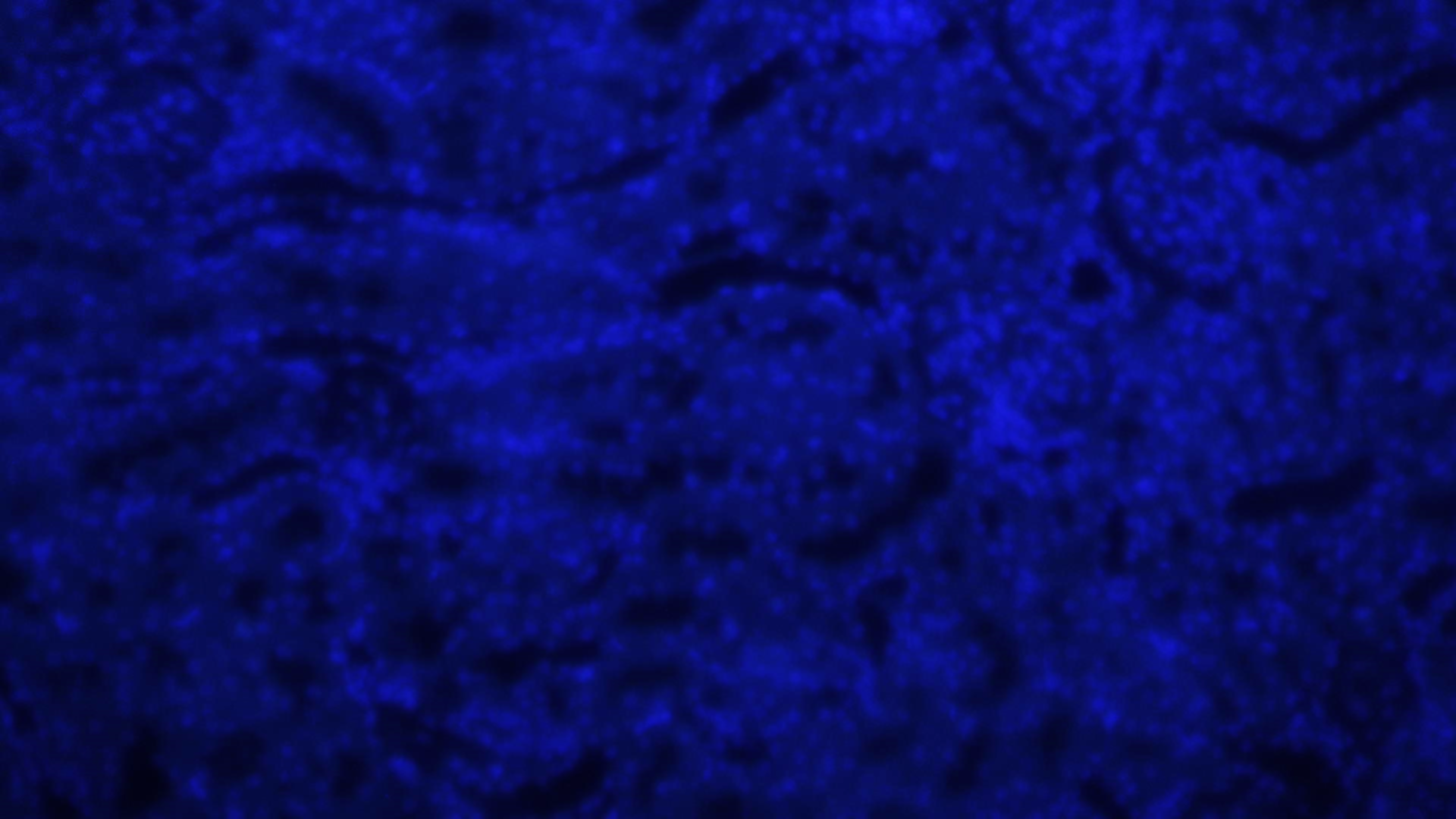

Supplement: Supplementary file 1 [file biomedicines-14-01385-s001.zip › biomedicines-4229880_Raw_Images_Figures_7-11.zipw folder/Original microscopy imgesRaw immunofluorescence results of Figures 7, 8, and 9 of the article/KIM1/Control/1/1.tif]

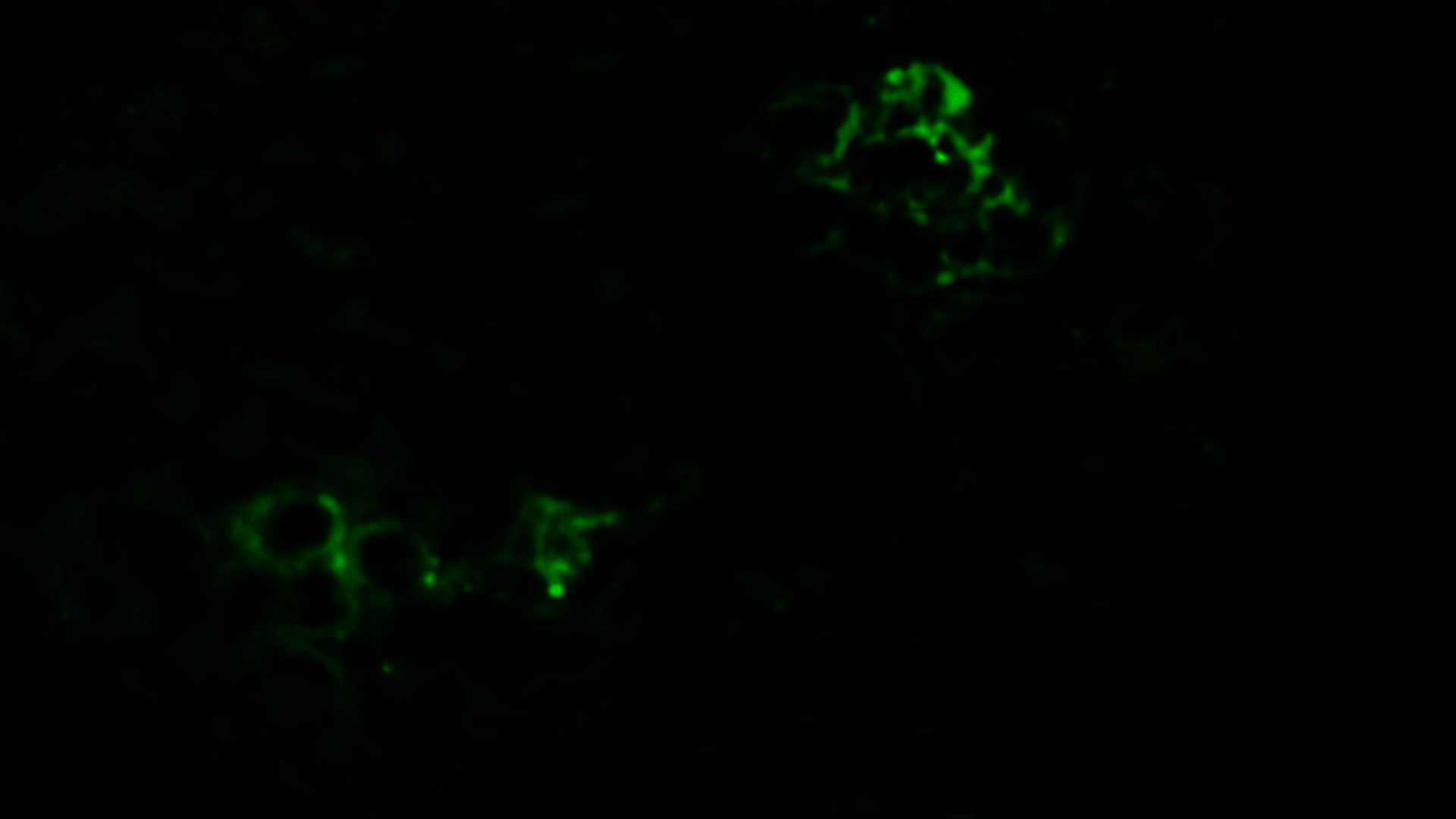

Supplement: Supplementary file 1 [file biomedicines-14-01385-s001.zip › biomedicines-4229880_Raw_Images_Figures_7-11.zipw folder/Original microscopy imgesRaw immunofluorescence results of Figures 7, 8, and 9 of the article/KIM1/Control/1/2.tif]

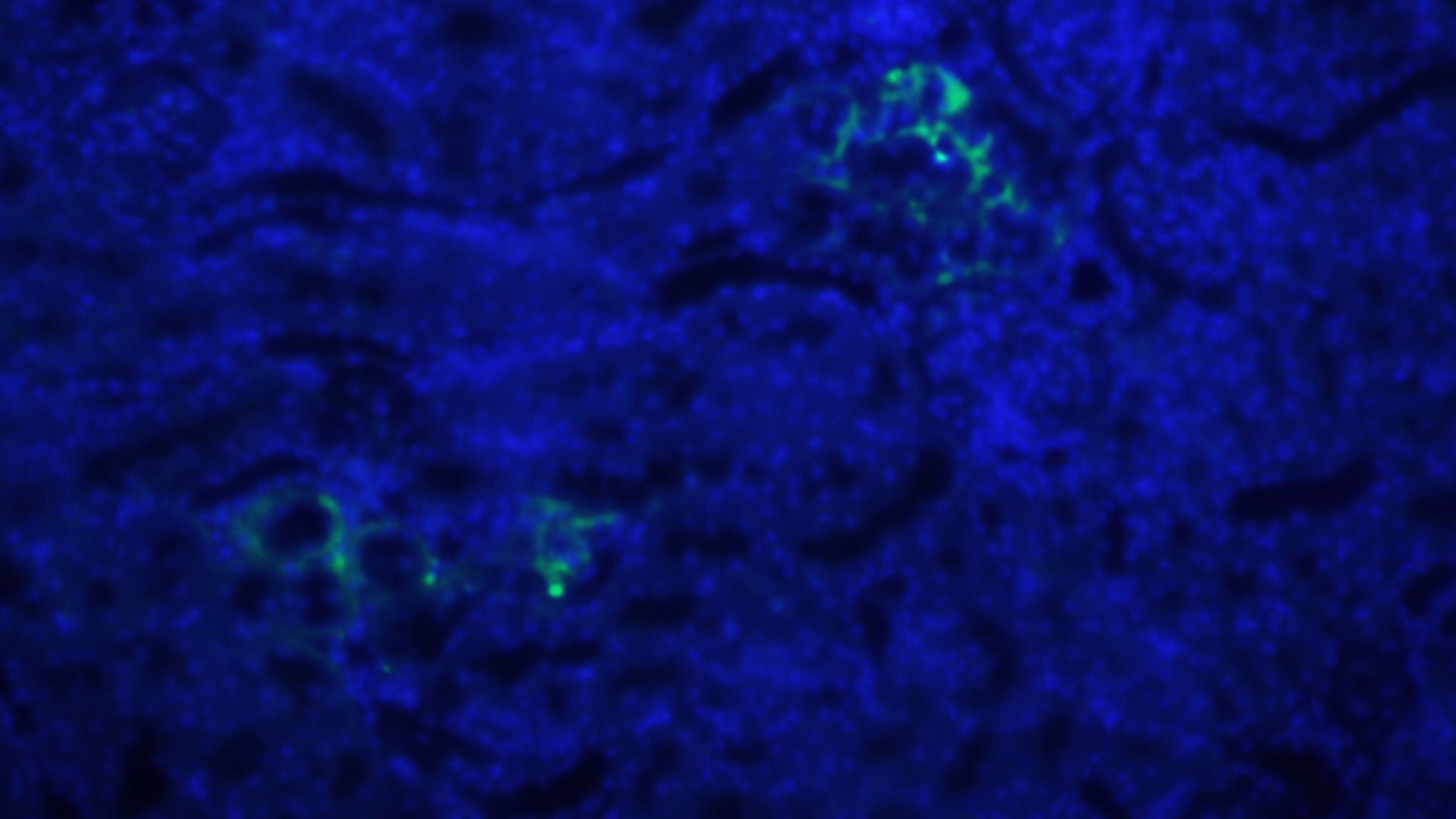

Supplement: Supplementary file 1 [file biomedicines-14-01385-s001.zip › biomedicines-4229880_Raw_Images_Figures_7-11.zipw folder/Original microscopy imgesRaw immunofluorescence results of Figures 7, 8, and 9 of the article/KIM1/Control/1/3.tif]

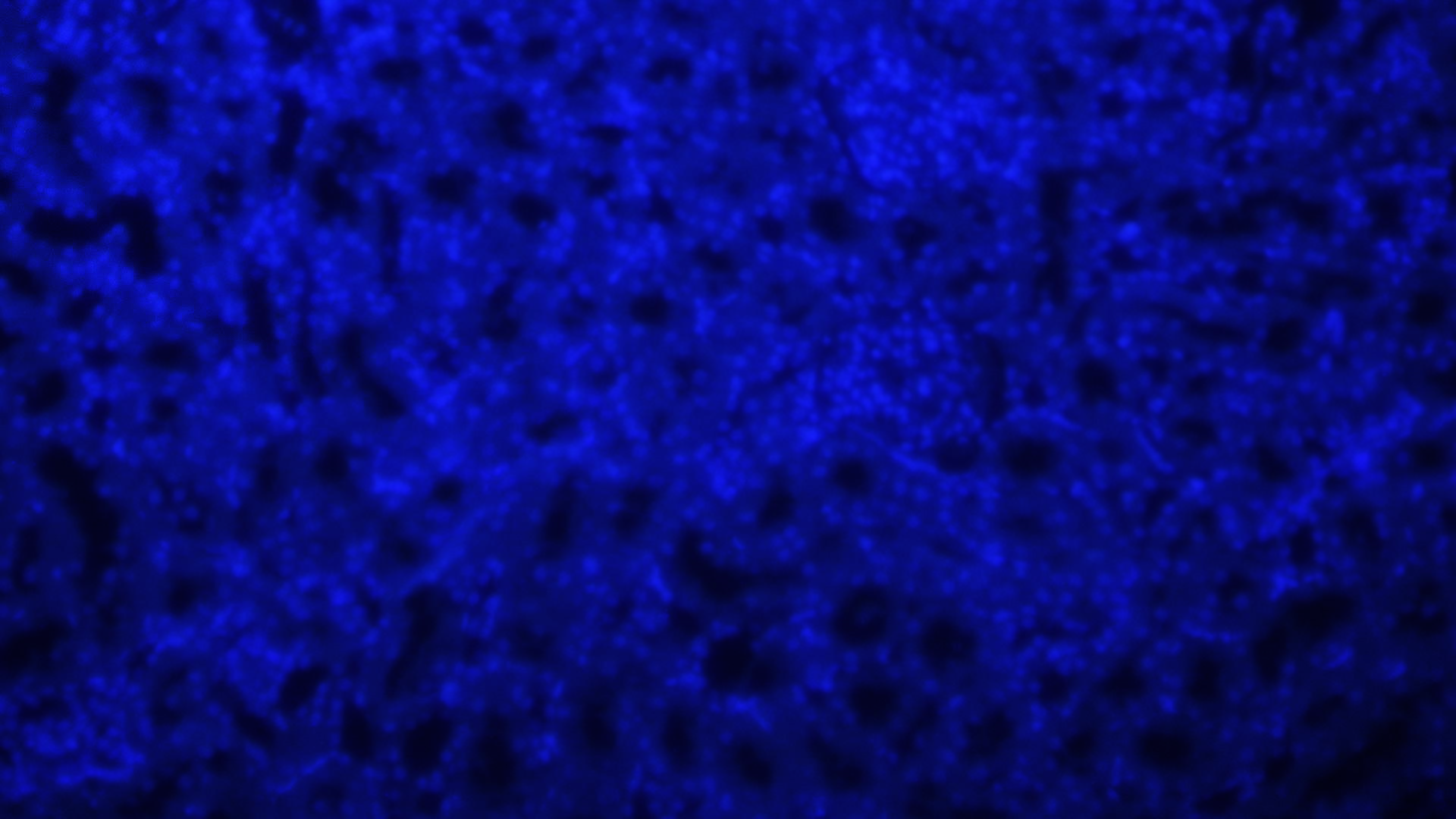

Supplement: Supplementary file 1 [file biomedicines-14-01385-s001.zip › biomedicines-4229880_Raw_Images_Figures_7-11.zipw folder/Original microscopy imgesRaw immunofluorescence results of Figures 7, 8, and 9 of the article/KIM1/Control/2/1.tif]

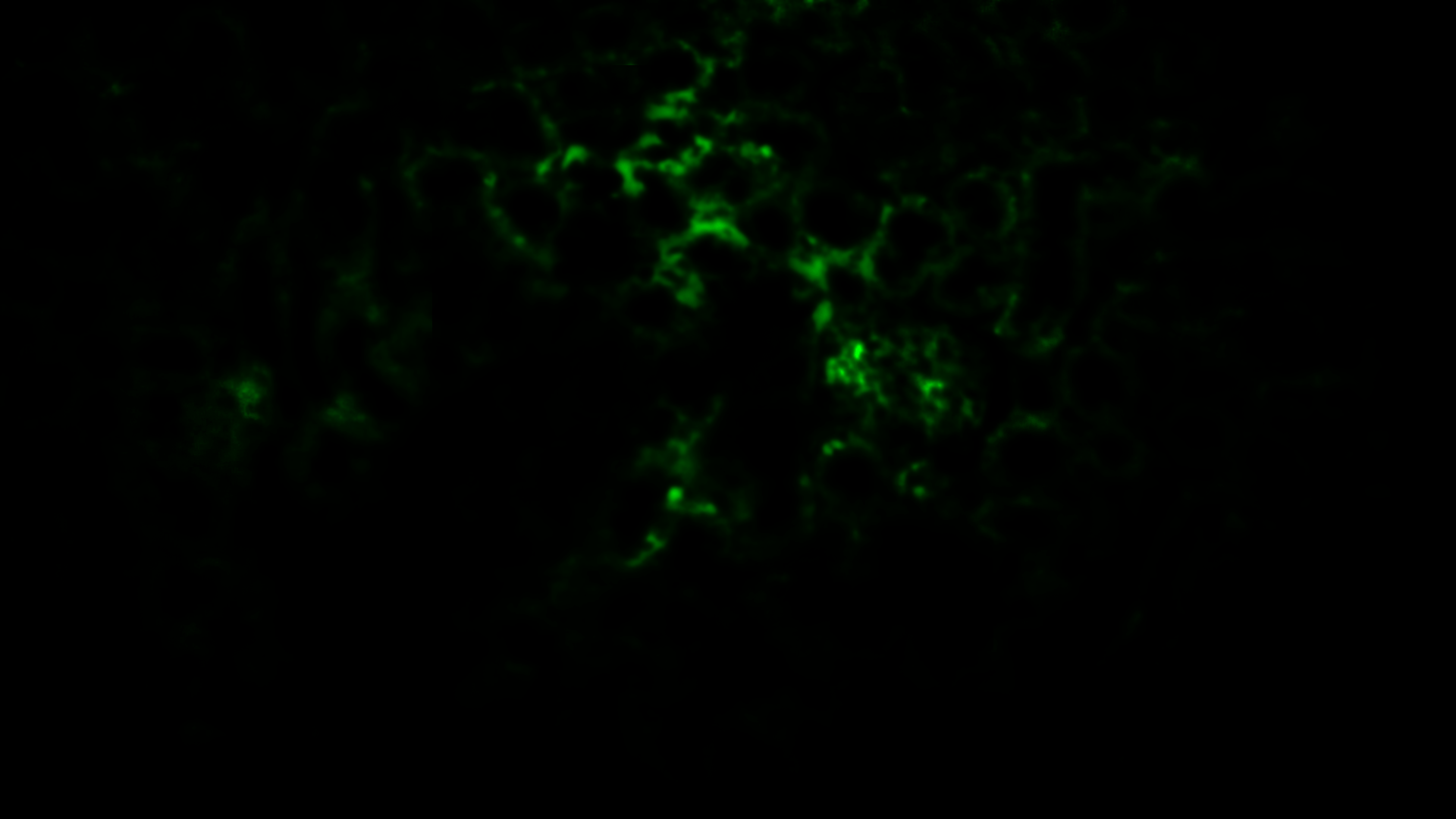

Supplement: Supplementary file 1 [file biomedicines-14-01385-s001.zip › biomedicines-4229880_Raw_Images_Figures_7-11.zipw folder/Original microscopy imgesRaw immunofluorescence results of Figures 7, 8, and 9 of the article/KIM1/Control/2/2.tif]

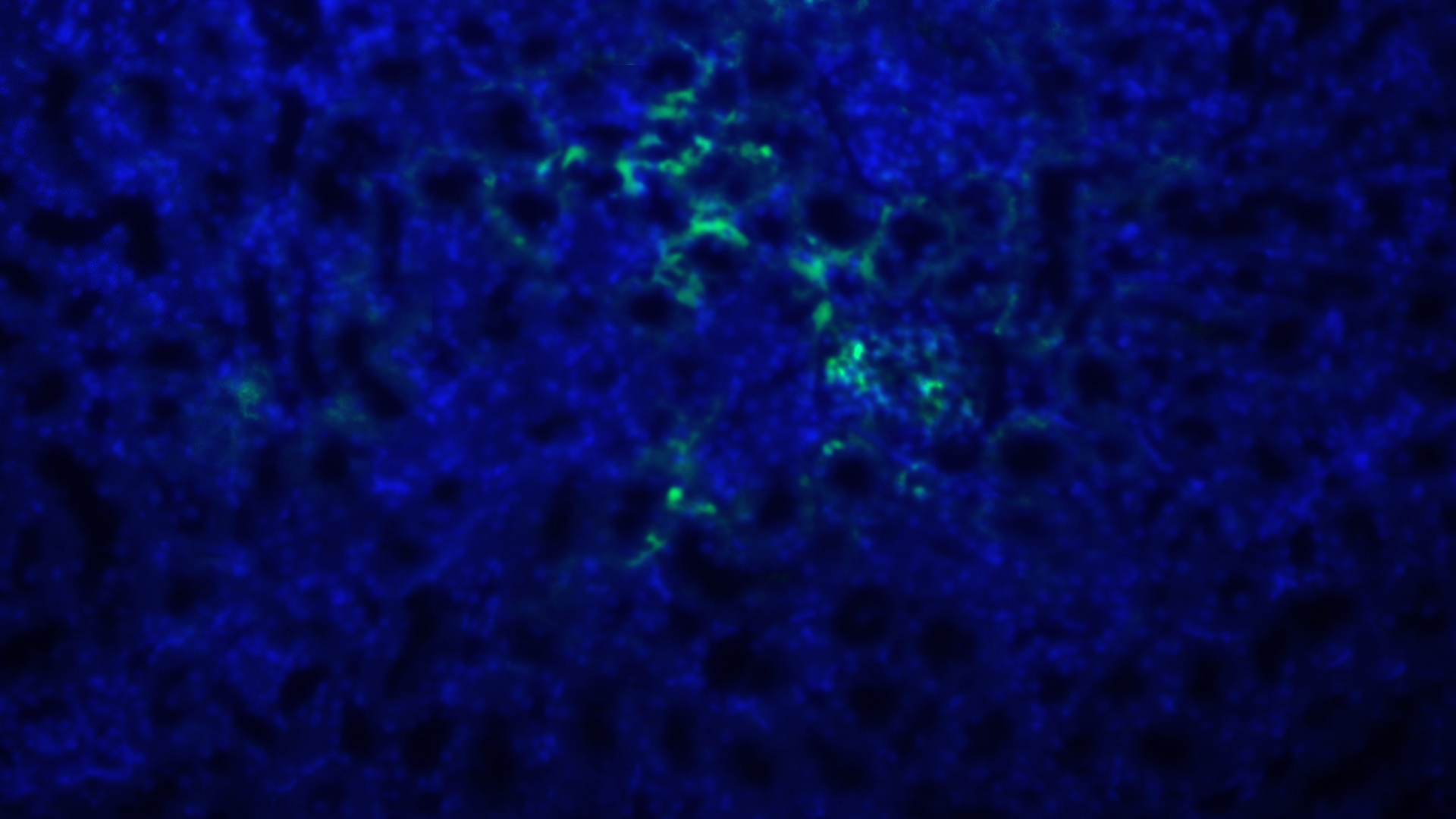

Supplement: Supplementary file 1 [file biomedicines-14-01385-s001.zip › biomedicines-4229880_Raw_Images_Figures_7-11.zipw folder/Original microscopy imgesRaw immunofluorescence results of Figures 7, 8, and 9 of the article/KIM1/Control/2/3.tif]

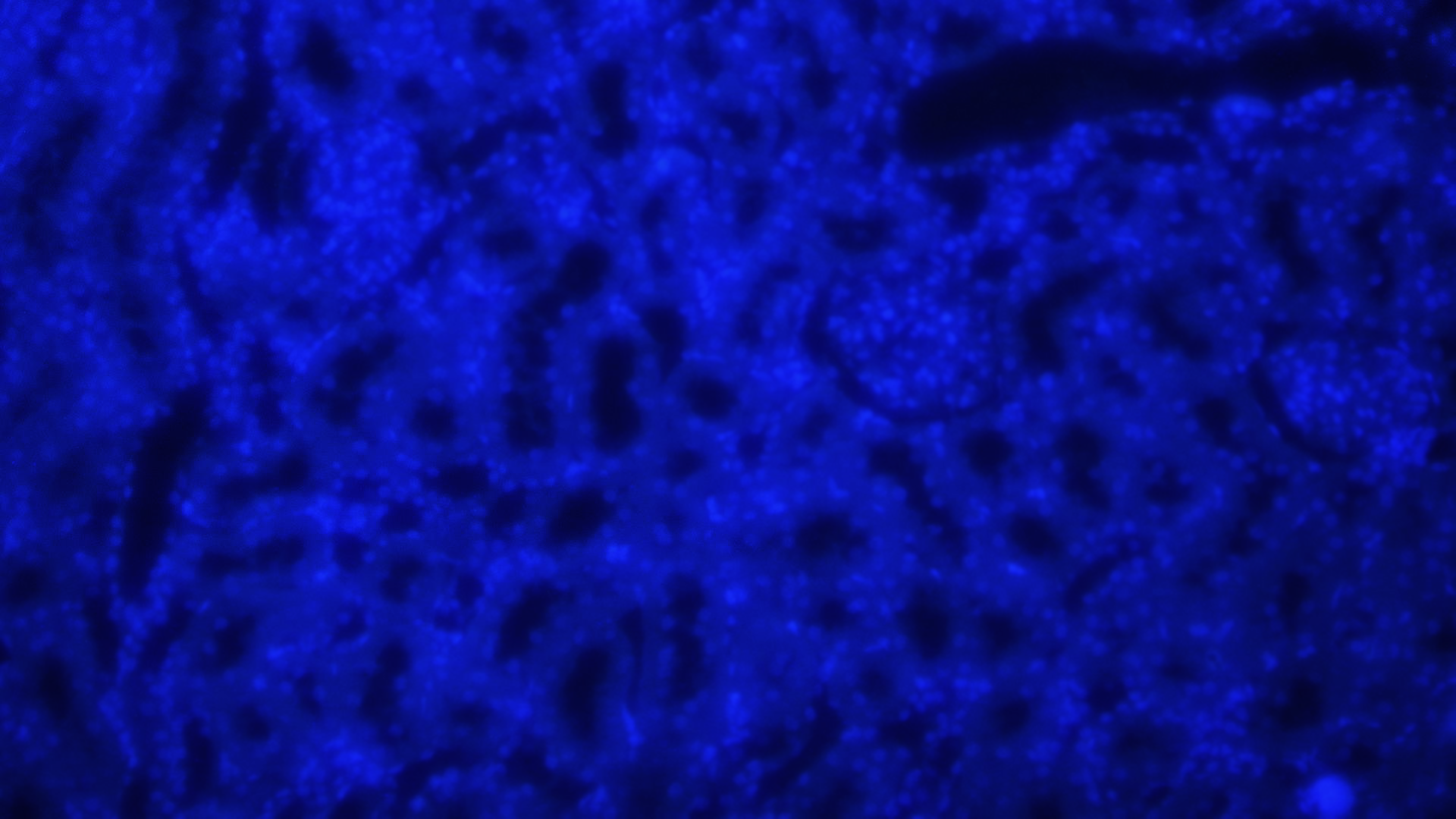

Supplement: Supplementary file 1 [file biomedicines-14-01385-s001.zip › biomedicines-4229880_Raw_Images_Figures_7-11.zipw folder/Original microscopy imgesRaw immunofluorescence results of Figures 7, 8, and 9 of the article/KIM1/Control/3/1.tif]

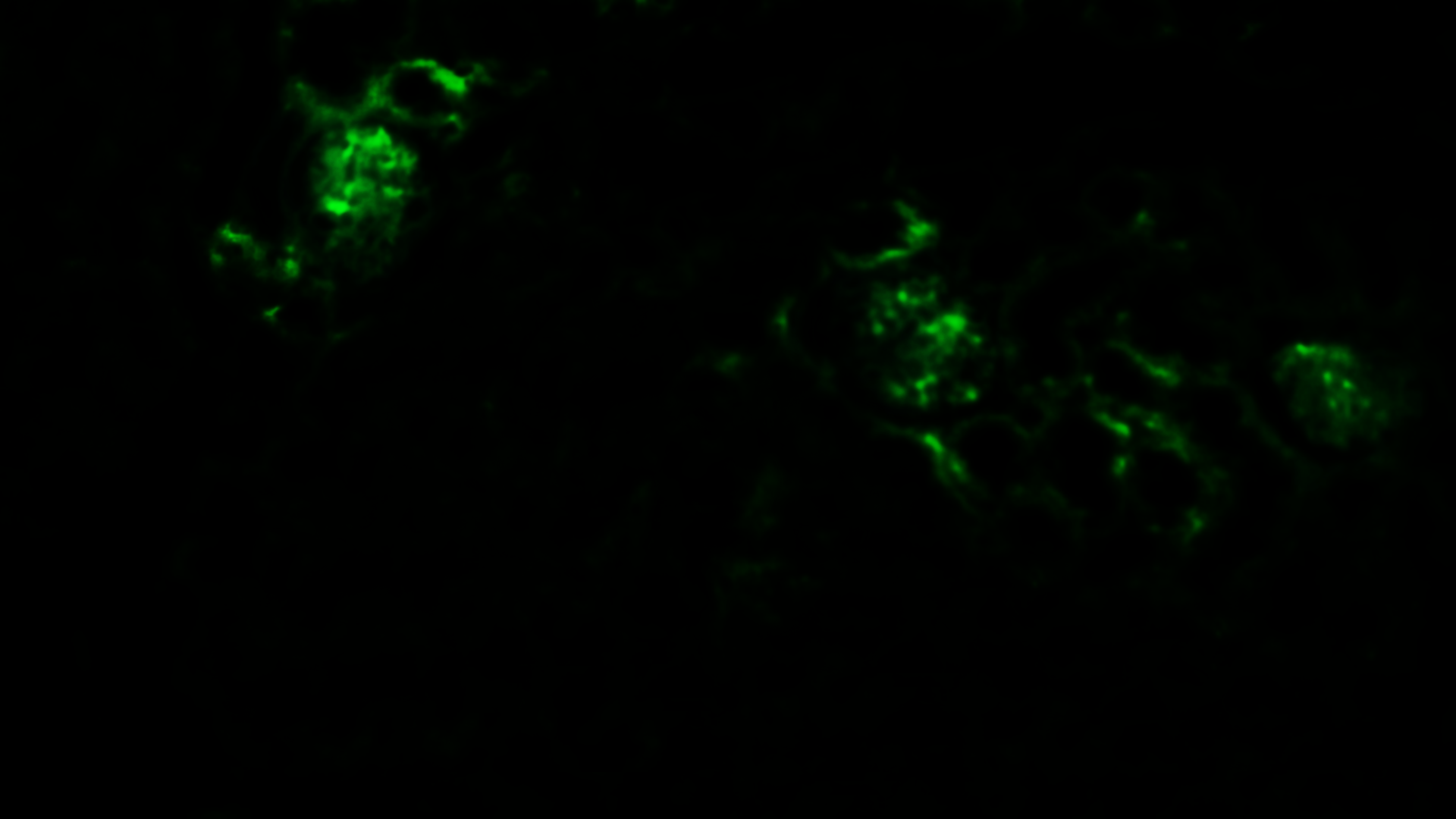

Supplement: Supplementary file 1 [file biomedicines-14-01385-s001.zip › biomedicines-4229880_Raw_Images_Figures_7-11.zipw folder/Original microscopy imgesRaw immunofluorescence results of Figures 7, 8, and 9 of the article/KIM1/Control/3/2.tif]

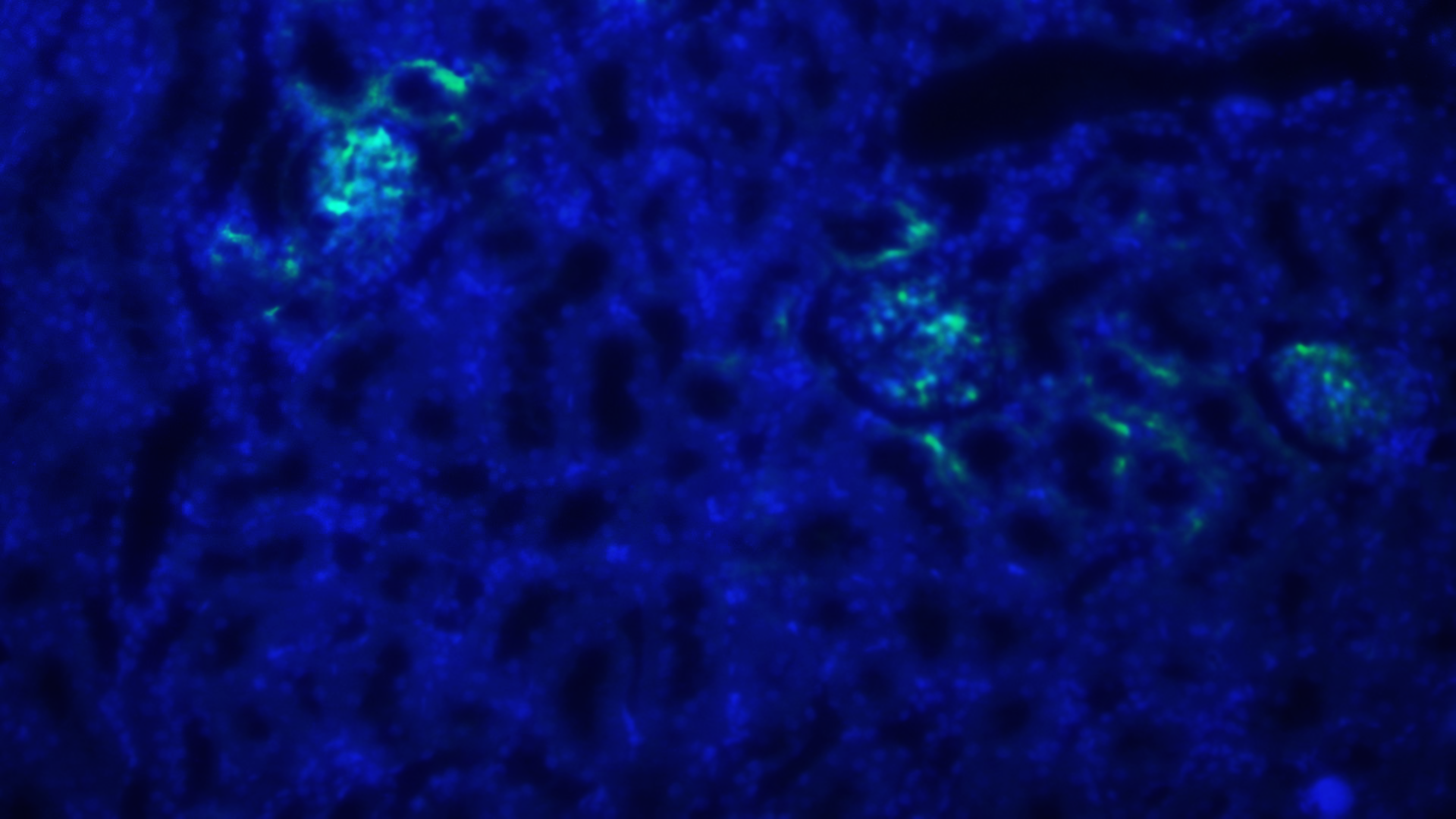

Supplement: Supplementary file 1 [file biomedicines-14-01385-s001.zip › biomedicines-4229880_Raw_Images_Figures_7-11.zipw folder/Original microscopy imgesRaw immunofluorescence results of Figures 7, 8, and 9 of the article/KIM1/Control/3/3.tif]

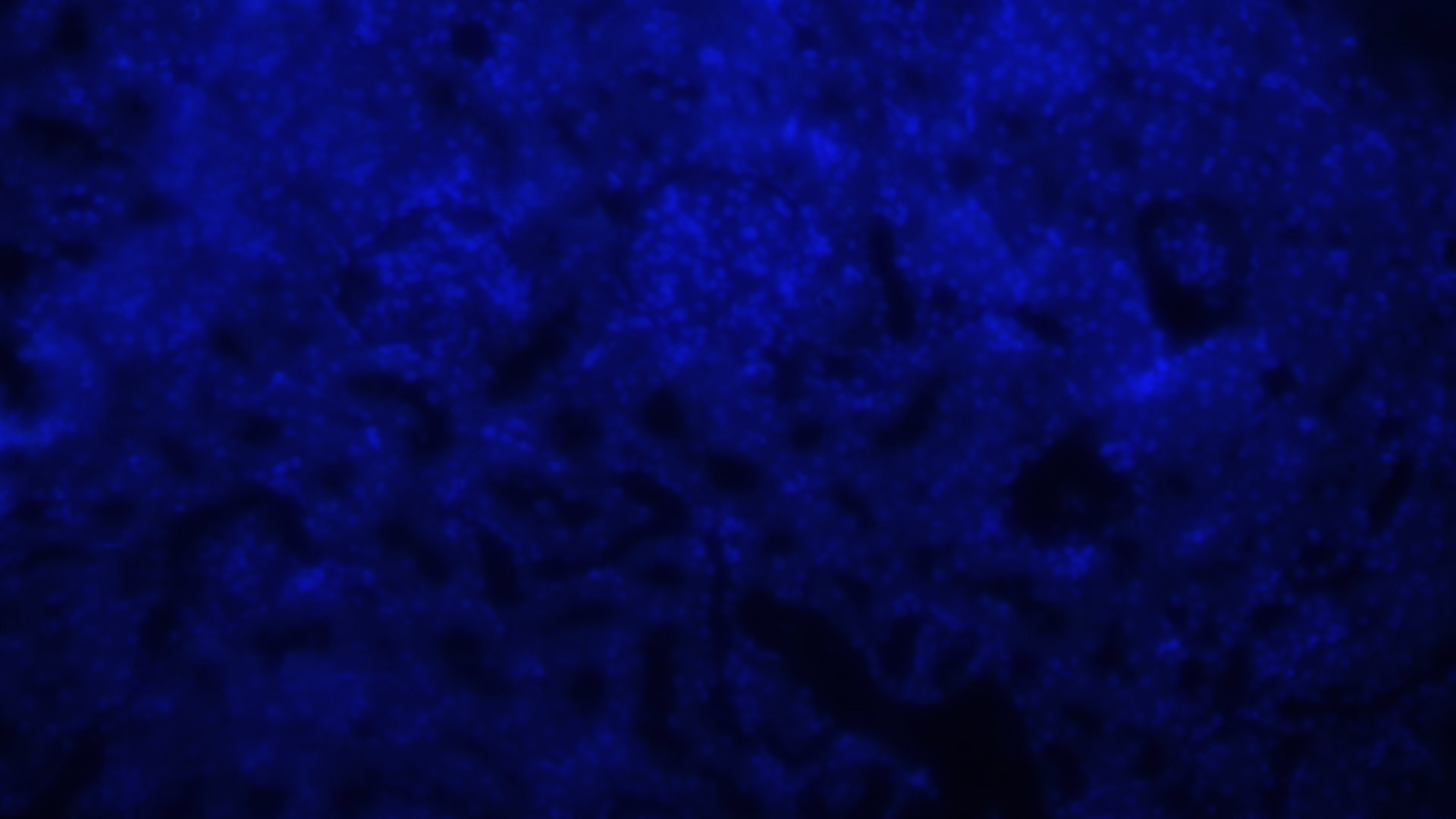

Supplement: Supplementary file 1 [file biomedicines-14-01385-s001.zip › biomedicines-4229880_Raw_Images_Figures_7-11.zipw folder/Original microscopy imgesRaw immunofluorescence results of Figures 7, 8, and 9 of the article/KIM1/Ellagic acid/1/1.tif]

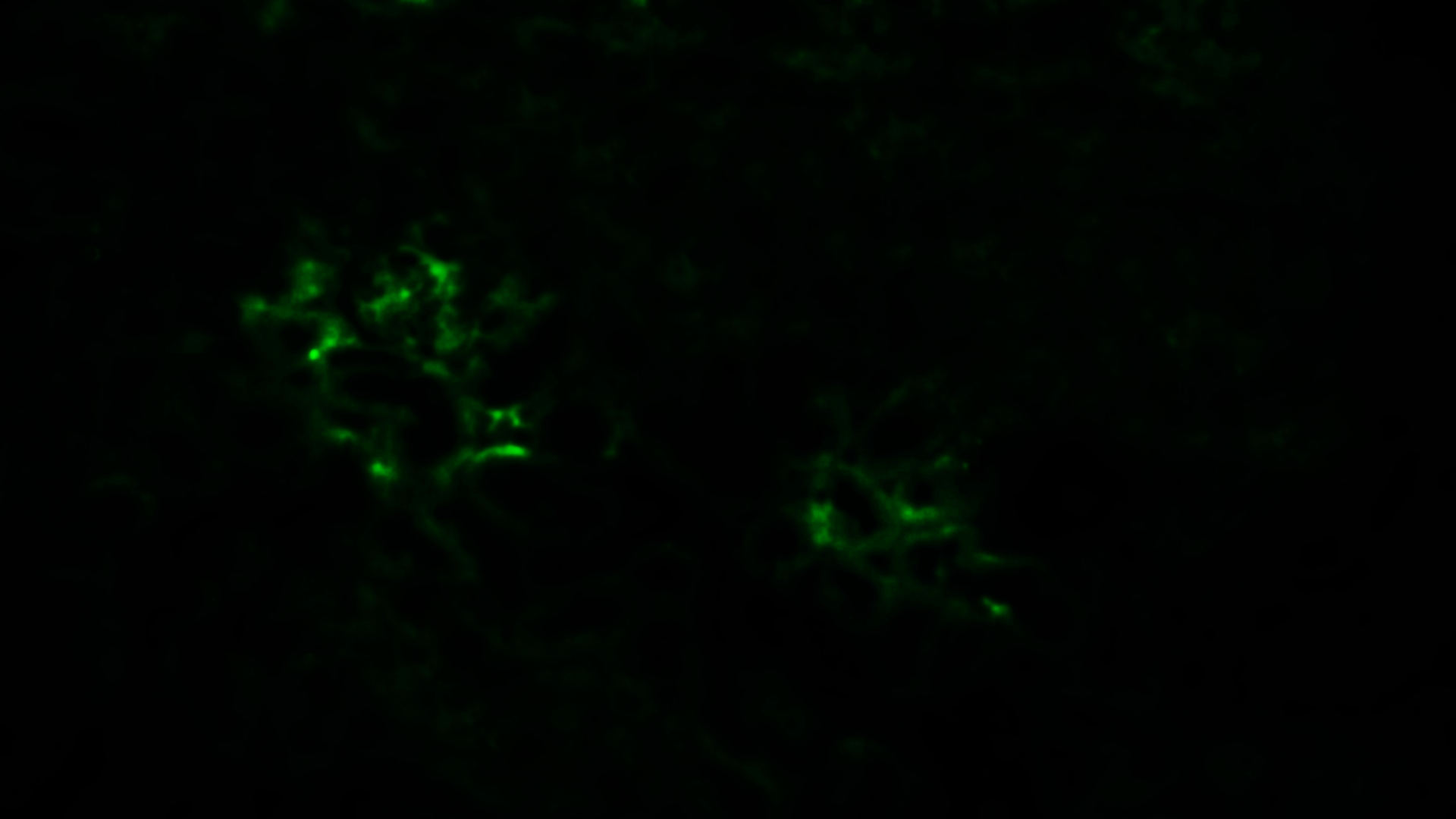

Supplement: Supplementary file 1 [file biomedicines-14-01385-s001.zip › biomedicines-4229880_Raw_Images_Figures_7-11.zipw folder/Original microscopy imgesRaw immunofluorescence results of Figures 7, 8, and 9 of the article/KIM1/Ellagic acid/1/2.tif]

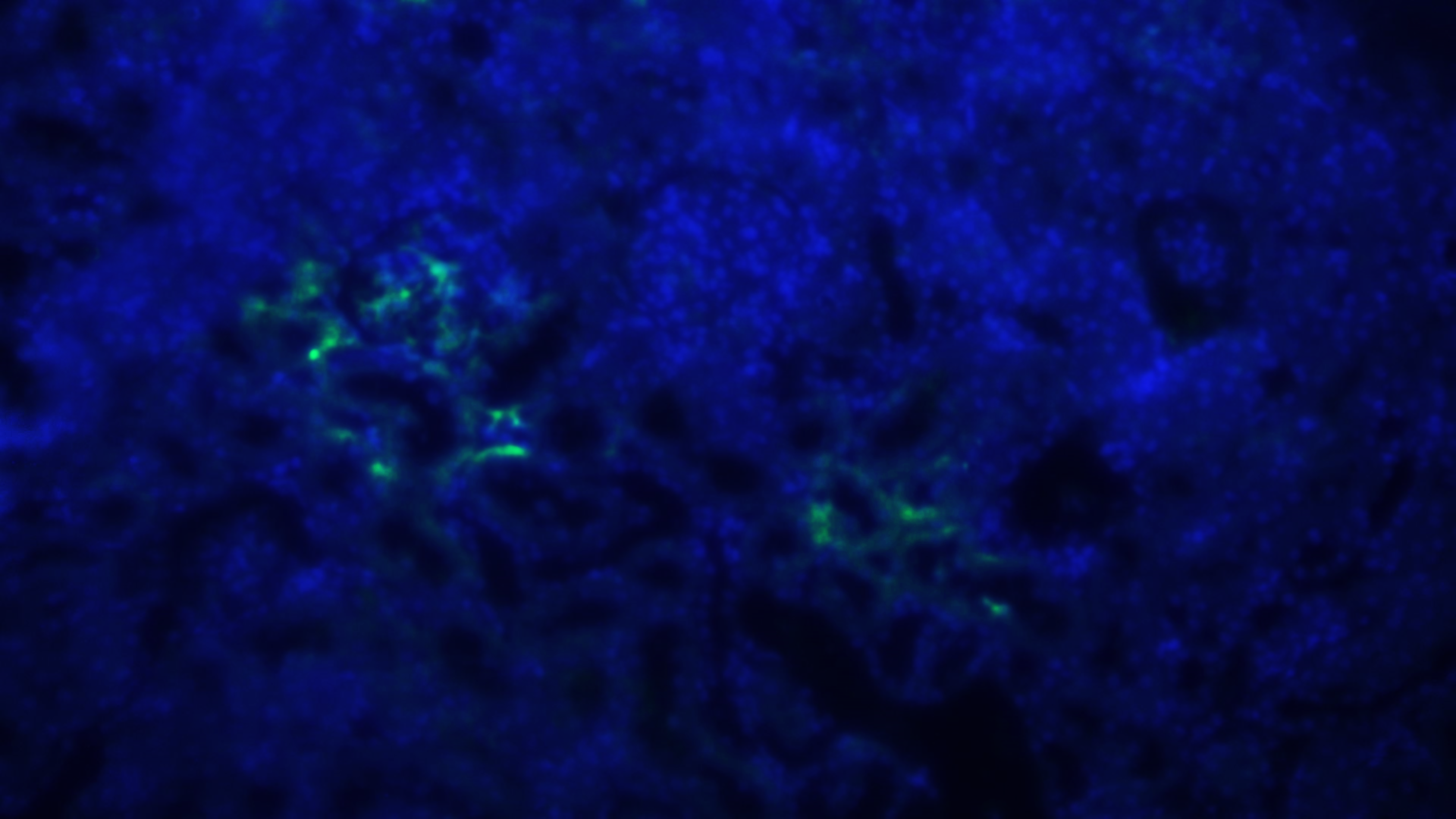

Supplement: Supplementary file 1 [file biomedicines-14-01385-s001.zip › biomedicines-4229880_Raw_Images_Figures_7-11.zipw folder/Original microscopy imgesRaw immunofluorescence results of Figures 7, 8, and 9 of the article/KIM1/Ellagic acid/1/3.tif]

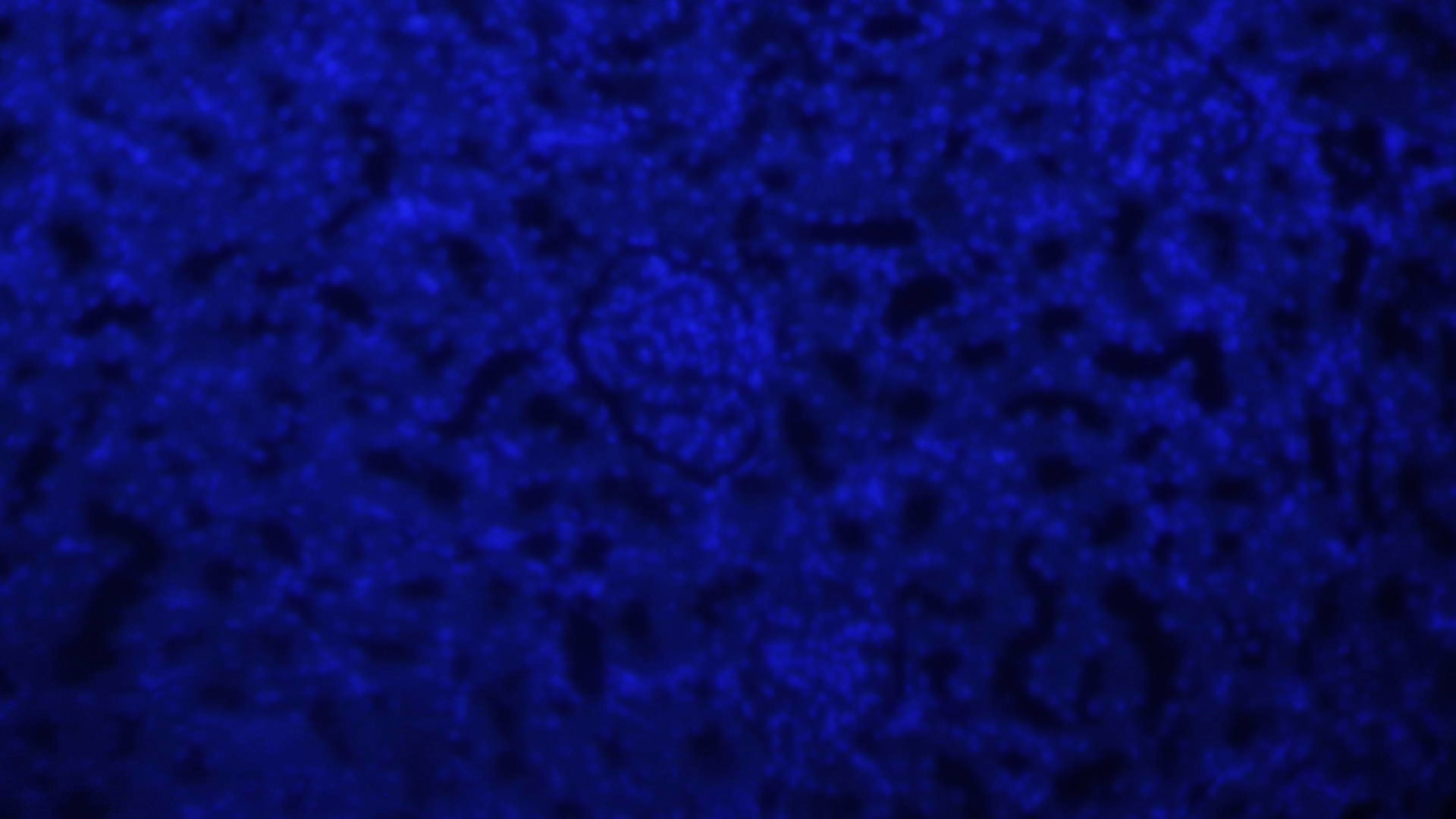

Supplement: Supplementary file 1 [file biomedicines-14-01385-s001.zip › biomedicines-4229880_Raw_Images_Figures_7-11.zipw folder/Original microscopy imgesRaw immunofluorescence results of Figures 7, 8, and 9 of the article/KIM1/Ellagic acid/2/1.tif]

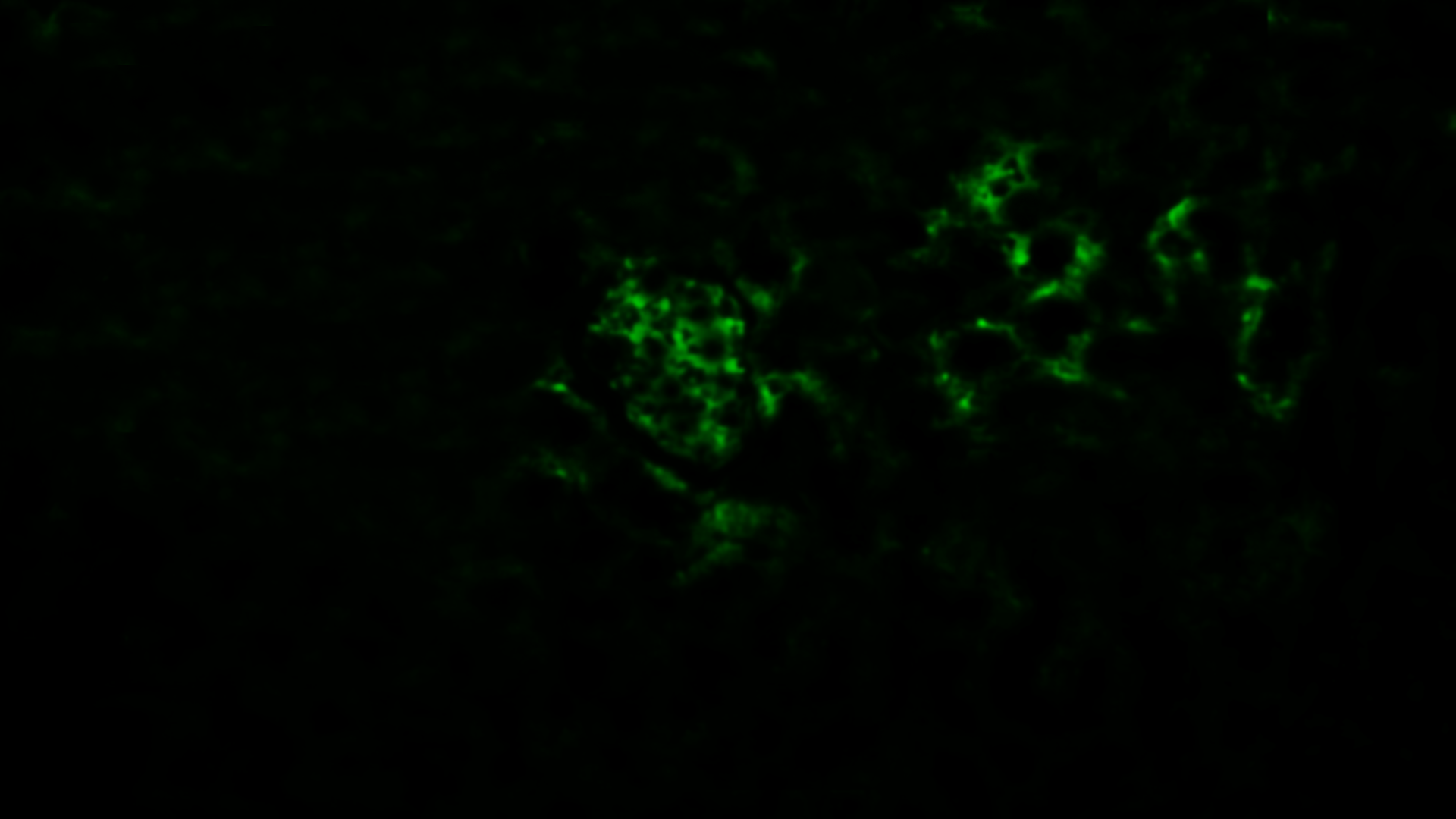

Supplement: Supplementary file 1 [file biomedicines-14-01385-s001.zip › biomedicines-4229880_Raw_Images_Figures_7-11.zipw folder/Original microscopy imgesRaw immunofluorescence results of Figures 7, 8, and 9 of the article/KIM1/Ellagic acid/2/2.tif]

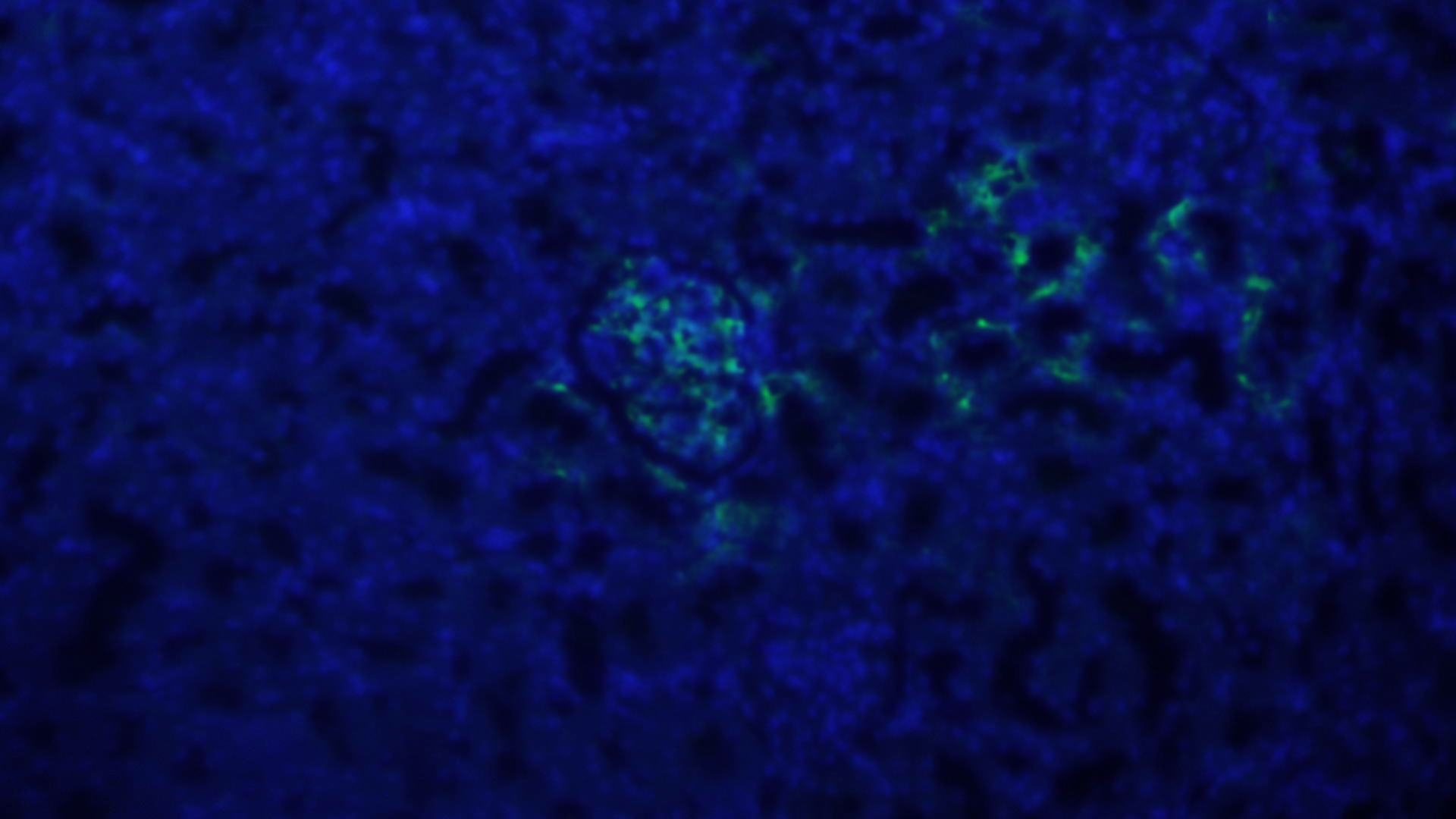

Supplement: Supplementary file 1 [file biomedicines-14-01385-s001.zip › biomedicines-4229880_Raw_Images_Figures_7-11.zipw folder/Original microscopy imgesRaw immunofluorescence results of Figures 7, 8, and 9 of the article/KIM1/Ellagic acid/2/3.tif]

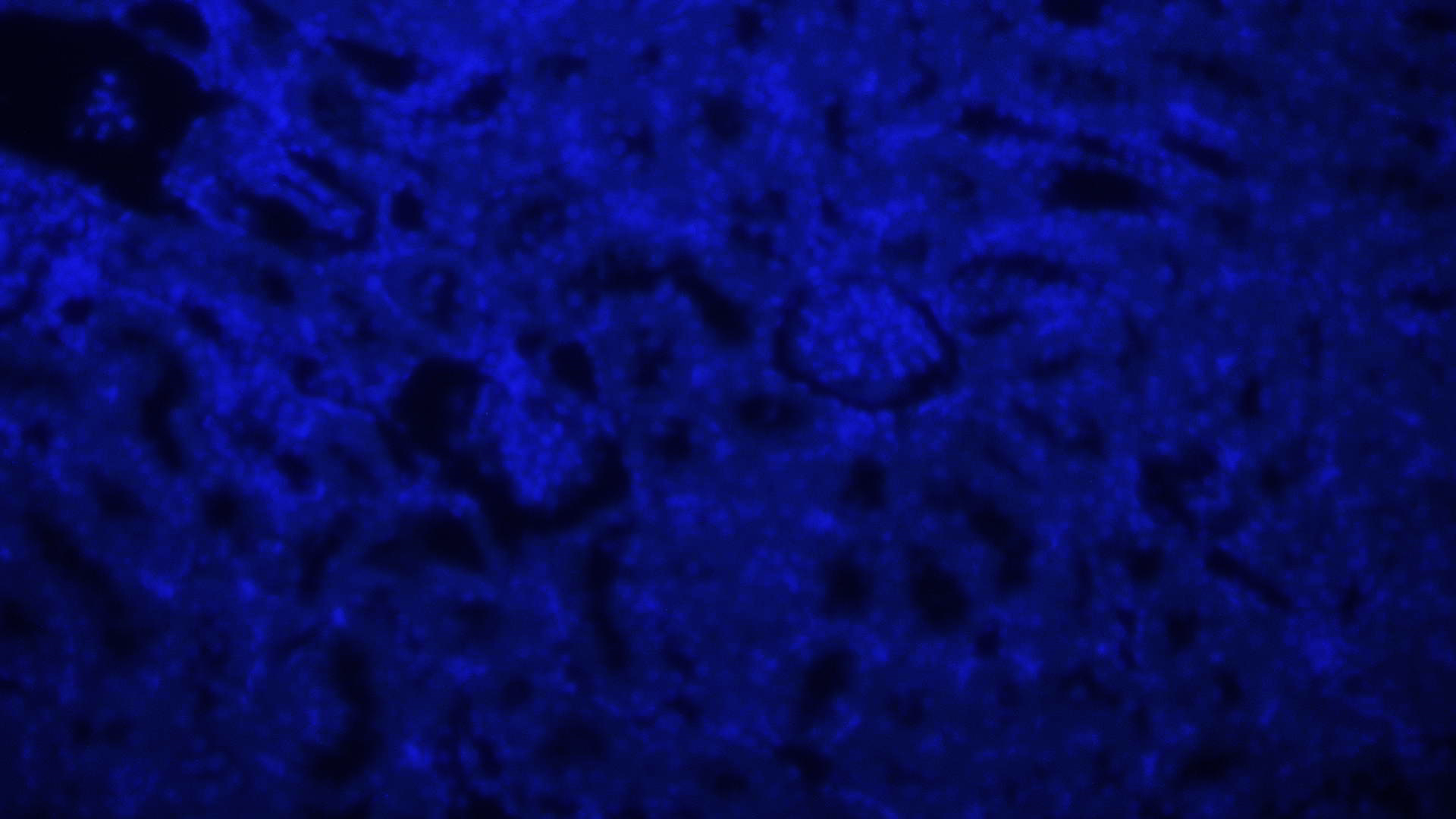

Supplement: Supplementary file 1 [file biomedicines-14-01385-s001.zip › biomedicines-4229880_Raw_Images_Figures_7-11.zipw folder/Original microscopy imgesRaw immunofluorescence results of Figures 7, 8, and 9 of the article/KIM1/Ellagic acid/3/1.tif]

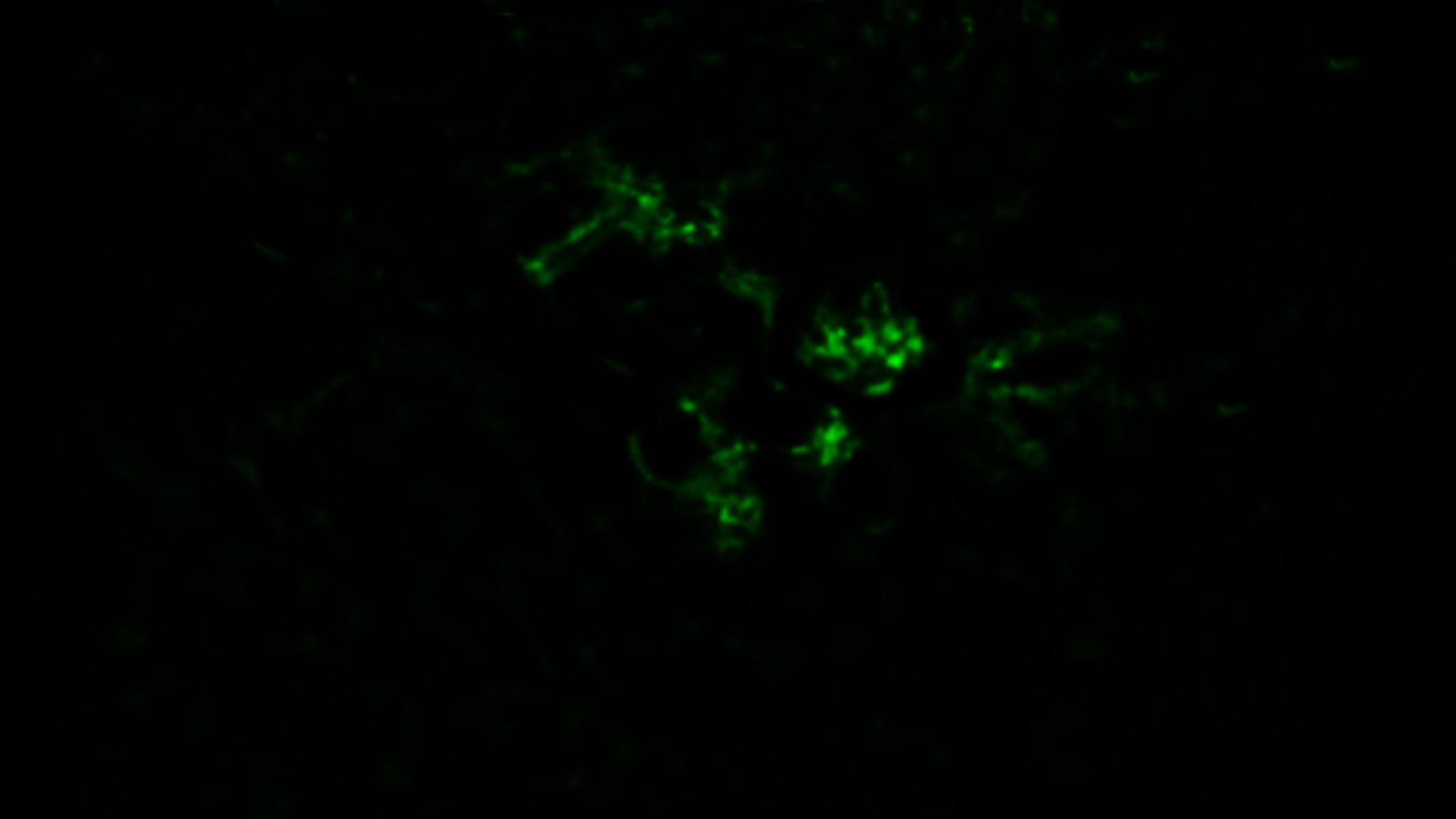

Supplement: Supplementary file 1 [file biomedicines-14-01385-s001.zip › biomedicines-4229880_Raw_Images_Figures_7-11.zipw folder/Original microscopy imgesRaw immunofluorescence results of Figures 7, 8, and 9 of the article/KIM1/Ellagic acid/3/2.tif]

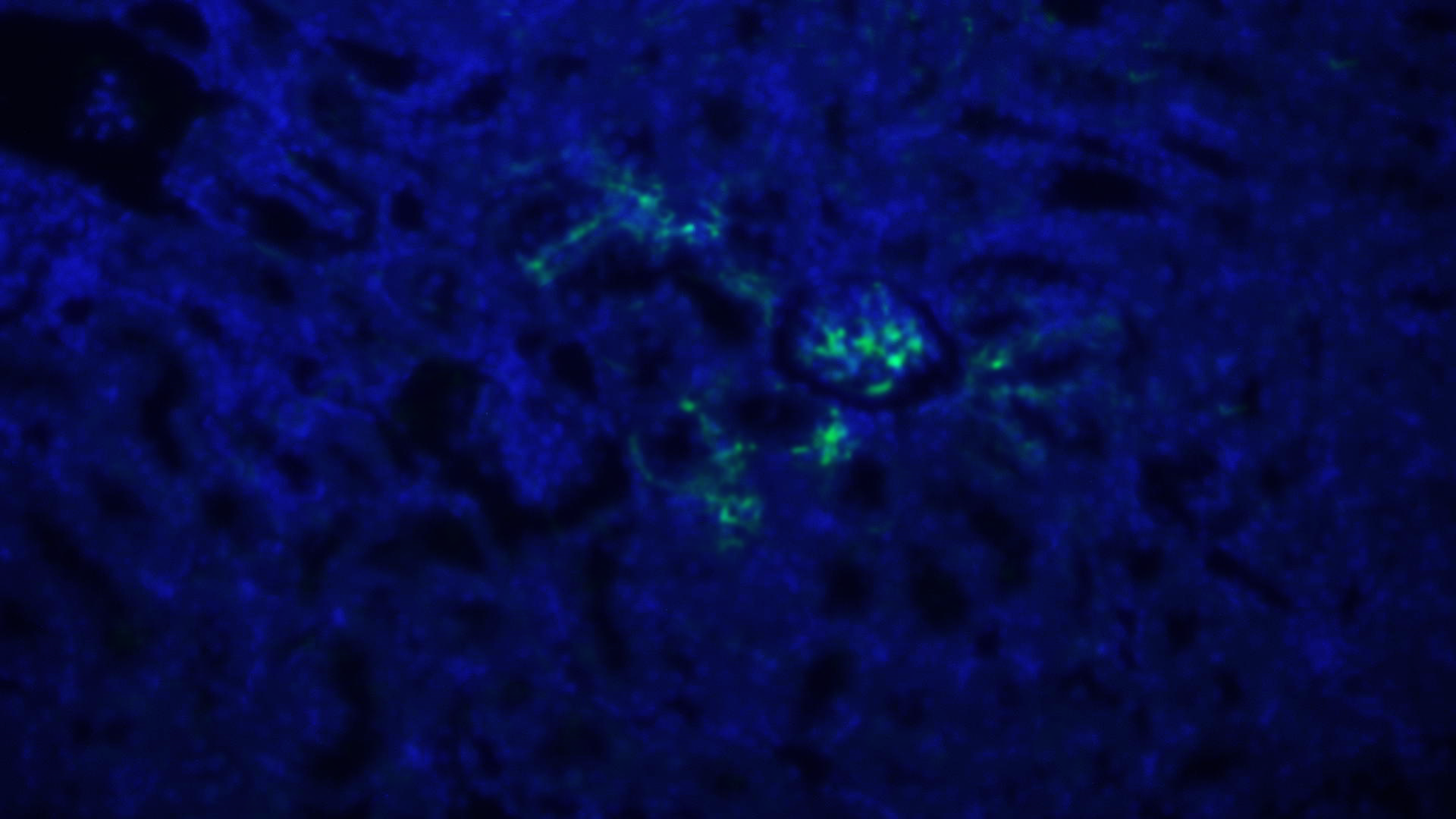

Supplement: Supplementary file 1 [file biomedicines-14-01385-s001.zip › biomedicines-4229880_Raw_Images_Figures_7-11.zipw folder/Original microscopy imgesRaw immunofluorescence results of Figures 7, 8, and 9 of the article/KIM1/Ellagic acid/3/3.tif]

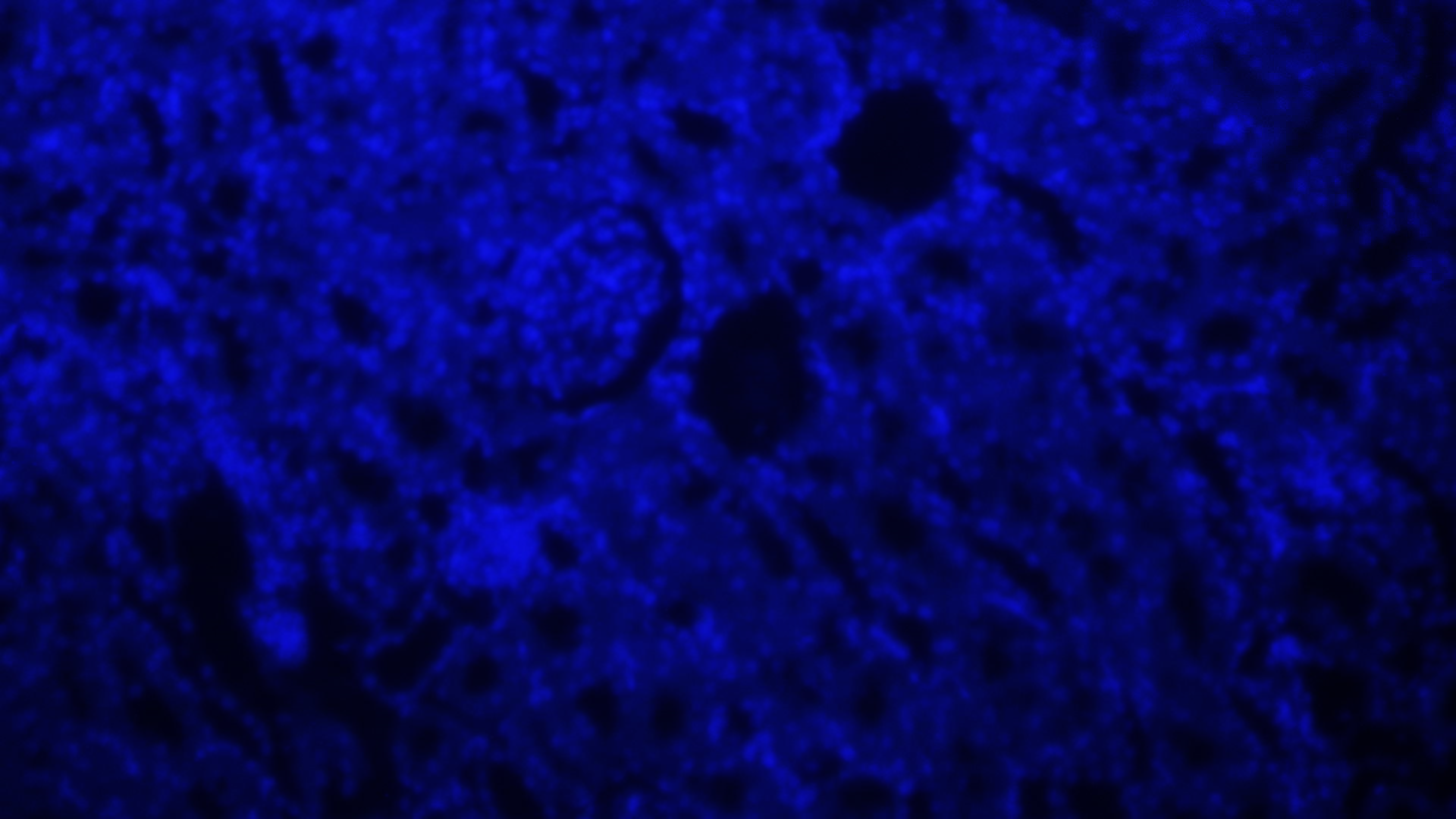

Supplement: Supplementary file 1 [file biomedicines-14-01385-s001.zip › biomedicines-4229880_Raw_Images_Figures_7-11.zipw folder/Original microscopy imgesRaw immunofluorescence results of Figures 7, 8, and 9 of the article/KIM1/Genta+Ellagic acid/1/1.tif]

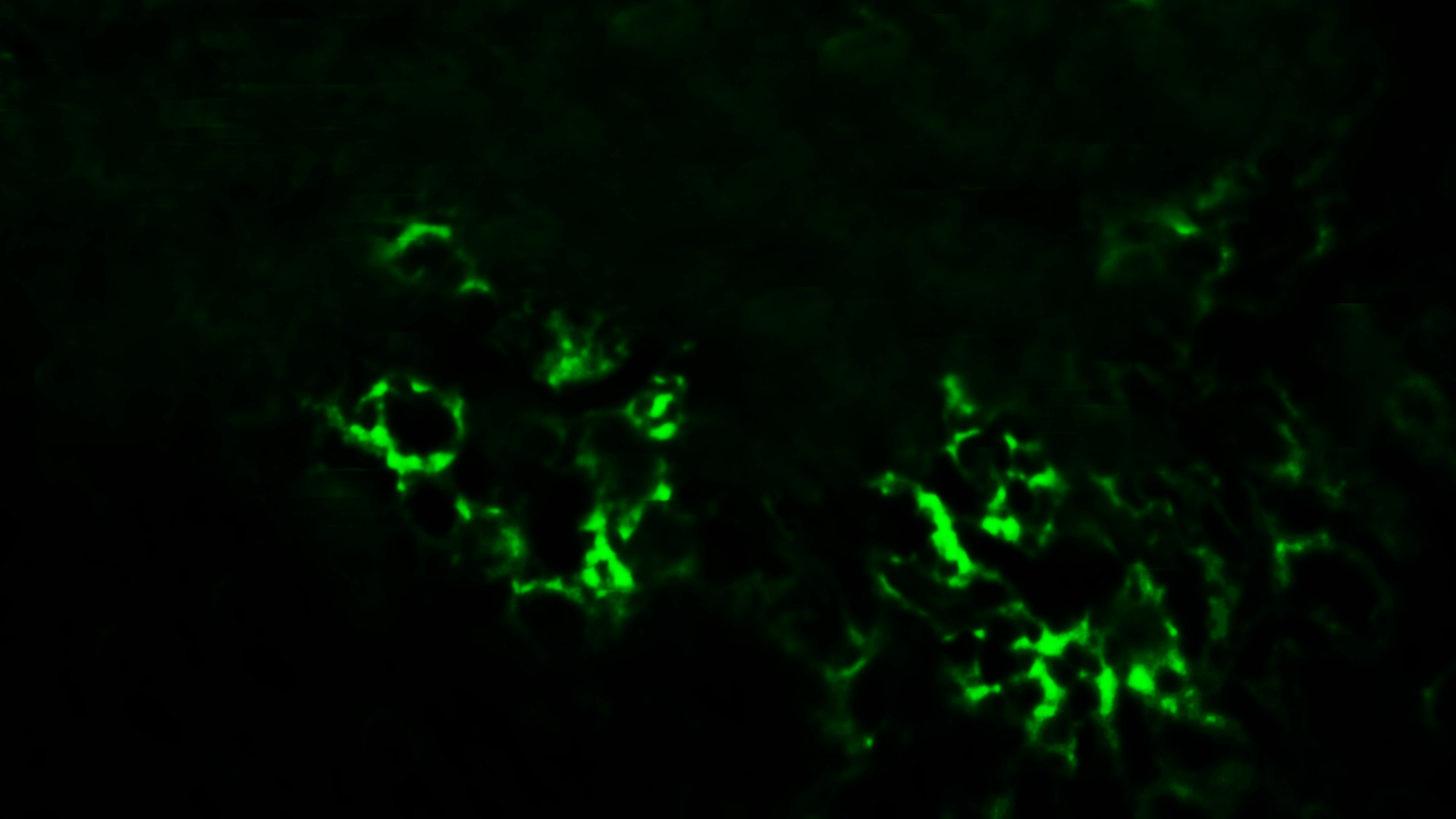

Supplement: Supplementary file 1 [file biomedicines-14-01385-s001.zip › biomedicines-4229880_Raw_Images_Figures_7-11.zipw folder/Original microscopy imgesRaw immunofluorescence results of Figures 7, 8, and 9 of the article/KIM1/Genta+Ellagic acid/1/2.tif]

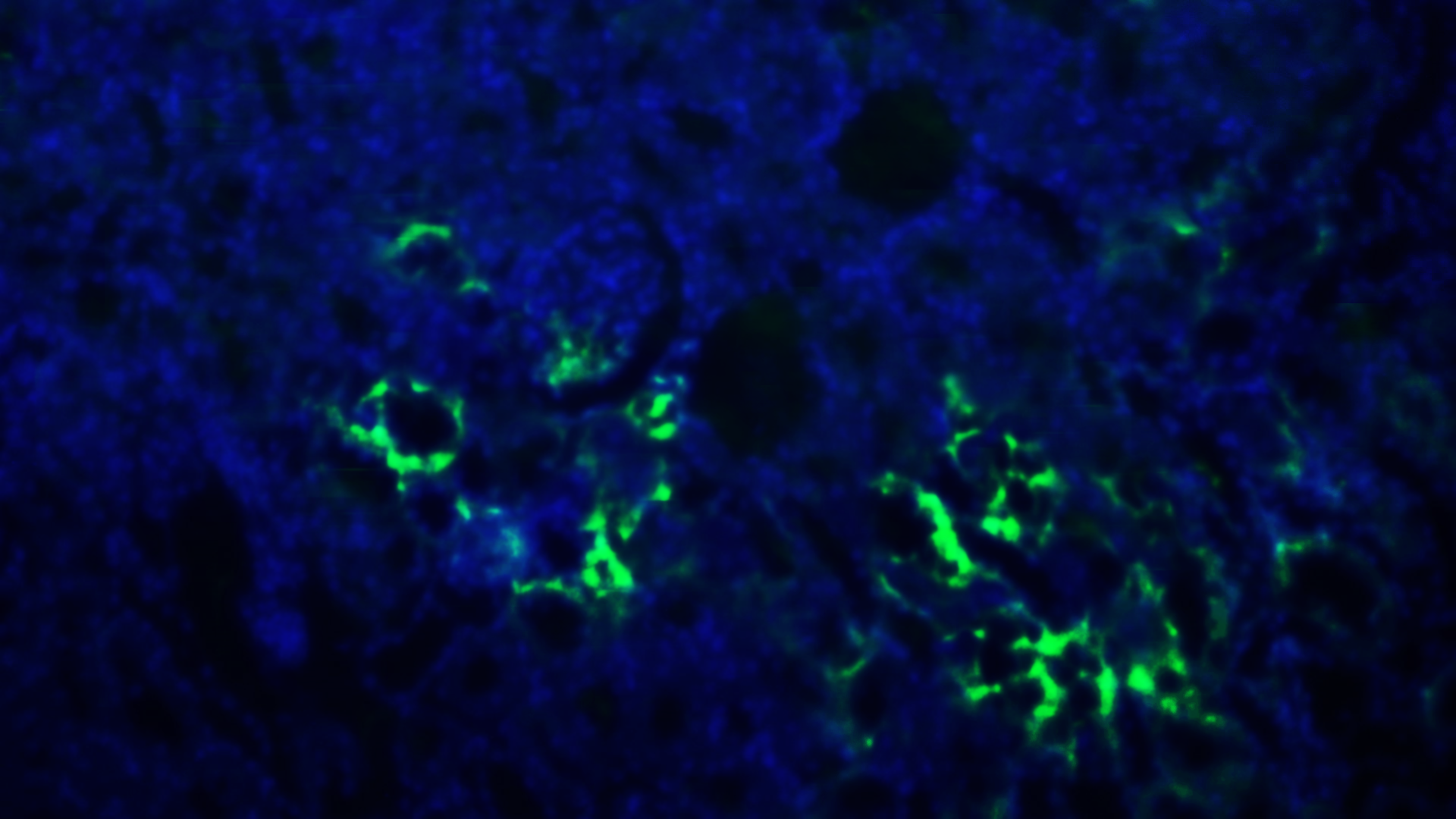

Supplement: Supplementary file 1 [file biomedicines-14-01385-s001.zip › biomedicines-4229880_Raw_Images_Figures_7-11.zipw folder/Original microscopy imgesRaw immunofluorescence results of Figures 7, 8, and 9 of the article/KIM1/Genta+Ellagic acid/1/3.tif]

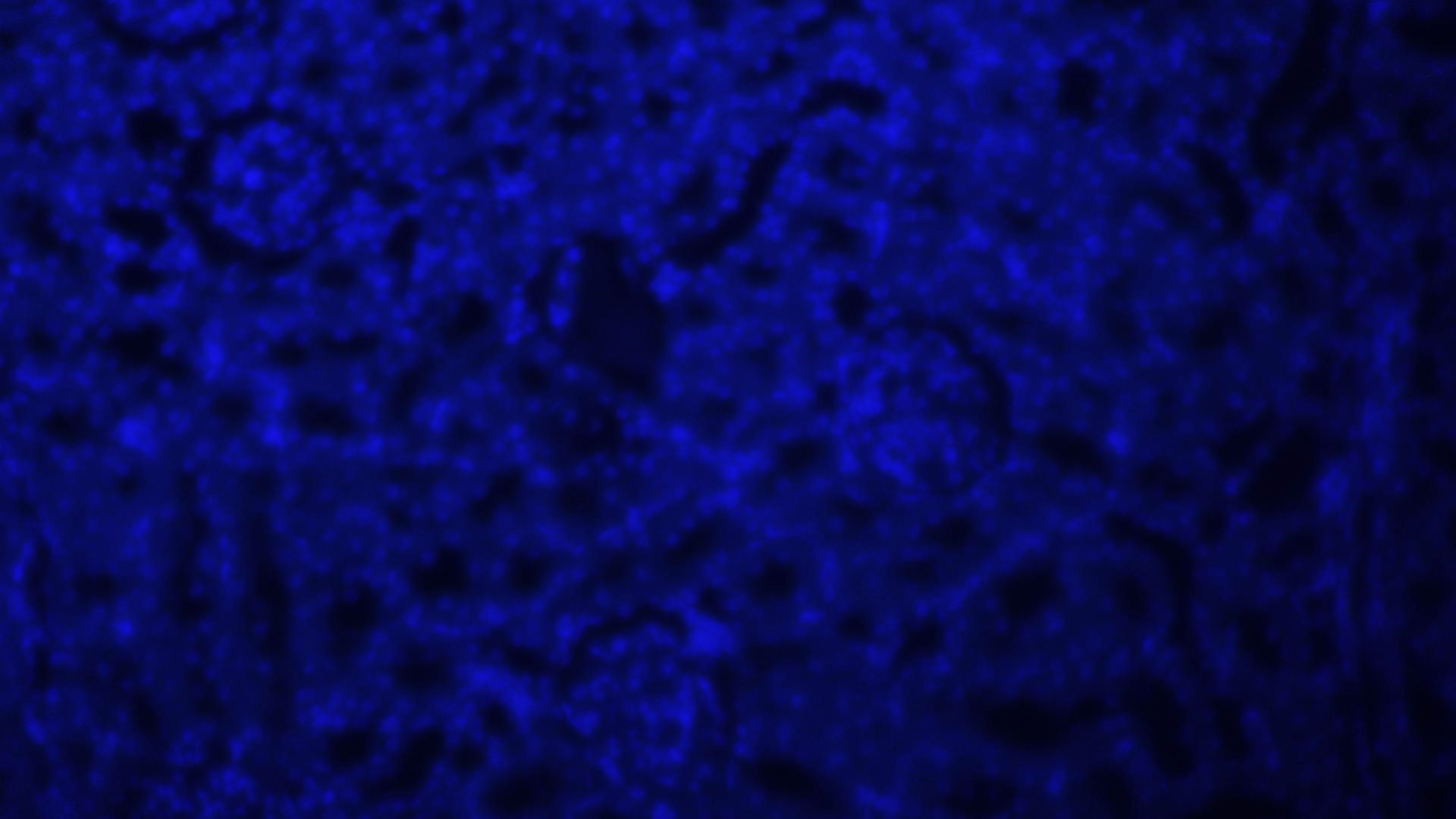

Supplement: Supplementary file 1 [file biomedicines-14-01385-s001.zip › biomedicines-4229880_Raw_Images_Figures_7-11.zipw folder/Original microscopy imgesRaw immunofluorescence results of Figures 7, 8, and 9 of the article/KIM1/Genta+Ellagic acid/2/1.tif]

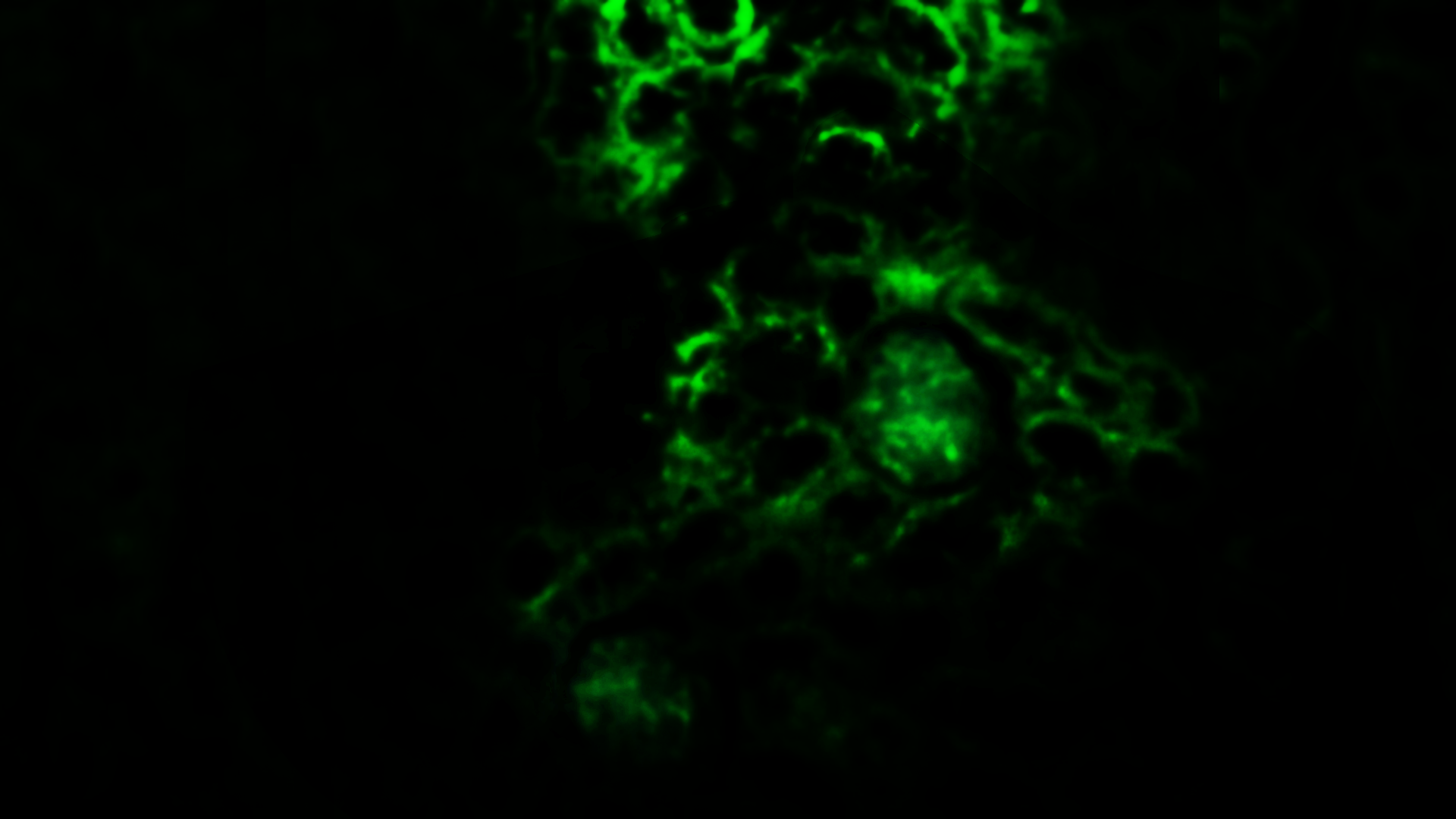

Supplement: Supplementary file 1 [file biomedicines-14-01385-s001.zip › biomedicines-4229880_Raw_Images_Figures_7-11.zipw folder/Original microscopy imgesRaw immunofluorescence results of Figures 7, 8, and 9 of the article/KIM1/Genta+Ellagic acid/2/2.tif]

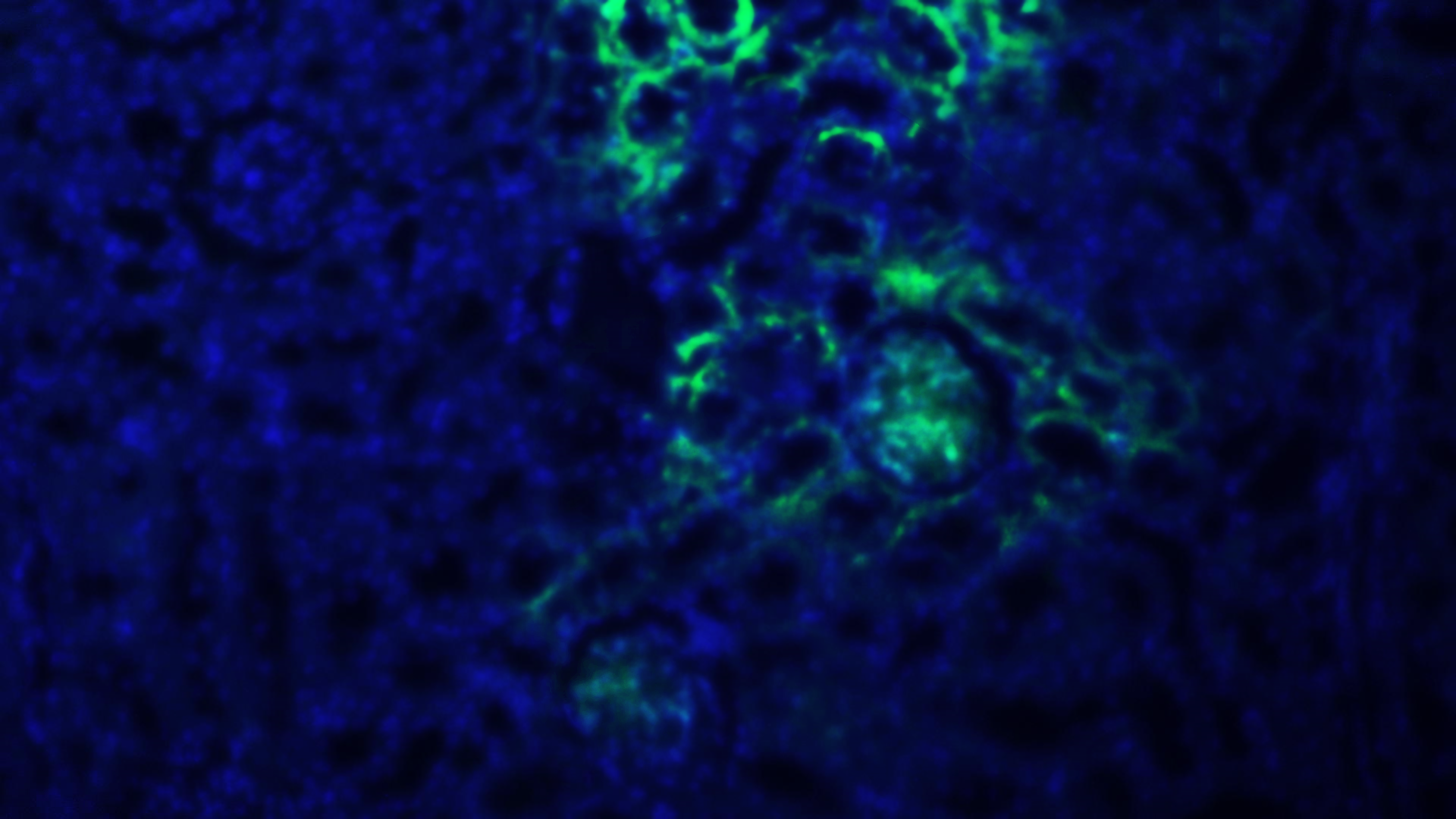

Supplement: Supplementary file 1 [file biomedicines-14-01385-s001.zip › biomedicines-4229880_Raw_Images_Figures_7-11.zipw folder/Original microscopy imgesRaw immunofluorescence results of Figures 7, 8, and 9 of the article/KIM1/Genta+Ellagic acid/2/3.tif]

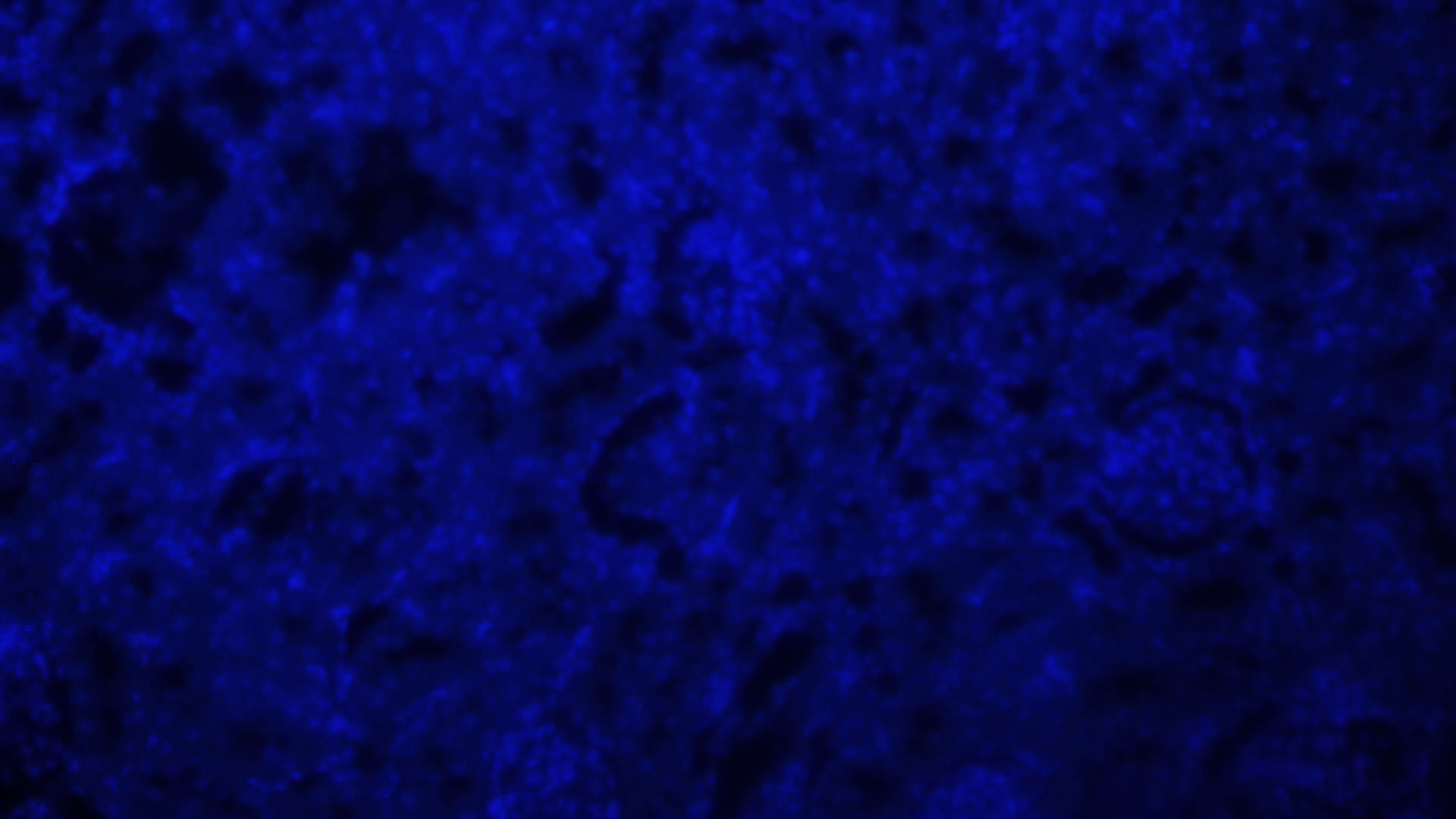

Supplement: Supplementary file 1 [file biomedicines-14-01385-s001.zip › biomedicines-4229880_Raw_Images_Figures_7-11.zipw folder/Original microscopy imgesRaw immunofluorescence results of Figures 7, 8, and 9 of the article/KIM1/Genta+Ellagic acid/3/1.tif]

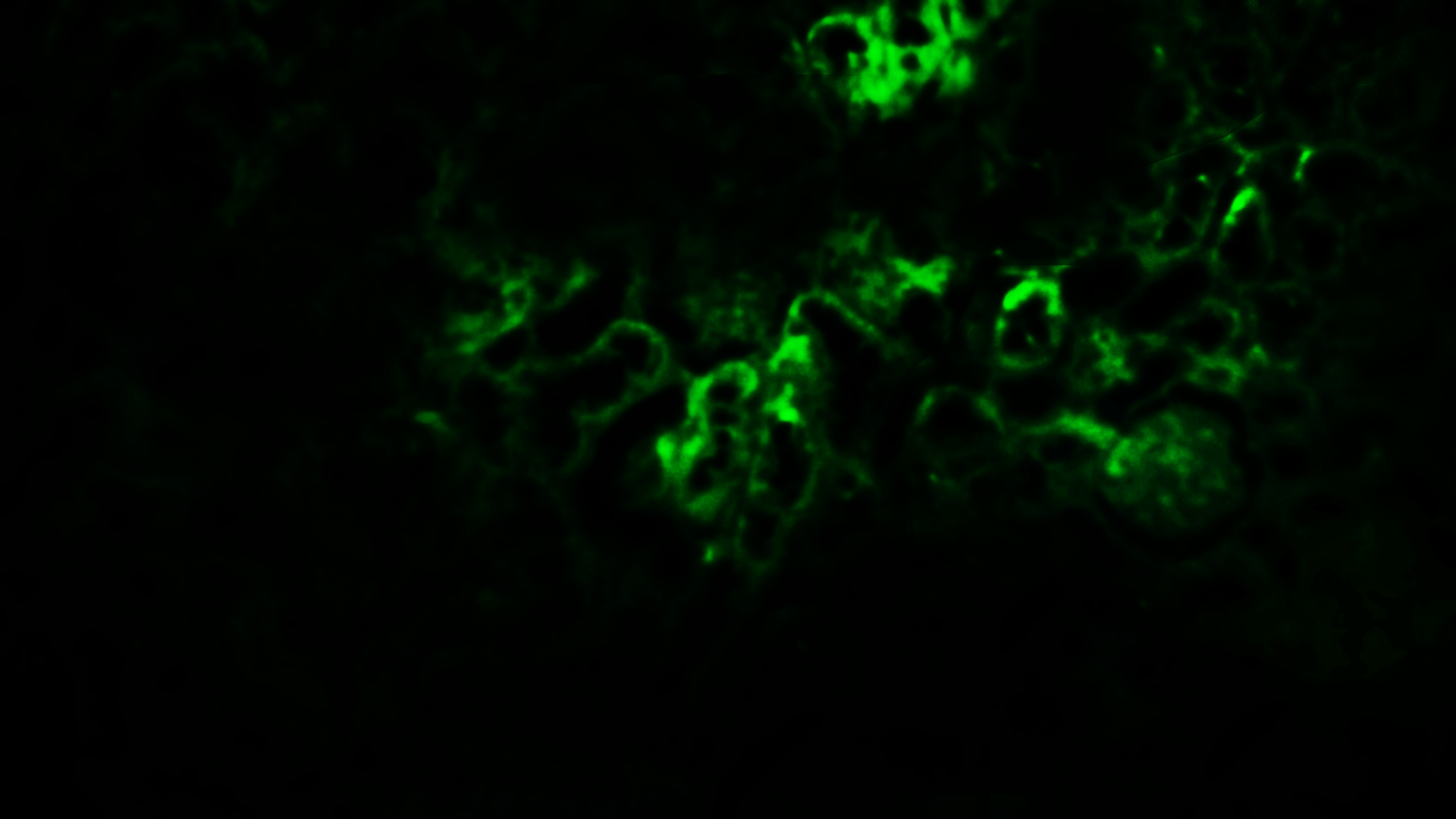

Supplement: Supplementary file 1 [file biomedicines-14-01385-s001.zip › biomedicines-4229880_Raw_Images_Figures_7-11.zipw folder/Original microscopy imgesRaw immunofluorescence results of Figures 7, 8, and 9 of the article/KIM1/Genta+Ellagic acid/3/2.tif]

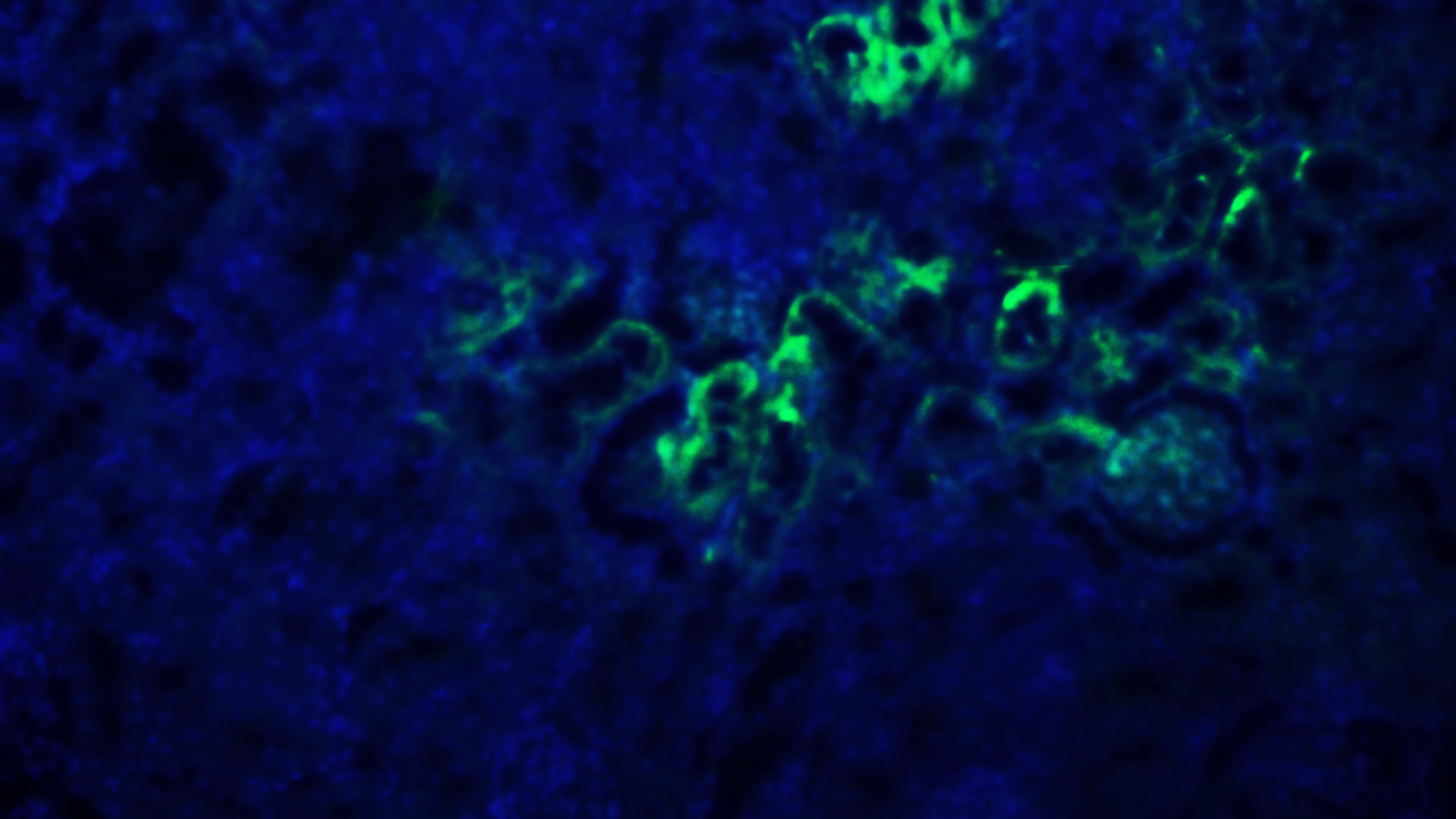

Supplement: Supplementary file 1 [file biomedicines-14-01385-s001.zip › biomedicines-4229880_Raw_Images_Figures_7-11.zipw folder/Original microscopy imgesRaw immunofluorescence results of Figures 7, 8, and 9 of the article/KIM1/Genta+Ellagic acid/3/3.tif]

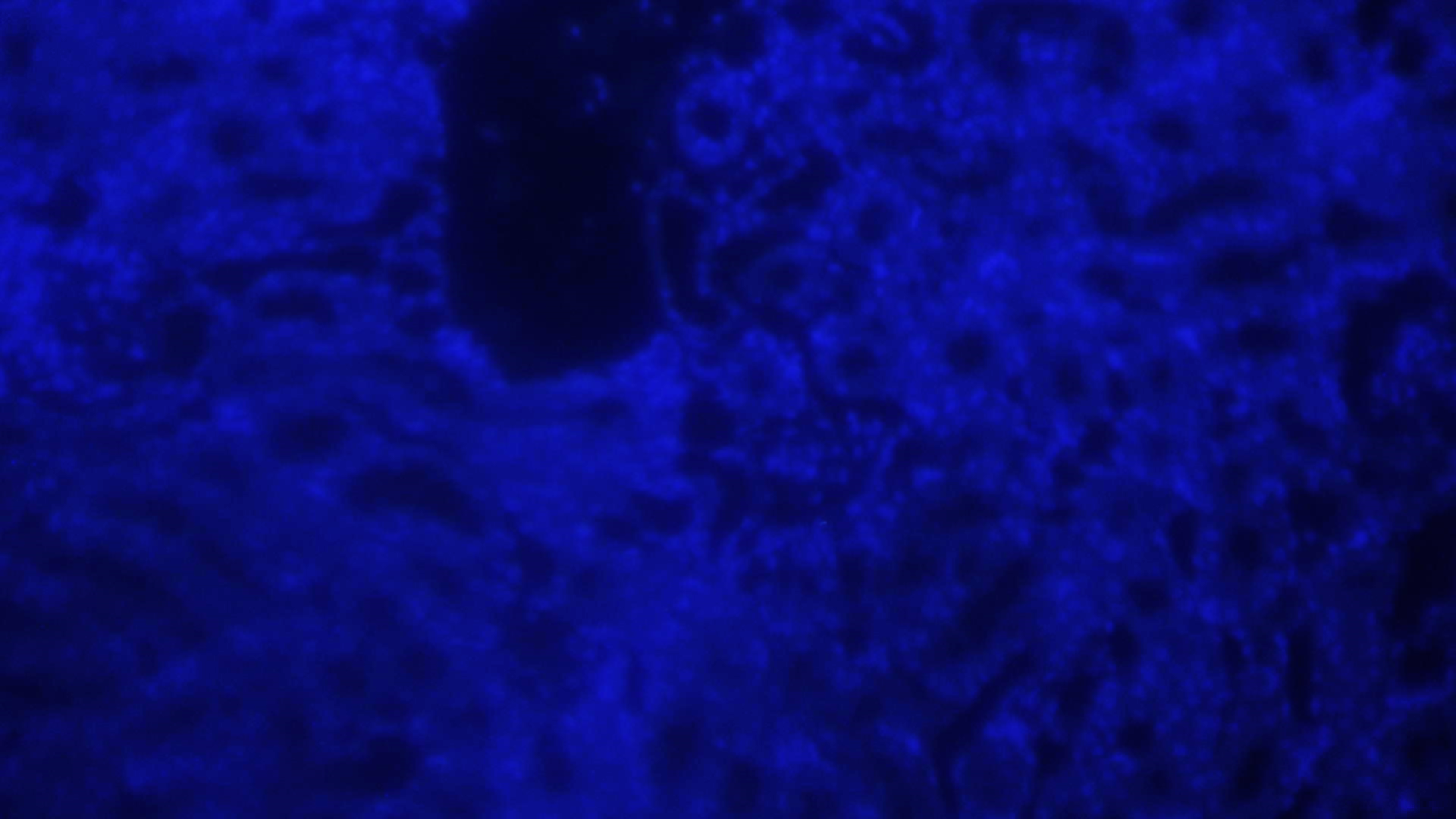

Supplement: Supplementary file 1 [file biomedicines-14-01385-s001.zip › biomedicines-4229880_Raw_Images_Figures_7-11.zipw folder/Original microscopy imgesRaw immunofluorescence results of Figures 7, 8, and 9 of the article/KIM1/Gentamicin/1/1.tif]

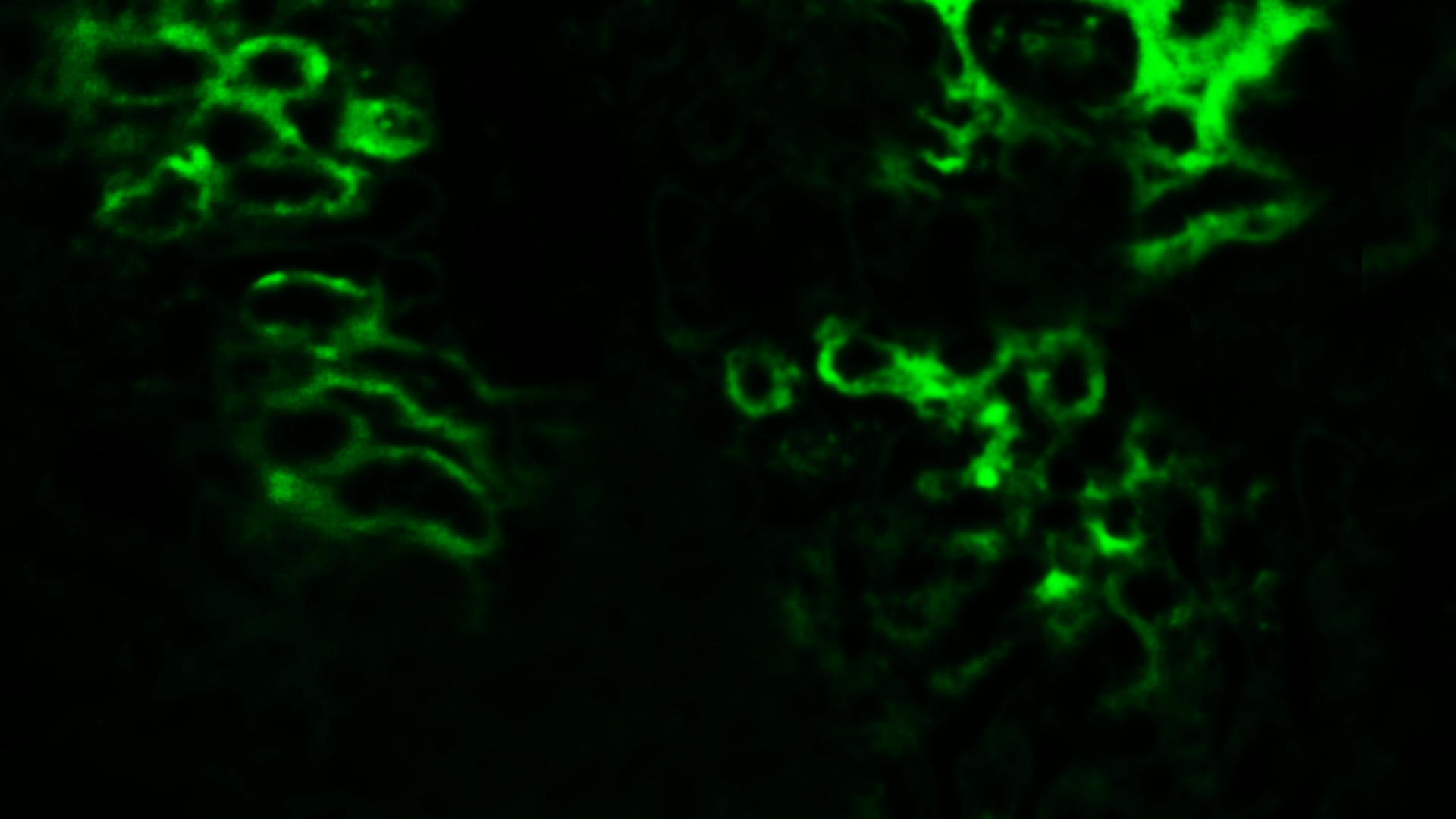

Supplement: Supplementary file 1 [file biomedicines-14-01385-s001.zip › biomedicines-4229880_Raw_Images_Figures_7-11.zipw folder/Original microscopy imgesRaw immunofluorescence results of Figures 7, 8, and 9 of the article/KIM1/Gentamicin/1/2.tif]

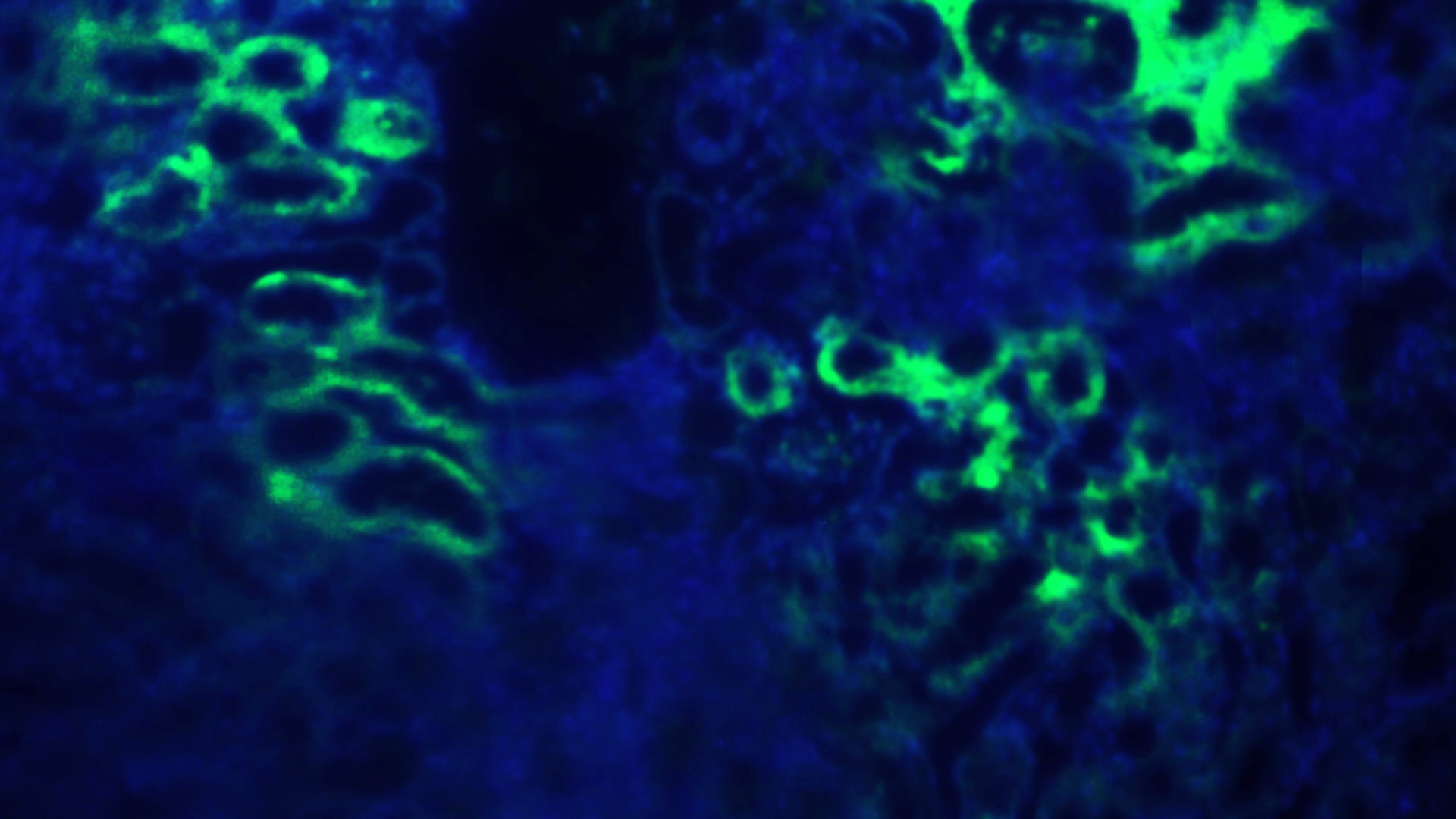

Supplement: Supplementary file 1 [file biomedicines-14-01385-s001.zip › biomedicines-4229880_Raw_Images_Figures_7-11.zipw folder/Original microscopy imgesRaw immunofluorescence results of Figures 7, 8, and 9 of the article/KIM1/Gentamicin/1/3.tif]

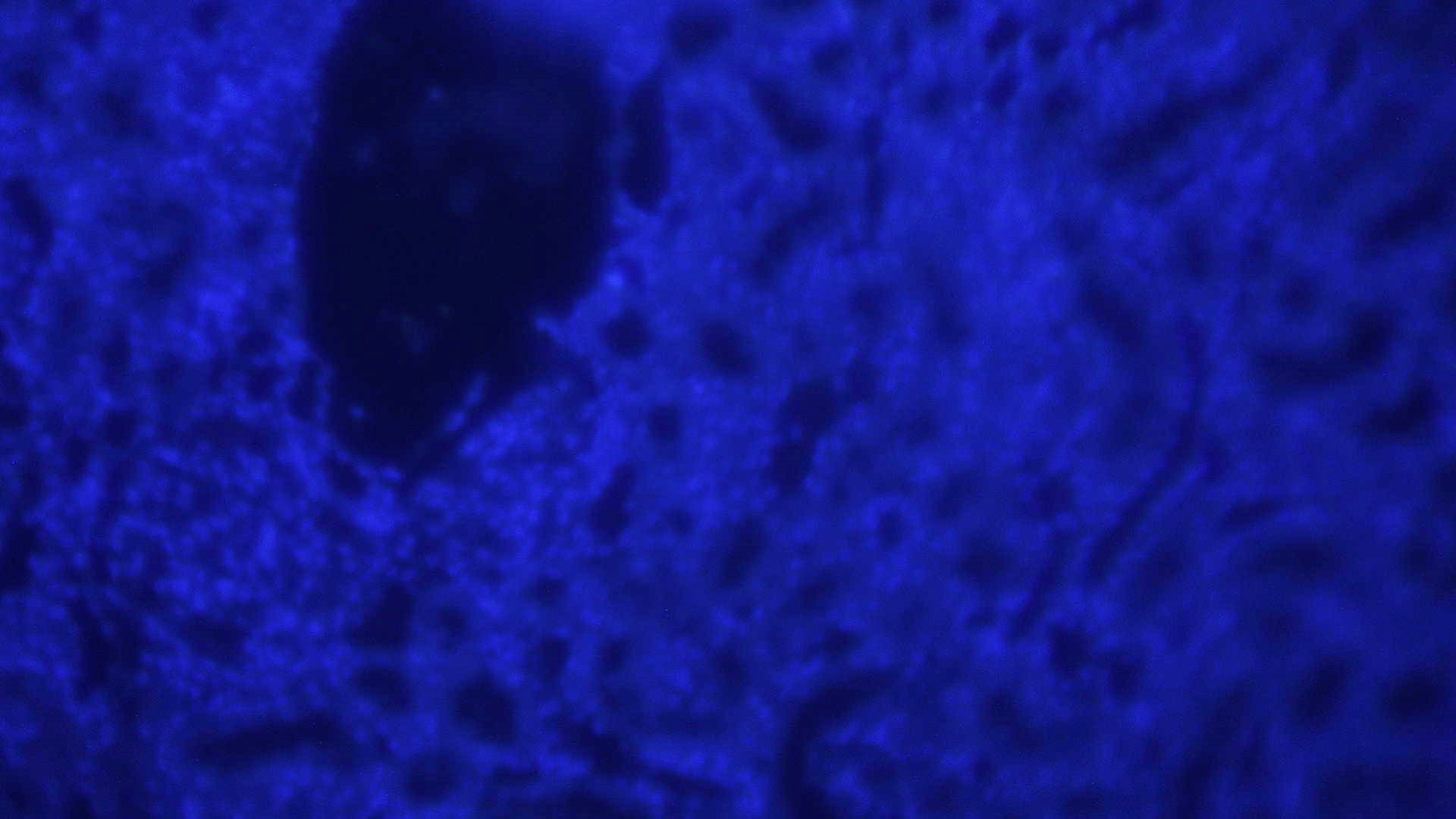

Supplement: Supplementary file 1 [file biomedicines-14-01385-s001.zip › biomedicines-4229880_Raw_Images_Figures_7-11.zipw folder/Original microscopy imgesRaw immunofluorescence results of Figures 7, 8, and 9 of the article/KIM1/Gentamicin/2/1.tif]

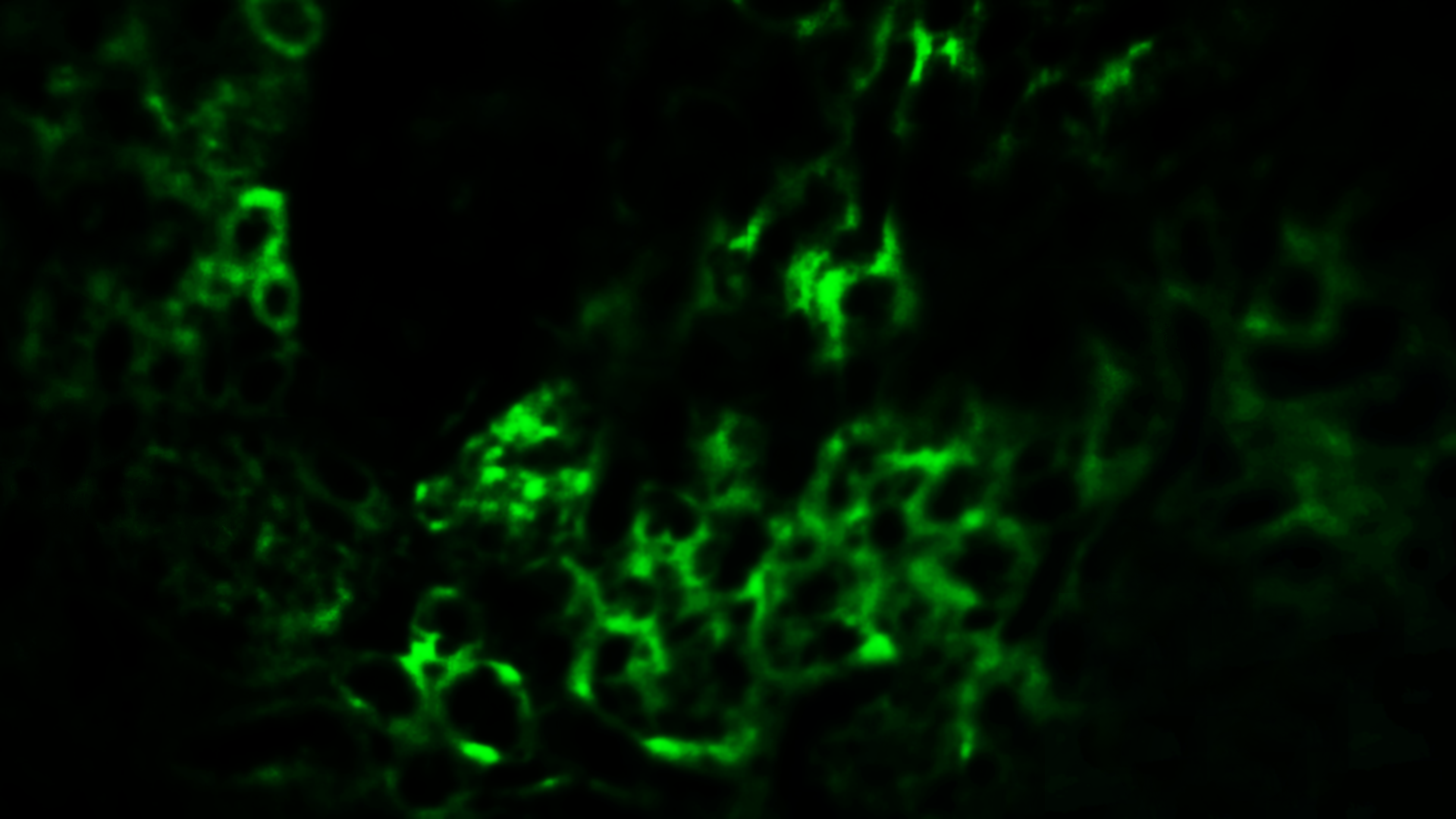

Supplement: Supplementary file 1 [file biomedicines-14-01385-s001.zip › biomedicines-4229880_Raw_Images_Figures_7-11.zipw folder/Original microscopy imgesRaw immunofluorescence results of Figures 7, 8, and 9 of the article/KIM1/Gentamicin/2/2.tif]

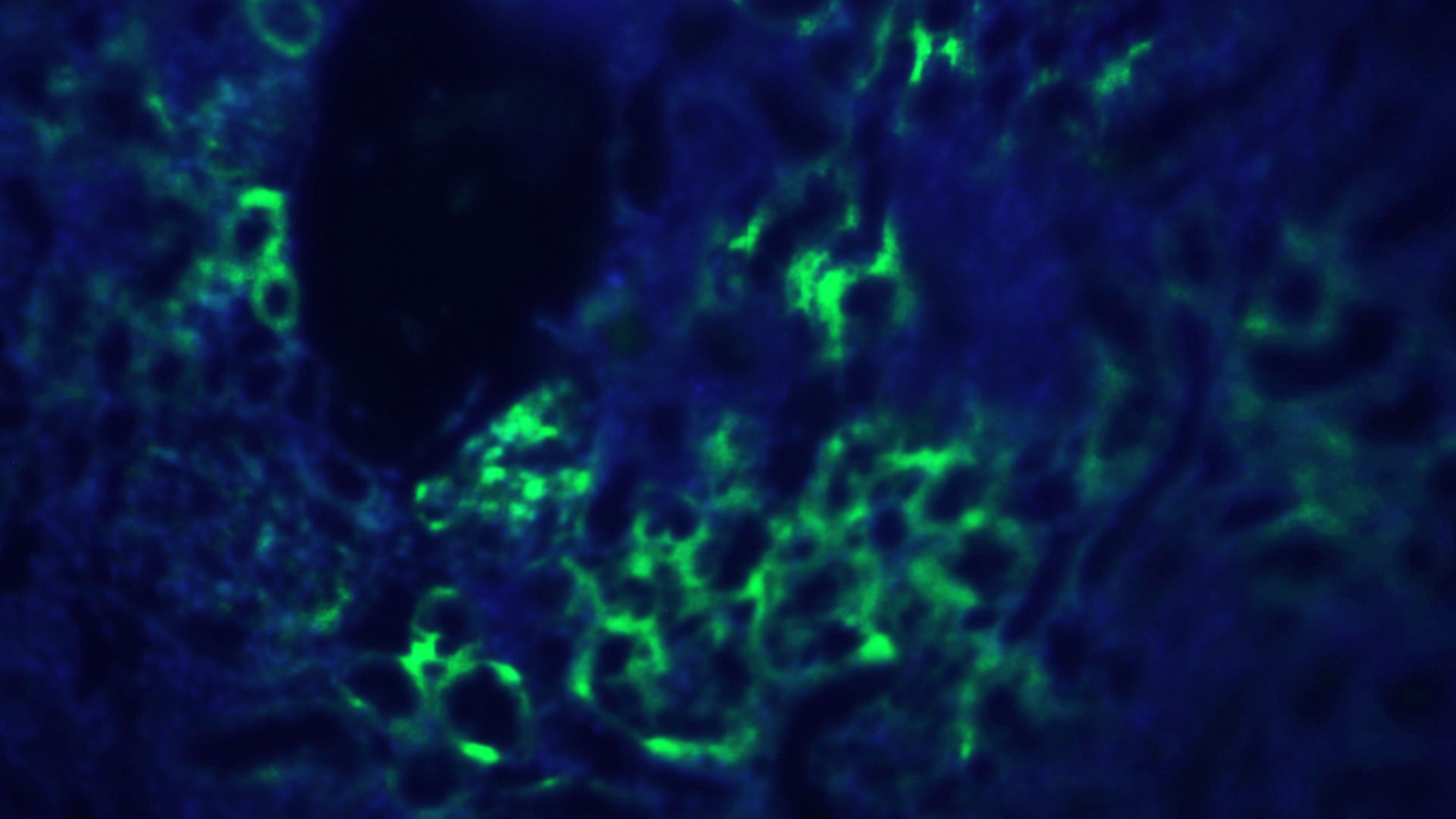

Supplement: Supplementary file 1 [file biomedicines-14-01385-s001.zip › biomedicines-4229880_Raw_Images_Figures_7-11.zipw folder/Original microscopy imgesRaw immunofluorescence results of Figures 7, 8, and 9 of the article/KIM1/Gentamicin/2/3.tif]

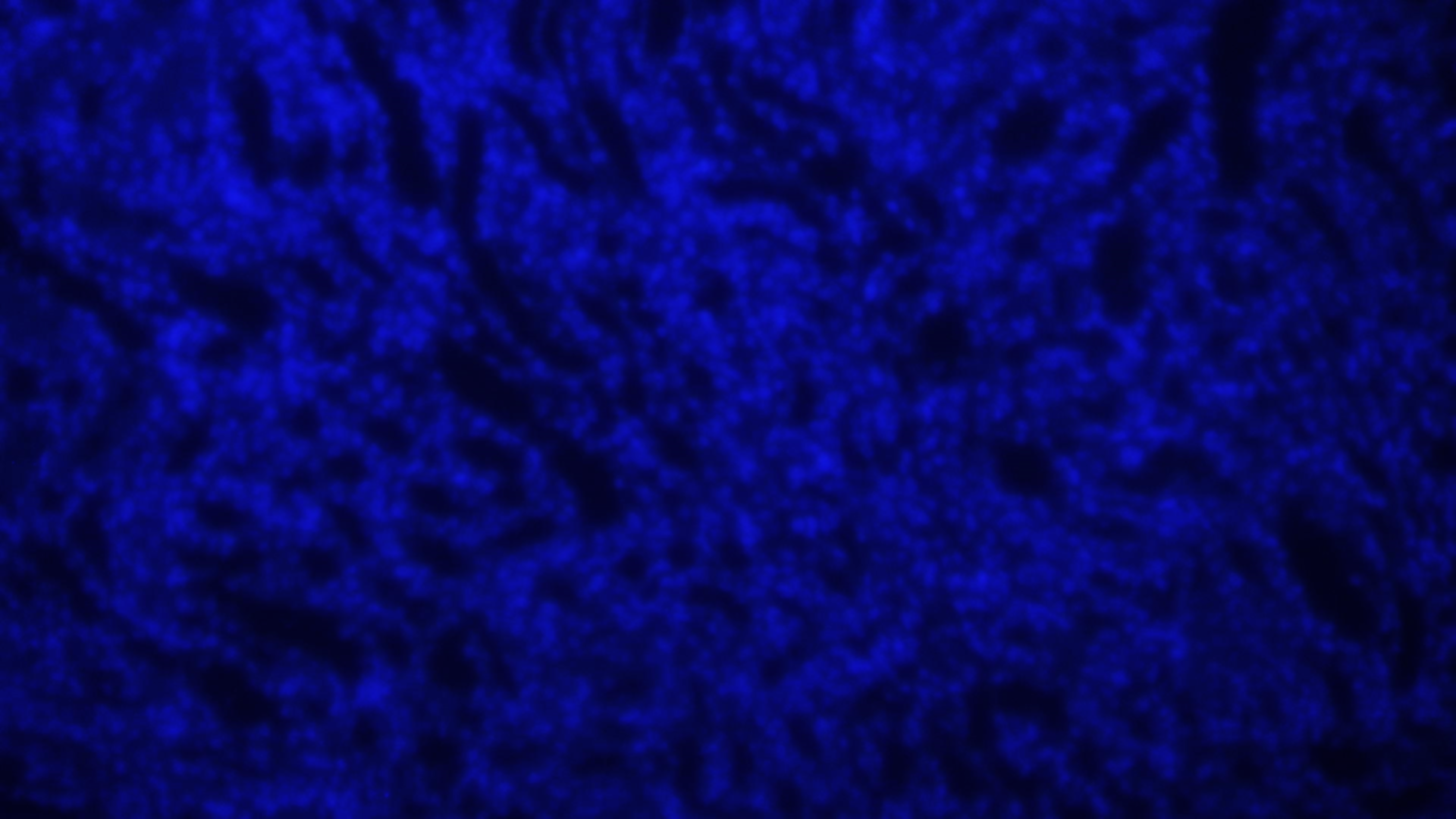

Supplement: Supplementary file 1 [file biomedicines-14-01385-s001.zip › biomedicines-4229880_Raw_Images_Figures_7-11.zipw folder/Original microscopy imgesRaw immunofluorescence results of Figures 7, 8, and 9 of the article/KIM1/Gentamicin/3/1.tif]

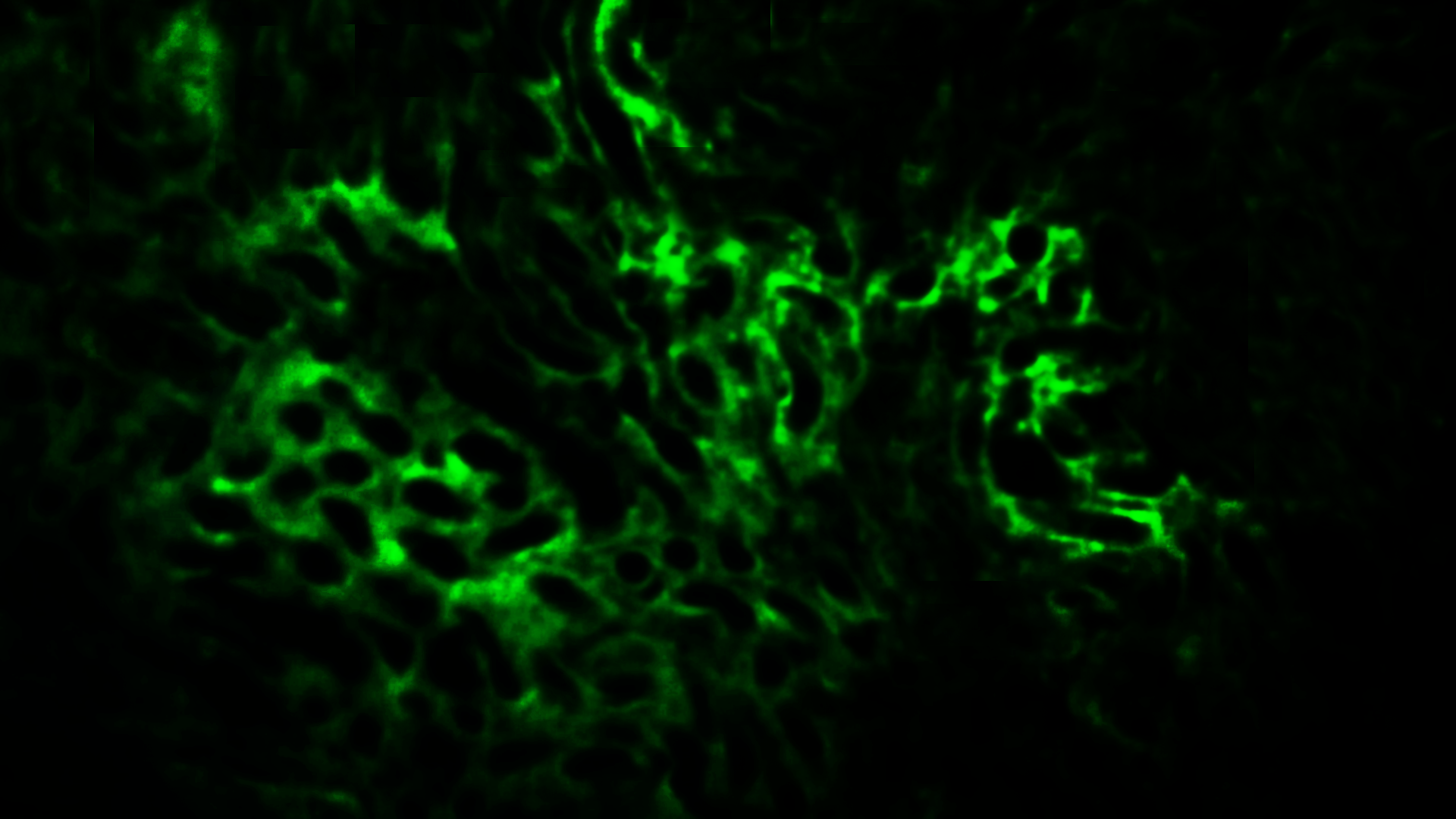

Supplement: Supplementary file 1 [file biomedicines-14-01385-s001.zip › biomedicines-4229880_Raw_Images_Figures_7-11.zipw folder/Original microscopy imgesRaw immunofluorescence results of Figures 7, 8, and 9 of the article/KIM1/Gentamicin/3/2.tif]

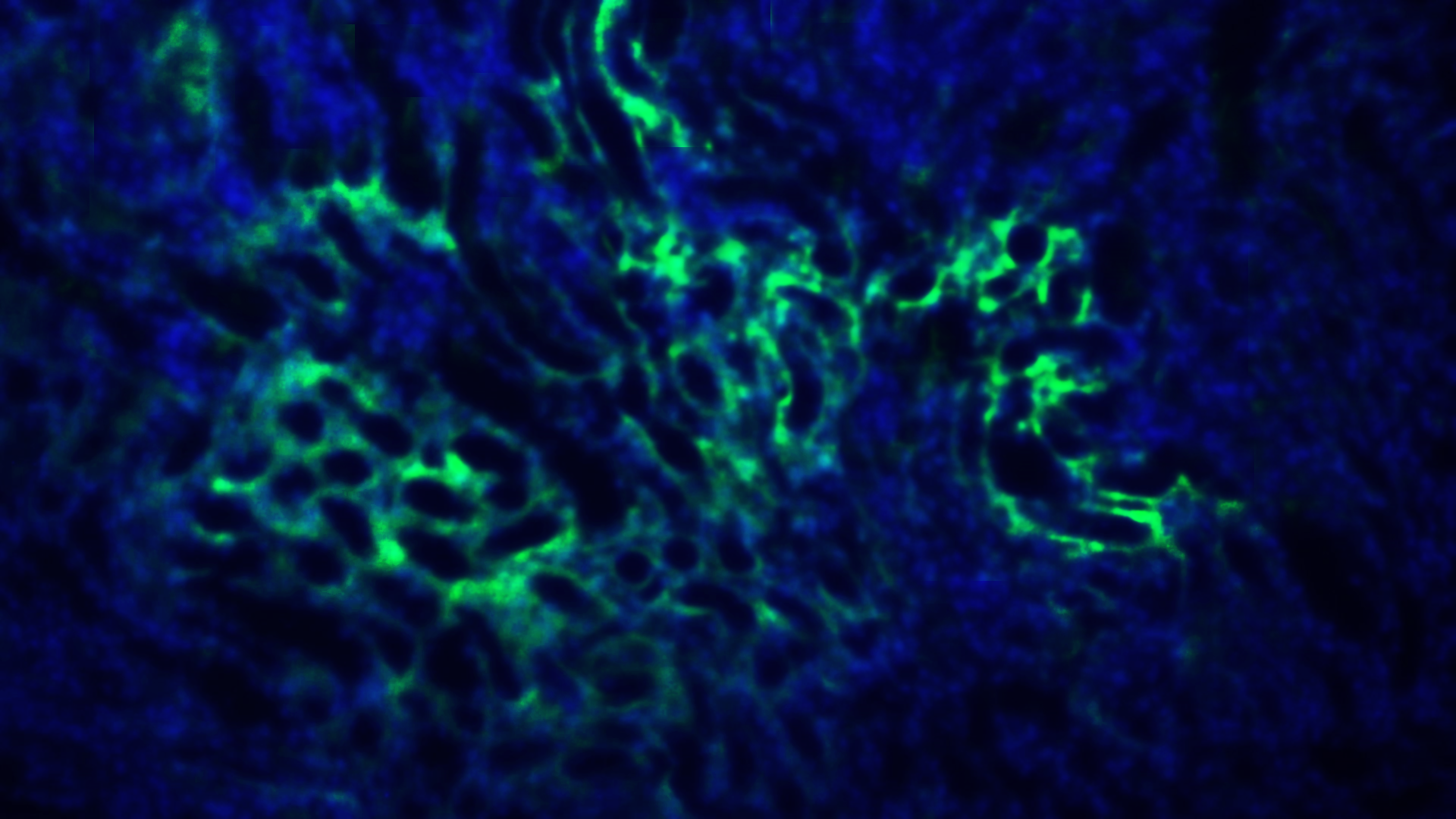

Supplement: Supplementary file 1 [file biomedicines-14-01385-s001.zip › biomedicines-4229880_Raw_Images_Figures_7-11.zipw folder/Original microscopy imgesRaw immunofluorescence results of Figures 7, 8, and 9 of the article/KIM1/Gentamicin/3/3.tif]

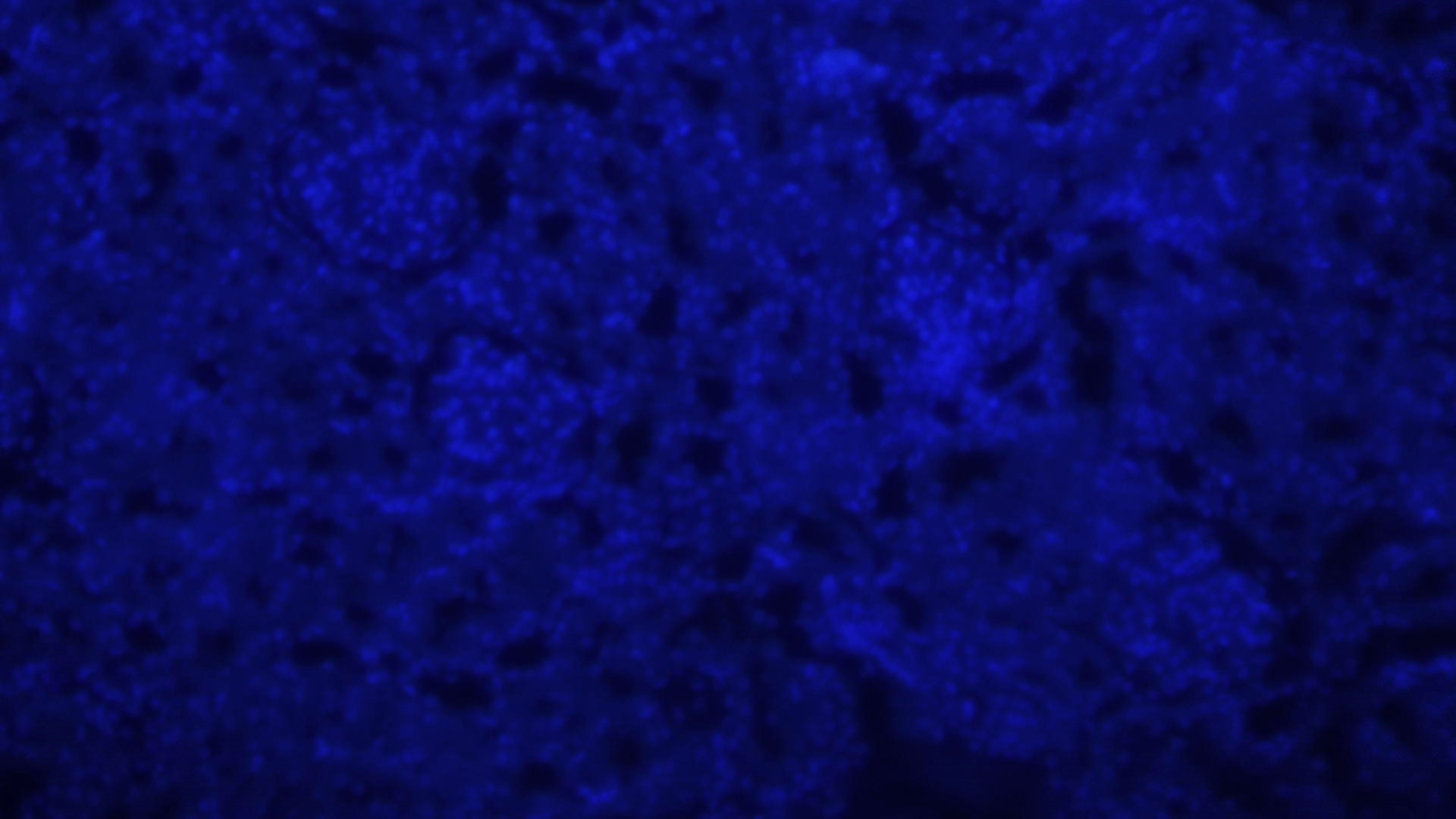

Supplement: Supplementary file 1 [file biomedicines-14-01385-s001.zip › biomedicines-4229880_Raw_Images_Figures_7-11.zipw folder/Original microscopy imgesRaw immunofluorescence results of Figures 7, 8, and 9 of the article/LC3A/Control/1/1.tif]

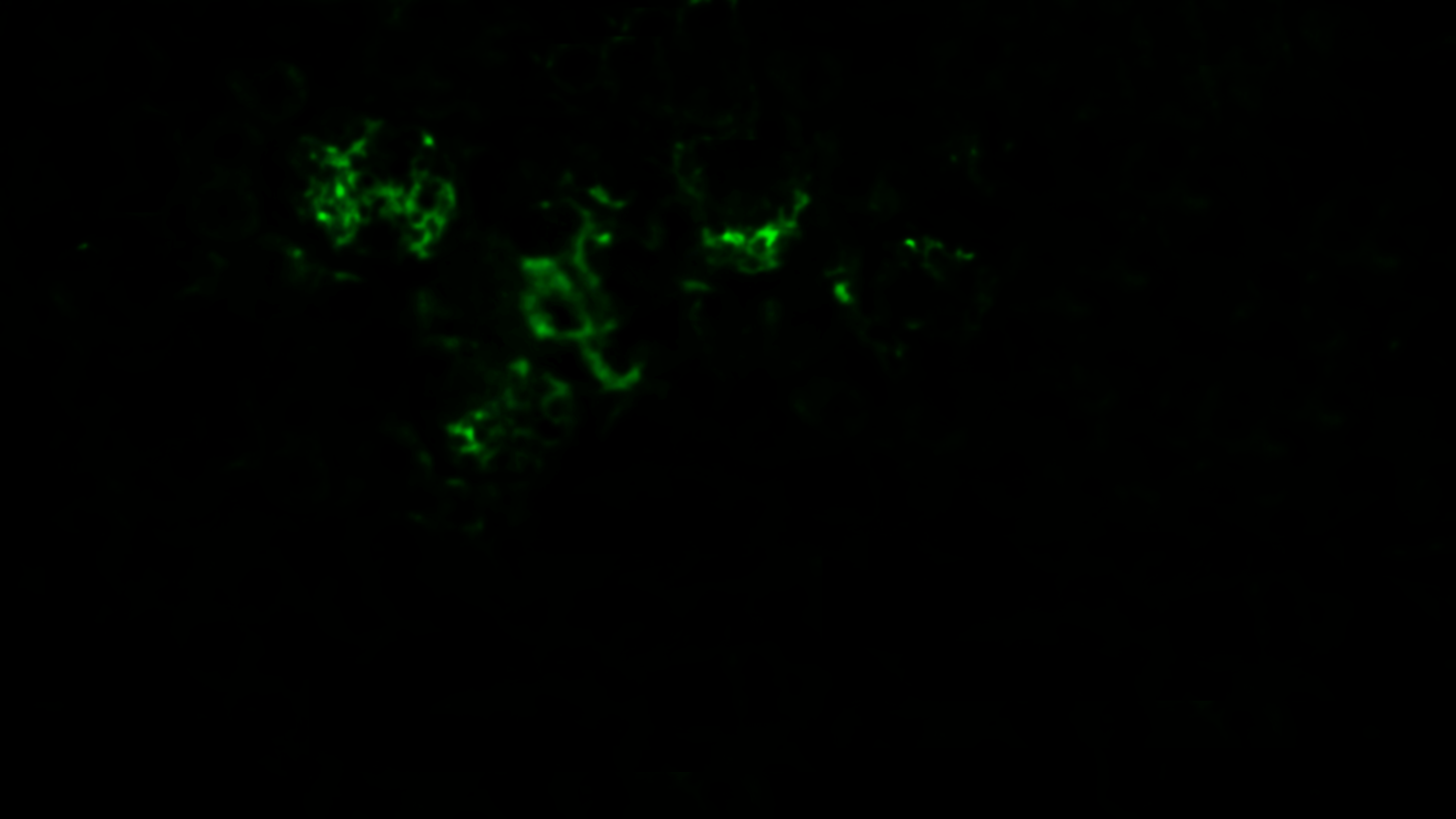

Supplement: Supplementary file 1 [file biomedicines-14-01385-s001.zip › biomedicines-4229880_Raw_Images_Figures_7-11.zipw folder/Original microscopy imgesRaw immunofluorescence results of Figures 7, 8, and 9 of the article/LC3A/Control/1/2.tif]

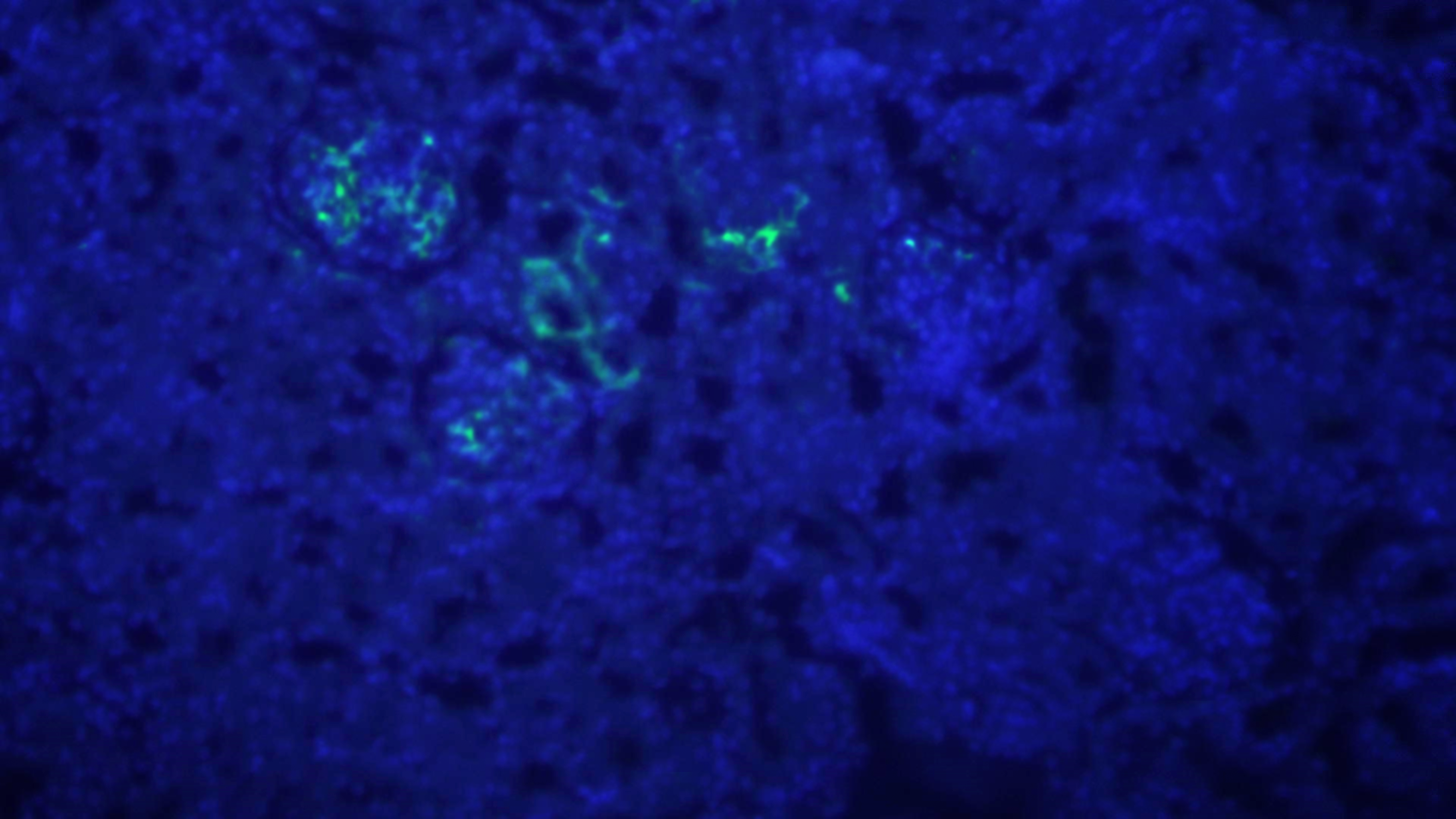

Supplement: Supplementary file 1 [file biomedicines-14-01385-s001.zip › biomedicines-4229880_Raw_Images_Figures_7-11.zipw folder/Original microscopy imgesRaw immunofluorescence results of Figures 7, 8, and 9 of the article/LC3A/Control/1/3.tif]

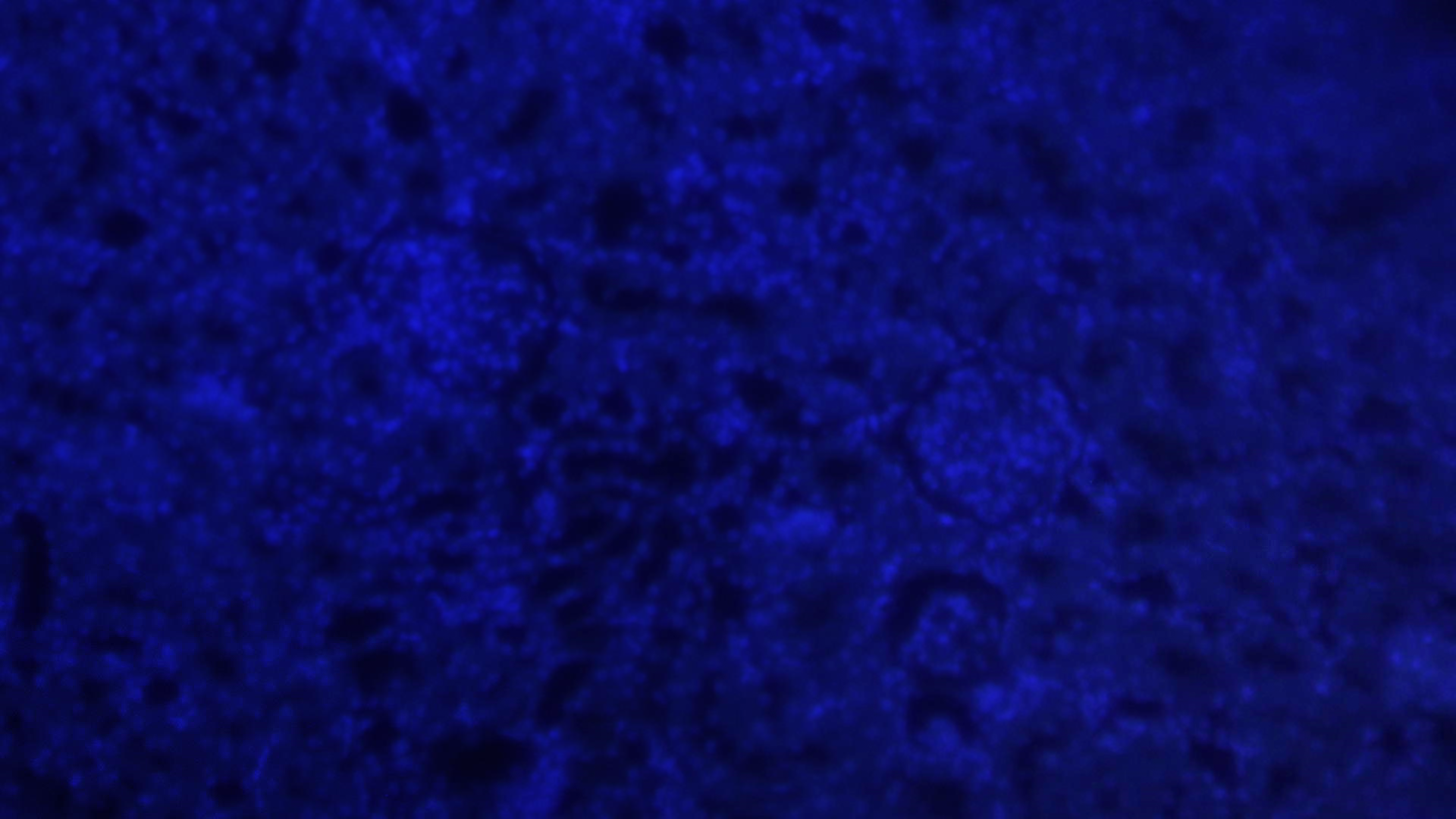

Supplement: Supplementary file 1 [file biomedicines-14-01385-s001.zip › biomedicines-4229880_Raw_Images_Figures_7-11.zipw folder/Original microscopy imgesRaw immunofluorescence results of Figures 7, 8, and 9 of the article/LC3A/Control/2/1.tif]

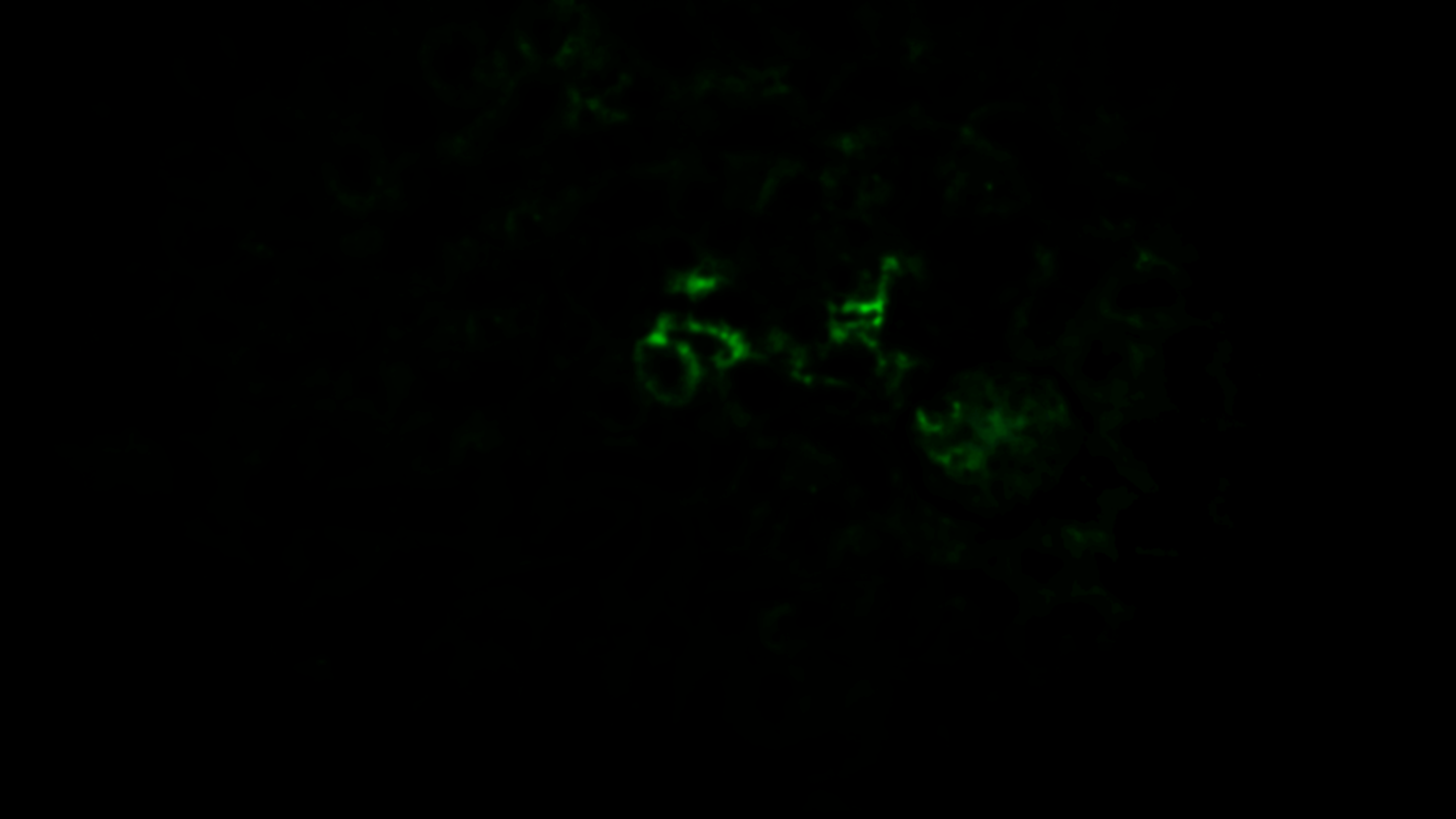

Supplement: Supplementary file 1 [file biomedicines-14-01385-s001.zip › biomedicines-4229880_Raw_Images_Figures_7-11.zipw folder/Original microscopy imgesRaw immunofluorescence results of Figures 7, 8, and 9 of the article/LC3A/Control/2/2.tif]

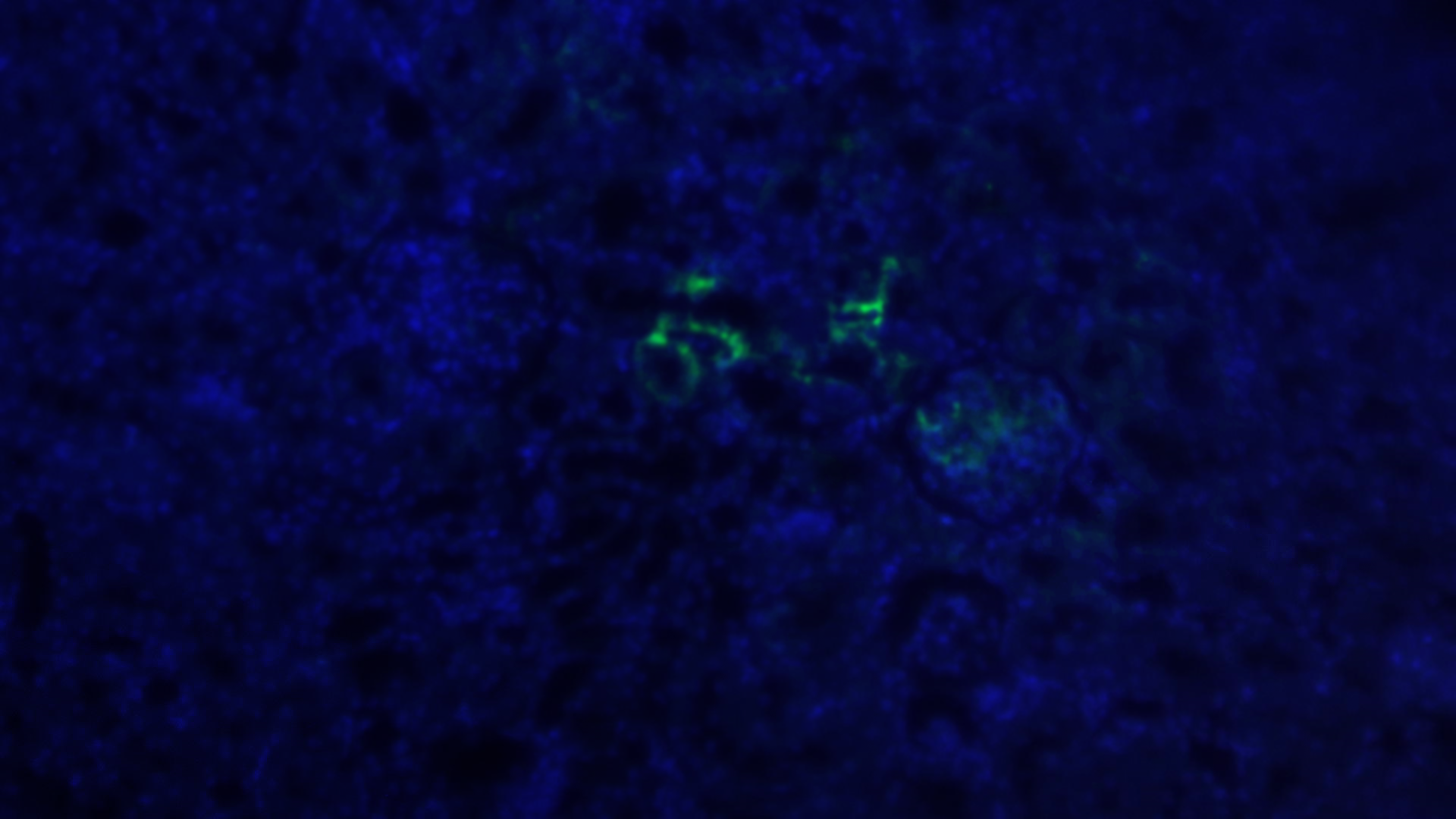

Supplement: Supplementary file 1 [file biomedicines-14-01385-s001.zip › biomedicines-4229880_Raw_Images_Figures_7-11.zipw folder/Original microscopy imgesRaw immunofluorescence results of Figures 7, 8, and 9 of the article/LC3A/Control/2/3.tif]

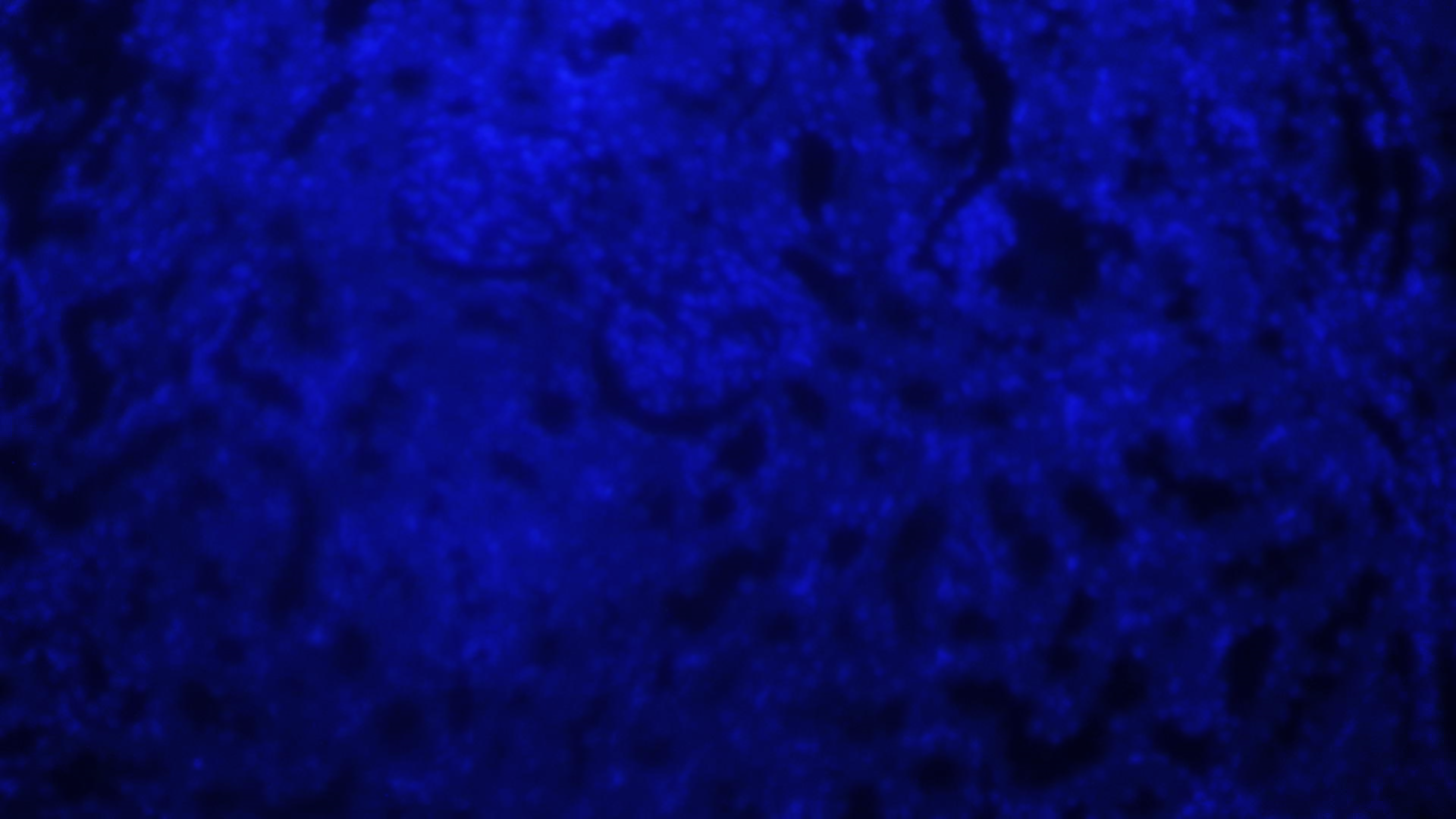

Supplement: Supplementary file 1 [file biomedicines-14-01385-s001.zip › biomedicines-4229880_Raw_Images_Figures_7-11.zipw folder/Original microscopy imgesRaw immunofluorescence results of Figures 7, 8, and 9 of the article/LC3A/Control/3/1.tif]

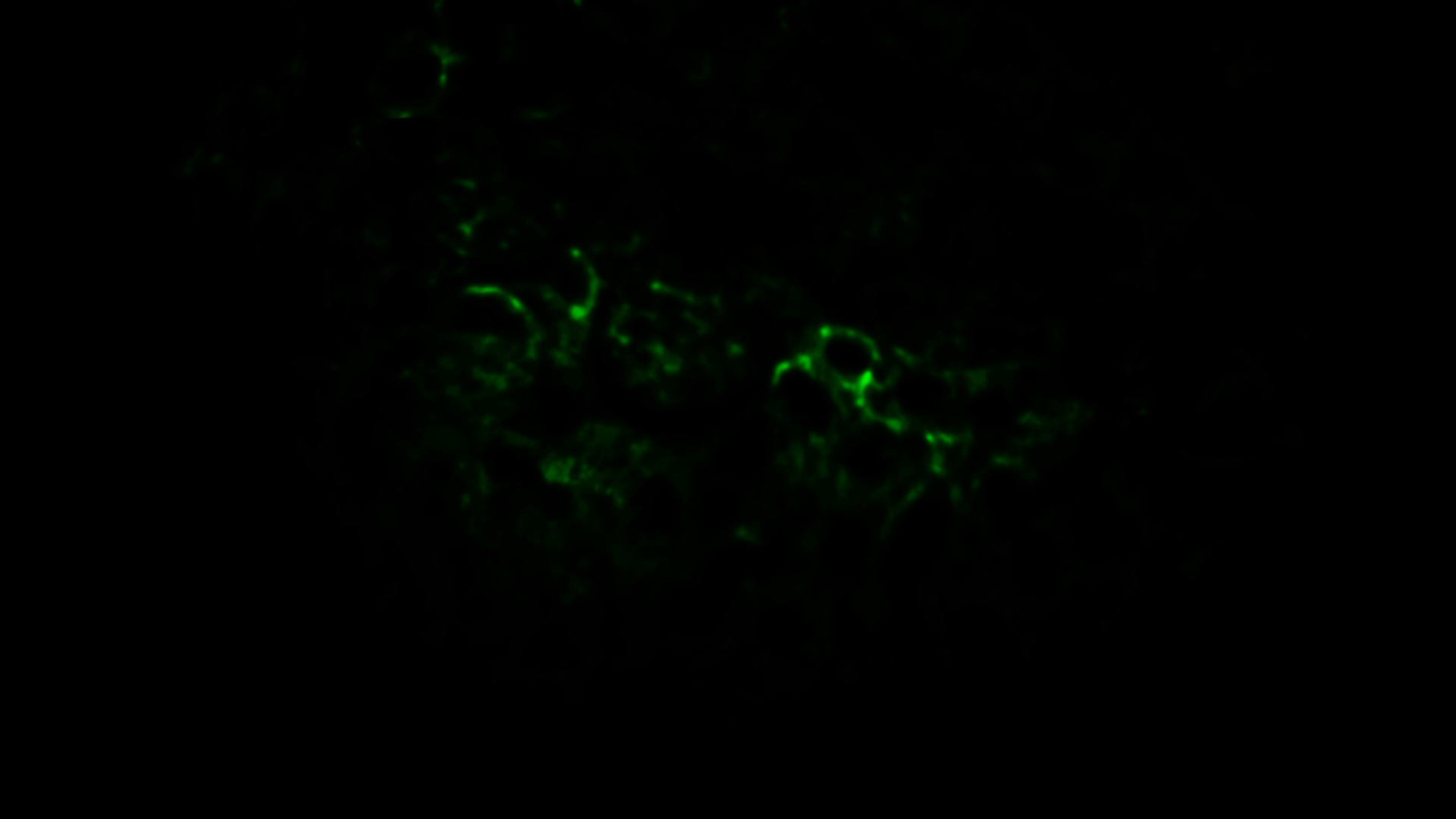

Supplement: Supplementary file 1 [file biomedicines-14-01385-s001.zip › biomedicines-4229880_Raw_Images_Figures_7-11.zipw folder/Original microscopy imgesRaw immunofluorescence results of Figures 7, 8, and 9 of the article/LC3A/Control/3/2.tif]

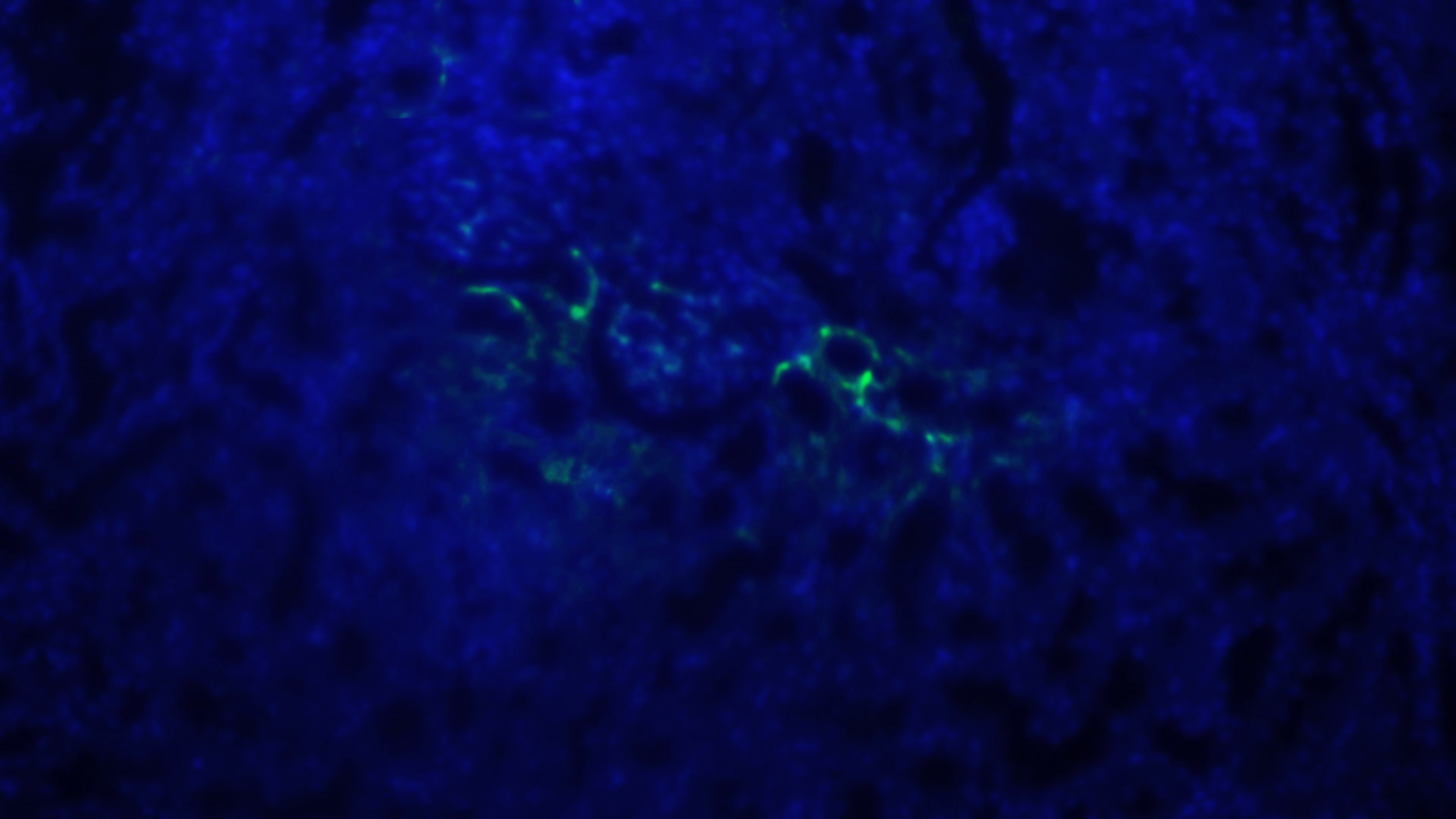

Supplement: Supplementary file 1 [file biomedicines-14-01385-s001.zip › biomedicines-4229880_Raw_Images_Figures_7-11.zipw folder/Original microscopy imgesRaw immunofluorescence results of Figures 7, 8, and 9 of the article/LC3A/Control/3/3.tif]

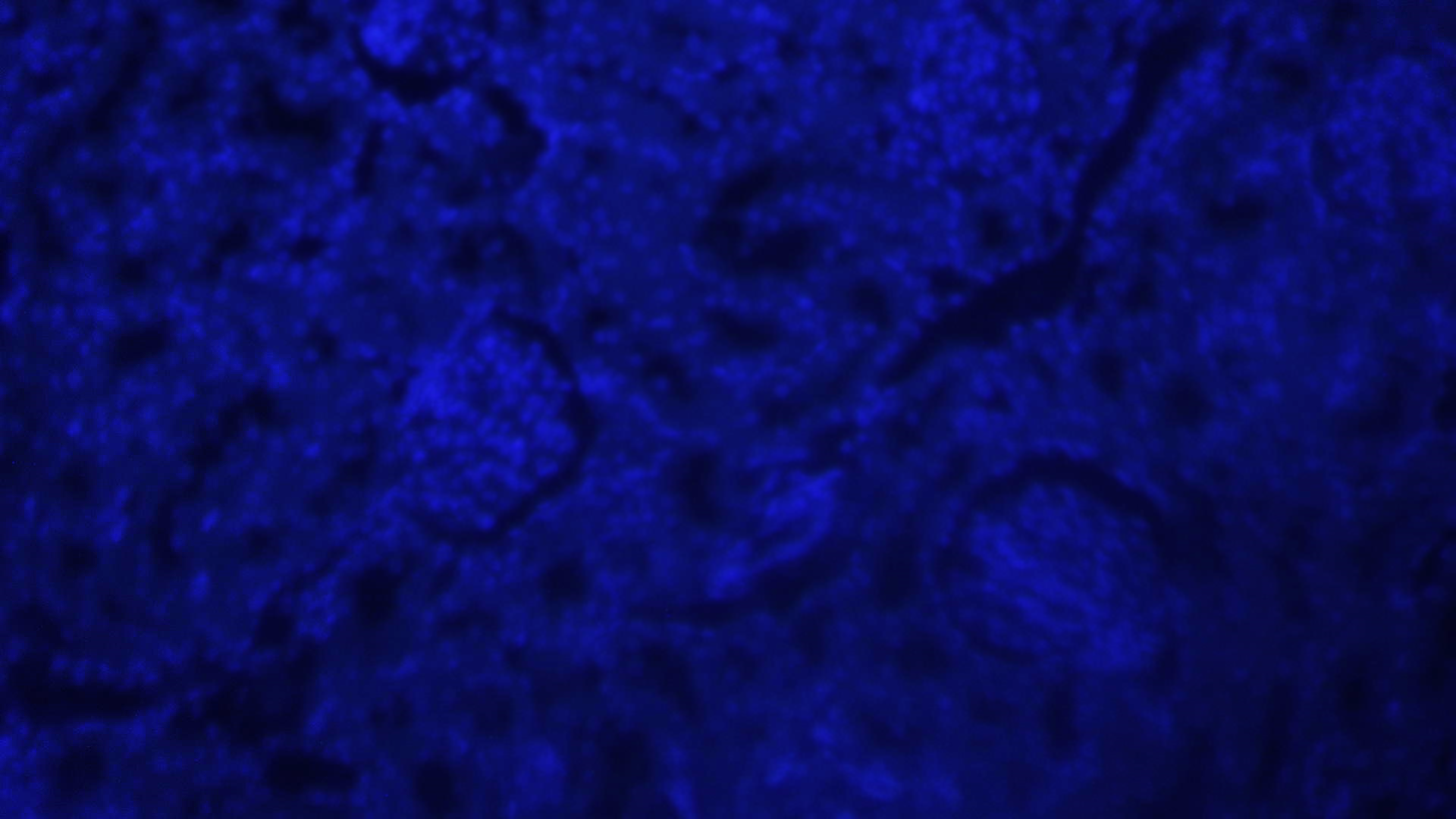

Supplement: Supplementary file 1 [file biomedicines-14-01385-s001.zip › biomedicines-4229880_Raw_Images_Figures_7-11.zipw folder/Original microscopy imgesRaw immunofluorescence results of Figures 7, 8, and 9 of the article/LC3A/Ellagic acid/1/1.tif]

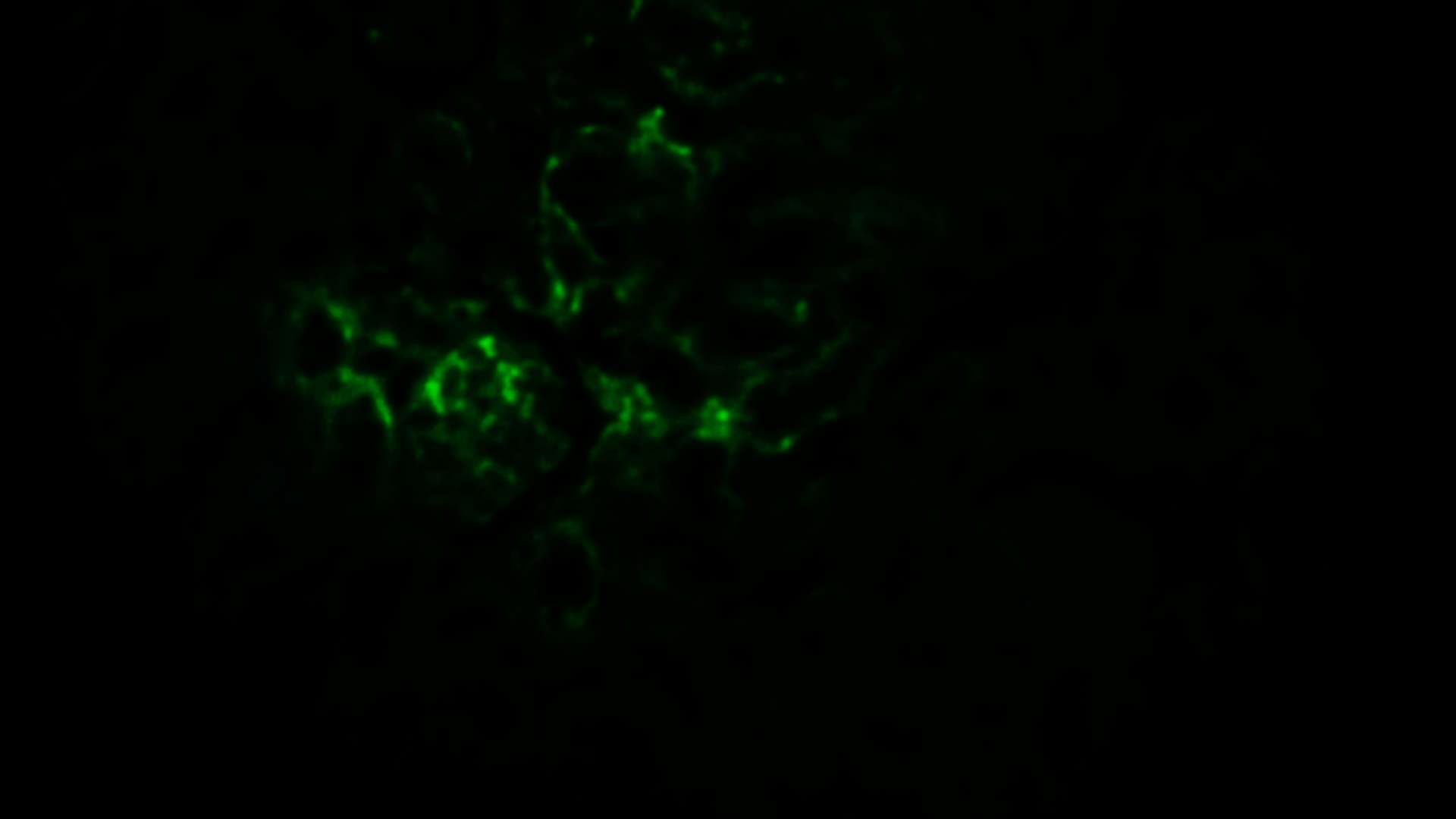

Supplement: Supplementary file 1 [file biomedicines-14-01385-s001.zip › biomedicines-4229880_Raw_Images_Figures_7-11.zipw folder/Original microscopy imgesRaw immunofluorescence results of Figures 7, 8, and 9 of the article/LC3A/Ellagic acid/1/2.tif]

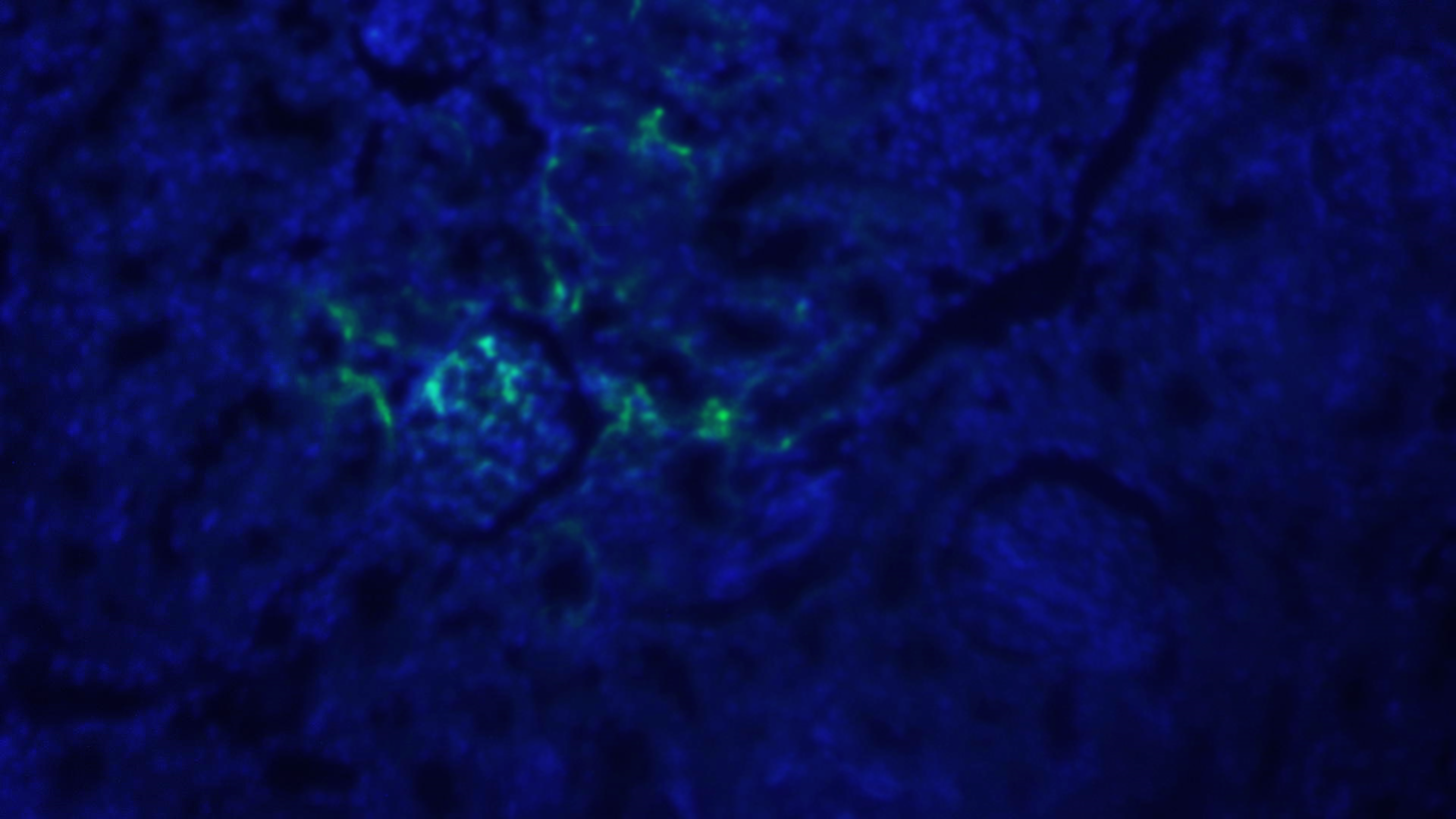

Supplement: Supplementary file 1 [file biomedicines-14-01385-s001.zip › biomedicines-4229880_Raw_Images_Figures_7-11.zipw folder/Original microscopy imgesRaw immunofluorescence results of Figures 7, 8, and 9 of the article/LC3A/Ellagic acid/1/3.tif]

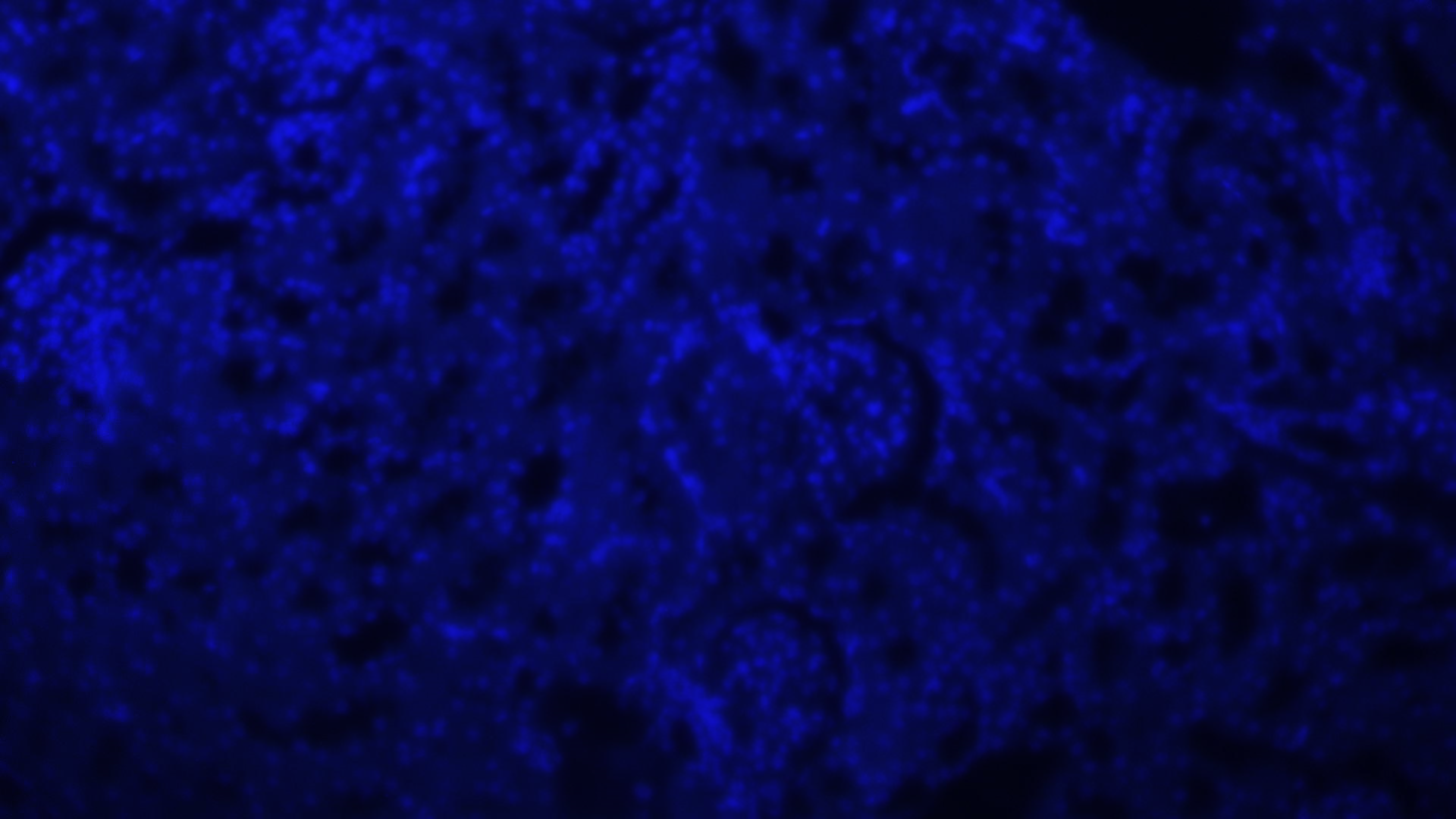

Supplement: Supplementary file 1 [file biomedicines-14-01385-s001.zip › biomedicines-4229880_Raw_Images_Figures_7-11.zipw folder/Original microscopy imgesRaw immunofluorescence results of Figures 7, 8, and 9 of the article/LC3A/Ellagic acid/2/1.tif]

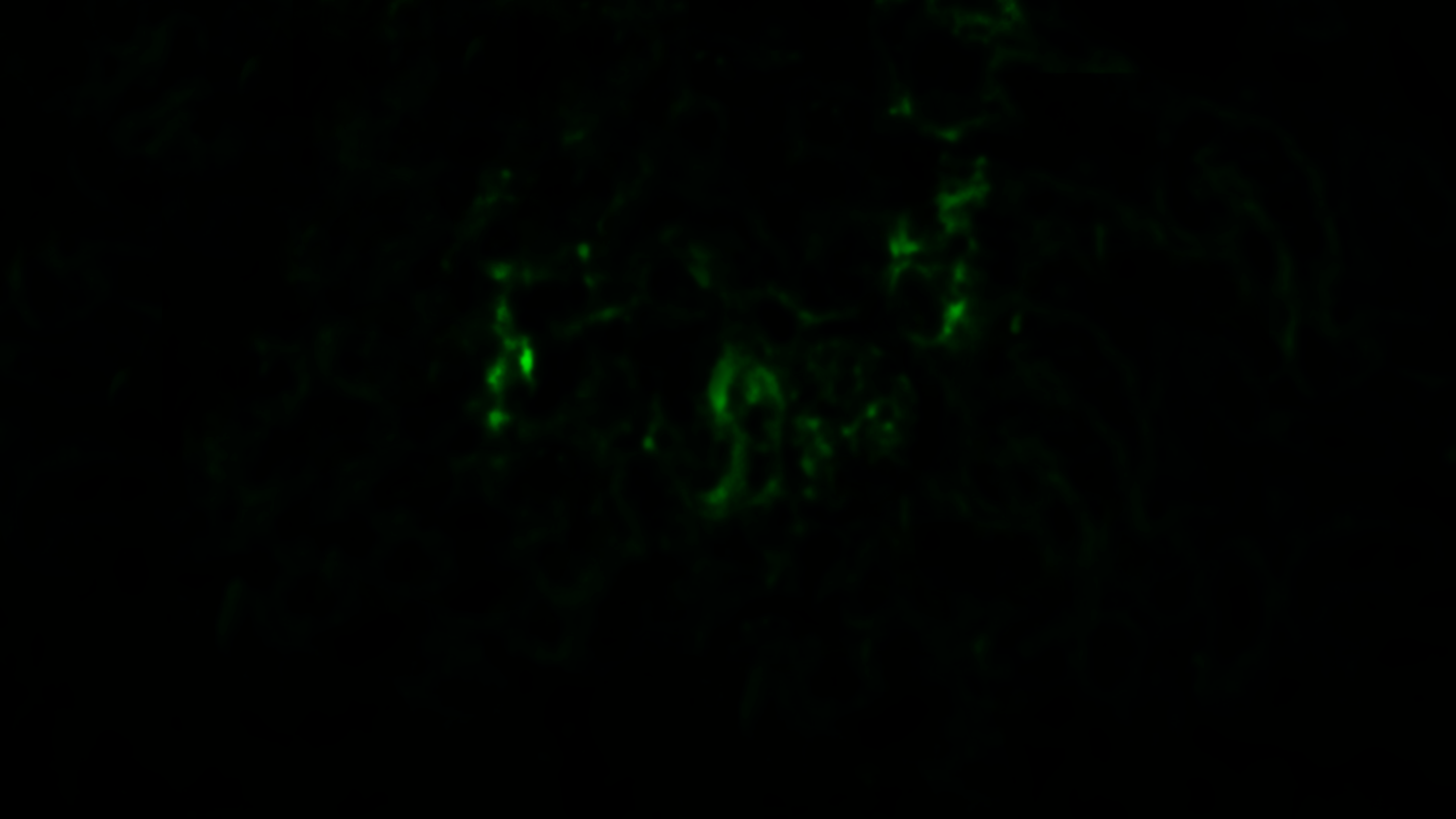

Supplement: Supplementary file 1 [file biomedicines-14-01385-s001.zip › biomedicines-4229880_Raw_Images_Figures_7-11.zipw folder/Original microscopy imgesRaw immunofluorescence results of Figures 7, 8, and 9 of the article/LC3A/Ellagic acid/2/2.tif]

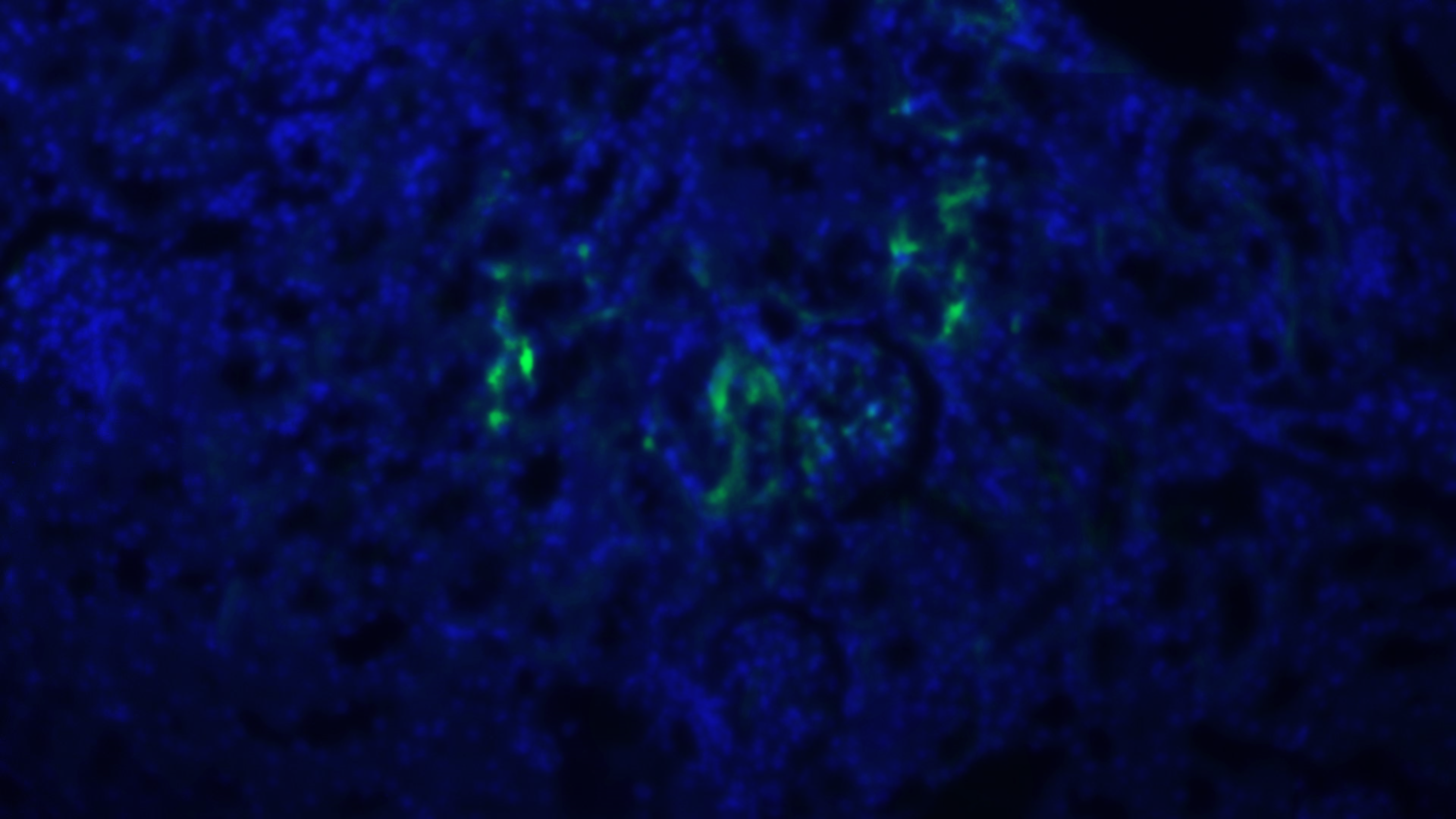

Supplement: Supplementary file 1 [file biomedicines-14-01385-s001.zip › biomedicines-4229880_Raw_Images_Figures_7-11.zipw folder/Original microscopy imgesRaw immunofluorescence results of Figures 7, 8, and 9 of the article/LC3A/Ellagic acid/2/3.tif]

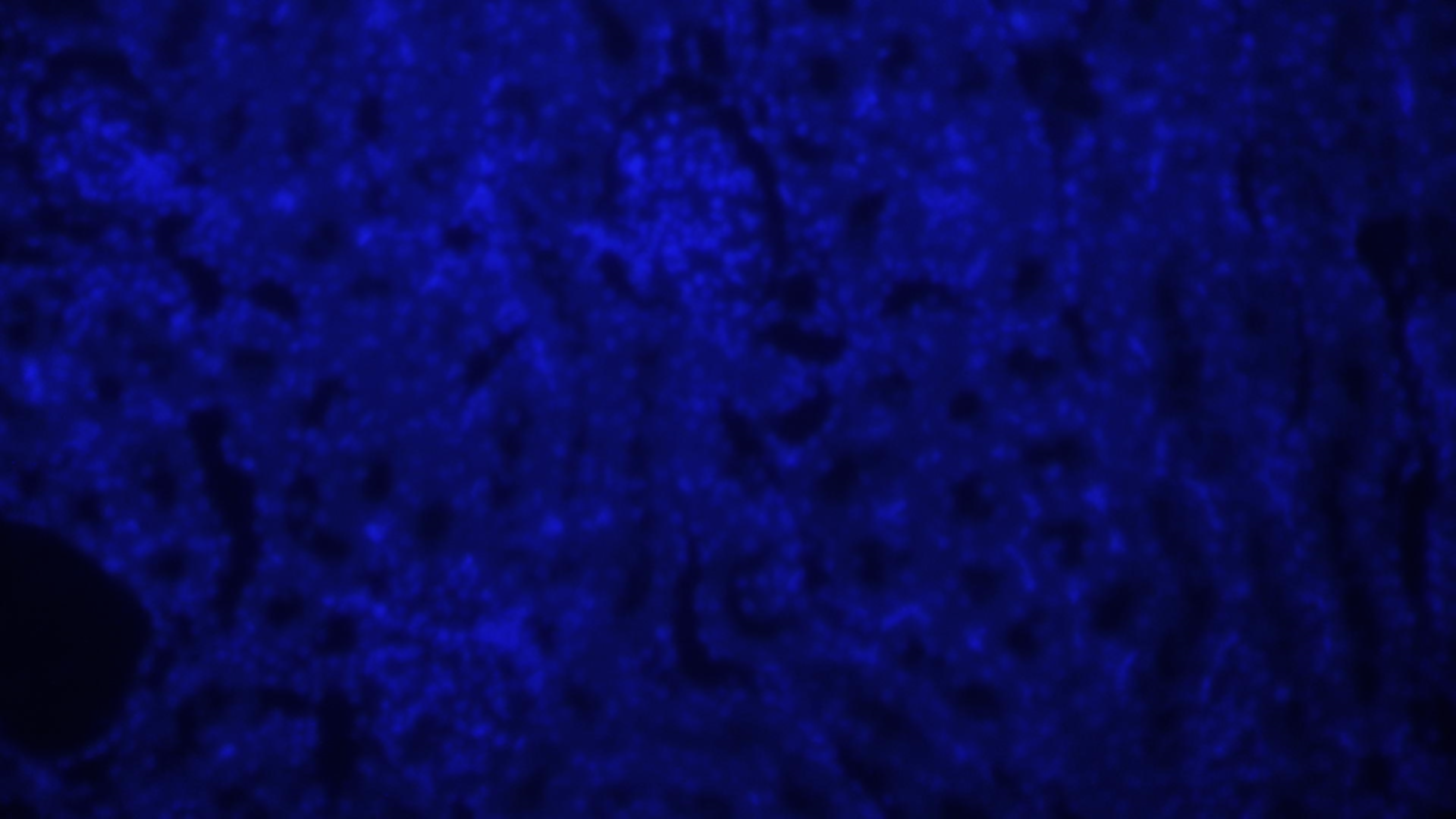

Supplement: Supplementary file 1 [file biomedicines-14-01385-s001.zip › biomedicines-4229880_Raw_Images_Figures_7-11.zipw folder/Original microscopy imgesRaw immunofluorescence results of Figures 7, 8, and 9 of the article/LC3A/Ellagic acid/3/1.tif]

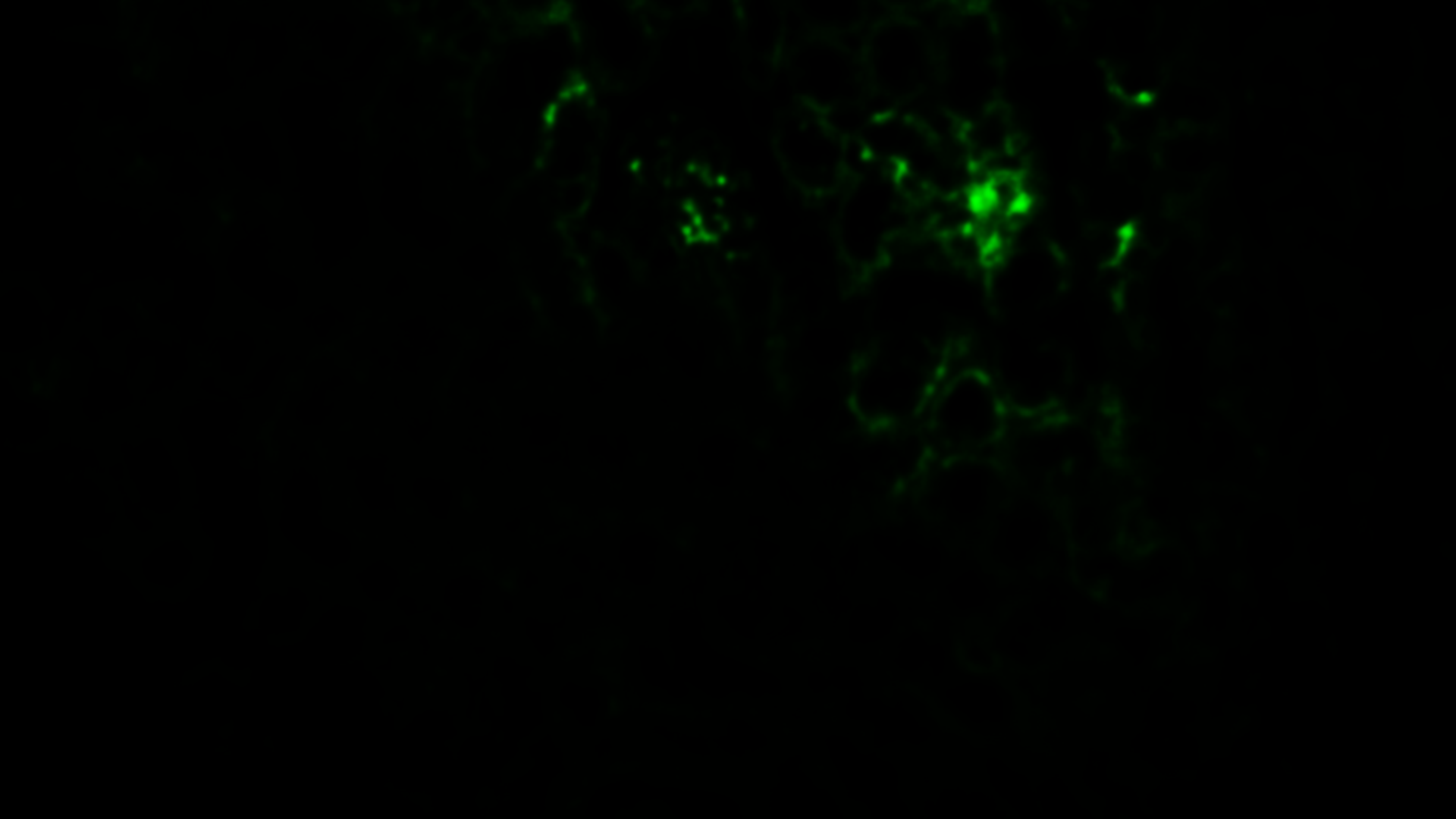

Supplement: Supplementary file 1 [file biomedicines-14-01385-s001.zip › biomedicines-4229880_Raw_Images_Figures_7-11.zipw folder/Original microscopy imgesRaw immunofluorescence results of Figures 7, 8, and 9 of the article/LC3A/Ellagic acid/3/2.tif]

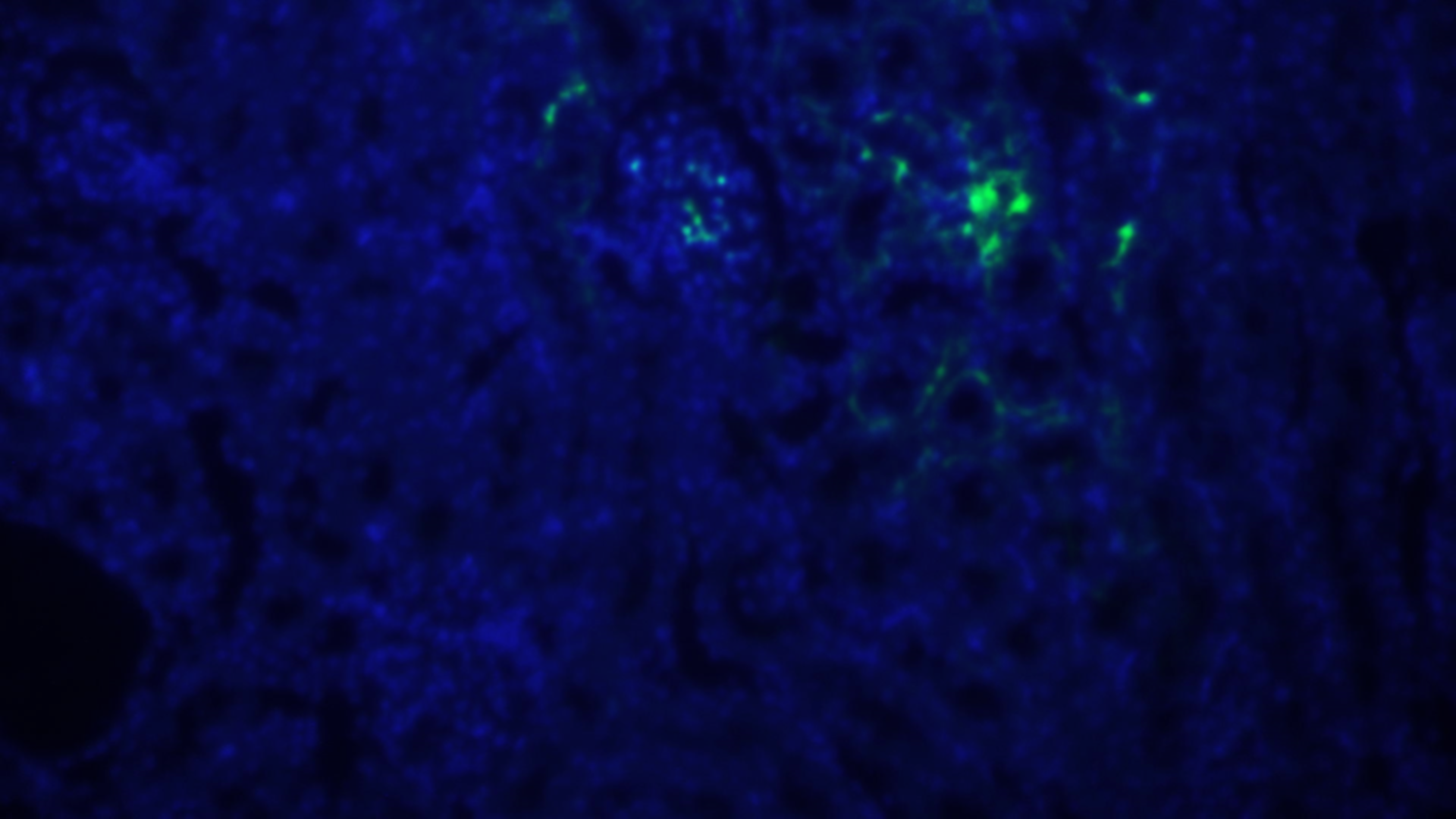

Supplement: Supplementary file 1 [file biomedicines-14-01385-s001.zip › biomedicines-4229880_Raw_Images_Figures_7-11.zipw folder/Original microscopy imgesRaw immunofluorescence results of Figures 7, 8, and 9 of the article/LC3A/Ellagic acid/3/3.tif]

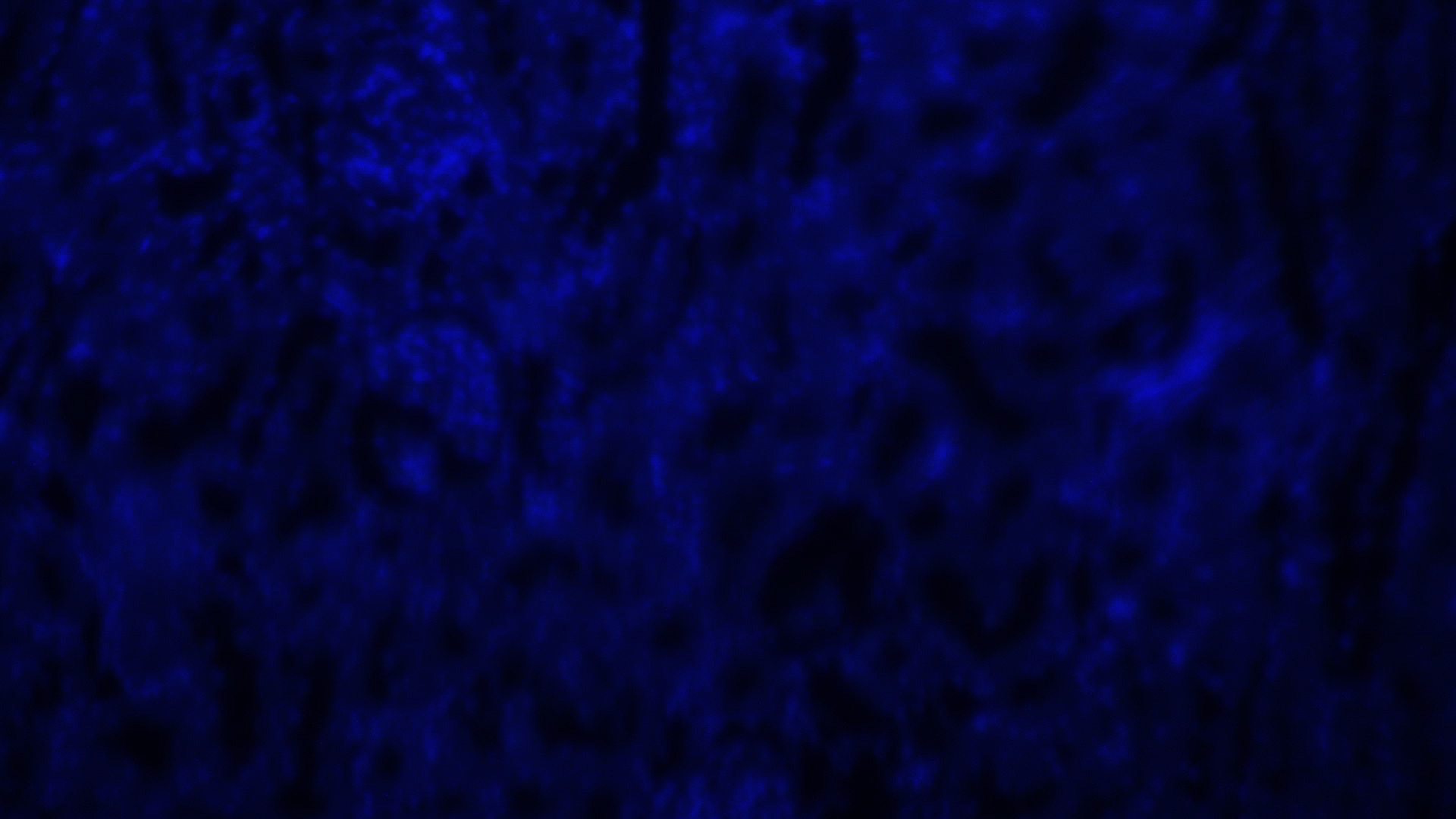

Supplement: Supplementary file 1 [file biomedicines-14-01385-s001.zip › biomedicines-4229880_Raw_Images_Figures_7-11.zipw folder/Original microscopy imgesRaw immunofluorescence results of Figures 7, 8, and 9 of the article/LC3A/Genta+Ellagic acid/1/1.tif]

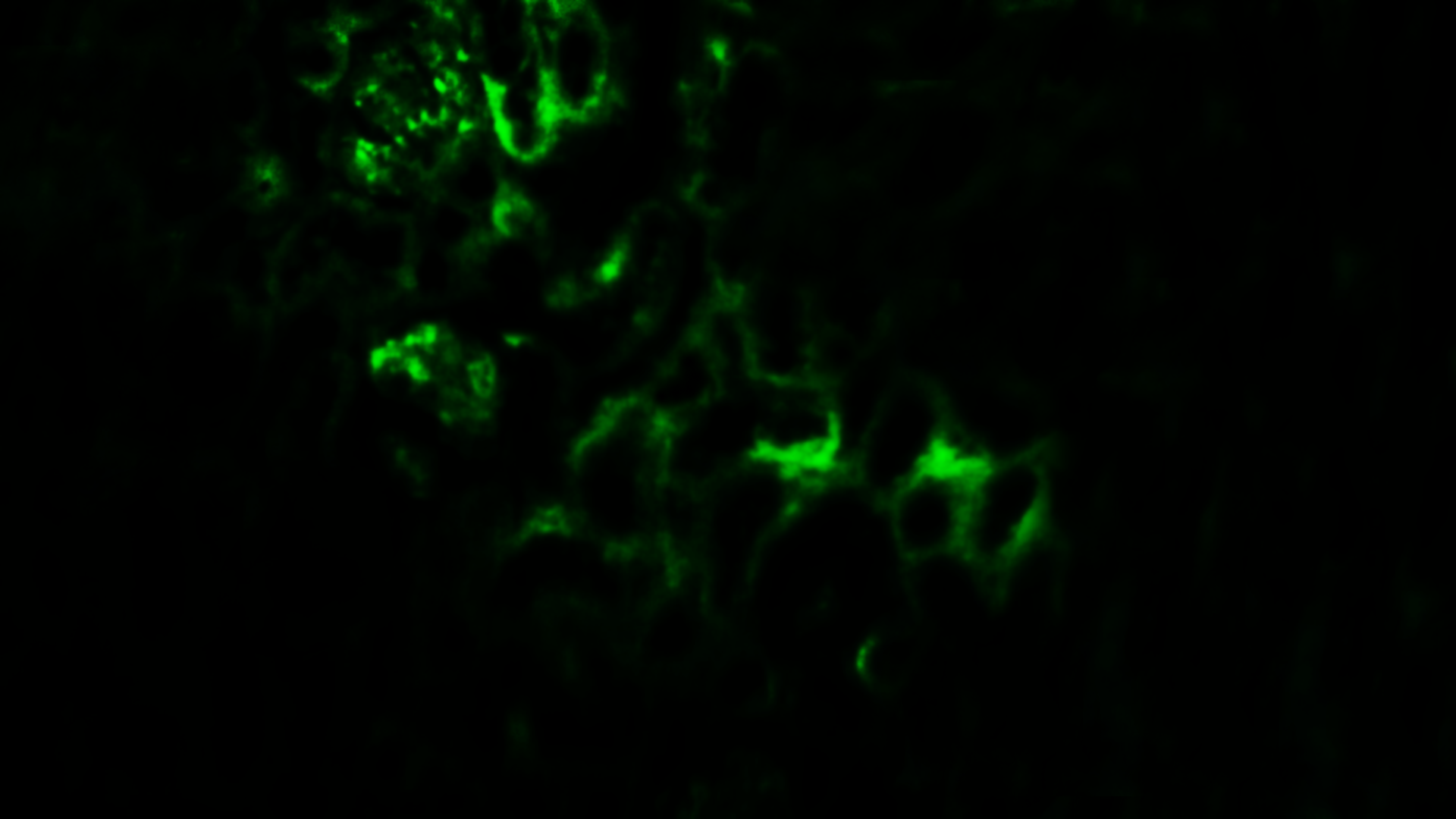

Supplement: Supplementary file 1 [file biomedicines-14-01385-s001.zip › biomedicines-4229880_Raw_Images_Figures_7-11.zipw folder/Original microscopy imgesRaw immunofluorescence results of Figures 7, 8, and 9 of the article/LC3A/Genta+Ellagic acid/1/2.tif]

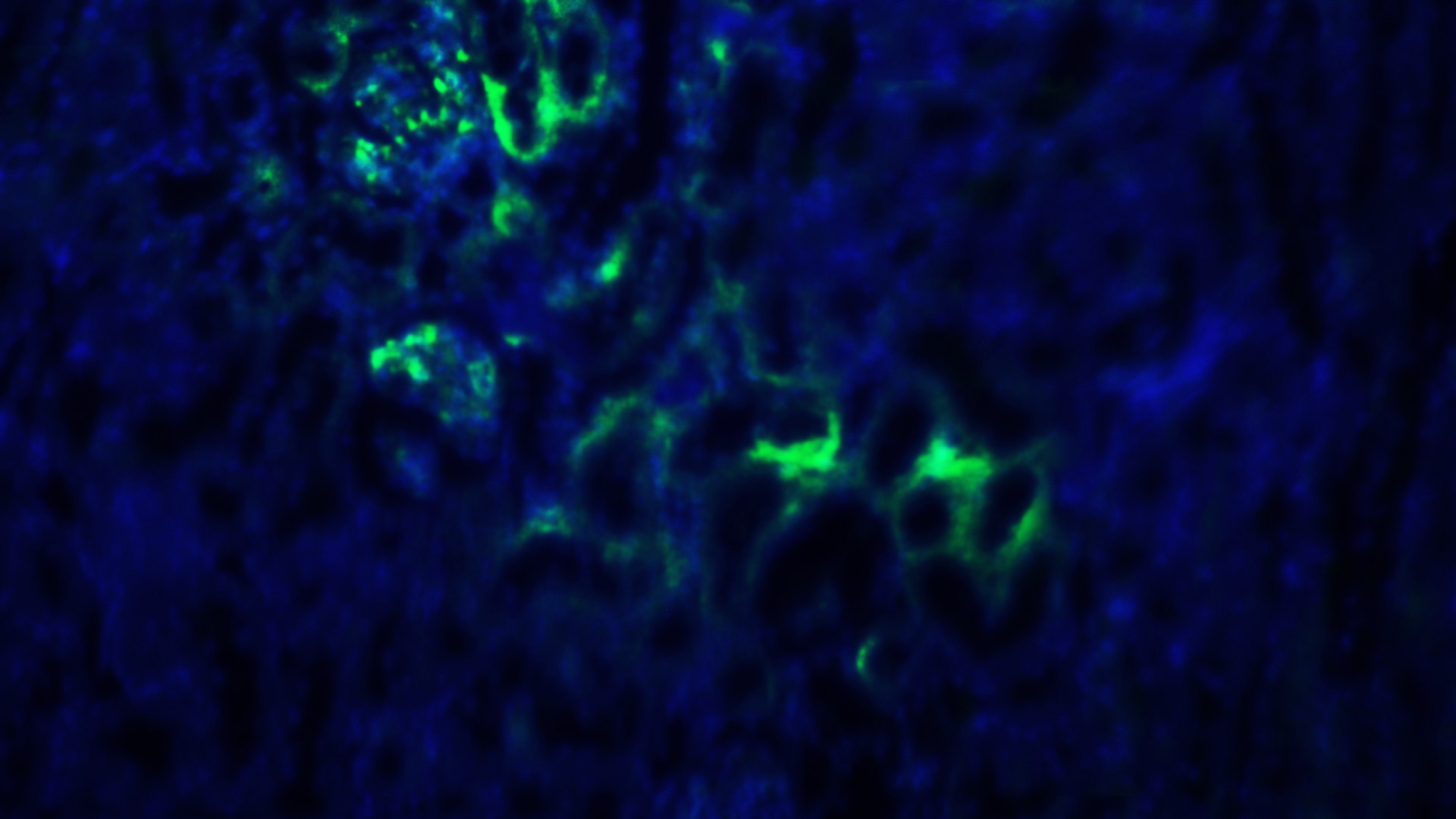

Supplement: Supplementary file 1 [file biomedicines-14-01385-s001.zip › biomedicines-4229880_Raw_Images_Figures_7-11.zipw folder/Original microscopy imgesRaw immunofluorescence results of Figures 7, 8, and 9 of the article/LC3A/Genta+Ellagic acid/1/3.tif]

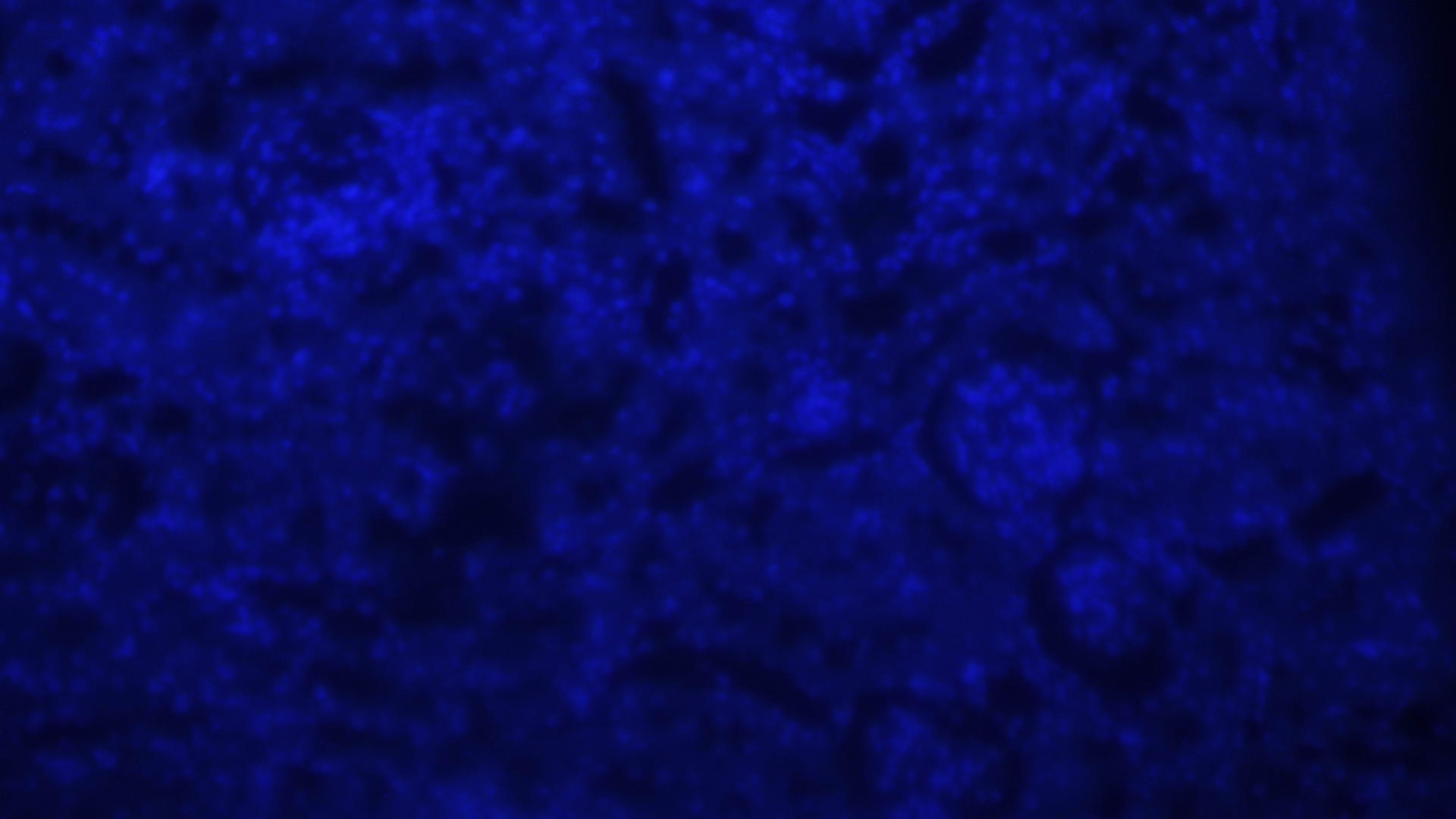

Supplement: Supplementary file 1 [file biomedicines-14-01385-s001.zip › biomedicines-4229880_Raw_Images_Figures_7-11.zipw folder/Original microscopy imgesRaw immunofluorescence results of Figures 7, 8, and 9 of the article/LC3A/Genta+Ellagic acid/2/1.tif]

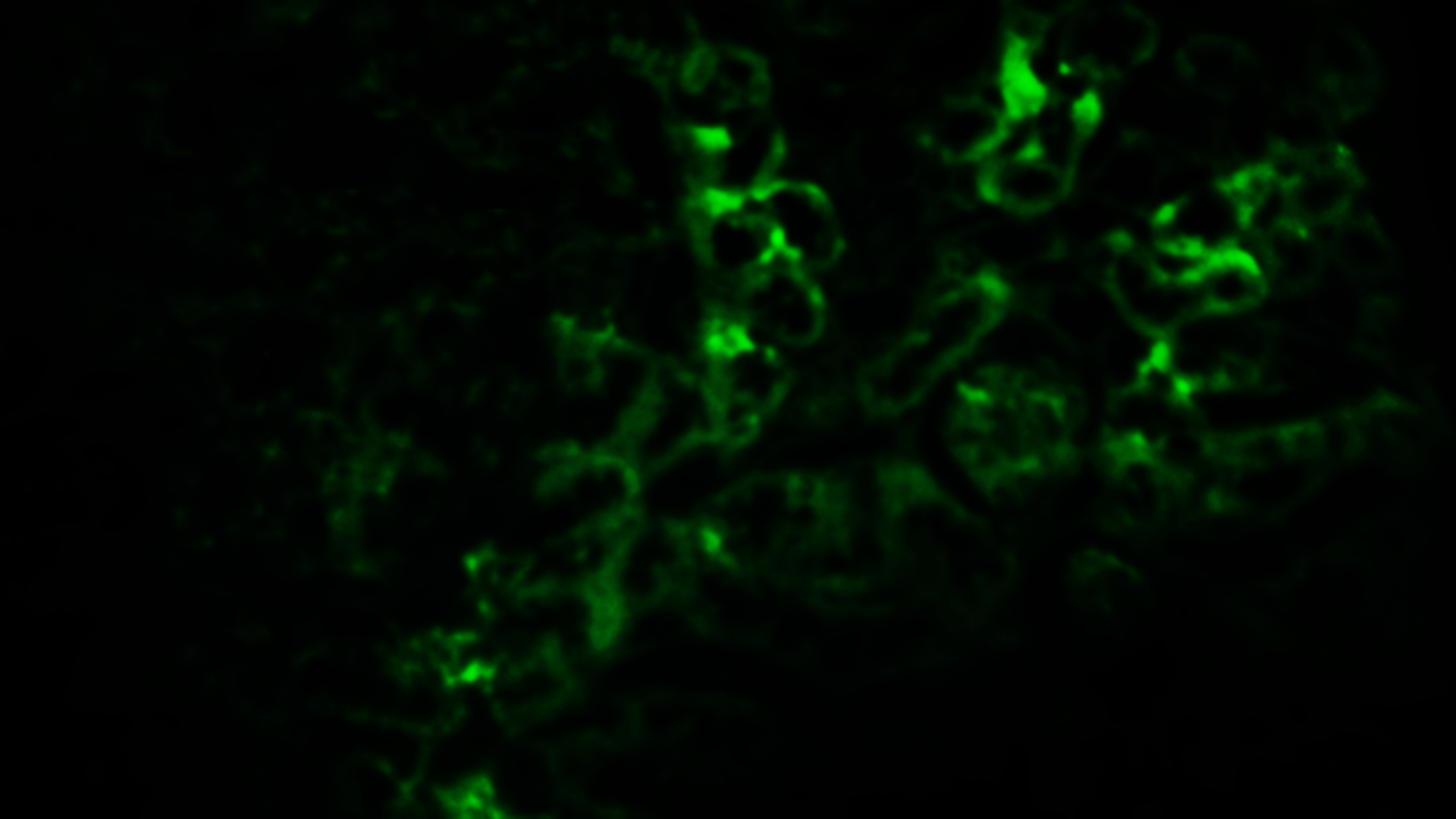

Supplement: Supplementary file 1 [file biomedicines-14-01385-s001.zip › biomedicines-4229880_Raw_Images_Figures_7-11.zipw folder/Original microscopy imgesRaw immunofluorescence results of Figures 7, 8, and 9 of the article/LC3A/Genta+Ellagic acid/2/2.tif]

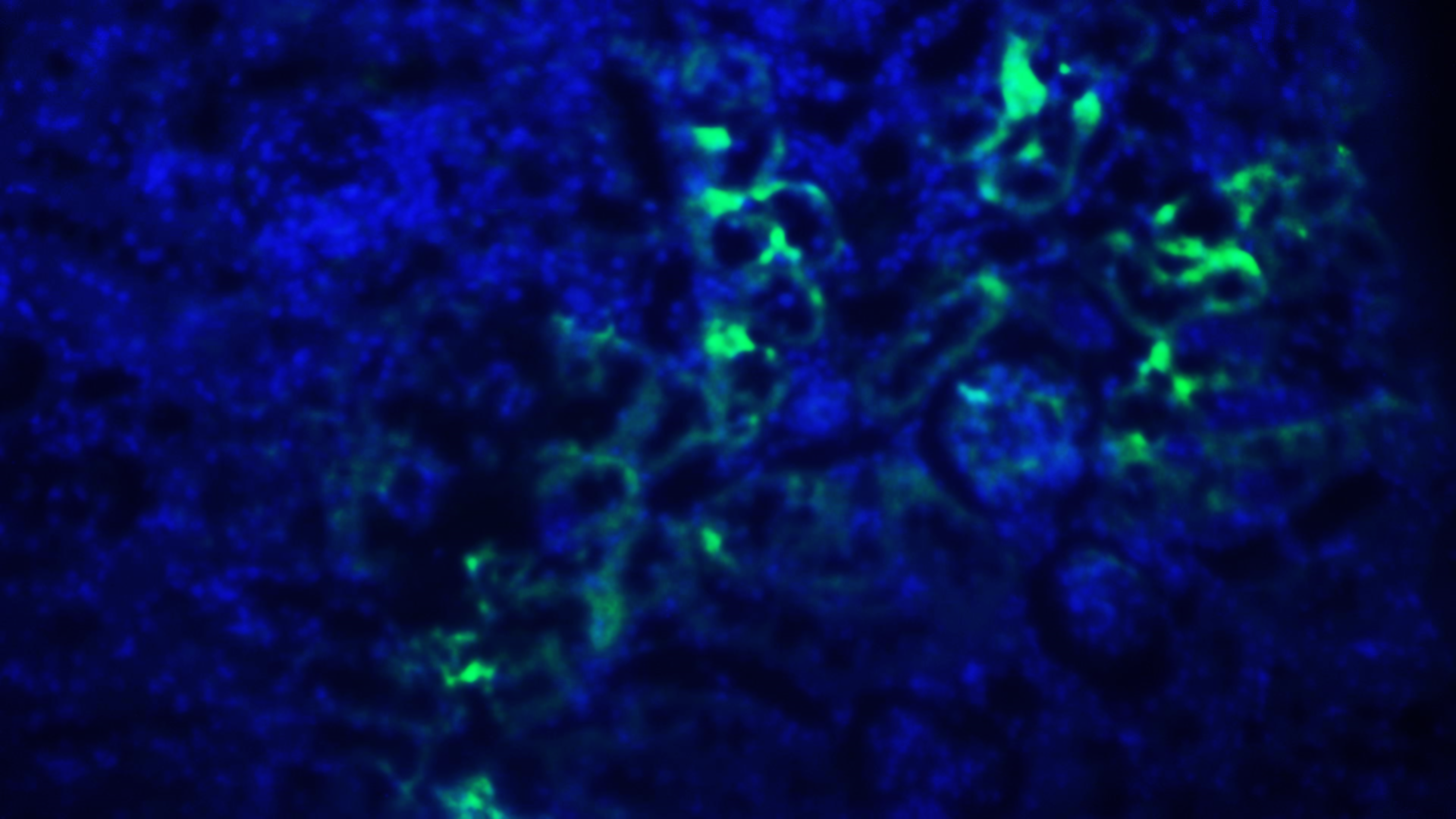

Supplement: Supplementary file 1 [file biomedicines-14-01385-s001.zip › biomedicines-4229880_Raw_Images_Figures_7-11.zipw folder/Original microscopy imgesRaw immunofluorescence results of Figures 7, 8, and 9 of the article/LC3A/Genta+Ellagic acid/2/3.tif]

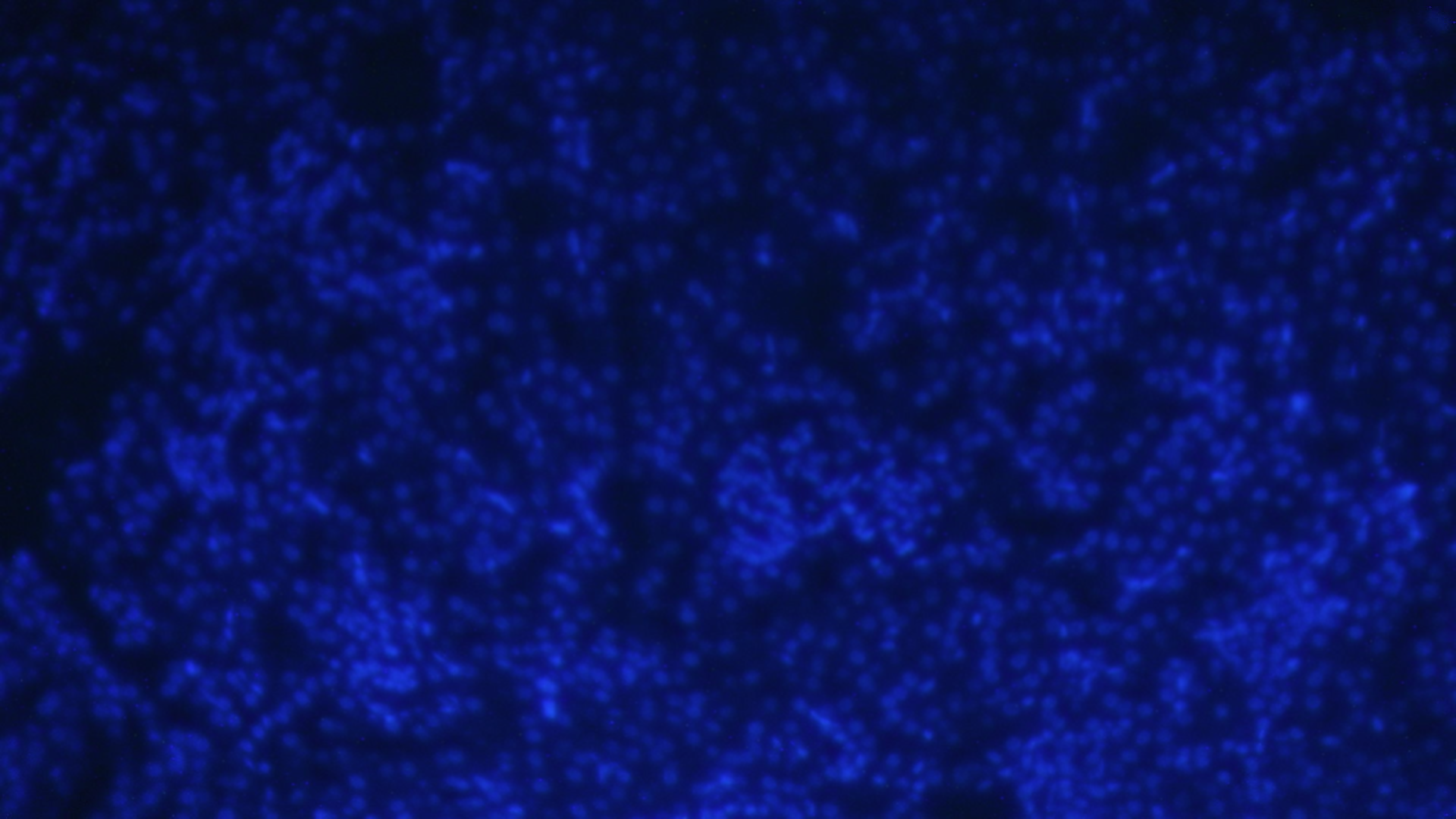

Supplement: Supplementary file 1 [file biomedicines-14-01385-s001.zip › biomedicines-4229880_Raw_Images_Figures_7-11.zipw folder/Original microscopy imgesRaw immunofluorescence results of Figures 7, 8, and 9 of the article/LC3A/Genta+Ellagic acid/3/1.tif]

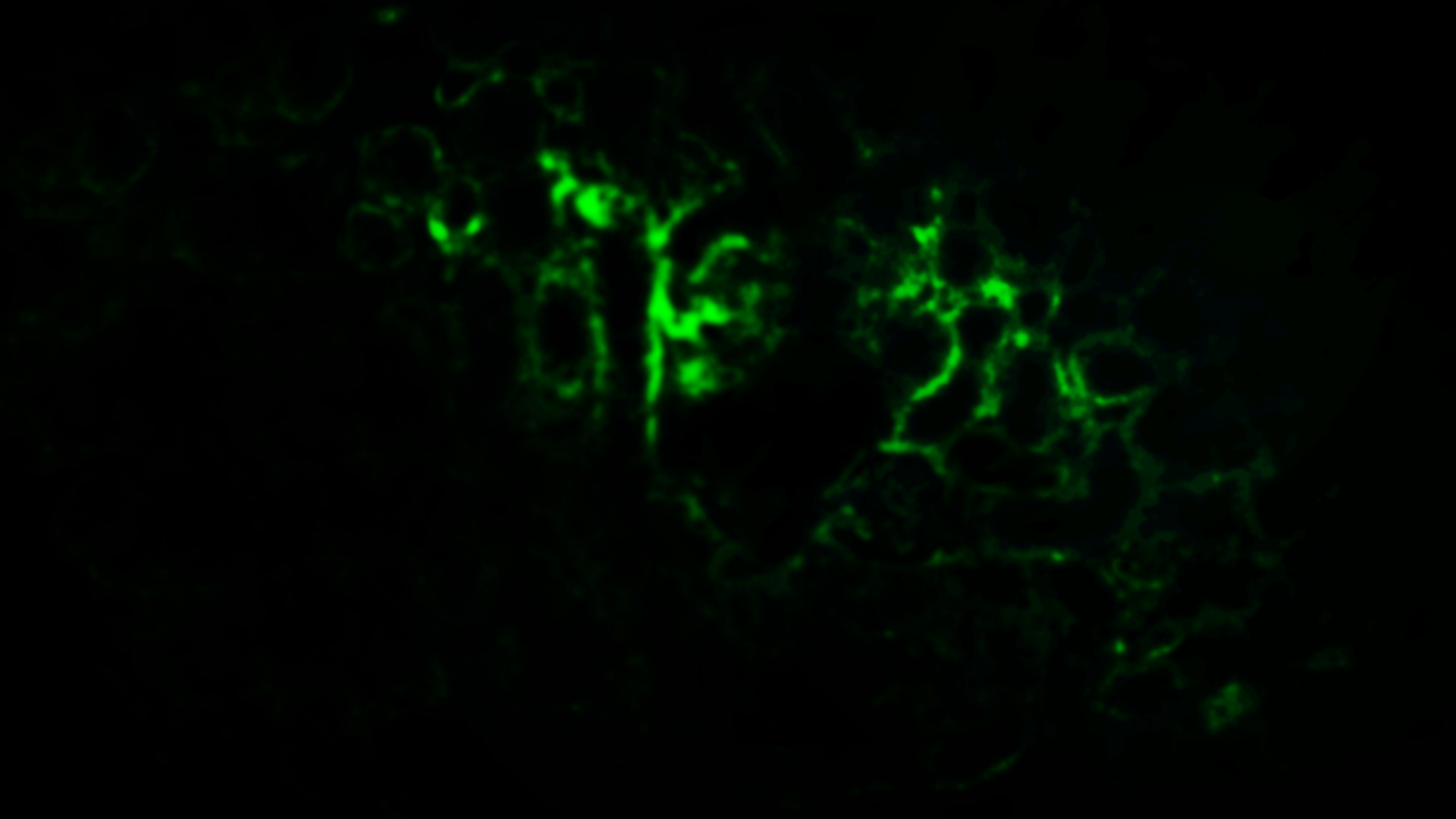

Supplement: Supplementary file 1 [file biomedicines-14-01385-s001.zip › biomedicines-4229880_Raw_Images_Figures_7-11.zipw folder/Original microscopy imgesRaw immunofluorescence results of Figures 7, 8, and 9 of the article/LC3A/Genta+Ellagic acid/3/2.tif]

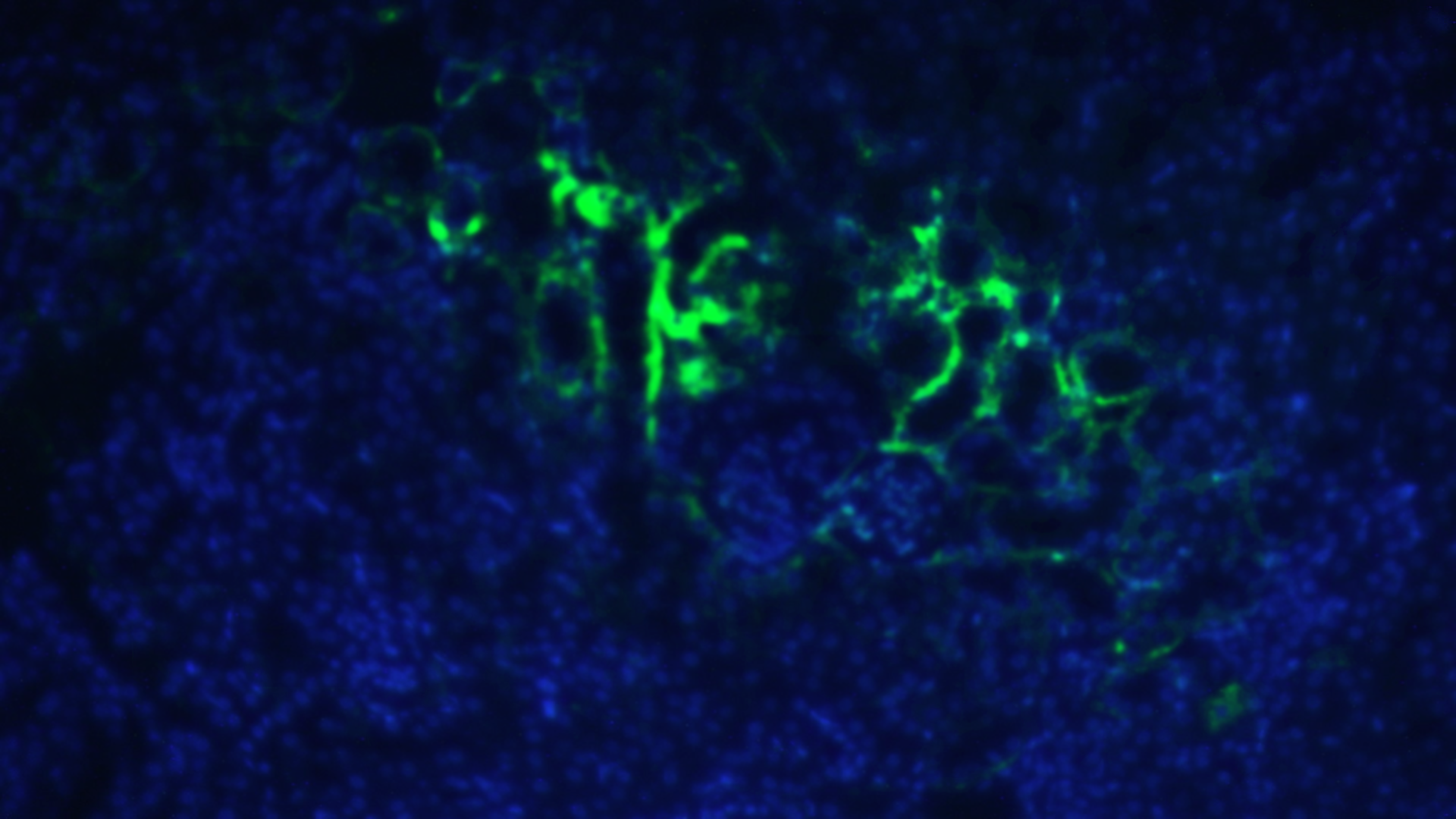

Supplement: Supplementary file 1 [file biomedicines-14-01385-s001.zip › biomedicines-4229880_Raw_Images_Figures_7-11.zipw folder/Original microscopy imgesRaw immunofluorescence results of Figures 7, 8, and 9 of the article/LC3A/Genta+Ellagic acid/3/3.tif]

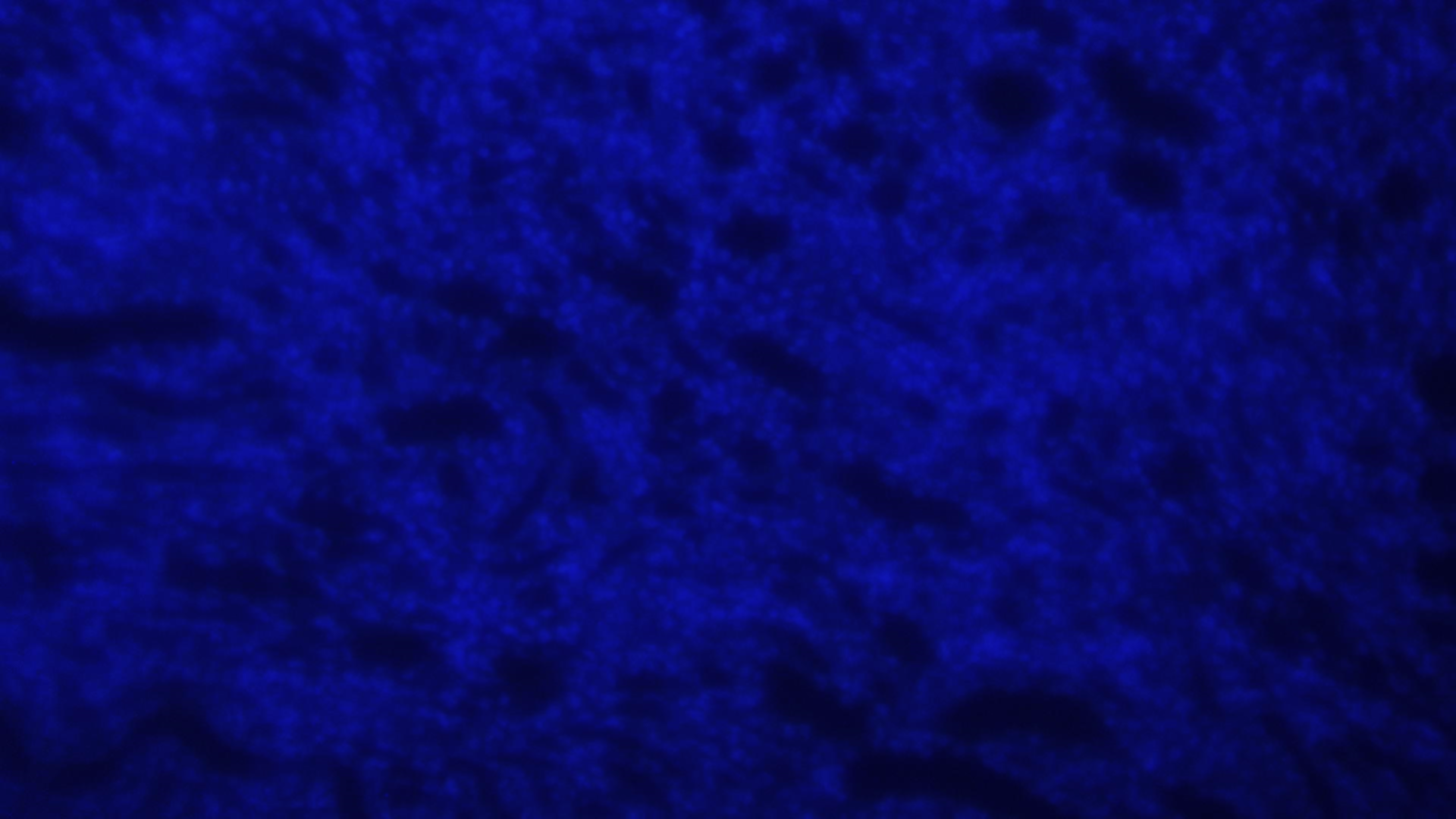

Supplement: Supplementary file 1 [file biomedicines-14-01385-s001.zip › biomedicines-4229880_Raw_Images_Figures_7-11.zipw folder/Original microscopy imgesRaw immunofluorescence results of Figures 7, 8, and 9 of the article/LC3A/Gentamicin/1/1.tif]

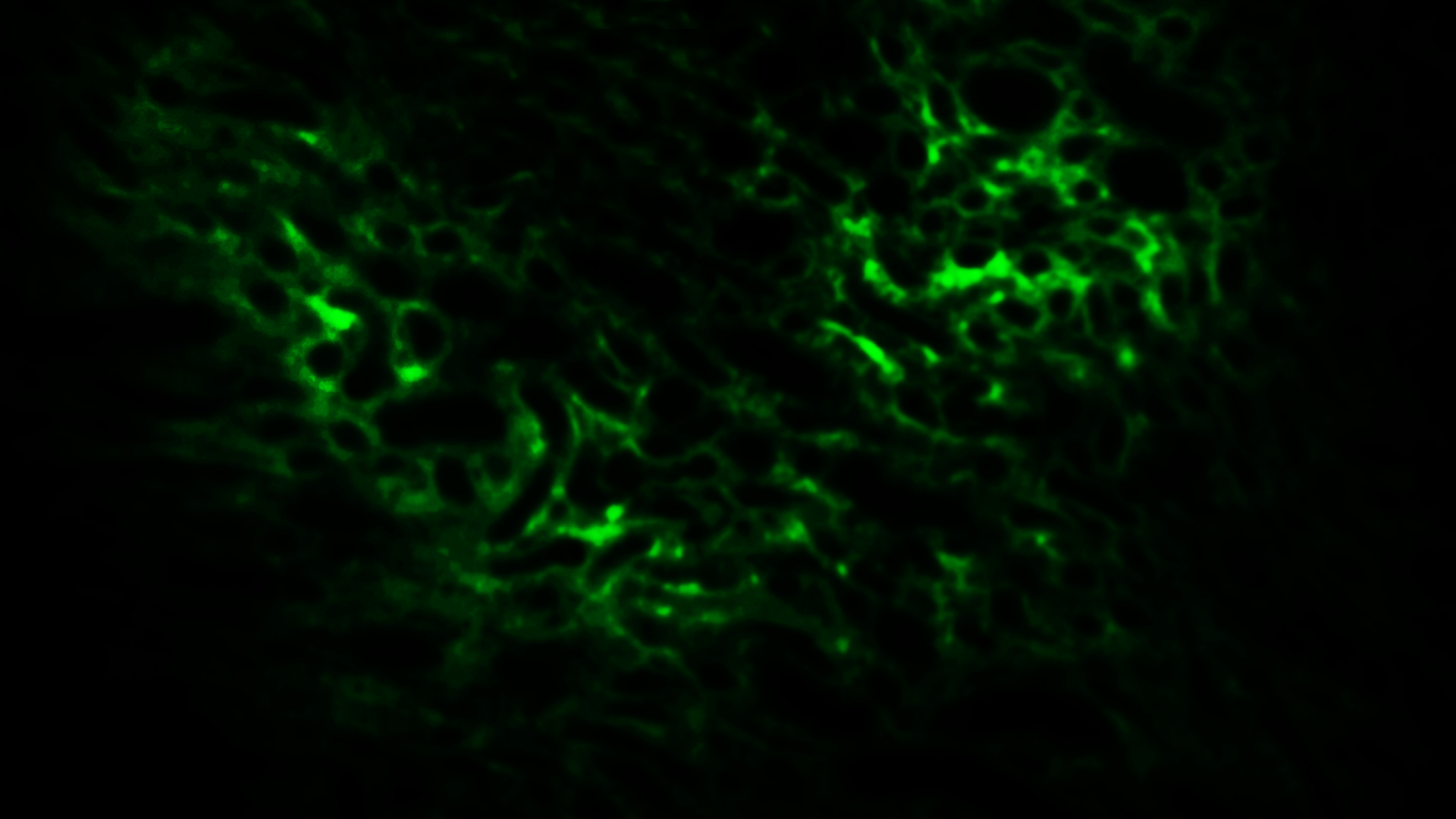

Supplement: Supplementary file 1 [file biomedicines-14-01385-s001.zip › biomedicines-4229880_Raw_Images_Figures_7-11.zipw folder/Original microscopy imgesRaw immunofluorescence results of Figures 7, 8, and 9 of the article/LC3A/Gentamicin/1/2.tif]

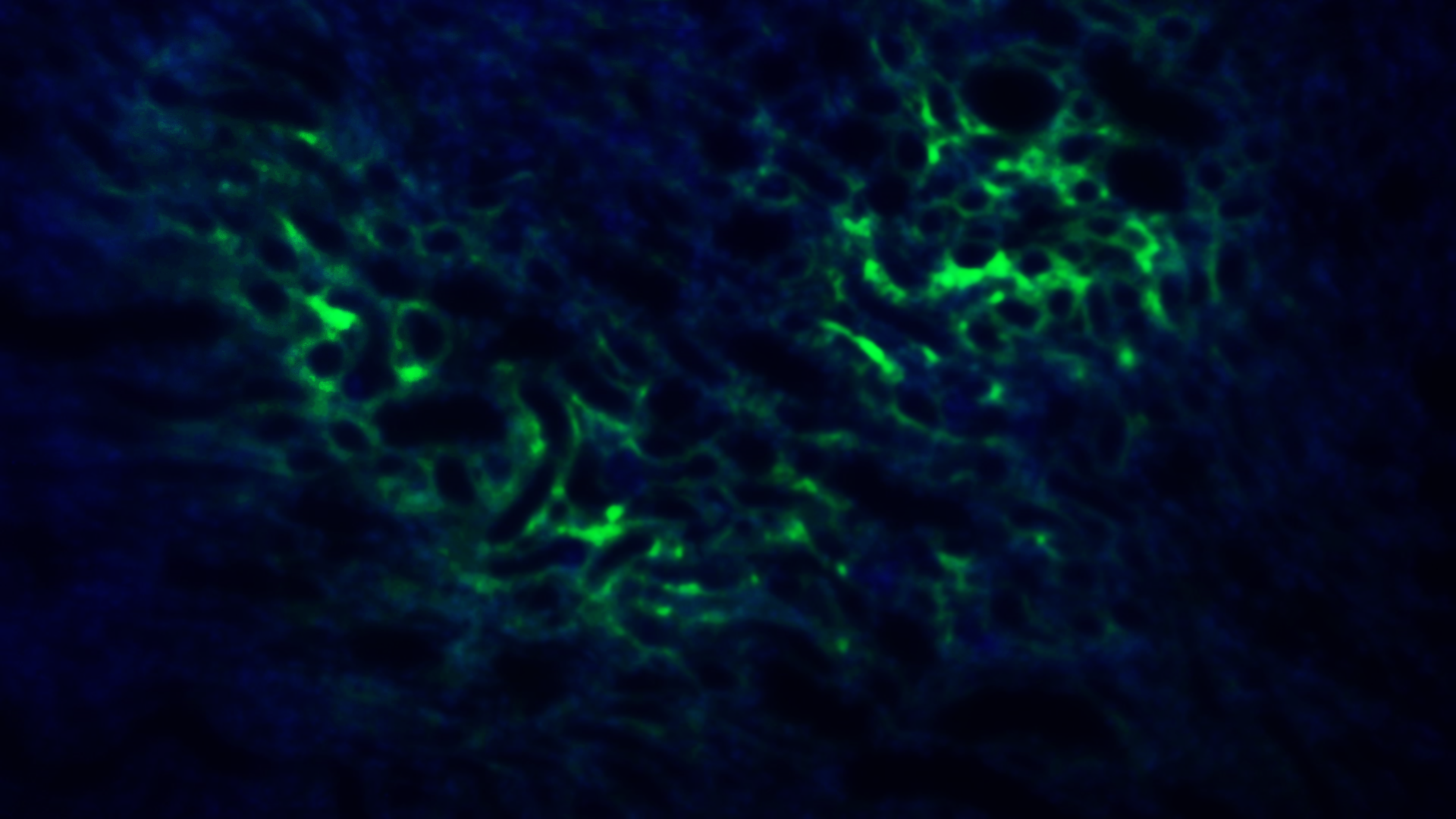

Supplement: Supplementary file 1 [file biomedicines-14-01385-s001.zip › biomedicines-4229880_Raw_Images_Figures_7-11.zipw folder/Original microscopy imgesRaw immunofluorescence results of Figures 7, 8, and 9 of the article/LC3A/Gentamicin/1/3.tif]

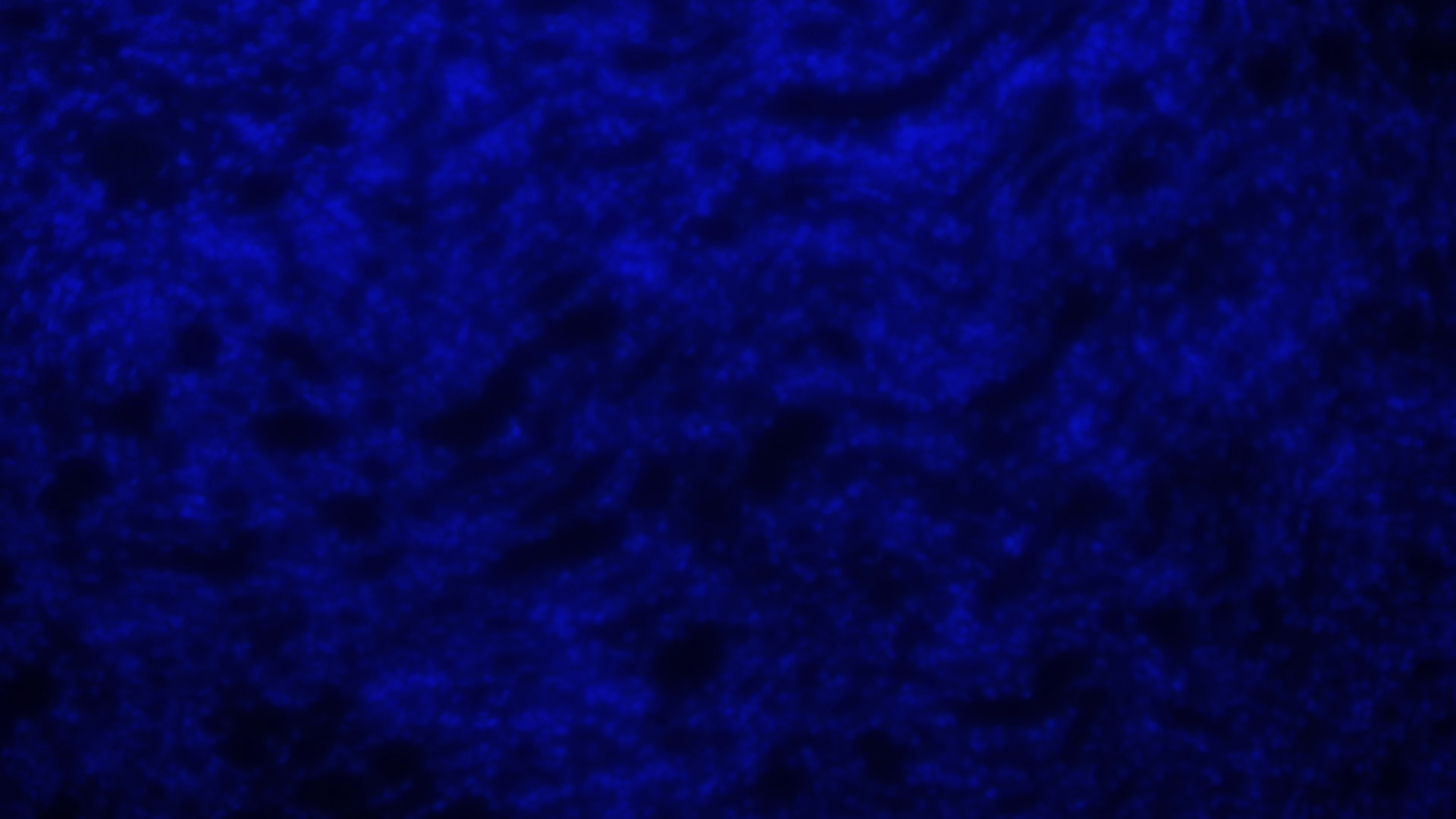

Supplement: Supplementary file 1 [file biomedicines-14-01385-s001.zip › biomedicines-4229880_Raw_Images_Figures_7-11.zipw folder/Original microscopy imgesRaw immunofluorescence results of Figures 7, 8, and 9 of the article/LC3A/Gentamicin/2/1.tif]

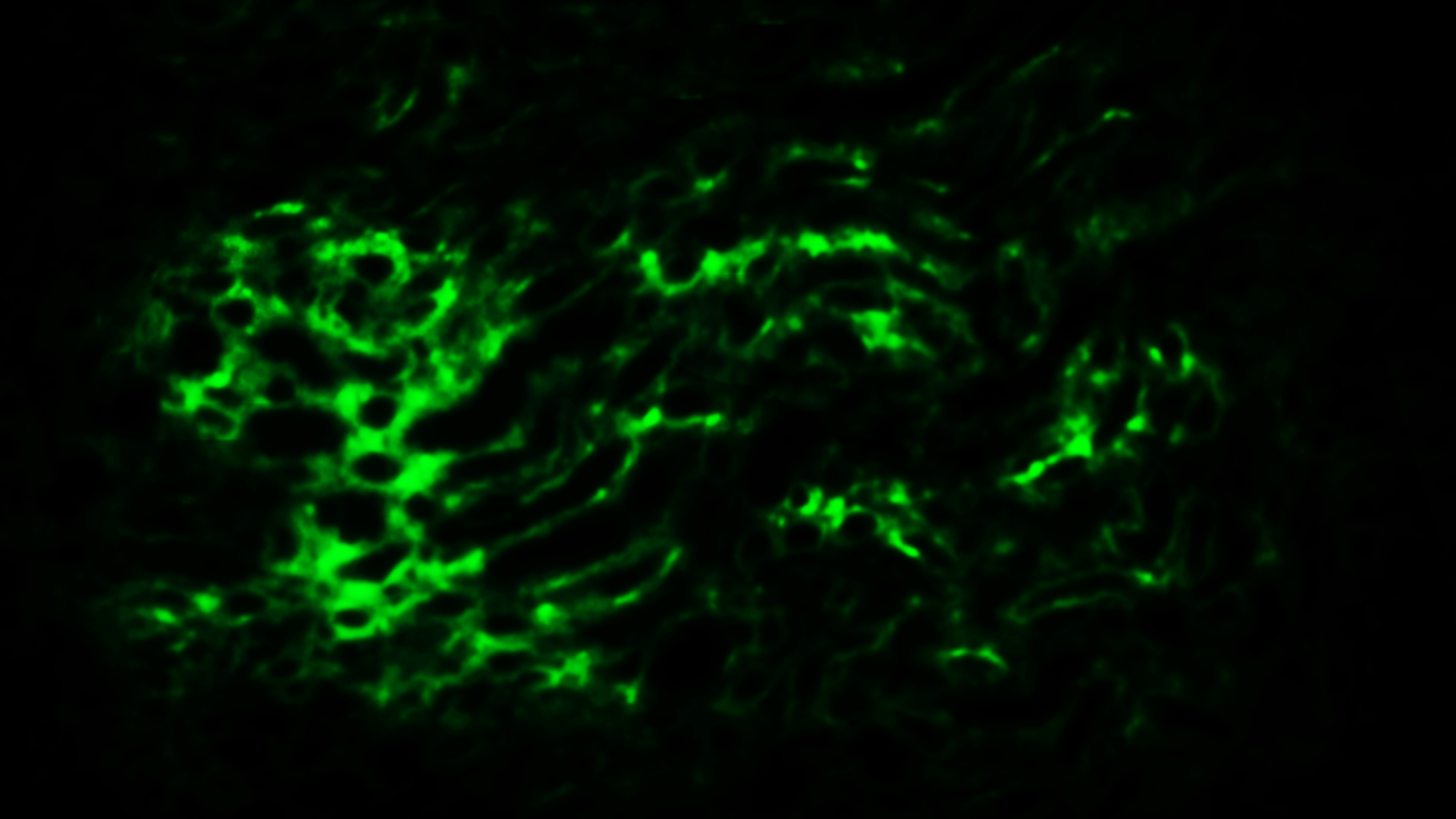

Supplement: Supplementary file 1 [file biomedicines-14-01385-s001.zip › biomedicines-4229880_Raw_Images_Figures_7-11.zipw folder/Original microscopy imgesRaw immunofluorescence results of Figures 7, 8, and 9 of the article/LC3A/Gentamicin/2/2.tif]
